# mHealth-Supported Delivery of an Evidence-Based Family Home-Visiting Intervention in Sierra Leone: Protocol for a Pilot Randomized Controlled Trial

**DOI:** 10.2196/25443

**Published:** 2021-02-02

**Authors:** Alethea Desrosiers, Carolyn Schafer, Rebecca Esliker, Musu Jambai, Theresa S Betancourt

**Affiliations:** 1 Boston College School of Social Work Chestnut Hill, MA United States; 2 University of Makeni Makeni Sierra Leone; 3 Caritas Freetown Freetown Sierra Leone

**Keywords:** mHealth, caregiver mental health, family functioning, early childhood development, community health workers

## Abstract

**Background:**

Past trauma and exposure to violence have been related to poor emotion regulation and household violence, which can have persistent mental health effects across generations. The Family Strengthening Intervention for Early Childhood Development (FSI-ECD/called Sugira Muryango in Rwanda) is an evidence-based behavioral home-visiting intervention to promote caregiver mental health, positive parenting practices, and early childhood development among families facing adversity. In Sierra Leone and other lower- and middle-income countries, mobile health (mHealth) technology has the potential to improve health care delivery and health outcomes.

**Objective:**

This study aims to (1) apply a user-centered design to develop and test mHealth tools to improve supervision and fidelity monitoring of community health workers (CHWs) delivering the FSI-ECD and (2) conduct a pilot randomized controlled trial of the FSI-ECD to assess feasibility, acceptability, and preliminary effects on caregiver mental health, emotion regulation, caregiving behaviors, and family violence in high-risk families with children aged 6-36 months in comparison with control families receiving standard care.

**Methods:**

We will recruit and enroll CHWs, supervisors, and families with a child aged 6-36 months from community health clinics in Sierra Leone. CHWs and supervisors will participate in 1 problem analysis focus group and 2 user interface/user experience cycles to provide feedback on mHealth tool prototypes. Families will be randomized to mHealth-supported FSI-ECD or standard maternal and child health services. We will collect quantitative data on caregiver mental health, emotion regulation, caregiving behaviors, and family functioning at baseline, postintervention, and 3-month follow up. We will use a mixed methods approach to explore feasibility and acceptability of mHealth tools and the FSI-ECD. Mixed effects linear modeling will assess FSI-ECD effects on caregiver outcomes. Cost-effectiveness analysis will estimate costs across FSI-ECD versus standard care.

**Results:**

Funding for this study was received from the National Institutes of Mental Health on August 17, 2020. Institutional Review Board approval was received on September 4, 2020. Data collection is projected to begin on December 15, 2020.

**Conclusions:**

This study will provide important data on the feasibility, acceptability, and preliminary efficacy of mHealth-supported delivery of an evidence-based family home-visiting intervention in a postconflict LMIC.

**Trial Registration:**

ClinicalTrials.gov NCT04481399; https://clinicaltrials.gov/ct2/show/NCT04481399.

**International Registered Report Identifier (IRRID):**

PRR1-10.2196/25443

## Introduction

### Background

Exposure to war, trauma, and other humanitarian crises can have persistent mental health effects across generations, including intergenerational violence [1-3]. The World Health Organization (WHO) estimates that 35% of women globally report experiencing intimate partner violence in their lifetime, and 75% of children in lower- and middle-income countries (LMICs) experience some form of violent or psychologically damaging discipline at home. Experiencing or witnessing family violence during early childhood increases risks for poor emotion regulation and other psychological problems, including posttraumatic stress disorder, externalizing and internalizing behavioral difficulties, and school problems. In postconflict Sierra Leone, research on the intergenerational impact of the 11-year civil conflict has shown that exposure to violence is related to poor parent/caregiver mental health and harsh parenting practices, which adversely affect child development [4-8]. The 2017 Sierra Leone Multiple Indicator Cluster Survey found that 85% of children aged 3-4 and 67% of those aged 1-2 experience violent discipline [9]. Given that poor caregiver emotion regulation is related to family violence and poor child development outcomes [4,5], evidence-based interventions focused on enhancing caregiver–child interactions (including father/male caregiver involvement), improving caregiver emotion regulation and mental health, and promoting alternatives to harsh discipline practices are urgently needed.

### Evidence-Based Family Strengthening

In prior research among families facing adversity in Rwanda, we developed and evaluated the Family Strengthening Intervention for Early Childhood Development (FSI-ECD/*Sugira Muryango*), a home-visiting behavioral intervention delivered by lay workers [10,11]. The FSI-ECD targets caregiver emotion regulation and caregiver–child interactions as major mechanisms to prevent the intergenerational transmission of emotional and behavioral difficulties related to past trauma. It has demonstrated effectiveness in improving caregiver emotion regulation, reducing family violence, and promoting healthy child development [7,11]. The FSI-ECD is a promising approach for targeting underlying mechanisms linked to poor child outcomes [6,7]. Vital for low-resource settings, it can be delivered feasibly by lay workers with strong supervision and quality improvement cycles. Given the limited health infrastructure in many LMICs, including Sierra Leone [12,13], behavioral interventions that can be delivered by well-trained and supervised lay workers, such as community health workers (CHWs), are a more viable option for implementation and sustainment of evidence-based practices.

To further address critical shortages in the mental health workforce in LMICs, intervention delivery strategies must innovate. In Sierra Leone, new government leadership is pursuing mobile health (mHealth) strategies as means to address significant health care workforce limitations that plague delivery of evidence-based behavioral interventions to vulnerable families. Mobile technology has the potential to transform health care delivery and improve health outcomes in Sierra Leone and other LMICs by providing training, supervision, and fidelity supports to enhance quality improvement while interventions are scaled out, but it has not been widely applied to mental health and family-based prevention, particularly in Sub-Saharan Africa [8,14,15]. mHealth supervision and fidelity monitoring tools could enhance quality of service delivery and expand the reach of evidence-based mental health services to vulnerable families by generating a rapid feedback loop between supervisors and facilitators unconstrained by geographical distances. However, successful implementation of mHealth tools in Sub-Saharan Africa has been limited by dependence on a reliable network connection and electricity [8]. Although 83% of adult Sierra Leoneans have access to a mobile phone, most lack internet access, particularly in rural areas [16]. In this context, innovative use of battery-powered tablets with offline functions and access to cloud storage are logistically feasible and could help improve delivery quality and supervision of CHWs.

### Study Objectives

The current study aims to (1) pilot a culturally adapted version of the FSI-ECD delivered by CHWs to vulnerable Sierra Leonean families with children aged 6-36 months to assess feasibility, acceptability, and preliminary effects of mHealth-supported delivery of the FSI-ECD on caregiver mental health and emotion regulation, caregiver–child interactions, and family violence in comparison to control families who receive standard care with standard supervision; and (2) develop and pilot innovative and cost-effective mHealth tools to support CHW delivery of the FSI-ECD. Development of mHealth tools will employ a user-centered design approach to design, prototype, and test digital tools that incorporate user feedback from supervisors and CHWs at each stage of development. User-centered design grounds the tool/app development process in the needs and preferences of those who will use the tool to make it more user-friendly, acceptable, and suitable to the real-world needs of the user; it creates a sense of engagement and shared ownership that aids adoption of the innovation [17-19]. We will use participatory methods and best practices in the user interface/user experience (UI/UX) design to engage CHWs and supervisors in the iterative development process to ensure that our mHealth tools and strategies meet their needs, align with local technological capacity and health service priorities, and support sustained evidence-based practice. The objectives are to develop mHealth tools for supervision, fidelity monitoring, and training of CHWs in Sierra Leone and to provide supervisors with quick visual data displays on CHW performance to inform quality improvement cycles. We will also conduct a preliminary cost-effectiveness analysis to assess the economic value of the mHealth-supported delivery of the FSI-ECD versus standard care with standard supervision.

## Methods

### Design

**Figure 1:**
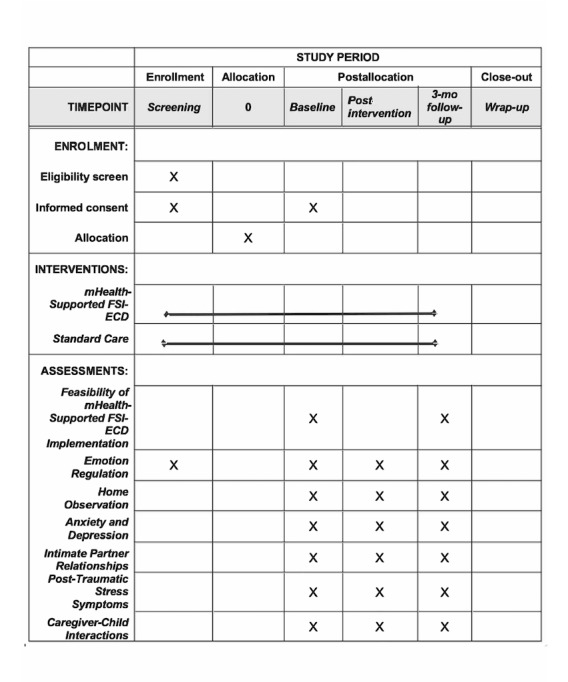
SPIRIT Schedule of Enrollment, Interventions, and Assessments.

This study is approved by the Boston College Institutional Review Board (reference number 21.006.01; Multimedia Appendix 1) and the Sierra Leone Ethics and Scientific Review Committee. The reporting of the trial follows the Standard Protocol Items: Recommendations for Interventional Trials (SPIRIT) guidelines [20] (Figure 1). This trial is registered with the Clinical Trials Registry maintained by the National Library of Medicine at the National Institutes of Health (Trial ID NCT04481399, registered on July 22, 2020). Any subsequent modifications to the study protocol will be reviewed by the Boston College Institutional Review Board and Sierra Leone Ethics and Scientific Review Committee for approval and then submitted to the Clinical Trials Registry as an amendment.

### Overview of Design

**Figure 2:**
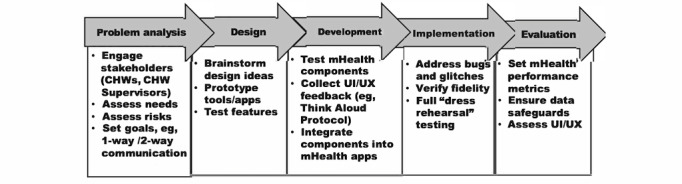
User-centered Design Process for mHealth Tool Development.

We will apply a 5-phase user-centered design approach [21] to develop and test mHealth supervision and fidelity monitoring tools (Figure 2). We will recruit CHWs delivering services to families with children aged 6-36 months (N=6; 3 male/3 female) and CHW supervisors (N=4; 2 male/2 female) to participate in end user focus group discussion sessions. We will hold 3 sessions: an initial problem analysis focus group sessions followed by 2 iterative cycles of UI/UX testing sessions. Problem analysis will seek to understand how CHWs and supervisors might use mHealth tools to enhance fidelity monitoring and supervision and what types of training resources might best support performance. Design of the mHealth tools will be informed by problem analysis findings. Development will test prototyped components of the mHealth tools to integrate audio, replay, visual displays of data, and summary features. UI/UX testing sessions will use Think Aloud Testing Protocols [22], where participants are instructed to “think aloud” while using mHealth tools to illuminate features that are user friendly versus confusing or hard to use. Assessment of strengths and weaknesses of the mHealth tools and the second round of UI/UX testing will inform further refinements prior to the pilot trial. All sessions will be audiotaped, translated, and transcribed. All UI/UX participants will complete a validated usability scale prior to deployment of the mHealth tools [23].

We will conduct a pilot randomized controlled trial to evaluate preliminary mental health benefits of the mHealth-supported FSI-ECD among vulnerable families (N=80) with children aged 6-36 months in the Makeni City region of Sierra Leone. Study research assistants will seek informed consent from families, CHWs, and supervisors for their participation. Families will be randomized to receive the FSI-ECD or standard maternal and child health services delivered by a CHW with standard supervision. To minimize contamination risk, we will use randomization rules developed in our prior work in Sierra Leone (eg, geographic information system mapping to ensure nonadjacency of control and FSI-ECD families). The randomization allocation sequence will be generated via computer-generated random number list in REDCap. Study research assistants and data analysts will be blinded to participant assignment and will assign participants to study condition based on the randomization allocation sequence. Different CHWs will provide the FSI-ECD and standard care to minimize contamination risks.

### Setting and Participants

Makeni is the largest city in the Northern Province of Sierra Leone. The city is the capital of Bombali District, and is the economic center of the Northern Province. Makeni is the Provincial Headquarters of the Northern Province of Sierra Leone. The total population is 125,970, of which 124,634 live in urban areas and 1336 live in rural areas [24]. The most common forms of employment are agriculture and trade. Krio is the primary language.

Inclusion criteria for CHW participation in problem analysis and UI/UX testing are as follows: currently providing maternal and child health services to families with children aged 6-36 months in the Makeni region, aged 18 or older, and ability to attend three 90-minute sessions. Inclusion criteria for supervisors are as follows: currently providing supervision to CHWs delivering the aforementioned services in the Makeni region and aged 18 or older. We will exclude individuals who do not meet CHW or supervisor inclusion criteria.

Inclusion criteria for families are as follows: (1) a Sierra Leonean household with cohabitating caregivers (eg, father/mother, mother/grandmother, mother/partner) and child (aged 6-36 months) with both caregivers aged 18 or older; and (2) 1 caregiver scoring at least 62.5 on the Difficulties in Emotion Regulation Scale (DERS). Both caregivers must agree to attend FSI-ECD sessions; however, if 1 caregiver decides to withdraw, the family can still continue to participate. If enrolled families have more than 1 child aged 6-36 months, we will include all eligible children as study participants. We will exclude families who do not meet all inclusion criteria or who experience active family crises (eg, current suicidality or psychosis, serious medical condition, or cognitive impairment as assessed by a study social worker).

We will recruit families from 2 communities within the Makeni region in coordination with the CHW Focal Person, who is the Ministry of Health and Sanitation Community Health Worker Program official responsible for coordinating the work of CHWs and supervisors within peripheral health units. Peripheral health units are key units within the Sierra Leone health care system. They deliver “first-line” care, including prenatal care, routine deliveries, immediate postnatal and neonatal care, community outreach services, routine vaccination, and treatment of childhood illnesses and malnutrition. Peripheral health units maintain records of families in the community who have sought services and we will be able to identify families with a child aged 6-36 months by reviewing their records. We anticipate that engaging at the community level with the peripheral health units will facilitate recruitment and enrollment of our target sample size.

We will recruit CHWs (N=8) and supervisors (N=2) from the 2 identified peripheral health units to deliver the mHealth-supported FSI-ECD and provide weekly supervision. CHW is a volunteer position and there are no educational qualifications that must be met in order to be engaged as a CHW. CHWs and supervisors who participate in problem analysis and UI/UX sessions will be eligible to participate in the FSI-ECD pilot study. Inclusion criteria are CHWs assigned to the peripheral health unit that provides health services in 1 of the 2 communities and 18 years or older. We will exclude CHWs who do not meet inclusion criteria. Inclusion criteria for supervisors are currently overseeing CHWs providing maternal and child health services in 1 of the 2 communities and aged 18 or older. We will exclude supervisors who do not meet inclusion criteria.

### FSI-ECD

The FSI-ECD is composed of 4 core components: (1) developing problem-solving, stress management, and emotion regulation skills; (2) cultivating positive parenting skills and fostering father/male co-caregiver engagement; (3) developing communication and conflict resolution skills; and (4) exploring alternatives to harsh punishment and practicing nonviolent child discipline. The FSI-ECD integrates key elements of the evidence-based Family-Based Prevention Intervention [25] and was culturally adapted to the Rwandan context through extensive community-based participatory research methods involving Rwandan community advisory boards. The FSI-ECD is delivered in 12 modules in the home via coaching by CHWs. Sessions are delivered once per week and last approximately 90 minutes. Prior to the trial, we will adapt the FSI-ECD to the cultural context of Sierra Leone. A Community Advisory Board will advise on local parenting and mental health terms and concepts drawing from previously collected qualitative data on parenting in Sierra Leone.

### Standard Services

Standard CHW care involves 3 home-visiting, educational sessions delivered to families following childbirth, with weekly supervision via phone or face-to-face. Topics of home-visiting sessions include skilled postnatal care for mothers, early initiation of breastfeeding, nutrition, immunization services, handwashing and hygiene practices, building the capacity of family members to take care of newborns and children under age 5. CHWs also conduct screenings for acute malnutrition and growth monitoring to identify early referrals, and they can provide family planning methods; deworming tablets; and other vitamins for acute malnutrition, dehydration, and antimalaria treatment. Each home-visiting session lasts approximately 60 minutes.

### Training and Supervision

CHWs and supervisors will be trained in the core components of the adapted FSI-ECD by FSI-ECD experts. Training will occur 5 days per week over the course of 3 weeks. At the conclusion of training, CHWs and supervisors will complete a competency assessment. CHWs and supervisors will also complete a 1-day technology training on use of the mHealth tools. During FSI-ECD delivery, CHWs and supervisors will participate in weekly 60-minute supervision sessions guided by mHealth tools to support delivery quality. CHWs and supervisors will complete fidelity monitoring checklists that are embedded in mHealth tools, and review of fidelity monitoring data will inform quality improvement feedback cycles during supervision.

### Measures

#### FSI-ECD Outcomes

We will collect quantitative data on caregiver mental health and emotion regulation, harsh parenting practices, the home environment, and family functioning at baseline, postintervention, and 3-month follow-up. All quantitative measures have undergone a thorough development, translation, and validation process [26] in a prior randomized controlled trial in Sierra Leone. The following quantitative measures will be used: the DERS (α=.95) [27], WHO Disability Assessment Schedule (α=.91) [28], the Conflict Tactics Scale (α=.72-.86) [29], Hopkins Symptom Checklist (α=.92) [30], and Post-traumatic Stress Disorder Reaction Index (α=.93) [31]. To assess caregiver–child interactions, we will use the Home Observation for Measurement of the Environment (α=.73) [32] and the Observation of Mother–Child Interaction (α=.83) [33]. We will also collect qualitative data at postintervention via key informant interviews with randomly selected caregivers (4 males/4 females) to assess FSI-ECD feasibility, acceptability, and satisfaction.

#### mHealth Outcomes

We will collect quantitative data on mHealth tool feasibility, acceptability, adoption, and appropriateness with CHWs and supervisors at baseline and postintervention via quantitative scales developed by researchers at Johns Hopkins Bloomberg School of Health [34]. We will track length of time to deliver FSI-ECD content, use of embedded fidelity monitoring and tracking features, and amount of CHW–supervisor contact via tablet, phone, and face-to-face. Fidelity data will include a CHW-completed electronic fidelity checklist designed to support self-monitoring and performance review with supervisors as well as a supervisor-completed electronic fidelity checklist to be completed while reviewing audiotaped FSI-ECD sessions and discussed during supervision. We will also collect data on mHealth tools postintervention via key informant interviews with CHWs (n=8) and supervisors (n=4) to understand usability of audio/video functions for FSI-ECD delivery, supervision, and quality improvement cycles.

Participant diagnostic and assessment data will be collected via tablets and deidentified. All tablets will be encrypted and password protected using a password known only to the research team. All data on the tablet will remain on the tablet until it is connected to Wi-Fi and uploaded to a secure server. Daily quality assurance and data monitoring checks will determine successful upload of the data, which will be backed up to Box, a secure, HIPAA (Health Insurance Portability and Accountability Act)-compliant, cloud-based storage platform, before it is remotely wiped from the tablet.

### Data Analysis

For quantitative data analysis, will use mixed effects linear models to assess the effects of the FSI-ECD on caregiver mental health and emotion regulation, caregiver–child interactions, and parenting practices. These models will account for clustering of families within CHWs delivering services and clustering of outcomes within families across time. If outcomes are skewed and violate the normality assumption for linear models, we will use generalized linear models with a Poisson distribution. We will conduct all analyses on an intent-to-treat basis. Paired *t*-tests (2-tailed) and Wilcoxon signed rank tests will examine postintervention change in quantitative implementation outcomes (ie, feasibility, acceptability, adoption, appropriateness), controlling for baseline scores.

Power calculations for sample size were calculated using the power command in STATA (StataCorp). The proposed pilot study is not powered to detect treatment effects of clinical significance. However, if we assume a standard α level of .05, 80 families with 2 eligible respondents per family on average, and 2 time points, with assumptions of moderate intraclass (within-family) correlation (approximately 0.5), this pilot randomized control trial has power of 0.80 to detect a standardized “medium” effect size of approximately 0.50 [35]. For outcomes with only 1 observation per time point, and using the same assumptions as above, this pilot randomized control trial has power of 0.80 to detect a standardized effect size of approximately 0.6. Multiple imputation will be used to deal with missing data.

Qualitative data analysis of key informant interviews will follow a 3-step analytical strategy derived from thematic content analysis and grounded theory [36,37]. We will use open coding to examine key interview themes (eg, barriers and facilitators to use, feasibility, and acceptability). We will iteratively develop a coding scheme organized by key themes. After we have identified major categories and established a codebook, we will conduct axial coding to link themes in terms of timing, context, and other dimensions. Poor agreement (ie, low κ ratings as scored in MAXQDA [38]) will be grounds for refining the codebook. We will repeat reliability testing until coding is at >80% agreement for all data sources. We will code all data sets in MAXQDA. Mixed methods analysis will synthesize qualitative and quantitative data using embedded quotes and joint display tables [39]. This approach will also be used for qualitative data analysis of key informant interviews with caregivers.

Cost-effectiveness analysis will estimate costs across FSI-ECD versus standard care. We will use budget, expenditure, supervision, and fidelity data to collect implementation, health, and service costs using standard costing methodologies [40]. Costs will include implementation activities (eg, staff and CHW/supervisor trainings, session delivery, supervision) and directly related recurrent or capital items (eg, tablets, tech support, broadband access, travel supplies). Costs of digital tools will be included as a capital item and amortized based on project duration. Service delivery costs will rely on in-country data or standard costs provided by WHO-CHOosing Interventions that are Cost-Effective published costs data. Outcomes will include a functional impairment measure (WHO Disability Assessment Schedule) that can be converted to quality-adjusted life years [41]. We will use standard incremental cost-effectiveness analysis to compare mHealth-supported delivery of the FSI-ECD to standard care and capture marginal variations in costs and effectiveness using incremental cost-effectiveness ratios. Differences in intervention cost will be divided by differences in intervention effectiveness to calculate incremental cost-effectiveness ratios that can be used to understand the cost of the intervention per unit of outcome (cost per quality-adjusted life year). We can compare this to the standard willingness to pay threshold and to alternative programs to determine which programs are relatively more cost-effective.

### Ethical Approval and Consent to Participate

This study received ethical approval from the relevant College Institutional Review Board and the Sierra Leone Scientific Review Committee (Multimedia Appendix 1). All participants provided verbal consent to participate due to low literacy levels. This procedure was approved by both ethics committees.

### Availability of Data And Materials

Data sharing will be in accordance with the NIH Data Sharing Policy and Implementation Guidance and more specifically the “Data Sharing Expectations for National Institute of Mental Health (NIMH)-funded Clinical Trials.” The data generated in this study will be entered into the NIMH Data Archive as required as prescribed by the Notice of Award as well as presented at national or international conferences and published in a timely fashion. All final peer-reviewed manuscripts that arise from this proposal will be submitted to the digital archive PubMed Central. Published data will be available in print or electronically from publishers, subject to subscription or printing charges. Research data that document, support, and validate research findings will be made available after the main findings from the final research data set have been accepted for publication.

## Results

Funding for this study was received from the National Institute of Mental Health on August 17, 2020 (Multimedia Appendix 1). Institutional Review Board approval was received on September 4, 2020. At the time of manuscript submission, the study has not yet initiated baseline data collection. Data collection is projected to begin on December 15, 2020. Table 1 presents information on the timeline of study activities across the 2 years of the project.

**Table 1:** Project activities and timeline.

Quarter		Year 1				Year 2			
		1	2	3	4	1	2	3	4
**AIM 1: mHealth tool/app development**									
	Focus group: UI^a^/UX^b^ participant recruitment	X							
	Problem analysis		X						
	Design and development		X	X					
**AIM 2: FSI-ECD^c^ adaptation and pilot study**									
	FSI-ECD adaptation	X	X						
	CHW^d^ and supervisor recruitment		X						
	CHW and supervisor FSI-ECD training (3 weeks)			X					
	Family recruitment, enrollment, and baseline diagnostics			X					
	FSI-ECD implementation and postintervention evaluation				X	X			
	FSI-ECD 3-month follow-up						X		
	Data analysis and dissemination						X	X	X

^a^UI: user interface.

^b^UX: user experience.

^c^FSI-ECD: Family Strengthening Intervention for Early Childhood Development.

^d^CHW: community health worker.

## Discussion

### Possible Challenges

There are several potential challenges that may arise during study implementation. In Sierra Leone, many caregivers are involved in employment that requires a high level of daily mobility, such as trade and agriculture. Some caregivers travel across districts, regions, or to neighboring countries for several weeks in order to work. In this work context, we may experience some challenges recruiting and retaining caregivers because participation in the FSI-ECD requires caregivers to attend twelve 90-minute sessions delivered once per week. Although the FSI-ECD may provide long-term benefits for caregiver mental health and child development, these benefits may not be a sufficient incentive for study participation. To help address this challenge, we will encourage highly flexible scheduling to accommodate the working hours of caregivers. The home-visiting nature of the FSI-ECD is also intended to improve service access for families with young children and will relieve the burden of traveling to attend services.

Technology literacy levels and potential technical issues that may occur with the mHealth tools could also pose challenges to this study. To address this, we will provide a 1-day technology literacy training on the use of mHealth tools and basic skills with tablet use. We will also provide ongoing technical support to troubleshoot any technical issues. It is possible that poor connectivity may impede rapid resolution of technical issues, because technical assistance will be remote. However, given that CHWs and supervisors will not need to use mHealth tools on a daily basis, our team should be capable of reasonably resolving any issues with enough time to ensure study activities proceed as planned. We will also train the study data manager in use of the mHealth tools to support greater in-country expertise. We will document any technical issues and keep a log of the strategy to resolve them in the event that the same issue is encountered on a subsequent occasion. To address potential difficulties with internet connectivity, we will place a modem for CHWs and supervisors in the peripheral health unit where they are based. Battery-powered tablets with offline functions and access to cloud storage will also ensure that mHealth tools can be feasibly used and data securely stored until connectivity is available.

### Study Strengths

This study has several strengths. We propose to recruit and enroll CHWs and supervisors, government health employees who work in the communities where they reside, to deliver the FSI-ECD. CHWs will likely already have familiarity with many families in their community before study recruitment and enrollment begins, which may facilitate recruitment and engagement of caregivers in study activities. CHWs will also be familiar with the social norms, typical work schedules, and family habits of their community members, which may also help increase engagement and retention of families in study activities.

### Conclusion

This study has the potential to build urgently needed capacity for both delivery of evidence-based mental health services to reduce family violence and harsh parenting practices and for effective use of mHealth strategies to improve lay worker health service delivery. This study will provide important data on feasibility, acceptability, and cost of both mHealth tools and mHealth-supported FSI-ECD. If mHealth tools are feasible, acceptable, and support high-quality FSI-ECD delivery, this platform could be used to improve efficiency and quality of service delivery for other CHW-delivered services in similar settings. The mHealth tools might also help expand the reach of evidence-based mental health services to vulnerable families in more rural areas by generating a more rapid feedback loop between supervisors and CHWs unconstrained by geographical distances. Finally, applying mHealth tools for supervision and quality improvement has the potential to reduce long-term costs associated with traditional modes of fidelity monitoring and supervision, thus enabling greater scalability in a setting with limited behavioral health professionals.

## Appendix

Multimedia Appendix 1 Boston College Institutional Review Board Approval.

## Abbreviations

CHW: community health worker
DERS: Difficulties in Emotion Regulation Scale
FSI-ECD: Family Strengthening Intervention for Early Childhood Development
LMIC: lower- and middle-income country
UI/UX: user interface/user experience
WHO: World Health Organization
