# Correction: mHealth-Supported Delivery of an Evidence-Based Family Home-Visiting Intervention in Sierra Leone: Protocol for a Pilot Randomized Controlled Trial

**DOI:** 10.2196/27711

**Published:** 2021-02-04

**Authors:** Alethea Desrosiers, Carolyn Schafer, Rebecca Esliker, Musu Jambai, Theresa S Betancourt

**Affiliations:** 1 Boston College School of Social Work Chestnut Hill, MA United States; 2 University of Makeni Makeni Sierra Leone; 3 Caritas Freetown Freetown Sierra Leone

In “mHealth-Supported Delivery of an Evidence-Based Family Home-Visiting Intervention in Sierra Leone: Protocol for a Pilot Randomized Controlled Trial” (JMIR Res Protoc 2021;10(2):e25443) the authors noted one error.

The name of the last author in the originally published paper was listed as *"Theresa Betancourt"*. This has been corrected to *"Theresa S Betancourt"*.

The correction will appear in the online version of the paper on the JMIR Publications website on February 4, 2021, together with the publication of this correction notice. Because this was made after submission to PubMed, PubMed Central, and other full-text repositories, the corrected article has also been resubmitted to those repositories.

