# Correction: mHealth-Supported Delivery of an Evidence-Based Family Home-Visiting Intervention in Sierra Leone: Protocol for a Pilot Randomized Controlled Trial

**DOI:** 10.2196/28359

**Published:** 2021-03-10

**Authors:** Alethea Desrosiers, Carolyn Schafer, Rebecca Esliker, Musu Jambai, Theresa S Betancourt

**Affiliations:** 1 Boston College School of Social Work Chestnut Hill, MA United States; 2 University of Makeni Makeni Sierra Leone; 3 Caritas Freetown Freetown Sierra Leone

In “mHealth-Supported Delivery of an Evidence-Based Family Home-Visiting Intervention in Sierra Leone: Protocol for a Pilot Randomized Controlled Trial” (JMIR Res Protoc 2021;10(2):e25443) one error was noted.

In the section “Ethical Approval and Consent to Participate”, the originally published paper read:

This study received ethical approval from the relevant College Institutional Review Board and the Sierra Leone Scientific Review Committee (Multimedia Appendices 1 and 2).

However, there is no Multimedia Appendix 2 in the published paper and this sentence has been corrected to:

This study received ethical approval from the relevant College Institutional Review Board and the Sierra Leone Scientific Review Committee (Multimedia Appendix 1).

The correction will appear in the online version of the paper on the JMIR Publications website on March 10, 2021, together with the publication of this correction notice. Because this was made after submission to PubMed, PubMed Central, and other full-text repositories, the corrected article has also been resubmitted to those repositories.

