# Supplementary material for: mHealth-Supported Delivery of an Evidence-Based Family Home-Visiting Intervention in Sierra Leone: Protocol for a Pilot Randomized Controlled Trial
Source: JMIR Res Protoc. 2021 Feb 2;10(2):e25443. doi: 10.2196/25443 (PMC7946434; doi:10.2196/25443)
Supplement: Multimedia Appendix 1 [file resprot_v10i2e25443_app1.pdf]

BOSTON COLLEGE
Institutional Review Board
Office for Research Protections
Waul House, 2nd Floor
Phone: (617) 552-4778, fax: (617) 552-0498

IRB Protocol Number: 21.006.01

DATE: September 4, 2020

TO: Alethea Desrosiers

FROM: Office of Research Protections

RE: mHealth Tools to Improve Service Delivery Quality of anEvidence-Based Family Home Visiting Intervention to Prevent Family Violence among High RiskFamilies in Sierra Leone

---

Notice of IRB Review and Approval
Full Board Review [Title 45 CFR Part 46.108 (b); 46.109]

The project identified above has been reviewed and approved by the Boston College Institutional Review Board (IRB) for the protection of human subjects in research. The protocol was reviewed by the fully convened IRB in accordance with HHS regulations requiring the IRB to employ additional criteria when reviewing research that does not qualify for an expedited review. The approval is based on the assumption that the materials you submitted to the IRB contain a complete and accurate description of all the ways in which human subjects are involved in your research.

This approval is given with the following standard conditions:

1. You are approved to conduct this research only during the period of approval cited below;
2. You will conduct the research according to the plans and protocol submitted (approved copy enclosed);
3. You will immediately inform the Office for Research Protections of any injuries or adverse research events involving subjects;
4. You will immediately request approval from the IRB of any proposed changes in your research, and you will not initiate any changes until they have been reviewed and approved by the IRB;
5. The IRB has waived the requirement for obtaining the signature as allowed under 45 CFR 46.117. The research presents no more than minimal risk of harm to subjects, and involves no procedures for which written consent is normally required outside of the research context;
6. The IRB has waived the requirement for the documentation of informed consent as allowed under 45 CFR 46.117. The research presents no more than minimal risk of harm to

subjects, and involves no procedures for which written consent is normally required outside of the research context;

7. You will only use the informed consent documents that have the IRB approval dates stamped on them (approved copies enclosed);

8. You will give each research subject a copy of the informed consent document;

9. You may enroll up to 160 participants. You may not enroll more than this number of participants without seeking IRB approval. To do so will be a violation of the conditions of IRB approval and, if federal funding is involved in your project, a matter of non-compliance that we must report to the federal government. This could significantly and negatively impact your research.

10. **You cannot begin recruitment or data collection until you receive approval by the local ethics board and submit it to the IRB.**

Additionally, any research personnel that have not completed an acceptable education/training program should be removed from the project until they have completed the training. When they have completed the training, you must submit a Protocol Amendment form to add their names to the protocol, along with a copy of their education/ training certificate.

NOTE: Protocols are only approved upon the understanding that there are no face to face interactions allowed until the ban is lifted and that all data must be collected completely online or over the phone. If data cannot be collected online or over the phone then the approval will only take effect once the ban is lifted

Boston College and the Office for Research Protections appreciate your efforts to conduct research in compliance with Boston College policy and the federal regulations that have been established to ensure the protection of human subjects in research. Thank you for your cooperation and patience with the IRB process.

Approval Period: September 4, 2020 to September 3, 2021

Sincerely,

Guillermo Nunez
Executive Director of Research Administration
Enclosures

MZ

Confidential

(For ORP use only) BC IRB Protocol#: 21.006.01

**INITIAL IRB APPLICATION FORM**<br><br>For Initial IRB Review Only	**Boston College Institutional Review Board**<br>**Office for Research Protections**<br>140 Commonwealth Ave., Waul House<br>Chestnut Hill, MA 02467<br>(617) 552-4778 Fax: (617) 552-0498 E mail: irb@bc.edu

**I. Study Title:** (If funded must match the sponsored title)	mHealth Tools to Improve Service Delivery Quality of an Evidence-Based Family Home Visiting Intervention to Prevent Family Violence among High Risk Families in Sierra Leone	**Today's Date:** 09/02/20

II. Principal Investigator Information

A. Name of Principal Investigator	Dr. Alethea Desrosiers		B. Are You? (Please check)<br>◉ Faculty
C. Mailing Address:	140 Commonwealth Ave<br>Chestnut Hill, MA 02467		○ Staff<br>○ Undergraduate Student
D. Department:	Social Work/ Graduate School of Social Work		○ Graduate Student
E. E-mail address:	alethea.desrosiers@bc.edu		
F. Primary Phone Number:	(609)602-1466	G. Alternate Phone:	617-552-8398

III. Research Risk

Minimal risk means that the probability and magnitude of harm or discomfort anticipated in the research are not greater in and of themselves than those ordinarily encountered in daily life or during the performance of routine physical or psychological examinations or tests (45 CFR 46. 102). Research must present no more than minimal risk to human subjects in order to qualify for exempt or expedited review. Research must fit within one of the categories of review in order to qualify for that level of review.

Projects that propose greater than minimal risks to human subjects are considered full board protocols and they must be reviewed by the IRB at a convened meeting. The IRB meetings are held every third Wednesday of the month. Full board protocols must be submitted ten business days in advance of an IRB meeting in order to be reviewed at that month's meeting. ***NOTE:** Research involving prisoners is always considered a full board protocol and must be reviewed at an IRB meeting.*

A. Does the research propose greater than minimal risk to participants? ○ Yes ◉ No

B. Does the research include prisoners? ○ Yes ◉ No

Boston College IRB Approved October 18, 2019 Through August 20, 2020

FOR APPROVALS WITH NO CR REQUIRED

Boston College IRB Approved September 4, 2020- September 3, 2021

C. Level of Review

Please select a level of review and the appropriate category that corresponds to that level of IRB review:

☐ Exempt

☐ Expedited

☑ Full Board

IV. General Study Information

Is this project a *Clinical Trial*? ○ Yes ◉ No

A. Funding

1. ☐ **None** (Go on to Section B)

2. ☐ **University Funded**
3. ☐ **External**
4. ☑ **Federal** : List agency: NIMH

☑ Sponsor Award Number (if known)
R21MH124071
☑ BC Project Number (if known)
IRB#: 21.006.01

5. Is BC the primary awardee for the grant? ◉ Yes ○ No
6. Are there subcontracts? ◉ Yes ○ No If Yes Please list sub-contractors: Caritas Freetown and University of Makeni

B. Participant Recruitment Numbers: This number must be the maximum number you intend to recruit. You will not be allowed to recruit more than this number without first coming back to the IRB to seek approval.
Females: 90 Males: 90

C. Participant Ages (please check)
☑ 0-7 (parental consent and oral child assent)
☐ 8-11 (parental consent and child written consent)
☐ 12-17 (parental consent and written consent)
☑ 18-65
☐ 65+

D. Estimated Project Duration
Start Date: 08/17/20 End Date: 08/16/22

E. Why is this Project being conducted? (please check)
☑ Faculty/Staff Research
☐ Undergraduate Coursework
☐ Master's Thesis
☐ Doctoral Dissertation
☐ Other:

F. Will This Study Involve Long-Term Follow-Up with participants: ○ Yes ◉ No

G. Special Study Populations
☑ Minors (under 18 years) If including minors, also complete Research with Minors Form
☐ Pregnant Women/Fetuses or products of labor & delivery
☐ Prisoners
☐ Physically or mentally challenged
☐ Diminished capacity for consent
☐ Other:
☐ No Special Study Populations

Boston College IRB
Approved
October 18, 2019
Through August 20, 2020

FOR APPROVALS WITH NO CR REQUIRED:

H. Does this study involve any of the following?
☐ Deception or Punishment

Boston College
IRB Approved
September 4, 2020-
September 3, 2021

☐ Use of drugs
☐ Covert observation
☐ Induction of mental and/or physical stress
☐ Procedures which may risk physical/mental harm to the participant
☐ Materials/issues commonly regarded as socially unacceptable
☐ Information relating to sexual attitudes, sexual orientation or practices
☐ Information relating to the use of alcohol, drugs or other addictive products
☐ Procedures that might be regarded as an invasion of privacy
☐ Information pertaining to illegal conduct
☐ Genetic information that may be linked to a participant's health status, such as genetic markers for cancer, heart disease, etc.
☐ Information normally recorded in a patient's medical record, and the disclosure of which could reasonably lead to social stigmatization or discrimination.
☐ Information pertaining to an individual's psychological well being or mental health.
☐ Information that if released could reasonably damage an individual's financial standing, employability, or reputation within the community.
☑ None of the above Procedures

V. Research Summary:

Instructions: A research summary is a narrative that provides the IRB with important information about a study. Please provide a thoughtful response to each section using simple language and avoid technical jargon. If a specific question is not applicable to your study, please state not applicable or n/a .

A. Introduction and Background:

1. State the problem and hypothesis

Exposure to war, trauma, and other humanitarian crises can have persistent mental health effects across generations. Our prior study on the intergenerational impact of war in post-conflict Sierra Leone showed that past trauma and exposure to war is related to poor emotion regulation and harsh parenting practices, which adversely affect child development. In prior research among families facing adversity in Rwanda, we developed and evaluated the Family Strengthening Intervention for Early Childhood Development (FSI-ECD/*Sugira Muryango*), a home-visiting behavioral intervention delivered by lay workers. The FSI-ECD targets parental emotion regulation and parent-child interactions as major mechanisms to prevent the intergenerational transmission of emotional and behavioral difficulties related to past trauma. It has demonstrated effectiveness in improving parental emotion regulation, reducing family violence, and promoting healthy child development. The FSI-ECD is a promising approach for targeting underlying mechanisms linked to poor child outcomes. Vital for low-resource settings, it can be delivered feasibly by lay workers with strong supervision. Given the limited health infrastructure in many Low and Middle Income Countries (LMICs), behavioral interventions that can be delivered by well-trained and supervised lay workers are a more viable option for implementation and sustainment of evidence-based practices.

New leadership within the Government of Sierra Leone (GoSL) is focused on mental health initiatives to respond to the Nation s history of compound adversity and is pursuing mHealth strategies as means to address significant healthcare workforce limitations that plague delivery of evidence-based behavioral interventions to vulnerable families. Mobile technology has the potential to transform health care delivery and improve health outcomes in Sierra Leone and other LMICs by providing training, supervision, and fidelity supports, but it has not been widely applied to mental health and family-based prevention, particularly in Sub-Saharan Africa

Our proposed exploratory research is twofold: we will pilot a culturally adapted version of FSI-ECD while developing and piloting innovative and cost-effective mHealth tools to support Community Health Workers (CHWs) in Sierra Leone s underserved, rural Western region. Our dual focus will help build urgently needed capacity both for delivery of evidence-based mental health services to reduce family violence and harsh parenting practices, and for effective use of mHealth strategies to improve healthcare performance via quality improvement feedback, reporting, and case management.

Boston College IRB
Approved
October 18, 2019
Through August 20, 2020

Aim 1. Employ a user-centered design approach to develop mHealth tools to improve supervision and ongoing fidelity monitoring of CHWs. We will design, prototype, and test mHealth tools that incorporate user feedback from supervisors and CHWs at each stage of development. Our objectives are to (a) develop mHealth tools for supervision, fidelity monitoring, and training of CHWs in Sierra Leone; (b) provide supervisors with quick data displays on CHW performance to inform quality improvement cycles; and (c) apply user-centered design principles to collect preliminary feasibility and acceptability data to

FOR APPROVALS WITH NO CR REQUIRED:

Boston College
IRB Approved
September 4, 2020-
September 3, 2021

refine mHealth tools to fit the needs and preferences of CHWs and supervisors. We hypothesize that mHealth tools will be feasible and acceptable for CHWs and supervisors.

<u>Aim 2.</u> Conduct a Type II Implementation-Effectiveness randomized controlled pilot study to assess feasibility, acceptability, and preliminary effects of mHealth-supported delivery of FSI-ECD on caregiver mental health, emotion regulation, and familial violence in high risk families with children aged 6 36 months (n=40) in comparison to control families (n=40) who receive standard care. We hypothesize (a) the effects of the FSI-ECD will be comparable to results observed with vulnerable families in Rwanda; (b) digital tools will be feasible and acceptable to CHWs and supervisors; and (c) mHealth-enhanced supervision and fidelity monitoring will increase supervisor engagement and support CHW quality improvement cycles.

(a) the effects of the FSI-ECD will be comparable to results observed with vulnerable families in Rwanda; (b) digital tools will be feasible and acceptable to CHWs and supervisors; and (c) mHealth-enhanced supervision and fidelity monitoring will increase supervisor engagement and support CHW quality improvement cycles.

2. Provide the scientific or scholarly reason for this study and background on the topic

<u>Reducing family violence and harsh parenting practices is a global concern and urgently needed</u>. War affected populations face higher levels of intergenerational violence. The World Health Organization (WHO) estimates that 35% of women globally report experiencing intimate partner violence in their lifetime, and 75% of children in LMICs experience some form of violent or psychologically damaging discipline at home. Experiencing or witnessing family violence during early childhood increases risks for emotion regulation and other psychological problems, including post-traumatic stress disorder, externalizing and internalizing behavioral difficulties and school problems. In post-conflict Sierra Leone, our prior study on the intergenerational impact of war (R01HD073349) showed that exposure to war violence is related to poor parental mental health and harsh parenting practices. The 2017 Sierra Leone Multiple Indicator Cluster Survey found that 85% of children aged 3-4 and 67% of those aged 1-2 experience violent discipline. These abuses can be fatal; the WHO estimates 41,000 global annual homicide deaths in children under age 15.

<u>The Family Strengthening Intervention is an evidence-based intervention developed for families facing compounded adversity and has a key focus on promoting parental emotion regulation and preventing family violence</u>. The Family Strengthening Intervention for Early Childhood Development (FSI-ECD) comprises 5 core components delivered in 12 modules via in-home coaching by CHWs. We developed and tested the FSI-ECD in Sub-Saharan Africa both for families affected by HIV and for families living in extreme poverty. The FSI-ECD focuses on improving transdiagnostic factors (i.e., emotion regulation) to reduce family violence and improve child development outcomes. The FSI-ECD has demonstrated effectiveness in reducing violent child discipline and intimate partner violence and improving parental mental health and functioning. Given that poor parental emotion regulation is related to family violence and poor child development outcomes, the FSI-ECD s focus on adult emotion regulation and alternatives to violent discipline is a central mechanism of interest related to the intergenerational trauma and violence.

Intervention delivery strategies must innovate to address critical shortages in the mental health workforce in Low and Middle Income Country (LMIC) settings. Access to any form of health service in LMICs is often limited, particularly in rural areas. Sierra Leone s health infrastructure has been unable to respond effectively to the mental health needs of caregivers whose lives were affected by war and other forms of adversity. Sierra Leone s population of nearly 7 million is served by two psychiatrists, fewer than 20 psychiatric nurses, and a handful of doctoral-level mental health professionals. In 2017, the Government of Sierra Leone s (GoSL) Ministry of Health and Sanitation launched the Community Health Worker Policy, which defines a key role for CHWs in strengthening the delivery of preventative maternal and child health services and linking communities with the health system. The Policy identifies the innovative use of technology to improve performance monitoring, data collection, and data usage as a high priority.

Capitalizing on advances in technology can improve the efficiency and effectiveness of evidence-based mental health services in underserved, rural areas and strengthen the public health impact. Innovative mHealth supervision and fidelity monitoring tools can enhance quality of CHW service delivery and expand the reach of evidence-based mental health services to vulnerable families by generating a rapid feedback loop between supervisors and CHWs unconstrained by geographical distances. Successful implementation of mHealth tools in Sub-Saharan Africa has been limited by dependence on a reliable network connection and electricity. Despite increased proliferation of mobile phones and affordability of broadband technology in LMICs, less than 30% of the global rural population has mobile phone access. The GoSL has made a commitment to making broadband more widely available, but access in rural areas remains poor. Although 83% of adult Sierra Leoneans have access to a mobile phone, most lack internet access. Because CHWs access to power sources and internet connectivity is limited especially in rural areas, innovative use of battery-powered tablets with offline functions and access to cloud storage is logistically feasible. Investigating delivery of the FSI-ECD via the existing CHW platform using mHealth tools for quality

Boston College IRB Approved
October 18, 2019
Through August 20, 2020

FOR APPROVALS WITH NO CR REQUIRED:

Boston College IRB Approved
September 4, 2020-
September 3, 2021

improvement also aligns with the NIMH strategic priority to examine measures of quality that produce optimal results for scaling up training and supervision of mental health care providers and integrating core packages into existing delivery platforms. If successful, we will be able to extend the reach of mHealth tools by incorporating offline features that can provide a platform for delivering higher quality mental health services and to better collect and disseminate health information to rural and low-resource areas.

CHWs in Sierra Leone are community based workers that help members of their own communities access health and social services. CHWs are selected by their community and trained to provide basic health services and health education. Guidelines for selection include gender parity, the ability to communicate, an interest in health, the ability to perform CHW tasks, the ability to read and write in the local language, aged 18 years or above, and physical and mental fitness to provide services. All CHWs are required to complete a 10-day standardized training program. The training program involves the following modules: (a) working with communities and households and behavior change communication; (b) water, sanitation, and hygiene; (c) maternal and newborn health; (d) infant and young child high impact preventive and treatment interventions; (e) community integrated management of newborn and childhood illnesses; (f) adolescent sexual and reproductive health rights; (g) sexual and gender-based violence. In its Policy for Community Health Workers, The Ministry of Health and Sanitation has emphasized the importance of CHWs in providing basic services to improve maternal and child health outcomes and incorporates this into the training of CHWs.

CHWs in Sierra Leone are community based workers that help members of their own communities access health and social services. CHWs are selected by their community and trained to provide basic health services and health education. Guidelines for selection include gender parity, the ability to communicate, an interest in health, the ability to perform CHW tasks, the ability to read and write in the local language, aged 18 years or above, and physical and mental fitness to provide services. All CHWs are required to complete a 10-day standardized training program. The training program involves the following modules: (a) working with communities and households and behavior change communication; (b) water, sanitation, and hygiene; (c) maternal and newborn health; (d) infant and young child high impact preventive and treatment interventions; (e) community integrated management of newborn and childhood illnesses; (f) adolescent sexual and reproductive health rights; (g) sexual and gender-based violence. In its Policy for Community Health Workers, The Ministry of Health and Sanitation has emphasized the importance of CHWs in providing basic services to improve maternal and child health outcomes and incorporates this into the training of CHWs.

B. Specific Aims/Study Objectives:

1. List the purpose(s) of the study (what are you hoping to learn as a result of the study)

We will pilot a culturally adapted Sierra Leonean version of the FSI-ECD delivered by CHWs supported by mHealth tools that will enable them to collect diagnostic data, engage with supervisors, and access training resources. Supervisors will use mHealth tools to improve fidelity monitoring and gain more insight into CHW performance. Critically, we will apply user-centered design principles to develop the mHealth digital tools/apps. User-centered design focuses on the needs and preferences of users in each phase of the design process in order to enhance usability and increase likelihood of adoption.

Prior to the trial, we will adapt the FSI-ECD to the Sierra Leone culture, context, and setting. A Community Advisory Board will advise on local parenting and mental health terms and concepts drawing from previously collected qualitative data on parenting in Sierra Leone. The Community Advisory Board will comprise key stakeholders and community members, including social workers, adult representatives with young children, and leaders of community organizations. During cultural adaptation of the FSI-ECD, the Community Advisory Board will convene to review intervention materials for cultural appropriateness, acceptability, and significance and provide a list of suggested modifications, including culturally relevant concepts or themes. If there is lack of consensus between Community Advisory Board participants, the group will be invited to further discuss the differing viewpoints until consensus is attained. We have used the Community Advisory Board model in prior research in Sierra Leone and Rwanda to culturally adapt evidence-based interventions with success. Cultural adaptation will take place during the initial phases while mHealth tools are designed and developed. **The Program Manager will convene and run the Community Advisory Board meetings. The Program Manager will schedule and attend all of these meetings in person and facilitate group discussions to obtain feedback on FSI-ECD adaptation. A research assistant will accompany the Program Manager to take notes during meetings.**

Boston College IRB Approved
October 18, 2019
Through August 20, 2020

Our study will expand and deepen research partnerships with GoSL policy leaders and the University of Makeni (UNIMAK) to build capacity for behavioral health, implementation science, and data science research. Our highly collaborative study will help advance Sierra Leonean research capacity and encourage investment in mHealth-enabled, data-driven research and decision making. Engagement of UNIMAK faculty and students in study activities will demonstrate interest and commitment to developing and using research skills that are relevant for scaffolding GoSL investments in mental health research and data science as well as expansion of mHealth-supported behavioral health services.

FOR APPROVALS WITH NO CR REQUIRED

Boston College
IRB Approved
September 4, 2020-
September 3, 2021

Specific Aims:

Aim 1. Employ a user-centered design approach to develop mHealth tools to improve supervision and ongoing fidelity monitoring of CHWs. We will design, prototype, and test mHealth tools that incorporate user feedback from supervisors and CHWs at each stage of development. Our objectives are to (a) develop mHealth tools for supervision, fidelity monitoring, and training of CHWs in Sierra Leone; (b) provide supervisors with quick data displays on CHW performance to inform quality improvement cycles; and (c) apply user-centered design principles to collect preliminary feasibility and acceptability data to refine mHealth tools to fit the needs and preferences of CHWs and supervisors.

Aim 2. Conduct a Type II Implementation-Effectiveness randomized controlled pilot study to assess feasibility, acceptability, and preliminary effects of mHealth-supported delivery of FSI-ECD on caregiver mental health, emotion regulation, and familial violence in high risk families with children aged 6 36 months (n=40) in comparison to control families (n=40) who receive standard care.

Sub-Aim 2.1: We will conduct a cost-effectiveness analysis to assess the economic value of the mHealth-supported delivery of the FSI-ECD vs. standard care with standard supervision.

Aim 3. Leverage well-established relationships and GoSL partners to strengthen capacity for mHealth research and quality healthcare delivery in Sierra Leone. We will engage experts in mental health and data science to build in-country capacity in implementation science, mHealth, and behavioral science research. Unite States and Sierra Leonean investigators at the University of Makeni will partner with the GoSL Ministry of Health and Sanitation. Our study will provide a foundation for expanding knowledge, fostering collaboration, and developing research expertise.

C. Materials, Methods and Analysis (quantitative and qualitative)

1. Describe data collection methods (Procedures) (be specific):

We will recruit 40 families to receive the FSI-ECD and 40 control families from rural areas in the Western region of Freetown. Randomization will occur at the level of the family. To minimize contamination risk, we will use randomization rules developed in our prior work in Sierra Leone (e.g., GIS mapping to ensure nonadjacency and reduce potential contact of control and FSI-ECD families). GIS mapping will consider home addresses and other points of interest, such as schools, markets, and places of worship (e.g., mosque, church). GIS software will be used to conduct a buffer analysis to determine the likelihood of contamination and create coordinate maps. Different CHWs will provide the FSI-ECD and standard care to minimize contamination risks.

The Program Manager and study RAs will recruit families in coordination with the CHW Focal Person, who is the Ministry of Health and Sanitation Community Health Worker Program official responsible for coordinating the work of CHWs and supervisors within peripheral health units. Peripheral Health Units are key units within the Sierra Leone healthcare system. Through smaller community-based subunits, they deliver first line care, including prenatal care, routine deliveries, immediate postnatal and neonatal care, community outreach services, routine vaccination, and treatment of childhood illnesses and malnutrition. Peripheral Health Units maintain records of families in the community who have sought services and will be able to identify families with a child aged 6-36 months by reviewing their records. The CHW Focal Person will generate a randomized list of all families in the community with children aged 6-36 months. Research Assistants will then contact potential families and explain the study using the recruitment script.

Drs. Esliker and Desrosiers will review the protocol for screening and consent with the Program Manager prior to launching recruitment. As part of training in Good Clinical Practice, CHWs will be trained in protocols for protection of participant privacy and confidentiality. This will include guidance about not disclosing the identity of any families who are participating to anyone outside of the research study. Study RAs will also receive this training in protection of privacy and confidentiality. Home-based delivery of sessions is also intended to protect the privacy of families, and this approach has been successfully implemented in prior research in Rwanda.

Boston College IRB
Approved
October 18, 2019
Through August 20, 2020

FOR APPROVALS WITH NO CR REQUIRED:

Boston College
IRB Approved
September 4, 2020-
September 3, 2021

The Program Manager and one RA will conduct screening and consent with families who express interest in the study. The Program Manager will administer the Eligibility Screening Instrument, which includes the Difficulties in Emotion Regulation Scale (DERS), to determine eligibility. Data from the screening will not be used in the research study; therefore, we will not consent for participation in the screening. Families will be told about the screening process when RAs read the recruitment script. RAs will say the following to potential families: If you decide you want to be in our study, I will ask you to participate in the screening interview, which means that we will ask you some questions and you can answer however you want to. We will ask you questions about problems you might have, things you might feel, and things you do when you have problems or when you are feeling sad. This interview will allow us to determine if you can be a part of this study or not. Please remember that you do not have to answer any questions you do not want to. Families will then be reminded about this during participation in the screening.

The same procedure will be used to recruit, consent and enroll both intervention and control families. Eligible families within a specific community will have an equal probability of selection into the intervention and control groups.

FSI-ECD families will receive family home visiting sessions once per week for 12 weeks (12 sessions). Sessions typically last 90 minutes. The FSI-ECD manual is attached and provides more description about intervention content. Control families will receive standard maternal and child health services delivered by a CHW via standard supervision. Standard CHW care involves three home visiting, educational sessions delivered to families following childbirth, with weekly supervision via phone or face-to-face. Topics of home visiting sessions include: skilled post-natal care for mothers, early initiation of breastfeeding and exclusive breastfeeding practices, adequate nutrition, immunization services and timely use of these services, hand washing and hygiene practices (including waste disposal and food hygiene), building the capacity of family members to appropriately take care of newborns and children under age 5, and building the capacity of family members to recognize and act on postnatal danger signs for newborns, mothers, and children under 5. CHWs also conduct screenings for acute malnutrition and growth monitoring to identify early referrals, and they can provide family planning methods, deworming tablets and other vitamins for acute malnutrition, dehydration, and anti-malaria treatment. Each home-visiting session last approximately 60 minutes.

Although this exploratory design does not allow us to parse out the effects of the mHealth supervision tools in comparison to standard FSI-ECD supervision, it does allow us to examine preliminary efficacy of the FSI-ECD with mHealth enhanced supervision and fidelity monitoring.

Inclusion criteria are (a) Sierra Leonean household with cohabitating **caregivers** (e.g., father/mother, mother/grandmother, mother/intimate partner) and child, with both **caregivers** aged 18 or older; (b) having a child aged 6 36 months; (c) one **caregiver** scoring at least 62.5 on the DERS. Both **caregivers** must agree to attend FSI-ECD sessions. Exclusion criteria are active family crises, i.e., current suicidality or psychosis, serious medical condition, ongoing divorce process. **If one caregiver decides to withdraw from the study, the family can still continue participation in the study.**

RAs, trained and supervised by Drs. Desrosiers and Esliker, will screen families for eligibility using a tablet-based survey app to seek informed consent and administer screening measures. This app incorporates algorithms to identify families who meet inclusion criteria and has been used successfully in the ongoing Youth Forward study

FSI-ECD quantitative data collection will be at 3 time points: baseline, post-intervention, and 3-month follow-up by UNIMAK RAs. FSI-ECD quantitative outcomes will be caregiver emotion regulation, use of harsh parenting techniques, and family functioning. Control families will also complete quantitative assessments at the same time points as families who receive the intervention. All measures have been forward and backward translated from English to Sierra Leonean Krio utilizing the WHO process of translation and adaptation. Although the pilot study is not powered to detect treatment effects, we will investigate whether change in core outcomes occurs at post-intervention and 3-month follow-up.

FSI-ECD qualitative data collection will be post-intervention via key informant interviews with randomly selected **caregivers** (4 males/4 females) to assess feasibility, acceptability, and satisfaction of the FSI-ECD.

mHealth quantitative data collection will be at baseline and post-intervention via a dissemination and implementation measure of feasibility, acceptability, adoption, and appropriateness that we used previously in our scale-up study of the Youth Readiness Intervention, a culturally adapted cognitive-behavioral intervention in Sierra Leone. At the CHW level, we will track length of time to deliver FSI-ECD content, use of embedded fidelity monitoring and tracking features, and amount of CHW-supervisor contact via tablet, phone, and face-to-face. Fidelity data will include a CHW-completed electronic fidelity checklist designed to support self-monitoring and performance review with supervisors as well as a supervisor-completed electronic fidelity checklist to be completed while reviewing audiotaped FSI-ECD sessions and discussed during supervision.

Boston College IRB Approved
October 18, 2019
Through August 20, 2020

FOR APPROVALS WITH NO CR REQUIRED:

mHealth qualitative data collection will be at post-intervention via key informant interviews conducted by UNIMAK RAs. We will randomly select CHWs (2 male/2 female) and supervisors (1 male/1 female). To understand use of audio/video functions for

Boston College IRB Approved
September 4, 2020-
September 3, 2021

delivery, supervision, and quality improvement, we will probe supervisor review and feedback experiences and CHW experiences with recording and uploading/encrypting data, accessing training materials, and using data visualization tools.

2. Describe the specific materials or tools that will be used to collect the data (be specific)**:**

Quantitative assessments will be administered via tablets using the RedCap platform, which is HIPAA compliant. All quantitative measures have been used by the RPCA lab in previous longitudinal research and RCTs in Sierra Leone. From our previous use of these measures with war-affected and/or vulnerable populations in Sierra Leone, Cronbach s alphas have ranged from .72-.96, demonstrating strong reliability of these measures.

The following measures will be used to assess quantitative mental health outcomes for **caregivers** and **caregive**r-child interactions.

Difficulties in Emotion Regulation (DERS) [α=.96]

WHO Disability Assessment Schedule (WHODAS) [α=.91]

Post-traumatic Stress Disorder (PTSD) Civilian Checklist [α=.93]

Conflict Tactics Scale [α=.72-.86]

Home Observation for Measurement of the Environment (HOME) [α=.73]

Observation of Mother-Child Interaction (OMCI) [α=.83]

Hopkins Symptom Checklist [α=.92]

Implementation outcomes will be measured using the following:

Feasibility, Acceptability, Adoption and Appropriateness will be measured with quantitative scales developed by researchers at Johns Hopkins Bloomberg School of Health (Haroz et al., 2019). We have used these measures in our prior research in Sierra Leone, and the scales demonstrated strong psychometric properties (α= .79 -.90).

Qualitative data will be collected by the University of Makeni RAs, supervised by Dr. Esliker and the in-country Program Manager. Problem analysis and UI/UX testing sessions will be facilitated by Story and Structure, with the close supervision of Drs. Desrosiers and Marsch. For qualitative data collected related to FSI-ECD implementation, RAs will use a semi-structured qualitative interview guide. Family-level key informant interviews on FSI-ECD feasibility and acceptability will be held at participant homes. CHW- and supervisor-level key informant interviews will be held at identified community centers in the target communities. Qualitative data in will be audio-recorded, transcribed and translated

For cost-effectiveness data collection, we will use a costing tool developed to collect necessary data. We will use budget, expenditure, supervision, and fidelity data to collect implementation, health, and service costs using standard costing methodologies. Costs will include implementation activities (e.g., staff and CHW/supervisor trainings, session delivery, supervision) and directly related recurrent or capital items (e.g., tablets, tech support, broadband access, travel supplies). Costs of digital tools will be included as a capital item and amortized based on project duration. We will use standard implementation costing techniques where time invested by CHWs and other staff are multiplied by standard salaries plus benefits. Service delivery costs will rely on in-country data or standard costs provided by WHO-CHOICE published costs data. Outcomes will include a functional impairment measure (WHO-DAS) that can be converted to Quality Adjusted Life Year (QALYs). The WHO-DAS will be included in the quantitative assessment battery administered to caregiver participants.

Boston College IRB Approved
October 18, 2019
Through August 20, 2020

FOR APPROVALS WITH NO CR REQUIRED:

Boston College IRB Approved
September 4, 2020-
September 3, 2021

3. Describe timeline of the procedures and how long each procedure will last

Year 1: In quarter 1, we will recruit problem analysis and UI/IX testing participants and select UNIMAK scholars to serve as RAs. FSI-ECD adaptation will occur in quarters 1 and 2. Problem analysis focus group discussions will be held in quarter 2 (1 session). The design and development of mHealth tools, including UI/UX testing sessions (2 sessions) will occur in quarters 2 and 3. We will recruit CHWs and supervisors in quarter 2 and conduct FSI-ECD training (3 weeks) and technology literacy training (1 day). FSI-ECD family recruitment, enrollment, and baseline assessments will begin in quarter 3. FSI-ECD delivery will begin in quarter 4 (12 consecutive 90-minute weekly sessions per family). Research training activities for UNIMAK RAs will being in quarter 2 and continue throughout the duration of the study.

Year 2: FSI-ECD delivery will continue in quarters 1 and 2. Post-intervention assessments will occur in quarter 2, and 3-month follow-up will occur in quarter 3. Data analysis and dissemination will occur in quarters 3 and 4 (after 3-month follow-up data is collected).

Participation Duration: *Problem analysis and UI/UX participation* will be 90 minute sessions. *Family participation* is approximately 7 months (weekly FSI-ECD sessions for 3-months and assessments). *CHW/supervisor participation* is approximately 8 months (3-week FSI-ECD training, 1 day technology literacy training, FSI-ECD delivery, and assessments).

4. Describe how you will analyze your data (be specific)**:**

Quantitative data analysis will use mixed effects linear models to assess the effects of the FSI-ECD on caregiver mental health and emotion regulation, **caregiver**-child interactions and harsh parenting practices in comparison to standard care (treatment as usual control families). These models will account for clustering of families within CHWs delivering services and clustering of outcomes within families across time. If outcomes are skewed and violate the normality assumption for linear models, we will use generalized linear models with a Poisson distribution. All analyses will be conducted on an intent-to-treat basis. Paired t-tests and Wilcoxon signed rank tests will examine post-intervention change in quantitative dissemination and implementation outcomes of feasibility, acceptability, adoption and appropriate ratings among CHWs and supervisors, and families controlling for baseline scores.

The proposed pilot study is not powered to detect treatment effects of clinical significance. However if we assume a standard alpha level of 0.05, 80 families with two eligible respondents per family on average, and two time points, with assumptions of moderate intra-class (within-family) correlation (approximately 0.5), this pilot RCT has power of 0.80 to detect a standardized medium effect size of approximately 0.50 (Cohen, 1988). For outcomes for which there is only one observation per time point, and using the same assumptions as above, this pilot RCT has power of 0.80 to detect a standardized effect size of approximately 0.6.

Families lost to follow up will be included in all analyses, and depending on the analytic technique, missing time points may be addressed by the method itself (e.g., multilevel modeling), multiple imputation, and/or inverse probability weighting for attrition. All findings will be viewed in the context of lack of power to find effect sizes of clinical significance.

-

Qualitative data analysis of key informant interviews and audio-recorded session content will follow a 3-step analytical strategy derived from thematic content analysis and grounded theory. We will use open-coding to examine key interview themes (e.g., CHW and supervisor experiences with digital interface, barriers and facilitators to use, overall feasibility and acceptability of mHealth tools). We will develop a code book for qualitative analyses of CHWs and supervisors and cross-cutting themes triangulated across data source. We will iteratively develop a coding scheme organized by key themes. Drs. Desrosiers and Betancourt will instruct Dr. Esliker and UNIMAK RAs on use of co-developed code books to independently code 10-20% of transcripts at the appropriate level of analysis to examine reliability in application. Poor agreement (i.e., low kappa ratings as scored in MAXQDA) will be grounds for refining the code book. We will repeat reliability testing until coding is at >80% agreement for all data sources. Once coding operates at high reliability, we will code all datasets in MAXQDA. Mixed methods analysis will synthesize qualitative and quantitative data. This approach will also be used for qualitative data analysis of key informant interviews with **caregivers**.

Boston College IRB Approved
October 18, 2019
Through August 20, 2020

FOR APPROVALS WITH NO CR REQUIRED:

Boston College IRB Approved
September 4, 2020-
September 3, 2021

-

Cost-effectiveness analysis will estimate costs across FSI-ECD vs. standard care. We will use budget, expenditure, supervision, and fidelity data to collect implementation, health, and service costs using standard costing methodologies. Costs will include implementation activities (e.g., staff and CHW/supervisor trainings, session delivery, supervision) and directly related recurrent or capital items (e.g., tablets, tech support, broadband access, travel supplies). Costs of digital tools will be included as a capital item and amortized based on project duration. We will use standard implementation costing techniques where time invested by CHWs and other staff are multiplied by standard salaries plus benefits. Service delivery costs will rely on in-country data or standard costs provided by WHO-CHOICE published costs data. Outcomes will include a functional impairment measure (WHO-DAS) that can be converted to Quality Adjusted Life Year (QALYs). We will use standard incremental cost effectiveness analysis to compare mHealth supported delivery of the FSI-ECD to standard care and capture marginal variations in costs and effectiveness using incremental cost-effectiveness ratios (ICERs). Differences in intervention cost will be divided by differences in intervention effectiveness to calculate ICER ratios that can be used to understand the cost of the intervention per unit of outcome (cost per QALY). We can compare this to the standard willingness to pay threshold and to alternative programs to determine which programs are relatively more cost effective

D. Research Population & Recruitment Methods:

Describe:

1. Inclusion and Exclusion Criteria (what participant traits are needed to be included, what traits exclude participants?)

Focus Group-User Interface/User Experience (UI/UX) Participants:

- *Inclusion Criteria:* Meeting the aforementioned criteria; over aged 18 or older; ability to attend 1-2 sessions (90 minutes per session) at a central location either the CARITAS offices in Freetown or the University of Makeni (UNIMAK) campus.

- *Exclusion Criteria:* Individuals younger than age 18 and individuals who do not meet inclusion criteria.

Families: We will recruit 40 families to receive the FSI-ECD and 40 control families from rural areas in the Western Region.

- *Inclusion Criteria:* We will include families who are (a) a Sierra Leonean household with cohabitating caregivers (e.g., father/mother, mother/grandmother, mother/intimate partner), and child (aged 6-36 months) with both **caregiver**s aged 18 or older and; (b) one **caregive**r scoring at least 62.5 on the Difficulties in Emotion Regulation Scale (DERS). The DERS cut-off score has been used successfully as a risk assessment screening tool in our prior (R01HD073349) and ongoing studies in Sierra Leone (U19MH109989). Sierra Leonean caregivers scoring above this threshold on the DERS have reported significantly higher levels of severe physical punishment with their children as well as intimate partner violence. Both caregivers must agree to attend FSI-ECD sessions. If enrolled families have more than one child aged 6-36 months, we will include all eligible children as study participants. **Enrolled families can still continue participation in the study if one caregiver decides to withdraw from the study.**

-*Exclusion Criteria:* We will exclude families who do not meet all inclusion criteria and/or who are experiencing active family crises (e.g., current suicidality or psychosis, cognitive impairment, ongoing divorce process).

-

Boston College IRB
Approved
October 18, 2019
Through August 20, 2020

To assess for exclusion criteria, we have included single items in the screening instrument to ask participants whether they are involved in an ongoing divorce process. We have also included one item that assesses suicidality. If a participant answer the question on suicidality in the affirmative, this will activate the risk of harm protocol immediately. The risk of harm protocol is detailed in the response 6 in the Consent Documents Section of this letter and in the text of the application. As part of the RA training, we will train RAs about identifying the signs and symptoms of psychosis and cognitive impairment. The Screening Instrument Section on Psychological Analysis includes questions pertaining to the interviewer s (RA s) perception of current

FOR APPROVALS WITH NO CR REQUIRED:

Boston College
IRB Approved
September 4, 2020-
September 3, 2021

functional status of the participant. If the RA determines that the participant shows signs of psychosis, the Mini-SCID section on psychosis will be administered. We have included the Mini-SCID in this application as well. Additionally, these before mentioned procedures have been used successfully in the BC IRB approved Youth Forward Study and R01 Diffusion and Spillover studies in Sierra Leone.

-

Community Health Workers: We will recruit 8 CHWs from Peripheral Health Units in 2 target communities in coordination with the CHW Focal Person charged with overseeing all CHWs in said communities and the CHW District Coordinator of the Western Region. The CHW focal person overseeing the work of CHWs in the designated communities will provide a randomized list of names of potential CHWs. Study RAs will then contact CHWs based on the randomization list and read the recruitment script. This process will be continued until our target sample size is reached. If CHWs decide not to participate, this will not impact their ability to serve as CHWs in their community or their status in the community. RAs will explain this during the consent process. Because there are approximately 14,500 trained CHWs in Sierra Leone that are distributed in communities throughout the country, we anticipate that we will be able to reach our target sample size of eight CHWs and two supervisors. Traditionally, CHW is a volunteer position and there are no educational qualifications or knowledge and skill prerequisites that must be met in order to be hired as a CHW. CHWs who participate in AIM 1 Focus Group-User Interface/User Experience (UI/UX) sessions will be eligible to participate in AIM 2 pilot study.

- *Inclusion Criteria*: CHWs who are 18 years or older and who are assigned to the Peripheral Health Unit that provides health services in one of our target communities.

- *Exclusion Criteria*: Individuals under age 18 cannot be recruited to work as a CHW.

Supervisors: We will recruit 2 CHW supervisors from Peripheral Health Units in the target communities in coordination with the CHW Focal Person and the CHW District Coordinator of the Western Region. Supervisors who participate in AIM 1 Focus Group-User Interface/User Experience (UI/UX) sessions will be eligible to participate in AIM 2 pilot study.

- *Inclusion Criteria:* Supervisors are 18 years or older and oversee CHWs providing maternal and child health services
-Exclusion Criteria: Individuals under age 18.

2. What is the scientific or scholarly justification for the number, gender, age, or race of the population you intend to recruit?

Our target enrollment for families (caregivers with child), CHWs and supervisors is approximately 50 percent male/50 percent female for each participant group; however, this even representation is a certainty for caregivers only. All participants will be Sierra Leoneans because this is the racial/ethnic demographic composition of the population in Sierra Leone. We will not limit inclusion of any group by sex/gender, race, or ethnicity.

In our prior studies of the Family Strengthening Intervention, our male/female distributions have been roughly equal. Our intent is to recruit families that allow for an equal distribution of male and female children. We aim to recruit an equal number of male and female participants; however, the majority of the CHWs and supervisors in Sierra Leone are female. All data analyses will report qualitative and quantitative data disaggregated by sex. We will also examine sex as a biological variable in our mixed methods analyses.

Boston College IRB
Approved
October 18, 2019
Through August 20, 2020

We will enroll children aged 6-36 months as previous iterations of the FSI-ECD have successfully targeted this group. Excluding children over age 36 months ensures we are targeting families with children in the home versus children attending pre-primary programs, which would increase barriers to intervention delivery with the entire family in the home. Excluding children under age 6 months ensures we are targeting families with children who have developed some degree of mobility and an ability to interact with caregivers rather than passively receive care.

FOR APPROVALS WITH NO CR REQUIRED:

Boston College
IRB Approved
September 4, 2020-
September 3, 2021

The proposed pilot study of 80 families is not powered to detect treatment effects of clinical significance. However if we assume a standard alpha level of 0.05, 80 families with two eligible respondents per family on average, and two time points, with assumptions of moderate intra-class (within-family) correlation (approximately 0.5), this pilot study has power of 0.80 to detect a standardized medium effect size of approximately 0.50 (Cohen, 1988). Enrolling more than 80 families is not feasible given the project budget, timeline, and scope.

3. How did you choose the source of participants or data? (census records, BC students, Mass General Hospital records, etc.)

The source of our participants is based on our collaborating partners at UNIMAK and our collaboration with the GoSL Ministry of Health and Sanitation.

4. Recruitment procedure; including who will recruit participants and, if applicable, how any conflict of interest/coercion/undue influence will be mitigated

The Program Manager will supervise RA activity throughout the entire research process. The Program Manager and UNIMAK RAs will recruit families in coordination with the CHW Focal Person, who is the Ministry of Health and Sanitation Community Health Worker Program official responsible for coordinating the work of CHWs and supervisors within peripheral health units. Peripheral Health Units are key units within the Sierra Leone healthcare system. Through smaller community-based subunits, they deliver first line care, including prenatal care, routine deliveries, immediate postnatal and neonatal care, community outreach services, routine vaccination, and treatment of childhood illnesses and malnutrition. Peripheral Health Units maintain records of families in the community who have sought services and will be able to identify families with a child aged 6-36 months by reviewing their records. The Program Manager and one UNIMAK RA will conduct screening and consent with families and will obtain informed consent prior to screening. Drs. Esliker and Desrosiers will review the protocol for screening and consent with the Program Manager prior to launching recruitment. The DERS will be used as the eligibility screening tool. The DERS is a 36-item scale that measures difficulties managing emotions (e.g., impulse control). High scores on the DERS have been related to frequency of intimate partner violence (e.g., Gratz & Roemer, 2004). If one **caregiver** scores at least 62.5 on the DERS, the family will be eligible. The score of 62.5 is a clinical cut-off that corresponds with a greater likelihood of experiencing clinical disorders (e.g., Staples & Mohlman, 2014). We have used the DERS clinical cut-off successfully as a risk assessment screening tool in our prior (R01HD073349) and ongoing studies in Sierra Leone (U19MH109989). In our prior research, Sierra Leonean caregivers scoring above this threshold on the DERS reported significantly higher levels of severe physical punishment with their children and intimate partner violence. The DERS screener will be administered via tablet, which will automatically calculate a score to determine caregiver eligibility. If eligible, caregivers will be invited to participate in the study and will be asked to provide detailed contact information for future follow-up.

The Program Manager, in coordination with the CHW Focal Person(s) at the Peripheral Health Units in the target communities, will manage recruitment and enrollment of Focus Group Discussion participants for the problem analysis and UI/UX testing.

The Program Manager, with the assistance of the Focal Person affiliated with the Peripheral Health Unit in the target community, will recruit CHWs. Equal consideration will be given to all qualified and interested individuals. CHWs will be consented prior to the start of training. CHWs will receive financial support for the duration of their study participation (approximately 9 months) and build skills and capacity in mHealth technology, mental health diagnostics, and intervention delivery with fidelity. We believe that these pieces will a provide strong incentive for CHWs to participate for the duration of the study.

Boston College IRB Approved
October 18, 2019
Through August 20, 2020

FOR APPROVALS WITH NO CR REQUIRED:

CHWs will not function as research staff. They will not collect study data from families. CHWs will deliver the intervention. The problem analysis focus group discussions will be facilitated in by the Program Manager and a Directorate of Science, Technology and Innovation team member. Consultant Chokdee Rutirasiri will provide expertise on the user-centered design

Boston College IRB Approved
September 4, 2020-
September 3, 2021

process and will lead the focus group discussions remotely (e.g., via zoom or teleconference). Study research assistants will collect participant data from families, CHWs, and CHW supervisors. The Program Manager will oversee all data collection activities. This includes the problem analysis focus group discussions, UI/UX testing sessions, quantitative assessments for families, key informant interviews with families, key informant interviews with CHWs, and supervisors who delivered the intervention, and implementation science quantitative assessments with CHWs and supervisors.

5. Tools that will be used to recruit (payment, advertisements and flyers attach copies to this application)

The Program Manager and UNIMAK RAs will work with the Ministry of Health and Sanitation and the CHW focal person to identify potential participants. A recruitment script will be used to explain the study to eligible participants and determine if they are interested in participating. The RAs will attempt to contact potential participants by phone up to three times before traveling to the address provided to contact them in person. The team will attempt three times to contact the potential participants in-person before considering that potential participant to be ineligible or uninterested. For those participants who consent to the study, their contact information will be securely stored, and they will be contacted by UNIMAK RAs.

6. Research Incentives and Payments: Please specify what form of payment you will be using and how it will be documented. (Note: participant payment beyond $600 must be reported to the IRS, and this requirement must be added to the consent form)

All caregiver participants will be compensated for their participation to include a small home gift (e.g., soap) equal to 30,000 Leones (equivalent to two days wages in Sierra Leone) for completion of each quantitative assessment. As advised by Sierra Leonean researchers and partners at the Ministry of Health and Sanitation, the household gift will be given in lieu of cash in order to maintain a non-coercive, culturally sensitive enrollment process while still providing strong incentives for participation. These incentives will be provided at each time point to encourage participation over time. A participant log will be maintained which will document the participant's unique ID, the time points in which they are participating in either an assessment or interview, and a box to mark when they are provided compensation. Caregivers who also participate in key informant interviews will be compensated with a home gift equal to 18,000 Leones.

CHWs who deliver the FSI-ECD will be paid 30,000 Leones per day (2-3 USD) that they work three hours on the project. This payment is in addition to the incentives CHWs receive from the Government of Sierra Leone. CHWs who deliver the FSI-ECD will receive 18,000 Leones for completion of questionnaires, and those who also participate in key informant interviews will receive 18,000 Leones for their participation in each interview. CHWs who participate in the user-centered design focus group discussions will also receive 18,000 Leones for their participation in each focus group discussion. All funds for CHWs will be provided by Boston College funds through the subaward to Caritas-Freetown, who will handle the processing of these local expenses.

E. Informed Consent Procedure:

1. Who will perform the informed consent procedure, and how will that person be trained? (*Note:* undergraduates should specify their qualifications and describe how the faculty research supervisor will closely monitor.)

Dr. Esliker will lead RA trainings with the Program Manager. UNIMAK RAs, in close coordination with the Program Manager, will obtain participant consent. Caritas-Freetown will house our in-country Program Manager. Caritas has worked with us during our previous randomized controlled trials in Sierra Leone and oversaw the consent process during these trials.

2. How will the prospective participant's competence or understanding of the procedures be assessed; will participants be asked questions about the procedures, or encouraged to ask questions?

Boston College IRB Approved
October 18, 2019
Through August 20, 2020

The consent letters will be read out loud to participants to ensure illiteracy does not interfere with the consenting process. Participants will be assured that their decisions about participation (yes or no) will in no way affect their relationship with other service facilitators currently in the community. The consent forms will describe all aspects of the study using literacy-appropriate language including procedures for handling data and explain that confidentiality will be maintained unless concerns about the

FOR APPROVALS WITH NO CR REQUIRED

Boston College
IRB Approved
September 4, 2020-
September 3, 2021

participant warrant reporting, such as suicidality, homicidality or abuse. The consent will describe the purpose of the study, the participant's involvement, where the study will be conducted, how much time participation is expected to entail, and the information they will be asked to provide. Participants will also be told that participation is voluntary and they are free to withdraw participation at any time. Sufficient time will be allowed for questions about the consent forms or about the study in general.

Participant comprehension of the study, including study details, risks, and benefits, and voluntary participation will be assessed and documented. Drs. Desrosiers, Betancourt, and Esliker will ensure that all research staff are fully trained to conduct the consent process and assess participant comprehension. RAs will evaluate comprehension by asking participants to repeat back their understanding of the study, what their participation entails, and the risks and benefits within. RAs will also use open-ended questions to make sure that potential participants understand all aspects of the study.

3. Please describe the process by which informed consent will be obtained. ***Note: research involving minors requires consent from a parent/legal guardian and assent from the child.***

RAs will read aloud in Krio, the local language. Reading informed consent forms aloud in Krio is necessary due to the low literacy level in the catchment area for the study. The form will include a description and purpose of the study, procedures to be followed, and what is expected of the participant (e.g., response to questionnaires, protection of confidentiality, the voluntary nature of participation in the intervention and study). In addition, study personnel will discuss any risks related to participation in the study as well as the benefits of participation with the prospective participant.

Sufficient time will be allowed for questions about the consent forms or about the study in general. For participants who request time to consider participation or to discuss the study with family/friends before deciding on participation, they will be given a week to do so. Our research team will follow up with those participants at the end of the one-week period.

Participants will provide verbal consent, which will be logged by the research staff obtaining consent. A research team member will witness the consent and sign the form. Each participant will be offered a copy for his/her reference; the form will include contact information for our Program Manager. Research staff will encourage participants to contact the Program Manager should they have any questions or concerns about the study. Prospective participants also will receive contact instructions for the PI (Dr. Desrosiers), the Sierra Leone Ethics and Scientific Review Committee chair, and the Boston College School of Social Work IRB. If an invited participant declines to participate, we will offer to answer any questions about the study. Only those who consent to participate will be enrolled.

Consent forms will provide participants information regarding conditional assurance of confidentiality for their participation. The section on consent forms indicates the steps the research team will take if they have reason to believe the participant or others are at serious risk for self-harm or harm by others (e.g., current suicidal ideation, reports of physical or sexual abuse). If confidentiality must be breached for a participant, research staff will inform the Project Manager and the PI. Subjects refusing to consent/assent will be thanked for their time and withdrawn from participation. Prior to each quantitative assessment, the core components of the consent form will be reviewed with participants again.

E. Confidentiality:

Boston College IRB Approved
October 18, 2019
Through August 20, 2020

Describe the Provisions for participant and data confidentiality:

1. Where will the data be stored, and who will have access to the data and the area? (**Please refer to the *References Tab* for the latest BC Research Data Policy.)**

FOR APPROVALS WITH NO CR REQUIRED:

Boston College IRB Approved
September 4, 2020-
September 3, 2021

Data will be collected on electronic tablets due to the risk associated with handling paper-based surveys. Tablet collected survey data will be de-identified, stored on encrypted devices, and downloaded routinely on encrypted and password-protected laptops. Trained, supervised research assistants will collect the data and maintain oversight of the password protected tablets. To address concerns of breach of confidentiality, data will be entered into RedCap, an electronic data management software with both online and offline features. Study staff will only able to access RedCap using a secure password on a password-protected tablet. All study documents and data will be maintained in password-protected computer files. All tablets will be kept in secure cabinets with a locking mechanism. Qualitative data collected will be stored electronically, with translations and transcriptions typed to ensure no loss of data. Participant identifiers will be removed from translations and transcriptions. Confidentiality of study documents will be maintained by assigning unique study IDs and using these rather than participant names on all study related materials. Paper copies of documents will be maintained in locked file cabinets in respective countries, which only study staff will have access to. Participant consent forms and ID logs will be kept in a separate location in two separate locked cabinets. Digital audio recorders will be used to obtain audio recordings. **Audio file recordings will be saved to a password protected computer hard drive as soon as possible after data collection, and recordings will be deleted immediately after they are saved.**

2. How will the data be stored, and in what format (hard or electronic copy, identifiable or de-identified)

All study documents and data will be maintained in password-protected computer files or on password protected tablets. Audiotapes and paper copies of documents will be maintained in locked file cabinets and transferred in locked document carrier bags. Participant consent forms and ID logs will be kept in two locked cabinets in a separate location. Transcriptions and audiotapes of interviews will be identified by ID numbers only and not connected with the participants names. Source documents will be shredded at the time of study completion in accordance with Boston College Standard Operating Procedures for Researchers Using Human Participants in Research. All hard copies of data will be stored in locked cabinets to which only the local Co-Investigator and RAs have access. After completion of an assessment with a study participant, data with Study ID numbers will be placed in a subject binder in a separate locked file cabinet while waiting for data entry. Once data is entered into computer files and password protected, only the local Co-Investigator, Program Manager, and data entry assistants (RAs) will have access to these files.

3. Will you collect the names, email addresses, phone numbers, student ID numbers, and/or any other data elements that individually identify the subjects of the research? Will the participant's identity be coded? Will the codes to identify participants be stored with the data?

Each district, chiefdom, and participant will have a Study ID#. The Study ID# is a 6-10 digit numerical code depending on the type of participant. It is the link between the participant s name and the data they provide for the study. A study ID log will be kept separate from study materials in locked cabinets and/or password-protected computers in a secure building at Caritas in Sierra Leone. All documents (e.g. questionnaires) will be labeled with the participant s Study ID#, not their name. This is to protect the privacy of each participant. All assessment materials will be labeled with the participant s Study ID#, not their name. This is to protect the privacy of each participant. Study IDs will be pre-populated. The Program Manager and team will work together to prepopulate all the assessment materials prior to data collection.

4. Will you collect any identifiable information from any organizations or individuals other than the subjects (or potential subjects) **themselves?** (e.g., a hospital or school) ○ Yes* ◉ No

G. Statement of potential research risks to subjects (e.g. breach of confidentiality, treatment complications)

1. Indicate the type of risk that may result from participation. Consider psychological or emotional risks, social stigma, change in status or employment, physical risks or harms, information risks-breach of confidentiality and any effect loss of confidentiality may have on status, employment, or insurability. If the protocol involves treatment, what are the risks compared to other treatments in terms of "standard of care"? *Please consider any populations who may be particularly vulnerable and may be triggered by this research topic and the risks they may face. Please check section IV.H. to ensure that you have considered how sensitive topics might lead to risks for some populations.*

Boston College IRB Approved
October 18, 2019
Through August 20, 2020

Planned procedures to protect against data loss or breaches in confidentiality: Participant diagnostic and assessment data will be collected via tablets by trained UNIMAK RAs. All CHWs will receive training in Good Clinical Practice including proper use of the tablets and transmission of participant data from the tablet to a secure server. During the intervention, CHWs will use the tablets to audiotape sessions. Prior to deployment to the field, the tablets will be encrypted by the Information Technology (IT) department at Boston College. All tablets will be password protected using a password known to the CHW, supervisor, and Program Manager. All data on the tablet will remain on the tablet until it is connected to WIFI and uploaded to a secure server.

FOR APPROVALS WITH NO CR REQUIRED:

Boston College
IRB Approved
September 4, 2020-
September 3, 2021

Daily quality assurance and data monitoring checks will determine successful upload of the data, which will be backed up to Box, a secure, HIPAA-compliant, cloud-based storage platform, before it is remotely wiped from the tablet. In the event a tablet is lost or stolen, Boston College IT can remotely wipe it. We will instruct CHWs that in the event of loss or theft, they must immediately inform their supervisor, who will then immediately notify the Program Manager and PI. The Program Manager will contact Boston College IT with the tablet serial number to initiate a remote wipe of the tablet, The PI will make a report to the Boston College IRB and Sierra Leone Ethics and Research Committee.

-

Planned procedures to protect against social harm resulting from breaches in confidentiality: In the event that a participant reports social harm that results from participation in the study, research study social workers will make every effort to provide appropriate short-term counseling to the subject, and/or referral to appropriate resources for the safety of the participant, as needed. This includes services for housing and food insecurity, domestic violence, mental health, and social services. Information regarding potential social harm will be recorded systematically in study logs. All instances of immediate risk of harm (e.g., situations involving risk of harm to self or others) will activate the study safety plan. This will involve intervention from study social workers and research coordinators, and participation in the study will stop. The PI will also be notified in all such instances. Serious risk of harm cases will be well documented and reported to the IRB, and appropriate referrals will be enacted.

-

Planned procedures to protect against distress related to participation in the FSI-ECD: The FSI-ECD targets vulnerable families with caregivers experiencing impairments in emotion regulation. Based on prior implementation of an adapted version of the FSI-ECD with families in Rwanda experiencing extreme poverty, strong procedures are in place in anticipation of issues that may arise during intervention delivery. A core focus of the FSI-ECD is to improve caregiver-child interaction and the overall functioning of the family. Issues that may cause distress for caregivers include violence against children and intimate partner violence, substance abuse, and caregiver mental health. CHWs will receive extensive training on how to identify these issues when delivering the intervention and how to facilitate conversations with caregivers and support their access of formal supports in the community. Naturalistic videos embedded in the app will provide CHWs with anytime, anywhere access to training and support to help them prepare for these conversations. The CHW supervisor and CARITAS social workers will be an additional source of support and can work with the CHW and family to further address any issues in the home. For cases needing extra support, a trusted community leader may be consulted, and for extreme cases, the risk of harm safety plan may be enacted.

-

Planned procedures to protect against distress related to psychosocial assessments: Some of the content and questions included in the psychosocial assessments may be perceived as sensitive and could cause some level of discomfort for participants. In order to reduce this risk for discomfort, informed consent forms will clearly indicate that individuals unwilling to discuss personal matters may choose not to enroll and may terminate participation at any time. In addition, informed consent forms will emphasize that participants who elect to enroll may choose to abstain from answering any question if it makes them feel uncomfortable.

Adverse events due to participation in psychosocial assessments are anticipated to be rare; however, we have a plan in place to respond should such adverse events occur. We will train the study team in how to identify signs of distress in caregivers. If a participant becomes distressed during the psychosocial assessment, social workers will be available to provide additional individual support and assessment of need for additional mental health services. Should any participant endorse survey items indicating thoughts of suicide, the interviewer will discontinue the survey administration to assess for risk of harm.

In any situation involving study participants in immediate risk of harm (i.e., current suicidal ideation or plan, report of current physical or sexual abuse) the study team member will activate the safety plan, regardless of whether or not the danger is due to study participation. This includes family participants, CHW participants, and CHW supervisor participants.

Boston College IRB Approved
October 18, 2019
Through August 20, 2020

FOR APPROVALS WITH NO CR REQUIRED:

Boston College IRB Approved
September 4, 2020-
September 3, 2021

Safety plan to identify and address risk of harm cases: Situations involving study participants in immediate risk of harm will be triaged by the study team member present at the time of interview. The Program Manager and UNIMAK RAs will discuss all risk of harm cases and report them to the PI. The PI will notify the Boston College IRB of risk of harm cases within 24 hours. Risk of harm cases will be referred to appropriate local mental health counselors and emergency facilities as needed within 24 hours. Study social workers or the Program Manager will be available to provide additional individual support and referrals to local social workers for additional mental health services. A standardized form for reporting any activation of the safety plan will be designed and all study team members will be trained in its application. For situations involving cases of suspected abuse (e.g., child abuse), the Program Manager will also contact the local Family Support Unit to report the case. All RAs will complete CITI training on research ethics and compliance and will receive extensive training on the proper protocol for recognizing and responding to risk of harm situations. The following situations will activate the action plan.

-

Flagged questions: Some survey items on the psychosocial assessment are designed to assess participants risk behaviors and welfare, including critical issues such as suicidal ideation, physical abuse, and sexual abuse. Specific questions related to these problems are flagged ; a positive response to flagged questions will mandate activation of the safety plan. The use of flagged questions is intended to minimize variability in research staff response and to ensure that all research personnel respond to certain indicators of risk of harm.

-

Concerning scores: For example, a concerning score for intimate partner violence reported by a   participant will activate the safety plan.

Potential action plan responses are:

-

If suicidal ideation is reported by a participant: Research staff will conduct an immediate follow-up assessment with the participant. Staff will use scripts, which have been developed for these follow-up sessions, and include specific text for discussing mental health problems and access to services (e.g., Based on your responses to some of these questions, I have concerns about your safety and would like to have someone on our team touch base with you). After having this session with any participants in distress, staff will contact the Program Manager for an initial assessment of suicidal risk and will inform the PI. Trained study social workers from Caritas will conduct this assessment to determine current suicidal ideation or psychosis. Appropriate referrals will be made to the most suitable mental health services (e.g., social work, psychology, or psychiatry) depending on level of clinical need. Referral services will be contacted if suicidal ideation is ongoing and the participant cannot be kept safe with social work and family level interventions.

-

If ongoing abuse is reported by a participant: Research staff will inform the Program Manager or PI, and if appropriate, a local authority through the current legal, health, and social services channels. If reported abuse relates to a child in the study, a report will be submitted to the Ministry of Social Welfare District Council or Family Support Unit of the Police, as required by national law. In abuse cases involving participants, the safety plan recommends informing the participants primary care or mental health counselors.

-

If a participant scores at very high levels of intimate partner violence or indicates ongoing intimate partner violence: Research study social workers will perform an initial evaluation, confer with the Program Manager and PI, and refer for appropriate care through the primary care counselor or mental health provider.

Boston College IRB Approved
October 18, 2019
Through August 20, 2020

-

FOR APPROVALS WITH NO CR REQUIRED:

Boston College IRB Approved
September 4, 2020-
September 3, 2021

Referrals for participants in need of stage two treatment: At the time of psychosocial assessment interviews, participants will be asked questions to assess for anxiety and stress. Participants will be asked, Do you feel you need any additional care or services due to your experiences today? Are there any questions or concerns that you would like to discuss with a mental health provider or other health professional? Any risk of harm concerns will be discussed at this time. If needed, the counselors will provide referrals to appropriate services. All questions will be referred to Dr. Desrosiers and the Program Manager. All Safety Plan Activation Forms will be saved as an encrypted file in a password protected folder on a password protected computer managed by the Program Manager. This folder will also be saved on Box, a secure HIPAA-compliant, cloud-based platform. The study team will minimize the use of paper logs, instead utilizing computers and tablets to track participant-related study logs.

We will refer study participants who show persistent impairment and distress due to post-traumatic stress disorder, anxiety, depression, or mental health concerns to mental health nurses with the Peripheral Health Unit in the community or to other community programs. If necessary and with the consent of participants, a trusted community or family member may also be enlisted as a source of interpersonal support. The Program Manager and research staff will discuss all cases requiring treatment and report them to the PI. We will design a standardized form for reporting referrals; all study team members will be trained in its application.

This protection against risk of harm plan has been successfully implemented and used in our prior work in Sierra Leone (U19MH109989/Youth Forward). RAs in the Youth Forward study have conducted resource and referral mapping to ensure that the appropriate referrals can be made given the geographic location of participants. Available health-related services as well as non-government organizations and community-based organizations have been documented at three levels: the community level, the chiefdom level, and the district level. This data provides a map of available referral services from which the flow of referrals can be documented depending on the situation. This ensures referrals are made to clinics and mental health professionals that are accessible to each participant and appropriate to their specific situation.

Adverse events (AEs) will be graded according to their attribution (either related or unrelated to the study) and their severity. Serious Adverse Events (SAE) are any adverse event that meets one of these criteria:

- The event results in death
- The event is life-threatening
- The event results in an inpatient hospitalization or prolongation of existing hospitalization
- The event results in permanent or severe disability or permanent damage
- A pregnancy results in a congenital anomaly or birth defect
- Based on appropriate medical judgment, the event may jeopardize the subject s health and may require medical or surgical intervention to prevent one of the other outcomes listed above

AEs and SAEs will be graded as:

- Mild (no limitation of usual activities),
- Moderate (some limitation), or
- Severe (inability to carry out usual activities).

Boston College IRB Approved
October 18, 2019
Through August 20, 2020

FOR APPROVALS WITH NO CR REQUIRED:

Boston College IRB Approved
September 4, 2020-
September 3, 2021

AEs and SAEs will be attributed according to the relationship to the study product and/or procedures as:

Not related,
Unlikely,
Possible,
Probable, or

Adverse Event Reporting:

Information on AEs and SAEs will be reported to the PI (Desrosiers) and in-country Program Manager on a weekly basis. An adverse event report will be generated for each event that will be included in the weekly report. This weekly AE/SAE report will only be submitted when there are relevant AEs and SAEs to report. The field-based managers and study social workers will meet with the research assistant who triggered the AE or SAE to follow up and document the presenting concerns. The response to ensure safety and access to relevant services will also be documented, including any referrals to social work or mental health services that were made and the subsequent outcome. These monthly reports and weekly AE and SAE reports will be stored in study records.

2. Consider the likelihood and magnitude of the risks or discomforts occurring? Are they unlikely, or likely to occur and what effect would the discomforts or risks have on the individual should they occur?

Participants may experience psychological or privacy risks; however, the likelihood and magnitude of risks or discomforts occurring are anticipated to be low.

3. How will you minimize the risks? Some examples include informed consent, adequate staff training and experience, debriefing, and monitoring adverse effects on participants

Some of the content and questions included in the psychosocial assessments may be perceived as sensitive and could cause some level of discomfort for participants. We will minimize risks by obtaining voluntary consent from all participants using informed culturally responsive consent procedures consistent with IRB guidelines. In order to reduce this risk for discomfort, informed consent forms will clearly indicate that individuals unwilling to discuss personal matters may choose not to enroll and may terminate participation at any time. In addition, informed consent forms will emphasize that participants who elect to enroll may choose to abstain from answering any question if it makes them feel uncomfortable.

Adverse events due to participation in psychosocial assessments and key informant interviews are anticipated to be rare; however, we have a plan in place to respond should such adverse events occur. We will train research assistants in how to identify signs of distress in participants. If a participant becomes distressed during the psychosocial assessment, social workers will be available to provide additional individual support and assessment of need for additional mental health services. Should any participant endorse survey items indicating thoughts of suicide, the interviewer will discontinue the survey administration to assess for risk of harm. In any situation involving study participants in immediate risk of harm (i.e., current suicidal ideation or plan; report of current physical or sexual abuse) the research team member will activate the safety plan (see above response for more detailed information on the safety plan).

Boston College IRB
Approved
October 18, 2019
Through August 20, 2020

If a participant decides to withdraw from the study, the research team will document this, and the participant will be withdrawn. The research team will refer the participant to the appropriate mental health services if needed. Our research team in Sierra Leone maintains a strong referral network to include universities with access to clinical staff in medical school and university-affiliated hospitals, and will refer to these resources when participants need mental health care. If a participant withdraws, all

FOR APPROVALS WITH NO CR REQUIRED:

Boston College
IRB Approved
September 4, 2020-
September 3, 2021

data collected up to that point will remain and be used in the study, unless otherwise indicated by the participant (which will be documented in the subject log).

All members of the research team will be trained in research ethics, including methods to protect participant confidentiality and the importance of that protection. Special emphasis will be placed on providing training in ethical research conduct for the local Sierra Leonean CWHs, supervisors and research assistants. In particular, for team members who reside in the study catchment area, training will focus on the maintenance of confidentiality when performing research.

As part of training in Good Clinical Practice, CHWs will be trained in protocols for protection of participant privacy and confidentiality. This will include guidance about not disclosing the identity of any families who are participating to anyone outside of the research study. Study RAs will also receive detailed training in protection of privacy and confidentiality. Additionally, it is common practice for CHWs to go to the homes of families and deliver basic services at a time convenient for the family (i.e., standard services), and therefore would not be considered unusual by neighboring families. CHWs will also be trained on how to identify times that can most ensure privacy in the home and minimize potential contact with neighboring families.

We will enroll children aged 6-36 months in the study. Caregivers must provide informed assent for children to participate in assessments and the FSI-ECD. During data collection, if a child presents with an issue necessitating additional care (e.g. malnutrition), the team member will notify the Program Manager and CHW supervisor to make a referral for additional services. Study RAs will be trained in use of an anthropometrics assessment tool to measure upper arm circumference as an indicator of malnutrition. This approach has been used successfully in prior research on the FSI-ECD in Rwanda to indicate malnutrition (NCT02510313). If there is a case of suspected abuse or neglect, the RA will enact the safety plan immediately to ensure that proper services are in place for the child and family.

H. Statement of potential research benefits to subjects (Please note that compensation/ payments are considered a recritment tool and should not be listed as a benefit)

1. Indicate the type of benefit that may result from participation. Consider psychological or emotional benefits, learning benefits, physical benefits and discuss if participant will benefit directly or if the benefit is largely to gather generalizable knowledge or provide scientific or social information on a topic that may benefit society. DO NOT OVERSTATE the benefit.

In prior implementation of the FSI-ECD in Rwanda, participants reported that their study participation helped them to improve their ability to care for their children and work in partnership with their intimate partner and resolve conflicts in healthy ways. Potential benefits for families enrolled in the FSI-ECD include enhanced contact with trained CHWs. A tablet interface that integrates validated diagnostic tools (e.g., the DERS,) will enable CHWs to identify and monitor family violence and mental health issues and potentially provide the Ministry of Health and Sanitation with surveillance data to inform policy and service delivery decisions**.** CHWs will be able to view results of mHealth data obtained on these diagnostics on dashboards via user-friendly visualization tools (e.g., charts, summaries) to enable immediate identification of families with concerning scores on indicators of violence or mental health problems as well as easier tracking of changes over time. Given the focus of the FSI-ECD, caregivers enrolled in the study may experience improved emotion regulation, with positive impacts on one s ability to utilize healthy coping strategies and respond to stressful situations. A structured safety plan, which includes a clinical supervisor, Caritas social workers, and respected community leaders, will be in place to assist families in navigating external formal support to address issues requiring a higher level of care. When these problems are addressed promptly and appropriately, the caregivers and their children will benefit.

Boston College IRB
Approved
October 18, 2019
Through August 20, 2020

Potential benefits for CHWs include the ability to expand their capabilities to utilize mHealth tools. More specifically, CHWs will improve their ability to perform diagnostics and deliver targeted services to families in need. They may also experience an enhanced supervision experience and/or greater support from supervisors through the mHealth app functions. As part of our fidelity monitoring and implementation approach, CHWs will participate in structured supervision that targets CHW capacity and skill building related to intervention delivery. As such, CHWs will receive one-on-one feedback regarding their strengths and

FOR APPROVALS WITH NO CR REQUIRED:

Boston College
IRB Approved
September 4, 2020-
September 3, 2021

weaknesses, with targeted recommendations and direction for improvement. Enhanced supervision for CHWs will not only improve their abilities as CHWs it will improve how the FSI-ECD is delivered in the home, which will strengthen FSI-ECD impact for participating families. Finally, CHWs in our study will be better positioned to meet the national priorities outlined in the National Community Health Worker Policy.

All participants will be engaged in research that may benefit families facing adversity in Sierra Leone and more broadly in Sub-Saharan Africa and other LMICs. Participants may gain emotional benefit or satisfaction from contributing to the body of knowledge regarding health and populations facing compounded adversity. Our data will be used to identify issues of priority to policy makers and program developers that can be the targets of improved policies and services in this and other settings. Beyond Sierra Leone, the general patterns and lessons learned from this research have the potential to improve services and service delivery for vulnerable families and to expand the reach of services to other regions. The data will not only contribute to implementation science research, but also will help our local partners, UN agencies, and other policy audiences to understand the nature of this population s needs and inform decision-making regarding scaling up of the m-health supported FSI-ECD.

2. Consider the likelihood of the benefits. Will all or some participants benefit?

We expect that all participants will be equally likely to benefit.

I. Investigator experience. Please attach a current copy of your C.V. unless a current copy is on file. Note: the IRB only needs the C.V. from the PI, not the faculty advisor.

VI. Informed Consent

If this project qualifies for Exempt review, category 4 (previously collected data), or Informed Consent is otherwise not applicable click here ☐ and skip to section IX.

Are you requesting an alteration or waiver? ◉ Yes* ○ No ○ Both - I have a consent form, but will be asking for a waiver for some cases

B. Consent Waiver/Alteration: In rare circumstances, the IRB may consider altering the informed consent requirements. In order for the IRB to approval a waiver of the informed consent process or a partial waiver of any of the required elements of consent, require that: (*Federal Regulations: 45 CFR 46.116(d)(1-4)*

1. The research involves no more than "minimal risk;"
2. The waiver will not adversely affect the rights and welfare of the research participants;
3. The research could not practicably be carried out without the waiver; and
4. Whenever appropriate, the research participants will be provided with additional pertinent information after participation.

Are you asking for a partial waiver of consent: ◉ Yes ○ No

Please indicate which elements you are asking to waive::

☐ A statement that the study involves research
☐ The purpose of the research in lay terms (in language understandable to the participant)
☐ A statement that they are being asked to participate in research, and how they were selected to participate
☐ The expected duration of the participant's participation "You will be asked to complete a survey every month for 1 year"
☐ The total time commitment of participation in the procedures "the survey will take 20 minutes to complete"
☐ A brief but complete description of all procedures to be followed (if research includes treatment describe which procedures are experimental and alternatives to those procedures)
☐ The risks or discomforts that are reasonably expected from the research, and a statement that "There may be unknown risks"
☐ The benefits to the participant or others that are reasonably expected from the research
☐ A statement of confidentiality that provides the participant a contact at the institution who may be reached if injury occurs or confidentiality is breached (this should be someone other than the researcher)
☐ A statement that participation is entirely voluntary and may be discontinued at any time
☐ A statement that withdrawal from participation will not result in denial of entitled benefits

Boston College IRB
Approved
October 18, 2019
Through August 20, 2020

FOR APPROVALS WITH NO CR REQUIRED:

Boston College
IRB Approved
September 4, 2020-
September 3, 2021

☐ Invasive biological, clinical or behavioral interventions require specific descriptions of the procedure
☑ The consent form must be signed and dated, or oral consent must be witnessed and signed and dated by the witness
☑ A statement and check box that indicates the participants have a copy of the informed consent document

Note: Individuals with added protections require both permission of a legal representative and assent of the individual.

Are you asking for a total waiver of consent: ○ Yes ◉ No

Are you asking for a waiver of the documentation of consent ◉ Yes ○ No

Are you asking for a waiver of the documentation of consent to waive the requirement for signature and name because this study will be conducted online: ○ Yes ◉ No

Please describe what elements of consent you wish to waive and provide a strong rationale for this alteration/waiver.
CHWs in Sierra Leone have the ability to read and write, but literacy levels are generally low. Due to the low literacy levels, we would like to obtain oral consent and are requesting a signature waiver. This is the same procedure we have used to obtain informed consent from interventionists in our Youth Forward study in Sierra Leone, which recruited lay workers from the community to deliver an evidence-based mental health intervention.

VII. Research with Minors

Instructions: Please complete this section only if you are using minors in your research. Otherwise, you may proceed to the next section. Research with minors requires either expedited or full board review.

Federal regulations recognize children as vulnerable subjects in research and require that special consideration be given to protecting their welfare. IRBs consider the potential benefits, risks, and discomforts of the research to children as well as their circumstances (e.g. age, health status, ability, etc.) and assess the justification for their inclusion in the research (*www.hhs.gov*).

Please provide a scientific or medical rationale for the inclusion of minors:
We will enroll children aged 6-36 months as previous iterations of the FSI-ECD have successfully targeted this group. Because the FSI-ECD targets reducing harsh disciplinary practices and increasing positive parent-child i

We will enroll children aged 6-36 months as previous iterations of the FSI-ECD have successfully targeted this group. Because the FSI-ECD targets reducing harsh disciplinary practices and increasing positive parent-child interactions, it is necessary to include children in observational assessments. Excluding children over age 36 months ensures we are targeting families with children in the home versus children attending pre-primary programs, which would increase barriers to intervention delivery with the entire family in the home. Excluding children under age 6 months ensures we are targeting families with children who have developed some degree of mobility and an ability to interact with caregivers rather than passively receive care.

nteractions, it is necessary to include children in observational assessments. Excluding children over age 36 months ensures we are targeting families with children in the home versus children attending pre-primary programs, which would increase barriers to intervention delivery with the entire family in the home. Excluding children under age 6 months ensures we are targeting families with children who have developed some degree of mobility and an ability to interact with caregivers rather than passively receive care.

Federal regulations classify research with minors into four categories based on the degree of risk. Please pick the appropriate category for your research.
☑ Category 1 **(45 CRF 46.404)**: Research involving minimal risk to minors
My research falls under this category because:
Caregivers must provide informed assent for children to participate in assessments and the FSI-ECD. During data collection, if a child presents with an issue necessitating additional care (e.g. malnutrition), the team member will notify the Program Manager and CHW supervisor to make a referral for additional services. Study RAs will be trained in use of an anthropometrics assessment tool to measure upper arm circumference as an indicator of malnutrition. This approach has been used successfully in prior research on the FSI-ECD in Rwanda to indicate malnutrition (NCT02510313). If there is a case of suspected abuse or neglect, the RA will enact the safety plan immediately to ensure that proper services are in place for the child and family.

Boston College IRB Approved
October 18, 2019
Through August 20, 2020

Are the risks involved in participating in this research those that parents would allow their minors to experience in the course of their everyday lives? ◉ Yes ○ No

FOR APPROVALS WITH NO CR REQUIRED:

Boston College IRB Approved
September 4, 2020-
September 3, 2021

Are adequate provisions in place for soliciting the assent of the minors and consent from the parents? **(45 CFR 46.408)** ◉ Yes ○ No

☐ Category 2 **(45 CFR 46.405)**: Research involving greater than minimal risk to minors with the prospect of direct benefit to the child:

☐ Category 3 **(45 CFR 46.406)**: Research involving greater than minimal risk with no direct benefit to the minors but it is likely to yield generalizable knowledge about the subject's disorder or condition

☐ Category 4 **(45 CFR 46.407)**: Research not meeting the criteria for Categories 1-3 that involves greater than minimal risk to healthy minors and presents no direct benefit to them but presents an opportunity to understand, prevent, or alleviate a serious problem affecting the health and welfare. *Please note: this fourth category of research with minors requires a special level of DHHS review after BC IRB review.*

Guidance on Remuneration: Minors may receive small gifts of appreciation for participation. Gifts should rarely be cash and should never be contingent upon study completion. Parents and guardians may be compensated for travel or time lost from work.

VIII. Performance Sites:

If you are conducting research at a site, (e.g. hospital, school, organization), the site must provide the BC IRB with evidence that they support the research being conducted at their location. If the site has an IRB, you should consult with that IRB to determine whether they require IRB approval from their institution in addition to IRB approval through BC. If they do not have an IRB, please provide a site permission letter. Site permission letters should be written on the letterhead of the institution and signed by a Principal or Administrator.

A. Are you conducting research at a site? ○ Yes ◉ No

IX. Acknowledgement

SUBMISSION OF A PROPOSAL TO THE BC IRB REQUIRES THAT THE PRINCIPAL INVESTIGATOR (AND MENTOR IF THE PI IS A STUDENT OR FELLOW) SIGN THIS PAGE AND READ COMPLETELY THE DEFINITION OF "SCIENTIFIC MISCONDUCT" AND ANSWER ALL "CONFLICT OF INTEREST" QUESTION GIVEN BELOW.

A. Scientific Misconduct

"Scientific Misconduct" shall be considered to include:

1. Fabrication, falsification, plagiarism or other unaccepted practices in proposing, carrying out or reporting results from research;
2. Material failure to comply with Federal requirements for the protection of human participants, researchers and/or the Public;
3. Failure to meet other material legal requirements governing research;
4. Failure to comply with established standards regarding author names on publications;
5. Failure to adhere to issues of confidentiality as provided in the participant consent form, the study protocol, and as outlined in the Code of Federal Regulations **(45 CFR 46)**.

Conflict of Interest

1. Are you or any member of your immediate family (spouse or domestic partner and/or dependent children) an officer, director, partner, trustee, Employee, advisory board member, or agent of (a) the external organization funding this Sponsored Project or (b) any external organization from which goods and services will be obtained under this Sponsored Project (including those to which you may be subcontracting a portion of the project work), (c) any external organization whose financial condition could benefit from the results of this Sponsored Project, or (d) any external organization having business dealings in an area related to the work under this Sponsored Project?

○ Yes (if so, describe in detail the nature and extent of the association on an attached sheet).
◉ No

2. Publicly-Traded Entities: Have you or any member of your immediate family derived income within the past year of $5,000 or more in a publicly-traded entity, or in the past year have you or any member of your immediate family owned equity interests in a public-traded interest, the fair market value of the equity being $5,000 or more?

○ Yes (If any of the following pertain, provide a full description on a separate sheet):

(a) The entity is co-funding this Sponsored Project;

(b) The income or equity is related the your university responsibilities (e.g. research, teaching, and service);

(c) The entity may provide goods and services under this Sponsored Project (including those to which you may be subcontracting a portion of the project work);

(d) The entity's financial condition may benefit from the results of this Sponsored Project; or

(e) The entity has business dealings in an area related to the work under this Sponsored Project?

Boston College IRB
Approved
October 18, 2019
Through August 20, 2020

FOR APPROVALS WITH NO CR REQUIRED:

Boston College
IRB Approved
September 4, 2020-
September 3, 2021

◉ No

3. Non-Publicly Traded (i.e. Privately Held) Entities: Have you or any member of your immediate family derived income within the past year of $5,000 or more in a non- publicly traded entity, or in the past year have you or any member of your immediate family owned any equity interests in a non-publicly traded entity?

○ Yes (If any of the following pertain, provide a full description on a separate sheet):

(a) The entity is co-funding this Sponsored Project;

(b) The income or equity is related the your university responsibilities (e.g. research, teaching, and service);

(c) The entity may provide goods and services under this Sponsored Project (including those to which you may be subcontracting a portion of the project work);

(d) The entity's financial condition may benefit from the results of this Sponsored Project; or

(e) The entity has business dealings in an area related to the work under this Sponsored Project?

◉ No

Research Staff

Added Personnel	Staff Type	EPPN	Date of IRB Training Certificate
Farrar, Jordan	Research Assistant	farrarjb	01/23/23
**Email**			
farrarjb@bc.edu			

Added Personnel	Staff Type	EPPN	Date of IRB Training Certificate
Betancourt, Theresa	Co-PI	betancth	10/11/21
**Email**			
Theresa.Betancourt@bc.edu			

Added Personnel	Staff Type	EPPN	Date of IRB Training Certificate
Rutirasiri, Chokdee	Research Assistant	rutirasi	11/28/18
**Email**			
chokdee.rutirasiri@bc.edu			

Added Personnel	Staff Type	EPPN	Date of IRB Training Certificate
Esliker, Rebecca	Research Assistant	REsliker	05/27/20
**Email**			
ebesliker@gmail.com			

SIGNATURE OF PRINCIPAL INVESTIGATOR

Boston College IRB Approved October 18, 2019 Through August 20, 2020

The undersigned accept(s) responsibility for the study, including adherence to the ethical guidelines set forth in the Belmont Report, Declaration of Helsinki, the Nuremberg Code, the ethical principles of your discipline, the Common Rule and Boston College policies regarding protection of the rights and welfare of human participants participating in this study. In the case of student protocols, the faculty supervisor and the student share responsibility for adherence to policies.

FOR APPROVALS WITH NO CR REQUIRED:

Signed by Alethea Desrosiers on Jun 03, 2020

Boston College IRB Approved September 4, 2020- September 3, 2021

SIGNATURE OF FACULTY RESEARCH SUPERVISOR- REQUIRED FOR STUDENT RESEARCH

By signing this form, I certify that:

Boston College IRB Approved
October 18, 2019
Through August 20, 2020

FOR APPROVALS WITH NO CR REQUIRED:

Boston College IRB Approved
September 4, 2020-
September 3, 2021

Alethea Desrosiers

Boston College School of Social Work
Chestnut Hill, MA 02467
alethea.desrosiers@bc.edu

EDUCATION

2011 **PhD, Clinical Psychology**
Teachers College, Columbia University, New York, NY

2007 **Msc, Clinical Psychology**
Teachers College, Columbia University, New York, NY

2004 **MA, Psychology in Education**
Teachers College, Columbia University, New York, NY

2001 **BA, Psychology**
McDaniel College, Westminster, MD

CAREER/ACADEMIC APPOINTMENTS

2019-present **Research Assistant Professor**
Boston College School of Social Work, Chestnut Hill, MA

2017-2019 **Visiting Scholar**,
Boston College School of Social Work, Chestnut Hill, MA

2017-2018 **Staff Counselor,**
Amherst College, Student Counseling Center, Amherst, MA

2016-2017 **Visiting Scientist**,
Harvard School of Public Health, Department of Global Health and Population, Boston, MA

2012-2016 **Associate Research Scientist,**
Yale School of Medicine, Department of Psychiatry, New Haven, CT

2011-2012 **Postdoctoral Fellow, T32 Substance Abuse Prevention Training Grant**
Yale School of Medicine, Department of Psychiatry, New Haven, CT

2010-2011 **Internship, Clinical Psychology**
Beth Israel Medical Center, Department of Psychiatry, New York, NY

PROFESSIONAL HONORS AND AWARDS

2012 Center for Interdisciplinary Research on AIDS (CIRA), Research Affiliate
2012 National Institute of Health, Loan Repayment Program Award
2006 Jodie Lane Scholarship, Psychology Department, Teachers College
2001 Rotary Ambassadorial Scholarship, University of Manchester, England
2001 Phi Beta Kappa
2001 Departmental Honors, Psychology, McDaniel College
1997-2001 Full Tuition Academic Scholarship, McDaniel College

Boston College IRB Approved
October 18, 2019
Through August 20, 2020

FOR APPROVALS WITH NO CR REQUIRED:

Boston College IRB Approved
September 4, 2020-
September 3, 2021

PROFESSIONAL LICENSES

2018-present Psychology License, Commonwealth of Massachusetts

RESEARCH EXPERIENCE

2019-present **Boston College School of Social Work, Research Program on Children and Adversity**
Research Assistant Professor
Chestnut Hill, Massachusetts

Expanding the Reach of Evidence-Based Mental Health Treatment: Diffusion and Spillover of Mental Health Benefits Among Peer Networks and Caregivers of Youth Facing Compounded Adversity in Sierra Leone
R01 MH117359-01 (NIMH)

Role: Co-investigator for an implementation science research study to investigate diffusion and spillover of mental health benefits of a CBT-based intervention to promote emotion regulation and daily functioning among youth facing compounded adversity in Sierra Leone.

Youth Forward: Capacity Building in Alternate Delivery Platforms and Implementation Models for Bringing Evidence-Based Behavior Interventions to Scale for Youth Facing Adversity in West Africa
U19 MH109989 (NIMH)
Principal Investigator: Theresa Betancourt, Sc.D.
Role: Co-investigator for a hybrid effectiveness-implementation trial to investigate a collaborative team approach to scaling up and sustaining a CBT-based intervention for at-risk youth in Sierra Leone and the effectiveness of the intervention delivered via an employment promotion program.

2018-2019 **World Health Organization**
Consultant

Role: To develop an individual and community level sexual and reproductive health-infused psychosocial intervention to improve reproductive health service usage and well-being among adolescent and young adult female Syrian refugees in Jordan, Turkey, and Lebanon.

2017-2019 **Boston College School of Social Work**
Visiting Scholar
Chestnut Hill, Massachusetts

Role: Led resubmission of an NIMH grant proposal to examine diffusion and spillover of mental health benefits of a CBT-based intervention among youth facing compounded adversity in Sierra Leone; coordinated multidisciplinary teams of research partners in Boston and Sierra Leone.

2016-2017 **Harvard School of Public Health, Department of Global Health and Population**
Visiting Scientist
Boston, Massachusetts

Boston College IRB Approved October 18, 2019 Through August 20, 2020

Role: Led development of an NIMH implementation science grant to examine diffusion and spillover of mental health benefits of a CBT-based intervention to promote emotion regulation and functioning among youth facing compounded adversity in Sierra Leone.

FOR APPROVALS WITH NO CR REQUIRED:

Boston College IRB Approved September 4, 2020- September 3, 2021

2012-2016 **Yale School of Medicine, Department of Psychiatry**
Associate Research Scientist
New Haven, Connecticut

Behavioral Drug and HIV Risk Reduction with MMT in China
5 R01 DA026797-03 (NIDA)
Principal Investigator: Marek Chawarski, Ph.D.
Role: Co-investigator for a randomized clinical trial comparing the efficacy of MMT combined with one of three manual-guided counseling approaches in reducing HIV risk behaviors and illicit opiate use in China.

Naltrexone and Behavioral Drug and HIV Risk Reduction Counseling in Russia
5 R01 DA027405-03 (NIDA)
Principal Investigator: Marek Chawarski, Ph.D.
Role: Co-investigator on a randomized clinical trial evaluating the efficacy of two medication formulations of naltrexone and two manually-guided counseling conditions in reducing HIV risk behaviors and illicit opiate use in Russia.

2012-2014 ***Drug Counseling and Abstinent-Contingent Take Home Buprenorphine in Malaysia***
R01 DA014718-05A1 (NIDA)
Principal Investigators: Richard Schottenfeld, M.D., Marek Chawarski, Ph.D.
Role: Co-investigator on a randomized clinical trial evaluating whether behavioral counseling, abstinent-contingent take-home buprenorphine, or their combination improves efficacy of standard treatment for opiate dependence in Malaysia.

2011-2012 **Yale School of Medicine, Department of Psychiatry**
Postdoctoral Fellow, **Research Training Program in Substance Abuse Prevention**
T32 DA019426 (NIDA)

Intervention to Reduce Acute Stress and HIV Risk in Newly Diagnosed Men
R34 MH087216-01A2 (NIMH)
Principal Investigator: Nathan Hansen, Ph.D.
Role: Provided STAY Healthy intervention to newly diagnosed HIV+ men who have sex with men (MSM) and contributed to development of a revised intervention manual.

HIV/STD Risk Among Young Expectant Fathers: Relationship Attachment and Transition
5 R01 MH075685-05 (NIMH)
Principal Investigator: Trace Kershaw, Ph.D.
Role: Collaborated with a multidisciplinary team to analyze a longitudinal data set of pregnant and parenting adolescent couples, develop manuscripts, and present findings.

The Healthy Girls Project
Yale Institute for Social Policy Studies
Principal Investigator: Susan Nolen-Hoeksema, Ph.D.
Role: Worked on implementation of an emotion regulation intervention for prevention of substance abuse, mood disorders, and risk behavior in ethnically diverse adolescent females.

Boston College IRB Approved
October 18, 2019
Through August 20, 2020

2009-2010 **Brown University**
Research Assistant
Providence, Rhode Island

FOR APPROVALS WITH NO CR REQUIR[illegible]

Boston College IRB Approved
September 4, 2020-
September 3, 2021

Meditation Training in Early Adolescents and College Students
(Mind and Life Institute)
Principal Investigator: Willoughby Britton, Ph.D.
Role: Coordinated and implemented a study investigating meditation practice and mental health in college students; analyzed data and presented findings at national conferences.

2009-2010 **Brown University, Center for Alcohol and Addiction Studies**
Intervention Specialist

Effects of the Family Check-up on Adolescents with Alcohol Related Events and their Siblings
R01 AA017659 (NIAAA)
Principal Investigator: Anthony Spirito, Ph.D.
Role: Provided a brief motivational interviewing based intervention to adolescents with alcohol infractions recruited from juvenile and family courts, analyzed video-taped parent-child discussion sessions, and conducted feedback sessions with families.

Substance Abuse Prevention for Preadolescents with Psychiatric Disorders
R21 DA024207 (NIDA)
Principal Investigator: Anthony Spirito, Ph.D.
Role: Provided a motivational interviewing based intervention to substance abusing adolescents with co-occurring psychiatric diagnoses and their parents; developed study protocols and intervention manuals; conducted training sessions with new staff.

2004-2008 **Teachers College, Columbia University**
Project Director
New York, New York

Religion and Resilience in Adolescents
(William T. Grant Foundation)
Principal Investigator: Lisa Miller, Ph.D.
Role: Coordinated and implemented a study on religion and mental health in adolescents; developed community partnerships with youth organizations; managed large quantitative databases; analyzed data and presented findings at national and international conferences.

2004-2008 **National Development & Research Institute**
Senior Research Assistant
New York, New York

Computer-Assisted HIV Prevention for Young Drug Users
3 R01 DA015964-04S1 (NIDA)
Principal Investigator: Lisa Marsch, Ph.D.
Role: Coordinated and implemented a study on HIV prevention education for substance abusing adolescents; collaborated with community drug treatment programs; conducted informed consent sessions; developed and managed study files and databases.

Boston College IRB
Approved
October 18, 2019
Through August 20, 2020

Expanding Computer-Based Drug Abuse Prevention
5 R42 DA016083-03 (NIDA)
Principal Investigator: Lisa Marsch, Ph.D

FOR APPROVALS WITH NO CR REQUIRED:

Boston College
IRB Approved
September 4, 2020-
September 3, 2021

Role: Coordinated and implemented a study on computer-based drug abuse prevention in elementary school students; conducted focus groups and feedback sessions with school- aged children; organized and maintained participant databases.

Science-Based Treatment for Opioid-Dependent Adolescents
5 R01 DA018297 04 (NIDA)
Principal Investigator: Lisa Marsch, Ph.D.
Role: Facilitated recruitment, initiated community partnerships, and facilitated participant recruitment efforts; organized and maintained files and databases for study participants.

PEER-REVIEWED PUBLICATIONS

Desrosiers, A., Betancourt, T., Kergoat, Y., Servilli, C., Say, L., Kobeissi, L. (2020). A systematic review of sexual and reproductive health interventions for young people in humanitarian and lower-and-middle-income country settings. *BMC Public Health*, 20, 666.

Desrosiers, A., Kumar, P., Dayal, A., Alex, L., Akram, T., Betancourt, T. (2020). Diffusion and spillover effects of an evidence-based mental health intervention among peers and caregivers of high risk youth in Sierra Leone: study protocol. *BMC Psychiatry*, 20, 85.

Lauckner, C., **Desrosiers, A.**, Muilenburg, J., Kilanin, A., Genter, E., & Kershaw, T. (2019). Social media photos of substance use and their relationship to attitudes and behaviors among minority emerging adult men. *Journal of Adolescence*, 77, 152-162.

Desrosiers, A., Vine, V., & Kershaw, T. (2019). "R U Mad?": Computerized text analysis of affect in social media relates to stress and substance use among emerging adult, ethnic minority males. *Anxiety, Stress and Coping*, 32, 109-123.

Cornelius, T., **Desrosiers, A.**, & Kershaw, T. (2017). Smoking together during pregnancy: Are there relationship benefits? *Social Science and Medicine*, 192, 30-35.

Rizor, A., Callands, T., **Desrosiers, A.,** & Kershaw, T. (2017). (S)He's gotta have it: Emotion regulation, emotional expression, and sexual risk behavior in emerging adult couples. *Sexual Addiction & Compulsivity,* 24, 203-216.

Chooi, W., **Desrosiers, A**., Zaharim, N., Ahmad, I., Yasin, M., Jaaper, S., Schottenfeld, R., Vicknasingam, B., & Chawarski, M. (2017). Early initiation of amphetamine-type stimulants (ATS) use associated with lowered cognitive performance among individuals with co-occurring opioid and ATS use disorders in Malaysia. *Journal of Psychoactive Drugs*, 49, 326-332.

Desrosiers, A., Blokhina, E., Zvartua, E., Krupitsky, E., Schottenfeld, R., & Chawarski, M. (2016). Psychiatric symptoms, quality of life, and HIV status among people using opioids in Saint Petersburg, Russia. *Drug and Alcohol Dependence*, 172, 60-65.

Cornelius, T., **Desrosiers, A.**, & Kershaw, T. (2016). Spread of health behaviors in young couples: How relationship power shapes relational influence. *Social Science & Medicine*, 165, 46-55.

Boston College IRB
Approved
October 18, 2019
Through August 20, 2020

Sipsma, H., Callands, T., **Desrosiers, A.**, Magriples, M., Jones, K., Albritton, T., & Kershaw, T. (2016). Exploring trajectories and predictors of depressive symptoms among young couples during their transition to parenthood. *Maternal and Child Health Journal,* 20, 2372-2381.

FOR APPROVALS WITH NO CR REQUIRED.

Boston College
IRB Approved
September 4, 2020-
September 3, 2021

Desrosiers, A., Chooi, W., Zaharim, N., Ahmad, I., Yasin, A., Jaapar, S., Schottenfeld, R., Vicknasingam, B., Chawarski, M. (2016). Emerging drug use trends in Kelantan, Malaysia. *Journal of Psychoactive Drugs*, 48, 218-216.

Desrosiers, A., Sipsma, H., Kershaw, T., Divney, A., & Magriples, U. (2015). Emotion expression and substance use in newly parenting adolescents and young adults. *Journal of Clinical Psychology*, 71, 684-695.

Desrosiers, A., Thompson, A., Divney, A., Magriples, A., & Kershaw, T. (2015). Romantic partner influences on substance use among pregnant and parenting young couples. *Journal of Public Health*, 38, 300-307.

Zhou, W., Wang, X., Zhou, S., Xie, N., Liu, P., Luo, L., Peng, J., Liu, M., **Desrosiers, A**., Schottenfeld, R., Chawarski, M. C. (2014). Hepatitis C seroconversion in methadone maintenance treatment programs in Wuhan, China. *Addiction*, 110, 796-802.

Zhang, Y., Xu, Z., Zhang, S., **Desrosiers, A.,** Schottenfeld, R. S., Chawarski, M. C. (2014). Profiles of psychiatric symptoms among amphetamine type stimulant and ketamine using inpatients in Wuhan, China. *Journal of Psychiatric Research*, 53, 99-102.

Desrosiers, A., Vine, V., Curtiss, J., & Klemanski, D. (2014). Observing Nonreactively: A conditional process model linking mindfulness facets, cognitive emotion regulation strategies, and depression and anxiety symptoms. *Journal of Affective Disorders*, 165, 31-37.

Desrosiers, A., Sipsma, H., Callands, T., Hansen, N., Divney, A., Magriples, U., & Kershaw, T. (2014). "Love Hurts": Romantic attachment and depressive symptoms in pregnant adolescent and young adult couples. *Journal of Clinical Psychology*, 70, 95-106.

Desrosiers, A., Vine, V., Klemanski, D., & Nolen-Hoeksema, S. (2013). Mindfulness and emotion regulation in depression and anxiety: Common and distinct mechanisms of action. *Depression and Anxiety*, 30, 654-661.

Desrosiers, A., Klemanski, D., & Nolen-Hoeksema, S. (2013). Mapping mindfulness facets onto dimensions of anxiety and depression. *Behavior Therapy*, 44, 373-384.

Nolen-Hoeksema, S., **Desrosiers, A**., & Wilsnack, S. C. (2013). Predictors of alcohol-related problems among depressed and non-depressed women. *Journal of Affective Disorders*, 150, 967-973.

Marsch, L. A., Grabinski, M. J., Bickel, W. K., **Desrosiers, A**., Guarino, H., Muehlbach, B., Solhkhah, R., Taufique, S., & Acosta, M. (2011). Computer-assisted HIV prevention for youth with substance use disorders. *Substance Use and Misuse*, 46, 46-56.

Desrosiers, A. (2011). Development of religion and spirituality across the lifespan. In J. Aten, K. O'Grady, & E. Worthington (Eds.). *The Psychology of Religion and Spirituality for Clinicians: Using Research in Your Practice*.

Desrosiers, A., Kelly, B., & Miller, L. (2010). Parent and peer facilitation of relational spirituality in adolescents and young adults. *Psychology of Religion and Spirituality*, vol. 3, 39-54.

Boston College IRB Approved
October 18, 2019,
Through August 20, 2020

Desrosiers, A. & Miller, L. (2008). Substance use versus anxiety in adolescents: Are some disorders more spiritual than others? *Research in the Social Scientific Study of Religion*, 9, 137-154.

FOR APPROVALS WITH NO CR REQUIRED:

Desrosiers, A. & Miller, L. (2007). Relational spirituality and depression in adolescent girls. *Journal of Clinical Psychology*, 63, 1021-1037.

Boston College
IRB Approved
September 4, 2020-
September 3, 2021

MANUSCRIPTS UNDER REVIEW

Desrosiers, A., Freeman, J., Mitra, R., Farrar, J., Borg, R., & Betancourt, T. Alternative delivery platforms for expanding evidence-based mental health interventions for youth in Sierra Leone: A pilot study. [Manuscript submitted for publication].

Su, S., Frounfelker, R., **Desrosiers, A.**, Brennan R. T., Farrar, J., & Betancourt T. S. Classifying childhood war trauma exposure: Latent profile analyses of Sierra Leone's former child soldiers. [Manuscript submitted for publication].

Cornelius, T., **Desrosiers, A.**, & Kershaw, T. Does drinking really make you popular? The effects of alcohol use on social ties. [Manuscript submitted for publication].

SYMPOSIUM AND POSTER PRESENTATIONS

Desrosiers, A. (January 2020). Alternative delivery platforms for expanding evidence-based mental health interventions for youth in Sierra Leone: A pilot study. Invited oral presentation at the Global & Local Center for Mental Health Disparities at Boston Medical Center, Boston, MA.

Desrosiers, A., Mitra, R., Borg, R., Farrar, J., & Betancourt, T. (December 2019). Fidelity monitoring of a cognitive-behavioral therapy-based intervention for youth facing compounded adversity in Sierra Leone. Poster session at the Annual Academy Health Conference on the Science of Dissemination and Implementation in Health, Arlington, VA.

Desrosiers, A. (July 2018). Depressive disorders. Invited oral presentation, American University of the Carribean, St. Maarten.

Desrosiers, A. (July 2017). Linking mindfulness facets, cognitive emotion regulation strategies, and depression and anxiety symptoms. Invited oral presentation, University of Medicine and Health Sciences, St. Kitts.

Desrosiers, A. (June 2017). Positive and negative emotion in social media: Associations with substance use among ethnic minority, emerging adult males. Poster session presented at the biannual meeting of the International Society for Child Indicators, Montreal, Canada.

Desrosiers, A. (April 2017). Relating mindfulness facets to depression and anxiety symptoms in a clinical sample of adults. Invited oral presentation at departmental grand rounds, St. George's University, Grenada.

Desrosiers, **A.** (February 2017). "LOL, I feel you": Linguistic indicators of emotion in social med stress, and substance use among young, minority males. Invited oral presentation at the department of psychology colloquium series, Williams College, MA.

Cornelius, T., **Desrosiers**, **A.**, & Kershaw, T. (January 2017). Drinking doesn't always make you popular. Poster session presented at the annual meeting of the Society of Personality and Social Psychology, San Antonio, TX.

Lauckner, C., **Desrosiers, A.**, Muilenburg, J., Kilanin, A., Genter, E., & Kershaw, T. (March 2016). Social media photos of substance use and their relationship to attitudes and behaviors among minority emerging adult men. Poster session presented at the annual meeting of behavioral medicine, Washington, D.C.

Boston College IRB Approved
October 18, 2019
Through August 20, 2020

FOR APPROVALS WITH NO CR REQUIRED:

Boston College
IRB Approved
September 4, 2020-
September 3, 2021

Desrosiers, A., Blokhina, E., Krupitsky, E., Zvartua, E., Schottenfeld, R. S., & Chawarski, M. (June 2015). HIV status and mental health among opioid using adults in Russia. Poster session presented at the annual meeting of the College on Problems of Drug Dependence, Phoenix, AZ.

Mohd-Zaharim, N., Chawarski, M., Chooi, W., **Desrosiers, A.**, Schottenfeld, R. S., & Kasinather, V. (March 2015). Behavioral drug and risk reduction counseling (BDRC): A new psychosocial treatment for co-occurring opioid and amphetamine-type stimulant dependent individuals in Kota Bharu, Malaysia. Poster session presented at the annual international conference of the Association for Psychological Science, Amsterdam, The Netherlands.

Desrosiers, A., Vine, V., Curtiss, J., Klemanski, D. H. (November 2014). Mindfulness facets interact to influence emotion regulation strategies and depression and anxiety symptoms. Poster session presented at the annual conference of the Association for Behavioral and Cognitive Therapies, Philadelphia, PA.

Desrosiers, A., Blokhina, E., Krupitsky, E., & Chawarski, M. (June 2014). Gender differences in psychiatric symptoms among opiate dependents patients in Russia. Poster session presented at the annual meeting of the College on Problems of Drug Dependence, San Juan, Puerto Rico.

Kasinather, B. V., **Desrosiers, A.**, Schottenfeld, R., & Chawarski, M. (June 2014). International Collaboration between academic institutions: University Sains Malaysia and Yale School of Medicine. Poster session presented at the annual meeting of the College on Problems of Drug Dependence, San Juan, Puerto Rico.

Syarif, Z., Susami, H., Sarasvita, R., Utami, D., Nurhidayat, A., **Desrosiers, A.,** Chawarski, M., Woody, G., & Metzger, D. (June 2014). Benzodiazepine use among methadone maintenance treatment patients in Jakarta, Indonesia. Poster session presented at the annual meeting of the College on Problems of Drug Dependence, San Juan, Puerto Rico.

Kasinather, V. B., Chawarski, M., Chooi, W. T., Zaharim, N. M., **Desrosiers, A.**, Yasin, A. M., Ahmad, I., & Schottenfeld, R. (November 2013). Characteristics of individuals with co-occurring opioid and amphetamine type stimulant dependence in Kota Bharu, Malaysia. Poster session at the annual conference of the International Society of Addiction Medicine, Kuala Lumpar, Malaysia.

Zaharim, N. M., Chooi, W. T., Kasinather, V. B., Yasin, A. M., Ahmad, I., **Desrosiers, A.**, Schottenfeld, R. S., Chawarski, M. C. (November 2013). A counseling model for co-ocurring opioid and amphetamine type stimulant dependence in Kota Bharu, Malaysia. Oral presentation in symposium (chair: K. Kamali) Psychosocial intervention in the treatment of addiction, at the annual conference of the International Society of Addiction Medicine, Kuala Lumpar, Malaysia.

Kershaw, T., Sipsma, H., **Desrosiers, A.**, Callands, T., Albritton, T., Magriples, U., & Gordon, D. M. (November 2013). Interconnectedness of parents' relationship functioning and mental health. Poster session presented at the annual conference of the American Public Health Association, Boston, MA.

Sipsma, H., **Desrosiers, A.**, Callands, T., Gordon, D., Magriples, U., Jones, K., & Kershaw, T. (November 2013). Mental health of young parents from pregnancy through 1-year postpartum. Poster session presented at the annual conference of the American Public Health Association, Boston, MA.

Boston College IRB
Approved
October 18, 2019
Through August [illegible], 2020

Desrosiers, A. (October 2013). Mapping out mindfulness: Relating mindfulness facets to depression and anxiety symptoms. Invited oral presentation at the clinical psychology department colloquium series, Teachers College, Columbia University.

FOR APPROVALS WITH NO CR REQUIRED:

Boston College
IRB Approved
September 4, 2020-
September 3, 2021

Desrosiers, A., Sipsma, H., Kershaw, T. (June 2013). Negative affect, emotional awareness, and substance use in adolescents and young adults. Poster session presented at the annual meeting of the College on Problems of Drug Dependence, San Diego, CA.

Desrosiers, A., Nolen-Hoeksema, S., & Klemanski, D. H. (November 2012). Mindfulness in depression and anxiety: Mechanisms of action via emotion regulation. Oral presentation in symposium (chair: Moria J. Smoski), Emotion regulation in psychopathology: What works for whom?, presented at the annual meeting of the Association for Behavioral and Cognitive Therapies, National Harbor, MD.

Desrosiers, A., Nolen-Hoeksema, S., & Klemanski, D. H. (November 2012). Modeling mindfulness: How mindfulness facets map onto dimensions of anxiety and depression. Poster session presented at the annual conference of the Association for Behavioral and Cognitive Therapies, National Harbor, MD.

Klemanski, D. H., Millstein, D. J., **Desrosiers, A**., & Nolen-Hoeksema, S. (November 2012). Effects of worry on quality of life in a clinical sample: The role of psychological flexibility, emotion regulation, and mindfulness. Poster session presented at the annual conference of the Association for Behavioral and Cognitive Therapies, National Harbor, MD.

Desrosiers, A., Sipsma, H., Hansen, N., Callands, T., & Kershaw, T. (June 2012). “Love Hurts”: Romantic attachment and depression in pregnant adolescent couples. Poster session presented at the Society for Psychotherapy Research Conference. Virginia Beach, VA.

Desrosiers, A., Vine, V., & Nolen-Hoeksema, H. (December 2011). Mindfulness in depression and anxiety: common and distinct mechanisms of action. Poster session presented at the Affective Science Institute meeting, Boston, MA.

Desrosiers, A., Donalds, R., & Britton, W. (June 2010). School-based mindfulness training may offer clinical benefits. Poster presented at the World Congress for Cognitive and Behavioral Therapies, Boston, MA.

Desrosiers, A. & Miller, L. (August 2008). Relational spirituality and psychopathology in adolescents. Invited Oral Presentation in Symposium (Chair: Chris Boyatzis), Religiosity and psychological adjustment in adolescents, college students, and elderly, presented at the annual conference of the American Psychological Association, Boston, MA.

Marsch, L. A., Bickel, W. K., Grabinski, M. J., **Desrosiers, A.** & Guarino, H. (August 2006). Computer delivery of HIV and hepatitis prevention in substance abuse treatment. Invited oral presentation in symposium (Chair: Jesse Dallery), Innovations in technology for substance abuse treatment, at the annual conference of the American Psychological Association, New Orleans, LA.

Marsch, L. A., Bickel, W.K., **Desrosiers, A.** & Guarino, H. (June 2005). Computer-delivered HIV and other disease prevention for substance-abusing youth. Paper presented in Oral Communications Session VIII “AIDS/HIV” (Co-Chairs: Marc Auriacombe & Lisa Marsch) at the annual meeting of the College on Problems of Drug Dependence, Orlando, FL.

Miller, L., **Desrosiers, A.**, Kelley, B., & Peters, T. (March, 2006). Spirituality and mental health in adolescents. Key Note Address, World Congress on Spirituality and Health, Monterey, Mexico.

Boston College IRB
Approved
October 18, 2019
Through August 30, 2020

TEACHING EXPERIENCE

FOR APPROVAL OF WORDING CERTIFICATED.

Boston College School of Social Work
Lecturer

Boston College
IRB Approved
September 4, 2020-
September 3, 2021

2020 Applied Methods in Multicultural Mental Health and Social Work Research (SCWK 9963 01)

Harvard T. H. Chan School of Public Health/Harvard School of Medicine
Lecturer

2020 Case Studies in Global Mental Health Delivery (GHP 208/SM 518.0)

Yale University, Department of Psychology
Lecturer

2012 Introduction to Psychology (Psych S110 01)
2013 Introduction to Psychology (Psych S110 01)

Yale School of Medicine, Department of Psychiatry
Co-Instructor

2012 Behavioral Drug and HIV-risk Reduction Counseling training workshop, Wuhan China
2013 Behavioral Drug and HIV-risk Reduction Counseling training workshop, Kota Bharu, Malaysia
2013 Behavioral Drug and HIV-risk Reduction Counseling training workshop, St. Petersburg, Russia
2014 Behavioral Drug and HIV-risk Reduction Counseling training workshop, St. Petersburg, Russia

Teachers College, Columbia University,
Department of Clinical and Counseling Psychology Co-Instructor

2009 Psychotherapy, religious diversity, and spirituality

CLINICAL EXPERIENCE

2017-2018 **Amherst College**
Staff Counselor
Amherst, MA

Role: Provided intake evaluations and individual psychotherapy to college students; provided consultation services, risk assessment, and crisis intervention; participated in campus outreach.

2010-2011 **Beth Israel Medical Center**
Psychology Intern
New York, New York

Psychiatric Inpatient Services for Adults, Division of Addictions
Role: Conducted intake evaluations and provided individual psychotherapy to adults in inpatient treatment for co-occurring substance use and psychiatric disorders; collaborated with multidisciplinary teams to coordinate care and disposition.

Psychiatric Outpatient Services for Adults (POSA)
Role: Conducted intake evaluations for adults; provided individual, family, and group psychotherapy to adults with Axis I and Axis II disorders, using multiple treatment approaches; provided psychological testing using projective and objective instruments.

Boston College IRB Approved
October 18, 2019
Through August 20, 2020

FOR APPROVALS WITH NO CR REQUIRED

Boston College
IRB Approved
September 4, 2020-
September 3, 2021

Psychiatric Outpatient Services for Children (POSC)
Role: Conducted intake evaluations for children, provided individual psychotherapy for adolescents and collateral parent sessions; provided psychological testing for children to assess cognitive and socio-emotional functioning; provided consultation-liaison services.

Yarmon Neurobehavior and Alzheimer's Disease Center
Role: Completed neuropsychological evaluations for adults with cognitive deficits; provided individual and group psychotherapy to older adults with cognitive disorders.

2009-2010 **Brown University, Center for Alcohol and Addiction Studies**
Intervention Specialist
Providence, Rhode Island

Role: provided motivational interviewing to Hispanic adults with alcohol use/abuse concerns for a study investigating a culturally adapted form of motivation interviewing.

2009-2010 **Rhode Island Hospital, Adult Partial Hospitalization Program**
Adult Psychotherapist
Providence, Rhode Island

Role: Provided individual and group psychotherapy to adults with Axis I (e.g., mood and anxiety disorders, substance abuse disorders) and Axis II disorders; conducted intake evaluations; collaborated with a multidisciplinary team; coordinated discharge planning.

2008-2009 **Connecticut College, Student Counseling Services**
Psychotherapist
New London, Connecticut

Role: Provided intake evaluations and individual psychotherapy to undergraduates with substance abuse disorders, mood and anxiety disorders, adjustment disorders, and eating disorders; participated in campus outreach activities for suicide prevention.

2007-2008 **St. Luke's-Roosevelt Hospital Center, Child and Family Institute (CFI)**
Child Psychotherapist
New York, New York

Role: Provided weekly individual, family, and group outpatient psychotherapy to ethnically diverse children and adolescents; provided parent training and education.

2005-2008 **Teachers College, Columbia University, Dean-Hope Center Child and Adult Psychotherapist**
New York, New York

Role: Provided individual outpatient psychotherapy to children and adults integrating multiple treatment approaches (e.g., CBT, psychodynamic, family systems); intake evaluations for children and adults; psychological testing for children and adults.

Boston College IRB Approved
October 18, 2019
Through August [illegible], 20[illegible]

PROFESSIONAL SERVICE

FOR APPROVALS [illegible] REQUIRED

Professional Affiliations:
2005-present American Psychological Association (APA)

Boston College
IRB Approved
September 4, 2020-
September 3, 2021

Ad Hoc Reviewer:
Addiction
Depression and Anxiety
Drug and Alcohol Dependence International Journal of Drug Policy Journal of Clinical Psychology
Journal of Research on Adolescence Mindfulness
Psychiatry Research
Substance Abuse
Substance Abuse Treatment, Prevention, and Policy

Professional Training and Development:

2019	Ethical Issues in Technology and Mental Health, Worcester, MA
2018	Progressive Counting Training for Trauma, Amherst, MA
2018	Stereotype and Implicit Bias, Amherst, MA
2017	Transgender Health, Boston, MA
2016	CITI Training on Human Subjects Research, Boston, MA
2016	Yoga and Mindfulness for Kids, PESI, Springfield, MA
2015	Advance Mindfulness Techniques, PESI, Cromwell, CT
2013	Behavioral Drug and HIV-Risk Reduction Counseling Training, Yale University
2012	Mindfulness and Emotion Regulation Skills Group Training, Yale University
2010	Attachment, Self-Regulation, and Competency Training, Beth Israel Medical Center
2009	Motivational Interviewing Training, Brown University
2008	Diversity Training, Connecticut College
2007	Dialectical Behavior Therapy Training, St. Luke's-Roosevelt Hospital
2007	Diversity Training, St. Luke's-Roosevelt Hospital Center
2006	Detection of Child Abuse Training, Columbia University

Boston College IRB Approved
October 18, 2019
Through August 20, 2020

FOR APPROVALS WITH NO CR REQUIRED:

Boston College IRB Approved
September 4, 2020-
September 3, 2021

CITI PROGRAM

Completion Date 09-Oct-2019
Expiration Date 08-Oct-2022
Record ID 33706548

This is to certify that:

Alethea Desrosiers

Has completed the following CITI Program course:

**Human Research**	(Curriculum Group)
**Social/Behavioral Research Course**	(Course Learner Group)
**2 - Refresher Course**	(Stage)

Under requirements set by:

Boston College

CITI
Collaborative Institutional Training Initiative

Verify at www.citiprogram.org/verify/?w08ea8d1a-615c-48fb-841e-ff97466f6fbf-33706548

Boston College IRB Approved
October 18, 2019
Through August 20, 2020

FOR APPROVALS WITH NO CR REQUIRED:

Boston College IRB Approved
September 4, 2020-
September 3, 2021

COLLABORATIVE INSTITUTIONAL TRAINING INITIATIVE (CITI PROGRAM)

COMPLETION REPORT - PART 1 OF 2
COURSEWORK REQUIREMENTS*

* NOTE: Scores on this Requirements Report reflect quiz completions at the time all requirements for the course were met. See list below for details. See separate Transcript Report for more recent quiz scores, including those on optional (supplemental) course elements.

- **Name:** Theresa Betancourt (ID: 3144591)
- **Institution Affiliation:** Boston College (ID: 1155)
- **Institution Email:** theresa.betancourt@bc.edu
- **Institution Unit:** School of Social Work
- **Phone:** 617-5528251

- **Curriculum Group:** Human Research
- **Course Learner Group:** Social/Behavioral Research Course
- **Stage:** Stage 2 - Refresher Course

- **Record ID:** 28913180
- **Completion Date:** 12-Oct-2018
- **Expiration Date:** 11-Oct-2021
- **Minimum Passing:** 75
- **Reported Score*:** 100

REQUIRED AND ELECTIVE MODULES ONLY	DATE COMPLETED	SCORE
SBE Refresher 1 – History and Ethical Principles (ID: 936)	14-Oct-2015	2/2 (100%)
SBE Refresher 1 – Federal Regulations for Protecting Research Subjects (ID: 937)	14-Oct-2015	2/2 (100%)
SBE Refresher 1 – Informed Consent (ID: 938)	14-Oct-2015	2/2 (100%)
SBE Refresher 1 – Research with Prisoners (ID: 939)	14-Oct-2015	2/2 (100%)
SBE Refresher 1 – Research in Educational Settings (ID: 940)	15-Oct-2015	2/2 (100%)
SBE Refresher 1 - Instructions (ID: 943)	14-Oct-2015	No Quiz
SBE Refresher 1 – Defining Research with Human Subjects (ID: 15029)	14-Oct-2015	2/2 (100%)
SBE Refresher 1 – Privacy and Confidentiality (ID: 15035)	15-Oct-2015	2/2 (100%)
SBE Refresher 1 – Assessing Risk (ID: 15034)	14-Oct-2015	2/2 (100%)
SBE Refresher 1 – Research with Children (ID: 15036)	15-Oct-2015	2/2 (100%)
SBE Refresher 1 – International Research (ID: 15028)	15-Oct-2015	2/2 (100%)
SBE Refresher 2 – Federal Regulations for Protecting Research Subjects (ID: 15040)	15-Oct-2015	1/1 (100%)
SBE Refresher 2 – Defining Research with Human Subjects (ID: 15038)	15-Oct-2015	1/1 (100%)
SBE Refresher 2 – Research with Children (ID: 15043)	15-Oct-2015	1/1 (100%)
SBE Refresher 2 – Research in the Public Schools (ID: 15042)	15-Oct-2015	1/1 (100%)
SBE Refresher 2 – International Research (ID: 15045)	15-Oct-2015	1/1 (100%)
Boston College (ID: 12098)	12-Oct-2018	No Quiz

For this Report to be valid, the learner identified above must have had a valid affiliation with the CITI Program subscribing institution identified above or have been a paid Independent Learner.

Verify at: www.citiprogram.org/verify/?k9f5e70eb-9578-484c-813f-3c38e5a8b8e4-28913180

Collaborative Institutional Training Initiative (CITI Program)
Email: support@citiprogram.org
Phone: 888-529-5929
Web: https://www.citiprogram.org

Boston College IRB Approved
October 18, 2019
Through August 20, 2020

FOR APPROVALS WITH NO CR REQUIRED:

Boston College IRB Approved
September 4, 2020-
September 3, 2021

COLLABORATIVE INSTITUTIONAL TRAINING INITIATIVE (CITI PROGRAM)

COMPLETION REPORT - PART 2 OF 2
COURSEWORK TRANSCRIPT**

** NOTE: Scores on this Transcript Report reflect the most current quiz completions, including quizzes on optional (supplemental) elements of the course. See list below for details. See separate Requirements Report for the reported scores at the time all requirements for the course were met.

- **Name:** Theresa Betancourt (ID: 3144591)
- **Institution Affiliation:** Boston College (ID: 1155)
- **Institution Email:** theresa.betancourt@bc.edu
- **Institution Unit:** School of Social Work
- **Phone:** 617-5528251

- **Curriculum Group:** Human Research
- **Course Learner Group:** Social/Behavioral Research Course
- **Stage:** Stage 2 - Refresher Course

- **Record ID:** 28913180
- **Report Date:** 04-Nov-2018
- **Current Score**:** 100

REQUIRED, ELECTIVE, AND SUPPLEMENTAL MODULES	MOST RECENT	SCORE
SBE Refresher 1 – History and Ethical Principles (ID: 936)	14-Oct-2015	2/2 (100%)
SBE Refresher 1 - Instructions (ID: 943)	14-Oct-2015	No Quiz
SBE Refresher 1 – Federal Regulations for Protecting Research Subjects (ID: 937)	14-Oct-2015	2/2 (100%)
SBE Refresher 1 – Informed Consent (ID: 938)	14-Oct-2015	2/2 (100%)
SBE Refresher 1 – Research with Prisoners (ID: 939)	14-Oct-2015	2/2 (100%)
SBE Refresher 1 – Research in Educational Settings (ID: 940)	15-Oct-2015	2/2 (100%)
SBE Refresher 1 – International Research (ID: 15028)	15-Oct-2015	2/2 (100%)
SBE Refresher 1 – Defining Research with Human Subjects (ID: 15029)	14-Oct-2015	2/2 (100%)
SBE Refresher 1 – Assessing Risk (ID: 15034)	14-Oct-2015	2/2 (100%)
SBE Refresher 1 – Privacy and Confidentiality (ID: 15035)	15-Oct-2015	2/2 (100%)
SBE Refresher 1 – Research with Children (ID: 15036)	15-Oct-2015	2/2 (100%)
SBE Refresher 2 – Defining Research with Human Subjects (ID: 15038)	15-Oct-2015	1/1 (100%)
SBE Refresher 2 – Federal Regulations for Protecting Research Subjects (ID: 15040)	15-Oct-2015	1/1 (100%)
SBE Refresher 2 – Research in the Public Schools (ID: 15042)	15-Oct-2015	1/1 (100%)
SBE Refresher 2 – Research with Children (ID: 15043)	15-Oct-2015	1/1 (100%)
SBE Refresher 2 – International Research (ID: 15045)	15-Oct-2015	1/1 (100%)
Boston College (ID: 12098)	12-Oct-2018	No Quiz

For this Report to be valid, the learner identified above must have had a valid affiliation with the CITI Program subscribing institution identified above or have been a paid Independent Learner.

Verify at: www.citiprogram.org/verify/?k9f5e70eb-9578-484c-813f-3c38e5a8b8e4-28913180

Collaborative Institutional Training Initiative (CITI Program)
Email: support@citiprogram.org
Phone: 888-529-5929
Web: https://www.citiprogram.org

Boston College IRB
Approved
October 18, 2019
Through August 20, 2020

FOR APPROVALS WITH NO CR REQUIRED:

Boston College
IRB Approved
September 4, 2020-
September 3, 2021

COLLABORATIVE INSTITUTIONAL TRAINING INITIATIVE (CITI PROGRAM)

COMPLETION REPORT - PART 1 OF 2
COURSEWORK REQUIREMENTS*

* NOTE: Scores on this Requirements Report reflect quiz completions at the time all requirements for the course were met. See list below for details. See separate Transcript Report for more recent quiz scores, including those on optional (supplemental) course elements.

- **Name:** Theresa Betancourt (ID: 3144591)
- **Institution Affiliation:** Boston College (ID: 1155)
- **Institution Email:** theresa.betancourt@bc.edu
- **Institution Unit:** School of Social Work
- **Phone:** 617-5528251

- **Curriculum Group:** Human Research
- **Course Learner Group:** Social/Behavioral Research Course
- **Stage:** Stage 2 - Refresher Course

- **Record ID:** 28913180
- **Completion Date:** 12-Oct-2018
- **Expiration Date:** 11-Oct-2021
- **Minimum Passing:** 75
- **Reported Score*:** 100

REQUIRED AND ELECTIVE MODULES ONLY	DATE COMPLETED	SCORE
SBE Refresher 1 – History and Ethical Principles (ID: 936)	14-Oct-2015	2/2 (100%)
SBE Refresher 1 – Federal Regulations for Protecting Research Subjects (ID: 937)	14-Oct-2015	2/2 (100%)
SBE Refresher 1 – Informed Consent (ID: 938)	14-Oct-2015	2/2 (100%)
SBE Refresher 1 – Research with Prisoners (ID: 939)	14-Oct-2015	2/2 (100%)
SBE Refresher 1 – Research in Educational Settings (ID: 940)	15-Oct-2015	2/2 (100%)
SBE Refresher 1 - Instructions (ID: 943)	14-Oct-2015	No Quiz
SBE Refresher 1 – Defining Research with Human Subjects (ID: 15029)	14-Oct-2015	2/2 (100%)
SBE Refresher 1 – Privacy and Confidentiality (ID: 15035)	15-Oct-2015	2/2 (100%)
SBE Refresher 1 – Assessing Risk (ID: 15034)	14-Oct-2015	2/2 (100%)
SBE Refresher 1 – Research with Children (ID: 15036)	15-Oct-2015	2/2 (100%)
SBE Refresher 1 – International Research (ID: 15028)	15-Oct-2015	2/2 (100%)
SBE Refresher 2 – Federal Regulations for Protecting Research Subjects (ID: 15040)	15-Oct-2015	1/1 (100%)
SBE Refresher 2 – Defining Research with Human Subjects (ID: 15038)	15-Oct-2015	1/1 (100%)
SBE Refresher 2 – Research with Children (ID: 15043)	15-Oct-2015	1/1 (100%)
SBE Refresher 2 – Research in the Public Schools (ID: 15042)	15-Oct-2015	1/1 (100%)
SBE Refresher 2 – International Research (ID: 15045)	15-Oct-2015	1/1 (100%)
Boston College (ID: 12098)	12-Oct-2018	No Quiz

For this Report to be valid, the learner identified above must have had a valid affiliation with the CITI Program subscribing institution identified above or have been a paid Independent Learner.

Verify at: www.citiprogram.org/verify/?k9f5e70eb-9578-484c-813f-3c38e5a8b8e4-28913180

Collaborative Institutional Training Initiative (CITI Program)
Email: support@citiprogram.org
Phone: 888-529-5929
Web: https://www.citiprogram.org

Boston College IRB
Approved
October 18, 2019
Through August 20, 2020

FOR APPROVALS WITH NO CR REQUIRED:

Boston College
IRB Approved
September 4, 2020-
September 3, 2021

COLLABORATIVE INSTITUTIONAL TRAINING INITIATIVE (CITI PROGRAM)

COMPLETION REPORT - PART 2 OF 2

COURSEWORK TRANSCRIPT**

** NOTE: Scores on this Transcript Report reflect the most current quiz completions, including quizzes on optional (supplemental) elements of the course. See list below for details. See separate Requirements Report for the reported scores at the time all requirements for the course were met.

- **Name:** Theresa Betancourt (ID: 3144591)
- **Institution Affiliation:** Boston College (ID: 1155)
- **Institution Email:** theresa.betancourt@bc.edu
- **Institution Unit:** School of Social Work
- **Phone:** 617-5528251

- **Curriculum Group:** Human Research
- **Course Learner Group:** Social/Behavioral Research Course
- **Stage:** Stage 2 - Refresher Course

- **Record ID:** 28913180
- **Report Date:** 04-Nov-2018
- **Current Score**:** 100

REQUIRED, ELECTIVE, AND SUPPLEMENTAL MODULES	MOST RECENT	SCORE
SBE Refresher 1 – History and Ethical Principles (ID: 936)	14-Oct-2015	2/2 (100%)
SBE Refresher 1 - Instructions (ID: 943)	14-Oct-2015	No Quiz
SBE Refresher 1 – Federal Regulations for Protecting Research Subjects (ID: 937)	14-Oct-2015	2/2 (100%)
SBE Refresher 1 – Informed Consent (ID: 938)	14-Oct-2015	2/2 (100%)
SBE Refresher 1 – Research with Prisoners (ID: 939)	14-Oct-2015	2/2 (100%)
SBE Refresher 1 – Research in Educational Settings (ID: 940)	15-Oct-2015	2/2 (100%)
SBE Refresher 1 – International Research (ID: 15028)	15-Oct-2015	2/2 (100%)
SBE Refresher 1 – Defining Research with Human Subjects (ID: 15029)	14-Oct-2015	2/2 (100%)
SBE Refresher 1 – Assessing Risk (ID: 15034)	14-Oct-2015	2/2 (100%)
SBE Refresher 1 – Privacy and Confidentiality (ID: 15035)	15-Oct-2015	2/2 (100%)
SBE Refresher 1 – Research with Children (ID: 15036)	15-Oct-2015	2/2 (100%)
SBE Refresher 2 – Defining Research with Human Subjects (ID: 15038)	15-Oct-2015	1/1 (100%)
SBE Refresher 2 – Federal Regulations for Protecting Research Subjects (ID: 15040)	15-Oct-2015	1/1 (100%)
SBE Refresher 2 – Research in the Public Schools (ID: 15042)	15-Oct-2015	1/1 (100%)
SBE Refresher 2 – Research with Children (ID: 15043)	15-Oct-2015	1/1 (100%)
SBE Refresher 2 – International Research (ID: 15045)	15-Oct-2015	1/1 (100%)
Boston College (ID: 12098)	12-Oct-2018	No Quiz

For this Report to be valid, the learner identified above must have had a valid affiliation with the CITI Program subscribing institution identified above or have been a paid Independent Learner.

Verify at: www.citiprogram.org/verify/?k9f5e70eb-9578-484c-813f-3c38e5a8b8e4-28913180

Collaborative Institutional Training Initiative (CITI Program)
Email: support@citiprogram.org
Phone: 888-529-5929
Web: https://www.citiprogram.org

Boston College IRB
Approved
October 18, 2019
Through August 20, 2020

FOR APPROVALS WITH NO CR REQUIRED:

Boston College
IRB Approved
September 4, 2020-
September 3, 2021

Completion Date 27-Nov-2018
Expiration Date 26-Nov-2021
Record ID 29476947

This is to certify that:

Chokdee Rutirasiri

Has completed the following CITI Program course:

**Human Research**	(Curriculum Group)
**Social/Behavioral Research Course**	(Course Learner Group)
**1 - Basic Course**	(Stage)

Not valid for renewal of certification through CME. Do not use for TransCelerate mutual recognition (see Completion Report).

Under requirements set by:

Boston College

CITI

Collaborative Institutional Training Initiative

Boston College IRB Approved
October 18, 2019
Through August 20, 2020

Verify at www.citiprogram.org/verify/?wac3ebf44-4c2d-4460-a49f-104efdb7f855-29476947

FOR APPROVALS WITH NO CR REQUIRED:

Boston College IRB Approved
September 4, 2020-
September 3, 2021

Completion Date 27-May-2020
Expiration Date 27-May-2023
Record ID 36780811

This is to certify that:

Rebecca Esliker

Has completed the following CITI Program course:

Not valid for renewal of certification through CME. Do not use for TransCelerate mutual recognition (see Completion Report).

**Human Research**	(Curriculum Group)
**Social/Behavioral Research Course**	(Course Learner Group)
**1 - Basic Course**	(Stage)

Under requirements set by:

Boston College

CITI
Collaborative Institutional Training Initiative

Boston College IRB Approved
October 18, 2019
Through August 20, 2020

Verify at www.citiprogram.org/verify/?w106d9386-c40a-416a-8a29-75ab287ac422-36780811

FOR APPROVALS WITH NO CR REQUIRED:

Boston College IRB Approved
September 4, 2020-
September 3, 2021

CITI PROGRAM

Completion Date 25-Jan-2020
Expiration Date 24-Jan-2023
Record ID 35052714

This is to certify that:

Jordan Farrar

Has completed the following CITI Program course:

**Human Research**	(Curriculum Group)
**Social/Behavioral Research Course**	(Course Learner Group)
**2 - Refresher Course**	(Stage)

Under requirements set by:

Boston College

CITI
Collaborative Institutional Training Initiative

Verify at www.citiprogram.org/verify/?wd33ebf76-1c8d-4d87-b772-e709377ad55c-35052714

Boston College IRB
Approved
October 18, 2019
Through August 20, 2020

FOR APPROVALS WITH NO CR REQUIRED:

Boston College
IRB Approved
September 4, 2020-
September 3, 2021

Title: mHealth Tools to Improve Service Delivery Quality of an Evidence-Based Family Home Visiting Intervention to Prevent Family Violence among High Risk Families in Sierra Leone

Community Health Worker Consent (FSI-ECD)

My name is [RESEARCHER NAME], and I am a researcher. I work with a team at Boston College. The goal of our study is to support parents raising children between ages 6-36 months in Sierra Leone. Our program is called the Family Strengthening Intervention for Early Childhood Development (FSI-ECD). We also want to help improve supervision and training of community health workers by using mobile technology. We are asking you to be in this study because you are a community health worker. A research study is a way to learn more about something. This form explains the study.

This study is being done by Boston College and the University of Makeni. Our goal is to help families be stronger and solve challenges they face in healthy ways. The program will provides education on parenting skills, nutrition, children's health, and hygiene.The family strengthening intervention will last about 4 months. This program has 12 sessions that happen in the home once per week. Each session lasts about 90 minutes. You will be asked to deliver these sessions with families and to audio record each session. You will also be asked to participate in weekly, 60-minute supervision sessions with a supervisor in your community.

Before you can decide if you want to be in the study, I will explain everything to you. We have to tell you so that you can understand. If you have any questions, JUST ASK! You can decide not to be in the study. There will be no negative effects if you decide not to join. You can also decide to say yes now and change your mind later. It is okay if you want to quit after you already said yes. It is your choice. This will not change any help that you or your family get from the government or any other group.

If you decide that you want to be in our study, you will receive training on the family strengthening intervention. The training will happen five days a week for three weeks. You will also complete a one-day technology training so you can learn how to use the tablets and mobile tools. When the program starts, you will use the mobile tools to help you and your supervisor talk about each family strengthening intervention session. This will include completing an electronic checklist after each family strengthening intervention session. The checklist will help us know whether important program components were delivered in each session.

You will also do two short surveys. This will happen before the program starts and after it finishes. We will ask you questions about your satisfaction with the mobile tools and feelings about the program. These surveys will be at a private location in your community. The surveys will take 20-30 minutes to finish. **For each survey that you finish, you will receive a home gift (i.e., soap) equal to 18,000 Leones**.

You also **might** be asked to be in an interview. This will be random, like flipping a coin to decide who will be selected. This interview will be at your community health unit or another private location in your community. You will be asked about your satisfaction with the mobile tools. All conversations will be audio recorded and transcribed. We will do everything we can to protect your confidentiality and privacy in this research. For finishing the interview, **you will receive a home gift (i.e., soap) equal to 18,000 Leones.**

In the interview, the researcher will use a small computer that looks like a mobile phone to write down notes. We will keep our notes safe and keep them on a **password protected** computer in a locked

Boston College IRB Approved
October 18, 2019
Through August 20, 2020

FOR APPROVALS WITH NO CR REQUIRED:

Boston College
IRB Approved
September 4, 2020-
September 3, 2021

office. When we write about the interview, we will remove all information about you, like your name or where you live, so that your name and identity will be protected. The audio recordings will be kept in the same place in the same way. These are all ways that we will protect your confidentiality and privacy.

The major benefit of being in this study is to improve your skills in providing family support services. Your input about the mobile tools will also be used to improve them so others can benefit too.

There also may be some risks of participating in this study **related to loss of confidentiality and privacy. All researchers have strict rules that they follow to make sure your information is on a computer with a password in a locked office. There might also be unknown risks. We will do everything we can to make sure that no other risks happen. If they do happen, our researchers and social workers received training, and they can respond to problems. If you decide to be in the study, you do not have to do anything that you do not want to and you can stop at any time.**

Other people will not know if you are in our study. The information we write down about you and other adult caregivers will be private. When we tell other people or write an article about this research, we will not use your name. This way, no one will know that you were in the study.

Everything you tell is confidential, but one risk is that confidentiality cannot be guaranteed. All participant information is kept locked away and stored on password protected computers to best protect your confidentiality. We will not tell anyone about your answers unless you say that it is okay, or unless we think that you or someone else might be in danger. If you tell us that you or other people might get hurt, we cannot keep that information private. We will take action to protect everyone's safety. If you decide to join, you do not have to do anything that you do not want to. You can stop at any time. Our study social workers will be available if you feel upset. You do not have to pay for this service.

Mainly, only the researchers will have access to information. However, a few other key people may also have access to information. These might include government agencies. The Institutional Review Board at Boston College and internal Boston College auditors may review the research records. Otherwise, the researchers will not release any information to others that identifies you unless you give your permission, or unless we are legally required to do so.

We will use some of the information we collect for academic presentations and publications. We will only present general information and we will not focus on individuals. All data will be de-identified so it is not be possible to identify the people in the study. All data will be saved so that other researchers can use it for research too.

You do not have to be in this **research** study. It is okay if you decide not to be in the study or if you change your mind and want to stop at any time. No one will be upset with you. **You will not lose your status as a CHW in your community if you decide not to participate.**

My telephone number is . You can call me if you have questions about the study. I will contact Dr. Alethea Desrosiers of Boston College at +16096021466. She is the leader of the study. **If you have any questions about your rights as a participant, you can call Willietta Vincent, the Secretary of the Sierra Leone Ethics Committee at 033427383, if you have questions about your**

Boston College IRB Approved
October 18, 2019
Through August 20, 20[illegible]

FOR APPROVALS WITH NO CR REQUIRED:

Boston College
IRB Approved
September 4, 2020-
September 3, 2021

rights as a participant. The Boston College Office for Research Protections Institutional Review Board can also be contacted at +16175524478. If you decide to be in this study, I will ask if you agree and I will write down a note that you agreed.

Do you feel like you understand the things I just told you? Do you have any questions about anything I just told you, or about the research study?

AGREEMENT (Statement of Consent)

Do you feel like you asked all of your questions, and that I answered you?

Do you agree to be in this study?

Do you want a copy of this information sheet?

Signature of Research Assistant obtaining consent

INDICATE ORAL CONSENT ON LOG: ☐
(check box if participant gives oral consent)

☐ Check this box to acknowledge the participant has received a copy of the informed consent document

Boston College IRB Approved
October 18, 2019
Through August 20, 2020

FOR APPROVALS WITH NO CR REQUIRED:

Boston College IRB Approved
September 4, 2020-
September 3, 2021

Title: mHealth Tools to Improve Service Delivery Quality of an Evidence-Based Family Home Visiting Intervention to Prevent Family Violence among High Risk Families in Sierra Leone

CHW Consent (UI/IX)

My name is [RESEARCHER NAME], and I am a researcher. I work with a team at Boston College. The goal of our study is to support parents raising children between ages 6-36 months in Sierra Leone. Our program is called the Family Strengthening Intervention for Early Childhood Development (FSI-ECD). We also want to help improve supervision and training of community health workers by using mobile technology. We are asking you to be in this study because you are a community health worker. A research study is a way to learn more about something. This form explains the study.

This study is being done by Boston College and the University of Makeni. Our goal is to help families solve challenges they face in healthy ways. We also want to design and develop mobile tools that can help community health worker supervisors provide training and supervision to community health workers. The mobile tools could provide data to help supervisors provide feedback more quickly on community health worker performance.

Before you can decide if you want to be in the study, I will explain everything to you. We have to tell you so that you can understand. If you have any questions, JUST ASK! You can decide not to be in the study. There will be no negative effects if you decide not to join. You can also decide to say yes now and change your mind later. It is okay if you want to quit after you already said yes. It is your choice. This will not change any help that you or your family get from the government or any other group.

If you decide that you want to be in our study, you will participate in three **focus group discussion** sessions related to mobile tool development. Each session will last 60-90 minutes. In each **focus group discussion**, you will be asked to provide ideas about what you would want in a mobile tool to help with supervision. After we develop the tools, you will be asked to provide your thoughts about what you like and dislike about them. We will use your ideas to help make the mobile tools easier to use and more helpful for you. After the last session, you will do a short survey about using the mobile tools. The survey will take about 20 minutes.

Focus group discussions about mobile tool development will happen at the University of Makeni or the community health unit in your community. All conversations during the **focus group discussions** will be audio recorded and transcribed. We will do everything we can to protect your confidentiality and privacy in this research. For participating in each **focus group discussion**s, **you will receive a home gift (i.e., soap) equal to 18,000 Leones.**

When we write about the **focus group discussions**, we will remove all information about you, like your name or where you live, so that your name and identity will be protected. The audio recordings will be kept in the same place in the same way. These are all ways that we will protect your confidentiality and privacy.

The major benefit of participating in this study is to help develop tools that can make supervision and training of community health workers easier. This could also help you in your own work as a community health worker.

Boston College IRB Approved October 18, 2019 Through August 20, 2020

There also may be some risks of participating in this study. **related to loss of confidentiality and privacy. All researchers have strict rules that they follow to make sure your information is on a**

computer with a password in a locked office. There might also be unknown risks. We will do everything we can to make sure that no other risks happen. If they do happen, our researchers and social workers received training, and they can respond to problems. If you decide to be in the study, you do not have to do anything that you do not want to and you can stop at any time.

Other people will not know if you are in our study. The information we write down about you and other adult caregivers will be private. When we tell other people or write an article about this research, we will not use your name. This way, no one will know that you were in the study.

Everything you tell is confidential, but one risk is that confidentiality cannot be guaranteed. All participant information is kept locked away and stored on password protected computers to best protect your confidentiality. We will not tell anyone about your answers unless you say that it is okay, or unless we think that you or someone else might be in danger. If you tell us that you or other people might get hurt, we cannot keep that information private. We will take action to protect everyone's safety. If you decide to join, you do not have to do anything that you do not want to. You can stop at any time. Our study social workers will be available if you feel upset. You do not have to pay for this service.

Mainly, only the researchers will have access to information. However, a few other key people may also have access to information. These might include government agencies. The Institutional Review Board at Boston College and internal Boston College auditors may review the research records. Otherwise, the researchers will not release any information to others that identifies you unless you give your permission, or unless we are legally required to do so.

We will use some of the information we collect for academic presentations and publications. We will only present general information and we will not focus on individuals. All data will be de-identified so it is not be possible to identify the people in the study. All data will be saved so that other researchers can use it for research too.

You do not have to be in this study. It is okay if you decide not to be in the study or if you change your mind and want to stop at any time. No one will be upset with you. **You will not lose your status as a CHW in the community if you decide not to participate.**

My telephone number is   . You can call me if you have questions about the study. I will contact Dr. Alethea Desrosiers of Boston College at +16096021466. She is the leader of the study. **If you have any questions about your rights as a participant,** you can call Willietta Vincent, the Secretary of the Sierra Leone Ethics Committee at 033427383, if you have questions about your rights as a participant. **The Boston College Office for Research Protections Institutional Review Board can also be contacted at +16175524478.** If you decide to be in this study, I will ask if you agree and I will write down a note that you agreed.

Do you feel like you understand the things I just told you? Do you have any questions about anything I just told you, or about the research study?

Boston College IRB Approved
October 18, 2019
Through August 20, 2020

AGREEMENT (Statement of Consent)

FOR APPROVALS WITH NO CR REQUIRED:

Boston College IRB Approved
September 4, 2020-
September 3, 2021

Title: mHealth Tools to Improve Service Delivery Quality of an Evidence-Based Family Home Visiting Intervention to Prevent Family Violence among High Risk Families in Sierra Leone

Do you feel like you asked all of your questions, and that I answered you?

Do you agree to be in this study?

Do you want a copy of this information sheet?

Signature of Research Assistant obtaining consent

INDICATE ORAL CONSENT ON LOG: ☐
(check box if participant gives oral consent)

☐ Check this box to acknowledge the participant has received a copy of the informed consent document

Boston College IRB Approved
October 18, 2019
Through August 20, 2020

FOR APPROVALS WITH NO CR REQUIRED:

Boston College IRB Approved
September 4, 2020-
September 3, 2021

Title: mHealth Tools to Improve Service Delivery Quality of an Evidence-Based Family Home Visiting Intervention to Prevent Family Violence among High Risk Families in Sierra Leone

Community Health Worker Consent (FSI-ECD)

My name is [RESEARCHER NAME], and I am a researcher. I work with a team at Boston College. The goal of our study is to support **cohabitating caregivers** raising children between ages 6-36 months in Sierra Leone. Our program is called the Family Strengthening Intervention for Early Childhood Development (FSI-ECD). We also want to help improve supervision and training of community health workers by using mobile technology. We are asking you to be in this study because you are a community health worker. A research study is a way to learn more about something. This form explains the study.

This study is being done by Boston College and the University of Makeni. Our goal is to help families be stronger and solve challenges they face in healthy ways. The program will provides education on parenting skills, nutrition, children's health, and hygiene. The family strengthening intervention will last about 4 months. This program has 12 sessions that happen in the home once per week. Each session lasts about 90 minutes. You will be asked to deliver these sessions with families and to audio record each session. You will also be asked to participate in weekly, 60-minute supervision sessions with a supervisor in your community.

Before you can decide if you want to be in the study, I will explain everything to you. We have to tell you so that you can understand. If you have any questions, JUST ASK! You can decide not to be in the study. There will be no negative effects if you decide not to join. You can also decide to say yes now and change your mind later. It is okay if you want to quit after you already said yes. It is your choice. This will not change any help that you or your family get from the government or any other group.

If you decide that you want to be in our study, you will receive training on the family strengthening intervention. The training will happen five days a week for three weeks. You will also complete a one-day technology training so you can learn how to use the tablets and mobile tools. When the program starts, you will use the mobile tools to help you and your supervisor talk about each family strengthening intervention session. This will include completing an electronic checklist after each family strengthening intervention session. The checklist will help us know whether important program components were delivered in each session.

You will also do two short surveys. This will happen before the program starts and after it finishes. We will ask you questions about your satisfaction with the mobile tools and feelings about the program. These surveys will be at a private location in your community. The surveys will take 20-30 minutes to finish. **For each survey that you finish, you will receive a home gift (i.e., soap) equal to 18,000 Leones**.

You also **might** be asked to be in an interview. This will be random, like flipping a coin to decide who will be selected. This interview will be at your community health unit or another private location in your community. You will be asked about your satisfaction with the mobile tools. All conversations will be audio recorded and transcribed. We will do everything we can to protect your confidentiality and privacy in this research. For finishing the interview, **you will receive a home gift (i.e., soap) equal to 18,000 Leones.**

In the interview, the researcher will use a small computer that looks like a mobile phone to write down notes. We will keep our notes safe and keep them on a password protected computer in a locked office.

Boston College IRB Approved
October 18, 2019
Through August 20, 2020

FOR APPROVALS WITH NO CR REQUIRED:

Boston College IRB Approved
September 4, 2020-
September 3, 2021

When we write about the interview, we will remove all information about you, like your name or where you live, so that your name and identity will be protected. The audio recordings will be kept in the same place in the same way. These are all ways that we will protect your confidentiality and privacy.

The major benefit of being in this study is to improve your skills in providing family support services. Your input about the mobile tools will also be used to improve them so others can benefit too.

There also may be some risks of participating in this study related to loss of confidentiality and privacy. **All researchers have strict rules that they follow to make sure your information is on a computer with a password in a locked office. There might also be unknown risks. We will do everything we can to make sure that no other risks happen. If they do happen, our researchers and social workers received training, and they can respond to problems**. If you decide to be in the study, you do not have to do anything that you do not want to and you can stop at any time.

Besides the other focus group discussion participants, other people will not know if you are in our study. The information we write down about you will be private. When we tell others or write an article about this research, we will not use your name. This way, no one will know that you were in the study.

Everything you tell is confidential, but one risk is that confidentiality cannot be guaranteed. All participant information is kept locked away and stored on password protected computers to best protect your confidentiality. We will not tell anyone about your answers unless you say that it is okay, or unless we think that you or someone else might be in danger. If you tell us that you or other people might get hurt, we cannot keep that information private. We will take action to protect everyone's safety. If you decide to join, you do not have to do anything that you do not want to. You can stop at any time. Our study social workers will be available if you feel upset. You do not have to pay for this service.

Mainly, only the researchers will have access to information. However, a few other key people may also have access to information. These might include government agencies. The Institutional Review Board at Boston College and internal Boston College auditors may review the research records. Otherwise, the researchers will not release any information to others that identifies you unless you give your permission, or unless we are legally required to do so.

We will use some of the information we collect for academic presentations and publications. We will only present general information and we will not focus on individuals. All data will be de-identified so it is not be possible to identify the people in the study. All data will be saved so that other researchers can use it for research too.

You do not have to be in this research study. It is okay if you decide not to be in the study or if you change your mind and want to stop at any time. No one will be upset with you. You will not lose your **occupational** status as a CHW in your community if you decide not to participate.

My telephone number is   . You can call me if you have questions about the study. I will contact Dr. Alethea Desrosiers of Boston College at +16096021466. She is the leader of the study. If you have any questions about your rights as a participant, you can call Willietta Vincent, the Secretary of the Sierra Leone Ethics Committee at 033427383, if you have questions about your rights as a participant. The Boston College Office for Research Protections Institutional Review Board can also be

Boston College IRB Approved
October 18, 2019
Through August 20, 2020

FOR APPROVALS WITH NO CR REQUIRED:

Boston College
IRB Approved
September 4, 2020-
September 3, 2021

contacted at +16175524478. If you decide to be in this study, I will ask if you agree and I will write down a note that you agreed.

Do you feel like you understand the things I just told you? Do you have any questions about anything I just told you, or about the research study?

AGREEMENT (Statement of Consent)

Do you feel like you asked all of your questions, and that I answered you?

Do you agree to be in this study?

Do you want a copy of this information sheet?

Signature of Research Assistant obtaining consent

INDICATE ORAL CONSENT ON LOG: ☐
(check box if participant gives oral consent)

☐ Check this box to acknowledge the participant has received a copy of the informed consent document

Boston College IRB Approved
October 18, 2019
Through August 20, 2020

FOR APPROVALS WITH NO CR REQUIRED:

Boston College IRB Approved
September 4, 2020-
September 3, 2021

Title: mHealth Tools to Improve Service Delivery Quality of an Evidence-Based Family Home Visiting Intervention to Prevent Family Violence among High Risk Families in Sierra Leone

CHW Consent (UI/UX)

My name is [RESEARCHER NAME], and I am a researcher. I work with a team at Boston College. The goal of our study is to support **cohabitating caregivers** raising children between ages 6-36 months in Sierra Leone. Our program is called the Family Strengthening Intervention for Early Childhood Development (FSI-ECD). We also want to help improve supervision and training of community health workers by using mobile technology. We are asking you to be in this study because you are a community health worker. A research study is a way to learn more about something. This form explains the study.

This study is being done by Boston College and the University of Makeni. Our goal is to help families solve challenges they face in healthy ways. We also want to design and develop mobile tools that can help community health worker supervisors provide training and supervision to community health workers. The mobile tools could provide data to help supervisors provide feedback more quickly on community health worker performance.

Before you can decide if you want to be in the study, I will explain everything to you. We have to tell you so that you can understand. If you have any questions, JUST ASK! You can decide not to be in the study. There will be no negative effects if you decide not to join. You can also decide to say yes now and change your mind later. It is okay if you want to quit after you already said yes. It is your choice. This will not change any help that you or your family get from the government or any other group.

If you decide that you want to be in our study, you will participate in three focus group discussion sessions related to mobile tool development. Each session will last 60-90 minutes. In each focus group discussion, you will be asked to provide ideas about what you would want in a mobile tool to help with supervision. After we develop the tools, you will be asked to provide your thoughts about what you like and dislike about them. We will use your ideas to help make the mobile tools easier to use and more helpful for you. After the last session, you will do a short survey about using the mobile tools. The survey will take about 20 minutes.

Focus group discussions about mobile tool development will happen at the University of Makeni or the community health unit in your community. All conversations during the focus group discussions will be audio recorded and transcribed. **We cannot guarantee confidentiality because there will be other participants in the focus group discussions who could disclose information.** We will still do everything we can to protect your confidentiality and privacy in this research. For participating in each focus group discussions, **you will receive a home gift (i.e., soap) equal to 18,000 Leones.**

When we write about the focus group discussions, we will remove all information about you, like your name or where you live, so that your name and identity will be protected. The audio recordings will be kept in the same place in the same way. These are all ways that we will protect your confidentiality and privacy.

The major benefit of participating in this study is to help develop tools that can make supervision and training of community health workers easier. This could also help you in your own work as a community health worker.

Boston College IRB Approved
October 18, 2019
Through August 20, 2020

FOR APPROVALS WITH NO CR REQUIRED:

Boston College IRB Approved
September 4, 2020-
September 3, 2021

There also may be some risks of participating in this study. related to loss of confidentiality and privacy. **All researchers have strict rules that they follow to make sure your information is on a computer with a password in a locked office. There might also be unknown risks. We will do everything we can to make sure that no other risks happen. If they do happen, our researchers and social workers received training, and they can respond to problems**. If you decide to be in the study, you do not have to do anything that you do not want to and you can stop at any time.

Besides the other focus group discussion participants, other people will not know if you are in our study. The information we write down about you will be private. When we tell others or write an article about this study, we will not use your name. This way, no one will know that you were in the study.

Everything you tell is confidential, but one risk is that confidentiality cannot be guaranteed. All participant information is kept locked away and stored on password protected computers to best protect your confidentiality. We will not tell anyone about your answers unless you say that it is okay, or unless we think that you or someone else might be in danger. If you tell us that you or other people might get hurt, we cannot keep that information private. We will take action to protect everyone's safety. If you decide to join, you do not have to do anything that you do not want to. You can stop at any time. Our study social workers will be available if you feel upset. You do not have to pay for this service.

Mainly, only the researchers will have access to information. However, a few other key people may also have access to information. These might include government agencies. The Institutional Review Board at Boston College and internal Boston College auditors may review the research records. Otherwise, the researchers will not release any information to others that identifies you unless you give your permission, or unless we are legally required to do so.

We will use some of the information we collect for academic presentations and publications. We will only present general information and we will not focus on individuals. All data will be de-identified so it is not be possible to identify the people in the study. All data will be saved so that other researchers can use it for research too.

You do not have to be in this study. It is okay if you decide not to be in the study or if you change your mind and want to stop at any time. No one will be upset with you. You will not lose your **occupational** status as a CHW in the community if you decide not to participate.

My telephone number is   . You can call me if you have questions about the study. I will contact Dr. Alethea Desrosiers of Boston College at +16096021466. She is the leader of the study. You can also call Willietta Vincent, the Secretary of the Sierra Leone Ethics Committee at 033427383, if you have questions about your rights as a participant. If you decide to be in this study, I will ask if you agree and I will write down a note that you agreed.

Do you feel like you understand the things I just told you? Do you have any questions about anything I just told you, or about the research study?

Boston College IRB
Approved
October 18, 2019
Through August 20, 2020

AGREEMENT (Statement of Consent)

FOR APPROVALS WITH NO CR REQUIRED:

Boston College
IRB Approved
September 4, 2020-
September 3, 2021

Title: mHealth Tools to Improve Service Delivery Quality of an Evidence-Based Family Home Visiting Intervention to Prevent Family Violence among High Risk Families in Sierra Leone

Do you feel like you asked all of your questions, and that I answered you?

Do you agree to be in this study?

Do you want a copy of this information sheet?

Signature of Research Assistant obtaining consent

INDICATE ORAL CONSENT ON LOG: ☐
(check box if participant gives oral consent)

☐ Check this box to acknowledge the participant has received a copy of the informed consent document

Boston College IRB Approved
October 18, 2019
Through August 20, 2020

FOR APPROVALS WITH NO CR REQUIRED:

Boston College IRB Approved
September 4, 2020-
September 3, 2021

Title: mHealth Tools to Improve Service Delivery Quality of an Evidence-Based Family Home Visiting Intervention to Prevent Family Violence among High Risk Families in Sierra Leone

CHW Supervisor Consent

My name is [RESEARCHER NAME], and I am a researcher. I work with a team at Boston College. The goal of our study is to support **cohabitating caregivers** raising children between ages 6-36 months in Sierra Leone. Our program is called the Family Strengthening Intervention for Early Childhood Development (FSI-ECD). We also want to help improve supervision and training of community health workers by using mobile technology. We are asking you to be in this study because you are a community health worker supervisor. A research study is a way to learn more about something. This form explains the study.

This study is being done by Boston College and the University of Makeni. Our goal is to help families be stronger and solve challenges they face in healthy ways. The family strengthening intervention will last about 4 months. This program has 12 sessions that happen in the home once per week. Each session lasts about 90 minutes. Community health workers in your community will lead the sessions. You will be asked to provide weekly, 60-minute supervision sessions for these community health workers.

The Family Strengthening Intervention focuses on helping parents or caregivers to raise their children well. The program will provides education on parenting skills, nutrition, children's health, and hygiene.

Before you can decide if you want to be in the study, I will explain everything to you. We have to tell you so that you can understand. If you have any questions, JUST ASK! You can decide not to be in the study. There will be no negative effects if you decide not to join. You can also decide to say yes now and change your mind later. It is okay if you want to quit after you already said yes. It is your choice. This will not change any help that you or your family get from the government or any other group.

If you decide that you want to be in our study, you will receive training on the family strengthening intervention. The training will happen five days a week for three weeks. You will also complete a one-day technology training so you can learn how to use the tablets and mobile tools. When the program starts, you will supervise four community health workers who deliver the program to families in your community. You will use the mobile tools to help you provide feedback during supervision. This will include completing an electronic checklist after each family strengthening intervention session. The checklist will help us know whether important program components were delivered in each session.

You will also do two short surveys. This will happen before the program starts and after it finishes. We will ask you questions about your satisfaction with the mobile tools. These surveys will be at a private location in your community. The surveys will take 20-30 minutes to finish. **For each survey that you finish, you will receive a home gift (i.e., soap) equal to 18,000 Leones**.

You also **might** be asked to be in an interview. This will be random, like flipping a coin to decide who will be selected. This interview will be at your community health unit or another private location in your community. You will be asked about your satisfaction with the mobile tools. All conversations will be audio recorded and transcribed. We will do everything we can to protect your confidentiality and privacy in this research. For finishing the interview, **you will receive a home gift (i.e., soap) equal to 18,000 Leones.**

Boston College IRB Approved
October 18, 2019
Through August 20, 2020

FOR APPROVALS WITH NO CR REQUIRED:

Boston College IRB Approved
September 4, 2020-
September 3, 2021

In the interview, the researcher will use a small computer that looks like a mobile phone to write down notes. We will keep our notes safe and keep them on a computer with a password in a locked office. When we write about the interview, we will remove all information about you, like your name or where you live, so that your name and identity will be protected. The audio recordings will be kept in the same place in the same way. These are all ways that we will protect your confidentiality and privacy.

The major benefit of participating in this study is to improve your skills with supervision. Your input about the mobile tools will also be used to improve them so other supervisors might benefit too.

There also may be some risks of participating in this study related to loss of confidentiality and privacy. **All researchers have strict rules that they follow to make sure your information is on a computer with a password in a locked office. There might also be unknown risks. We will do everything we can to make sure that no other risks happen. If they do happen, our researchers and social workers received training, and they can respond to problems**. If you decide to be in the study, you do not have to do anything that you do not want to and you can stop at any time.

Besides the other focus group discussion participants, other people will not know if you are in our study. The information we write down about you will be private. When we tell others or write an article about this research, we will not use your name. This way, no one will know that you were in the study.

Everything you tell is confidential, but one risk is that confidentiality cannot be guaranteed. All participant information is kept locked away and stored on password protected computers to best protect your confidentiality. We will not tell anyone about your answers unless you say that it is okay, or unless we think that you or someone else might be in danger. If you tell us that you or other people might get hurt, we cannot keep that information private. We will take action to protect everyone's safety. If you decide to join, you do not have to do anything that you do not want to. You can stop at any time. Our study social workers will be available if you feel upset. You do not have to pay for this service.

Mainly, only the researchers will have access to information. However, a few other key people may also have access to information. These might include government agencies. The Institutional Review Board at Boston College and internal Boston College auditors may review the research records. Otherwise, the researchers will not release any information to others that identifies you unless you give your permission, or unless we are legally required to do so.

We will use some of the information we collect for academic presentations and publications. We will only present general information and we will not focus on individuals. All data will be de-identified so it is not be possible to identify the people in the study. All data will be saved so that other researchers can use it for research too.

You do not have to be in this study. It is okay if you decide not to be in the study or if you change your mind and want to stop at any time. No one will be upset with you. You will not lose your **occupational** status as a CHW supervisor in the community if you decide not to participate.

My telephone number is   . You can call me if you have questions about the study. I will contact Dr. Alethea Desrosiers of Boston College at +16096021466. She is the leader of the study. If you have any questions about your rights as a participant, you can call Willietta Vincent, the Secretary

Boston College IRB Approved
October 18, 2019
Through August 20, 2020

FOR APPROVALS WITH NO CR REQUIRED:

Boston College IRB Approved
September 4, 2020-
September 3, 2021

of the Sierra Leone Ethics Committee at 033427383, if you have questions about your rights as a participant. The Boston College Office for Research Protections Institutional Review Board can also be contacted at +16175524478. If you decide to be in this study, I will ask if you agree and I will write down a note that you agreed.

Do you feel like you understand the things I just told you? Do you have any questions about anything I just told you, or about the research study?

AGREEMENT (Statement of Consent)

Do you feel like you asked all of your questions, and that I answered you?

Do you agree to be in this study?

Do you want a copy of this information sheet?

Signature of Research Assistant obtaining consent

INDICATE ORAL CONSENT ON LOG: ☐
(check box if participant gives oral consent)

☐ Check this box to acknowledge the participant has received a copy of the informed consent document

Boston College IRB Approved
October 18, 2019
Through August 20, 2020

FOR APPROVALS WITH NO CR REQUIRED:

Boston College IRB Approved
September 4, 2020-
September 3, 2021

Title: mHealth Tools to Improve Service Delivery Quality of an Evidence-Based Family Home Visiting Intervention to Prevent Family Violence among High Risk Families in Sierra Leone

CHW Supervisor Consent (UI/UX)

My name is [RESEARCHER NAME], and I am a researcher. I work with a team at Boston College. The goal of our study is to support **cohabiting caregivers** raising children between ages 6-36 months in Sierra Leone. Our program is called the Family Strengthening Intervention for Early Childhood Development (FSI-ECD). We also want to help improve supervision and training of community health workers by using mobile technology. We are asking you to be in this study because you are a community health worker supervisor. A research study is a way to learn more about something. This form explains the study.

This study is being done by Boston College and the University of Makeni. Our goal is to help families solve challenges they face in healthy ways. We also want to design and develop mobile tools that can help community health worker supervisors provide training and supervision to community health workers. The mobile tools could provide data to help supervisors provide feedback more quickly on community health worker performance.

Before you can decide if you want to be in the study, I will explain everything to you. We have to tell you so that you can understand. If you have any questions, JUST ASK! You can decide not to be in the study. There will be no negative effects if you decide not to join. You can also decide to say yes now and change your mind later. It is okay if you want to quit after you already said yes. It is your choice. This will not change any help that you or your family get from the government or any other group.

If you decide that you want to be in our study, you will participate in three focus group discussion sessions related to mobile tool development. Each session will last 60-90 minutes. In each focus group discussion, you will be asked to provide ideas about what you would want in a mobile tool to help with supervision. After we develop the tools, you will be asked to provide your thoughts about what you like and dislike about them. We will use your ideas to help make the mobile tools easier to use and more helpful for you. After the last session, you will do a short survey about using the mobile tools. The survey will take about 20 minutes.

Focus group discussions about mobile tool development will happen at the University of Makeni or the community health unit in your community. All conversations during the focus group discussions will be audio recorded and transcribed. **We cannot guarantee confidentiality because there will be other participants in the focus group discussions who could disclose information.** We will still do everything we can to protect your confidentiality and privacy in this research. For participating in each focus group discussion, **you will receive a home gift (i.e., soap) equal to 18,000 Leones.**

When we write about the focus group discussions, we will remove all information about you, like your name or where you live, so that your name and identity will be protected. The audio recordings will be kept in the same place in the same way. These are all ways that we will protect your confidentiality and privacy.

The major benefit of participating in this study is to help develop tools that can make supervision and training of community health workers easier. This could help you in your work as a supervisor.

Boston College IRB Approved
October 18, 2019
Through August 20, 2020

FOR APPROVALS WITH NO CR REQUIRED:

Boston College IRB Approved
September 4, 2020-
September 3, 2021

There also may be some risks of participating in this study related to loss of confidentiality and privacy. **All researchers have strict rules that they follow to make sure your information is on a computer with a password in a locked office. There might also be unknown risks. We will do everything we can to make sure that no other risks happen. If they do happen, our researchers and social workers received training, and they can respond to problems**. If you decide to be in the study, you do not have to do anything that you do not want to and you can stop at any time.

Besides the other focus group discussion participants, other people will not know if you are in our study. The information we write down about you will be private. When we tell others or write an article about this research, we will not use your name. This way, no one will know that you were in the study.

Everything you tell is confidential, but one risk is that confidentiality cannot be guaranteed. All participant information is kept locked away and stored on password protected computers to best protect your confidentiality. We will not tell anyone about your answers unless you say that it is okay, or unless we think that you or someone else might be in danger. If you tell us that you or other people might get hurt, we cannot keep that information private. We will take action to protect everyone's safety. If you decide to join, you do not have to do anything that you do not want to. You can stop at any time. Our study social workers will be available if you feel upset. You do not have to pay for this service.

Mainly, only the researchers will have access to information. However, a few other key people may also have access to information. These might include government agencies. The Institutional Review Board at Boston College and internal Boston College auditors may review the research records. Otherwise, the researchers will not release any information to others that identifies you unless you give your permission, or unless we are legally required to do so.

We will use some of the information we collect for academic presentations and publications. We will only present general information and we will not focus on individuals. All data will be de-identified so it is not be possible to identify the people in the study. All data will be saved so that other researchers can use it for research too.

You do not have to be in this study. It is okay if you decide not to be in the study or if you change your mind and want to stop at any time. No one will be upset with you. You will not lose your **occupational** status as a CHW supervisor in the community if you decide not to participate.

My telephone number is . You can call me if you have questions about the study. I will contact Dr. Alethea Desrosiers of Boston College at +16096021466. She is the leader of the study. If you have any questions about your rights as a participant, you can call Willietta Vincent, the Secretary of the Sierra Leone Ethics Committee at 033427383, if you have questions about your rights as a participant. The Boston College Office for Research Protections Institutional Review Board can also be contacted at +16175524478. If you decide to be in this study, I will ask if you agree and I will write down a note that you agreed.

Do you feel like you understand the things I just told you? Do you have any questions about anything I just told you, or about the research study?

Boston College IRB Approved
October 18, 2019
Through August 20, 2020

FOR APPROVALS WITH NO CR REQUIRED:

Boston College IRB Approved
September 4, 2020-
September 3, 2021

Title: mHealth Tools to Improve Service Delivery Quality of an Evidence-Based Family Home Visiting Intervention to Prevent Family Violence among High Risk Families in Sierra Leone

AGREEMENT (Statement of Consent)

Do you feel like you asked all of your questions, and that I answered you?

Do you agree to be in this study?

Do you want a copy of this information sheet?

Signature of Research Assistant obtaining consent

INDICATE ORAL CONSENT ON LOG: ☐
(check box if participant gives oral consent)

☐ Check this box to acknowledge the participant has received a copy of the informed consent document

Boston College IRB Approved
October 18, 2019
Through August 20, 2020

FOR APPROVALS WITH NO CR REQUIRED:

Boston College IRB Approved
September 4, 2020-
September 3, 2021

Title: mHealth Tools to Improve Service Delivery Quality of an Evidence-Based Family Home Visiting Intervention to Prevent Family Violence among High Risk Families in Sierra Leone

Parent/Caregiver Participant Consent

My name is [RESEARCHER NAME], and I am a researcher. I work with a team at Boston College. The goal of our study is to support **cohabitating caregivers** raising children between ages 6-36 months in Sierra Leone. Our program is called the Family Strengthening Intervention for Early Childhood Development (FSI-ECD). We are asking you to be in this study because you have at least one child between the ages of 6-36 months. A research study is a way to learn more about something. This form explains the study.

This study is being done by Boston College and the University of Makeni. Our goal is to help families be stronger and solve challenges they face in healthy ways. In this research study, participants will be evenly placed in different groups. Assignment to either group is based on chance, like flipping a coin, not choice. This process is called randomization and allows researchers to compare results between two groups. One group receives the family strengthening intervention and one group receives the standard home visiting services provided by community health workers to families with young children. **As you might know, standard services include 3 home visits to support maternal and child health.**

If you are selected for the family strengthening intervention, you will receive 12 sessions in your home once per week. The program will last about 4 months. Each session lasts about 90 minutes. Community health workers in your community who are part of our study will lead the sessions. Sometimes these meetings will just be with you and other adults in the family, such as your spouse or partner. Sometimes the meetings will involve you and your child/children.

The Family Strengthening Intervention focuses on helping caregivers to raise their children well. Program activities will also focus on the major events your family has gone through, and how those events made family members feel. The program will provide some education on parenting skills, nutrition, children's health, and hygiene.

If you are selected for standard services, you will receive 3 home visiting sessions by a community health worker in your community. These sessions will provide information on things like nutrition, health, and hygiene for families with young children. Each session lasts about 60 minutes. These services will happen over 3-4 months.

Your participation in the study will last about 7 months. This includes 3-4 months of participation in **either** the family strengthening intervention **or standard services** and completion of a survey 3-months after the program **or standard services** are done.

Before you can decide if you want to be in the study, I will explain everything to you. We have to tell you so that you can understand. If you have any questions, JUST ASK! You can decide not to be in the study. There will be no negative effects if you decide not to join. You can also decide to say yes now and change your mind later. It is okay if you want to quit after you already said yes. It is your choice. This will not change any help that you or your family get from the government or any other group.

If you decide that you want to be in our study, you will do three surveys. This will happen before the **family strengthening intervention and standard services** start, after they finish, and then another 3-months later. We will ask you questions about how your family is doing, particularly your children, and

Boston College IRB Approved
October 18, 2019
Through August 20, 2020

FOR APPROVALS WITH NO CR REQUIRED:

Boston College
IRB Approved
September 4, 2020-
September 3, 2021

other aspects of life. We will ask you about problems you might have and how you feel. We will also ask you about your satisfaction with the program. These surveys will be at your home to protect your privacy and make it easier for you. The surveys will take 60-90 minutes to finish. **For each survey that you finish, you will receive a home gift (i.e., soap) equal to 30,000 Leones**. Before each survey, we will review the basic things that we discuss today and ask for your verbal consent to participate in the survey.

You also **might** be asked to be in an interview. This will be random, like flipping a coin to decide who will be selected. This interview will be in your home to protect your privacy. You will be asked about your satisfaction with the family strengthening intervention. All conversations will be audio recorded and transcribed. We will do everything we can to protect your confidentiality and privacy in this research. For finishing the interview, **you will receive a home gift (i.e., soap) equal to 18,000 Leones.**

In the interview, the researcher will use a small computer that looks like a mobile phone to write down notes. We will keep our notes safe, on a computer with a password in a locked office. When we write about the interview, we will remove all information about you, like your name or where you live, so that your name and identity will be protected. Audio recordings will also be kept in the same place in the same way. These are all ways that we will protect your confidentiality and privacy.

The major benefit of participating in this study is to get support to strengthen your family. Your input about the program will also be used to improve it so other families in Sierra Leone can benefit **from it**.

There also may be some risks of participating in this study. Some people may feel upset when talking about their thoughts and feelings, or about their problems. This might happen to you. If you decide to join, you and your child do not have to do anything that you do not want to. You can stop at any time. You do not have to respond to a survey item if it makes you feel upset. If you or your child feels upset after a survey or interview, you can talk to a social worker. You do not have to pay for this service.

There also may be risks of participating in this study related to loss of confidentiality and privacy. **All researchers have strict rules that they follow to make sure your information is on a computer with a password in a locked office. There might also be unknown risks. We will do everything we can to make sure that no other risks happen. If they do happen, our researchers and social workers received training, and they can respond to the problems**. If you decide to be in the study, you do not have to do anything that you do not want to and you can stop at any time.

Other people will not know if you are in our study. The information we write down about you and other adult caregivers will be private. When we tell other people or write an article about this research, we will not use your name. This way, no one will know that you were in the study.

Everything that you tell us in this study is confidential. We will not tell anyone about your answers unless you say that it is okay, or unless we think that you or someone else might be in danger. If you tell us that you or other people might get hurt, we cannot keep that information private, and we will take action to protect everyone's safety. Sometimes the research team finds out from a mother that her spouse/partner hits her or the child. If that happens, the research team has to tell the nearest Family Support Unit in the community. If we learn that you or your family need medical or social services, we can give you information privately.

Boston College IRB Approved
October 18, 2019
Through August 20, 2020

FOR APPROVALS WITH NO CR REQUIRED:

Boston College IRB Approved
September 4, 2020-
September 3, 2021

Mainly, only the researchers will have access to information. However, a few other key people may also have access to information. These might include government agencies. The Institutional Review Board at Boston College and internal Boston College auditors may review the research records. Otherwise, the researchers will not release any information to others that identifies you unless you give your permission, or unless we are legally required to do so.

We will use some of the information we collect for academic presentations and publications. We will only present general information and we will not focus on individuals. All data will be de-identified so it is not be possible to identify the people in the study. All data will be saved so that other researchers can use it for research too.

You do not have to be in this study. It is okay if you decide not to be in the study or if you change your mind and want to stop at any time. No one will be upset with you. If your spouse/partner decides to stop and you would like to continue, you can still be in the study. If you decide to stop the study, your spouse/partner can still participate. Sometimes spouses/partners disagree about whether to participate in the study. If this happens, you can take your time to think about it. You can contact me later if you decide you would like to be in the study.

My telephone number is   . You can call me if you have questions about the study. I will contact Dr. Alethea Desrosiers of Boston College at +16096021466. She is the leader of the study. If you have any questions about your rights as a participant, you can call Willietta Vincent, the Secretary of the Sierra Leone Ethics Committee at 033427383. The Boston College Office for Research Protections Institutional Review Board can also be contacted at +16175524478. If you decide to be in this study, I will ask if you agree and I will write down a note that you agreed.

Do you feel like you understand the things I just told you? Do you have any questions about anything I just told you, or about the research study?

AGREEMENT (Statement of Consent)

Do you feel like you asked all of your questions, and that I answered you?

Do you give permission for you and your child/children between ages 6 and 36 months to be in this study?

Do you want a copy of this information sheet?

Signature of Research Assistant obtaining consent

INDICATE ORAL CONSENT ON LOG: ☐
(check box if participant gives oral consent)

Boston College IRB Approved
October 18, 2019
Through August 20, 2020

FOR APPROVALS WITH NO CR REQUIRED:

Boston College IRB Approved
September 4, 2020-
September 3, 2021

Title: mHealth Tools to Improve Service Delivery Quality of an Evidence-Based Family Home Visiting Intervention to Prevent Family Violence among High Risk Families in Sierra Leone

☐ Check this box to acknowledge the participant has received a copy of the informed consent document

Boston College IRB Approved
October 18, 2019
Through August 20, 2020

FOR APPROVALS WITH NO CR REQUIRED:

Boston College IRB Approved
September 4, 2020-
September 3, 2021

Title: mHealth Tools to Improve Service Delivery Quality of an Evidence-Based Family Home Visiting Intervention to Prevent Family Violence among High Risk Families in Sierra Leone

CHW Supervisor Consent

My name is [RESEARCHER NAME], and I am a researcher. I work with a team at Boston College. The goal of our study is to support **cohabitating caregivers** raising children between ages 6-36 months in Sierra Leone. Our program is called the Family Strengthening Intervention for Early Childhood Development (FSI-ECD). We also want to help improve supervision and training of community health workers by using mobile technology. We are asking you to be in this study because you are a community health worker supervisor. A research study is a way to learn more about something. This form explains the study.

This study is being done by Boston College and the University of Makeni. Our goal is to help families be stronger and solve challenges they face in healthy ways. The family strengthening intervention will last about 4 months. This program has 12 sessions that happen in the home once per week. Each session lasts about 90 minutes. Community health workers in your community will lead the sessions. You will be asked to provide weekly, 60-minute supervision sessions for these community health workers.

The Family Strengthening Intervention focuses on helping caregivers to raise their children well. The program will provides education on parenting skills, nutrition, children's health, and hygiene.

Before you can decide if you want to be in the study, I will explain everything to you. We have to tell you so that you can understand. If you have any questions, JUST ASK! You can decide not to be in the study. There will be no negative effects if you decide not to join. You can also decide to say yes now and change your mind later. It is okay if you want to quit after you already said yes. It is your choice. This will not change any help that you or your family get from the government or any other group.

If you decide that you want to be in our study, you will receive training on the family strengthening intervention. The training will happen five days a week for three weeks. You will also complete a one-day technology training so you can learn how to use the tablets and mobile tools. When the program starts, you will supervise four community health workers who deliver the program to families in your community. You will use the mobile tools to help you provide feedback during supervision. This will include completing an electronic checklist after each family strengthening intervention session. The checklist will help us know whether important program components were delivered in each session.

You will also do two short surveys. This will happen before the program starts and after it finishes. We will ask you questions about your satisfaction with the mobile tools. These surveys will be at a private location in your community. The surveys will take 20-30 minutes to finish. **For each survey that you finish, you will receive a home gift (i.e., soap) equal to 18,000 Leones**.

You also **might** be asked to be in an interview. This will be random, like flipping a coin to decide who will be selected. This interview will be at your community health unit or another private location in your community. You will be asked about your satisfaction with the mobile tools. All conversations will be audio recorded and transcribed. We will do everything we can to protect your confidentiality and privacy in this research. For finishing the interview, **you will receive a home gift (i.e., soap) equal to 18,000 Leones.**

Boston College IRB Approved
October 18, 2019
Through August 20, 2020

FOR APPROVALS WITH NO CR REQUIRED:

Boston College IRB Approved
September 4, 2020-
September 3, 2021

In the interview, the researcher will use a small computer that looks like a mobile phone to write down notes. We will keep our notes safe and keep them on a computer with a password in a locked office. When we write about the interview, we will remove all information about you, like your name or where you live, so that your name and identity will be protected. The audio recordings will be kept in the same place in the same way. These are all ways that we will protect your confidentiality and privacy.

The major benefit of participating in this study is to improve your skills with supervision. Your input about the mobile tools will also be used to improve them so other supervisors might benefit too.

There also may be some risks of participating in this study related to loss of confidentiality and privacy. **All researchers have strict rules that they follow to make sure your information is on a computer with a password in a locked office. There might also be unknown risks. We will do everything we can to make sure that no other risks happen. If they do happen, our researchers and social workers received training, and they can respond to problems**. If you decide to be in the study, you do not have to do anything that you do not want to and you can stop at any time.

Besides the other focus group discussion participants, other people will not know if you are in our study. The information we write down about you will be private. When we tell others or write an article about this research, we will not use your name. This way, no one will know that you were in the study.

Everything you tell is confidential, but one risk is that confidentiality cannot be guaranteed. All participant information is kept locked away and stored on password protected computers to best protect your confidentiality. We will not tell anyone about your answers unless you say that it is okay, or unless we think that you or someone else might be in danger. If you tell us that you or other people might get hurt, we cannot keep that information private. We will take action to protect everyone's safety. If you decide to join, you do not have to do anything that you do not want to. You can stop at any time. Our study social workers will be available if you feel upset. You do not have to pay for this service.

Mainly, only the researchers will have access to information. However, a few other key people may also have access to information. These might include government agencies. The Institutional Review Board at Boston College and internal Boston College auditors may review the research records. Otherwise, the researchers will not release any information to others that identifies you unless you give your permission, or unless we are legally required to do so.

We will use some of the information we collect for academic presentations and publications. We will only present general information and we will not focus on individuals. All data will be de-identified so it is not be possible to identify the people in the study. All data will be saved so that other researchers can use it for research too.

You do not have to be in this study. It is okay if you decide not to be in the study or if you change your mind and want to stop at any time. No one will be upset with you. You will not lose your **occupational** status as a CHW supervisor in the community if you decide not to participate.

My telephone number is   . You can call me if you have questions about the study. I will contact Dr. Alethea Desrosiers of Boston College at +16096021466. She is the leader of the study. If you have any questions about your rights as a participant, you can call Willietta Vincent, the Secretary

Boston College IRB
Approved
October 18, 2019
Through August 20, 2020

FOR APPROVALS WITH NO CR REQUIRED:

Boston College
IRB Approved
September 4, 2020-
September 3, 2021

of the Sierra Leone Ethics Committee at 033427383, if you have questions about your rights as a participant. The Boston College Office for Research Protections Institutional Review Board can also be contacted at +16175524478. If you decide to be in this study, I will ask if you agree and I will write down a note that you agreed.

Do you feel like you understand the things I just told you? Do you have any questions about anything I just told you, or about the research study?

AGREEMENT (Statement of Consent)

Do you feel like you asked all of your questions, and that I answered you?

Do you agree to be in this study?

Do you want a copy of this information sheet?

Signature of Research Assistant obtaining consent

INDICATE ORAL CONSENT ON LOG: ☐
(check box if participant gives oral consent)

☐ Check this box to acknowledge the participant has received a copy of the informed consent document

Boston College IRB Approved
October 18, 2019
Through August 20, 2020

FOR APPROVALS WITH NO CR REQUIRED:

Boston College IRB Approved
September 4, 2020-
September 3, 2021

Recruitment Script
mHealth Supported FSI-ECD

Caregiver Participant Recruitment Script

Hello, my name is_______. I am working with the Boston College School of Social Work**, the University of Makeni** and Caritas Freetown. We are inviting parents with children aged 6-36 months who live in your community to participate in our research study. The goal of the study is to help parents raise their children well. Our program is called the Family Strengthening Intervention. The program has 12 sessions that would happen once per week in your home. Sessions last for 90 minutes and are provided by community health workers in your community. Sometimes, these meetings will just be with you and other adults in the family, such as your spouse or partner. Sometimes the meetings will involve you and your child/children. We will also ask you to complete a survey before and after the program, and then again 3-months later. The survey should take approximately 60-90 minutes to complete. Surveys will take place in your home. To thank you for your time, we will compensate you with a home gift **(e.g.,** soap equal**)** to 30,000 Leones for completing each survey. We are collecting this data only for study purposes, and the identification will be confidential. With your participation, we hope that the development of knowledge from this study may allow researchers and policy makers to create more effective programs for families with young children in Sierra Leone. If you decide you want to be in our study, I will ask you to participate in a short screening interview. We will ask you some questions about problems you might have, things you might feel, and things you do when you have problems. This interview will allow us to determine if you can be a part of this study or not. Participating in this screening interview does not mean that you will be enrolled in the program.

Boston College IRB Approved
October 18, 2019
Through August 20, 2020

FOR APPROVALS WITH NO CR REQUIRED:

Boston College IRB Approved
September 4, 2020-
September 3, 2021

CHW Recruitment Script

Hello, my name is_______. I am working with the Boston College School of Social Work**, the University of Makeni** and Caritas Freetown. We are inviting community health workers in your community to participate in our research study. The goal of the study is to help parents raise their children well. Our program is called the Family Strengthening Intervention. The program has 12 sessions that happen once per week in a family's home. Sessions last for about 90 minutes. You will be asked to deliver these sessions to families with children aged 6-36 months and to participate in weekly supervision with a supervisor in your community. You will also be asked to use mobile tools to prepare for supervision and monitor your performance. We will provide a three week training on the Family Strengthening Intervention and a one-day technology training. We will also ask you to complete a brief survey before and after the program. The survey should take approximately 20-30 minutes to complete. To thank you for your time, we will compensate you with a home gift **(e.g.,** soap**)** equal to 18,000 Leones for completing each survey. We are collecting this data only for study purposes, and the identification will be confidential. With your participation, we hope that the development of knowledge from this study may allow researchers and policy makers to create more effective programs for families with young children in Sierra Leone. We also hope to improve service delivery quality and supervision through developing mobile-based tools.

Boston College IRB Approved
October 18, 2019
Through August 20, 2020

FOR APPROVALS WITH NO CR REQUIRED:

Boston College IRB Approved
September 4, 2020-
September 3, 2021

Supervisor Recruitment Script

Hello, my name is_______. I am working with the Boston College School of Social Work, **the University of Makeni** and Caritas Freetown. We are inviting community health worker supervisors in your community to participate in our research study. The goal of the study is to help parents raise their children well. Our program is called the Family Strengthening Intervention. The program has 12 sessions that happen once per week in a family's home. Sessions last for about 90 minutes. You will be asked to provide weekly supervision to community health workers delivering these sessions to families with children aged 6-36 months. You will also be asked to use mobile tools to help you provide supervision and monitor the performance of community health workers. We will provide a three week training on the Family Strengthening Intervention and a one-day technology training. We will also ask you to complete a brief survey before and after the program. The survey should take approximately 20-30 minutes to complete. To thank you for your time, we will compensate you with a home gift **(e.g.,** soap**)** equal to 18,000 Leones for completing each survey. We are collecting this data only for study purposes, and the identification will be confidential. With your participation, we hope that the development of knowledge from this study may allow researchers and policy makers to create more effective programs for families with young children in Sierra Leone. We also hope to improve service delivery quality and supervision through developing mobile-based tools.

Boston College IRB Approved
October 18, 2019
Through August 20, 2020

FOR APPROVALS WITH NO CR REQUIRED:

Boston College IRB Approved
September 4, 2020-
September 3, 2021

UI/UX Participant Recruitment Script

Hello, my name is_______. I am working with the Boston College School of Social Work**, the University of Makeni** and Caritas Freetown. We are inviting community health workers and community health worker supervisors to participate in our research study. The ultimate goal of the study is to help parents raise their children well. Our program is called the Family Strengthening Intervention, and it will be delivered by community health workers to some families with children aged 6-36 months. We want to develop mobile tools to aid supervision and performance monitoring of community health workers. We would like community health workers and supervisors to provide their thoughts and ideas about the mobile tools. You will also be asked to participate in three discussion sessions about mobile tool development. Each discussion session will last 60-90 minutes. We will ask you to complete a brief survey about using the mobile tools. The survey will take approximately 20 minutes to complete. To thank you for your time, we will compensate you with a home gift **(e.g.,** soap**)** equal to 18,000 Leones for completing each survey. We are collecting this data only for study purposes, and the identification will be confidential. With your participation, we hope that the development of knowledge from this study may allow researchers and policy makers to create more effective programs for families with young children in Sierra Leone. We also hope to improve service delivery quality and supervision of community health workers through developing mobile-based tools.

Boston College IRB Approved
October 18, 2019
Through August 20, 2020

FOR APPROVALS WITH NO CR REQUIRED:

Boston College IRB Approved
September 4, 2020-
September 3, 2021

mHealth Tools to Improve Service Delivery Quality of an Evidence-Based Family Home Visiting Intervention
R21 Caregiver Assessment Battery

Table of Measures

**Section 1: Anthropometrics**	Demographic and Household Survey
**Section 2: Household Composition**	
**Section 3: Education**	
**Section 4: Parent-Child Interactions**	Observation of Mother-Child interactions (OMCI)
**Section 5: Home Environment**	Home Observation for Measurement of the Environment (HOME)
**Section 6: Health**	Demographic and Household Survey
**Section 7: Intimate Partner Relationships**	Revised Conflict Tactics Scale (CTS2)
**Section 8: Emotion Regulation**	Difficulties in Emotional Regulation (DERS)
**Section 9: Anxiety and Depression**	Hopkins Symptoms Checklist (HSCL)
**Section 10: Post-traumatic Stress**	PTSD Civilian Checklist
**Section 11: Functioning**	WHO Disability Assessment Schedule
**Section 12**	Closing

Boston College IRB Approved
October 18, 2019
Through August 20, 2020

FOR APPROVALS WITH NO CR REQUIRED:

Boston College IRB Approved
September 4, 2020-
September 3, 2021

SECTION 1: **Anthropometrics**

	Enter caregiver ID
	Enter child ID
	Enter enumerator ID
AN_1_C	Amos iya di pikin ole ?<br>*What is the child's age?*
AN_2_C	Us tem yu pikin bon?<br>*What is child's birthday?*
AN_3_C	*Calculate child's age in months.*
AN_4_C	Pik di dokument for sho di tem way di pikin bon.<br>*Select document used to verify child's birthdate.*
AN_5_C	Di pikin (pikin in nem) get pepa way sho di tem way e bon?<br>*Does (child's name) have a birth certificate?*
AN_6_C	Pik if na boy or gal pikin<br>*Select sex of child.*
AN_7_C	Duya put di fos leta for di pikin in fos nem.<br>*Enter first letter of child's name.*
AN_8_C	Duya put di fos leta for di pikin in las nem/sornem.<br>*Enter first letter of the child's last name/surname.*
LABEL	*Ask caregiver to undress child as much as possible.*
AN_9_C	*Was the child undressed to the minimum?*
AN_10_C	*How will the child's weight be assessed?*
AN_11_C	*Caregiver and the child together (kg)*
AN_12_C	*Caregiver alone (kg)*
AN_13_C	*Child's weight (kg)*
LABEL	*Age under 2 years: measure lengthy lying down;*<br>*Age 2 years or more: measure height of child standing*
AN_14_C	*Child's length (in cm)*
AN_15_C	*Child's height (in cm)*
AN_16_C	*Enter child's MUAC (cm)*

Boston College IRB Approved
October 18, 2019
Through August 20, 2020

FOR APPROVALS WITH NO CR REQUIRED:

Boston College IRB Approved
September 4, 2020-
September 3, 2021

SECTION 2: HOUSEHOLD COMPOSITION (HC_1_C- HC_15_C)

Label	Naw ar day kam aks yu som kweshon dem bot yu en di wan dem way una day it na di sam pot. Yu go tok bot den pipul dem ya eni aw yu go lek, wans yu abul tok bot alman.<br><br>*Now I am going to ask you several questions about EACH person who lives in your household. You can talk about these people in any order you would like, just as long as we talk about every person.*
HC_1_C	 *Enter caregiver sex*
HC_2_C	Ol in ol, amos pipul dem day liv na yu os way una day it na di sam pot?<br>*In total, how many people live in your household?*
HC_3_C	Ol in ol, amos pikin den day way na 17 iyas or nor rich, way den day liv na yu os?<br>*In total, how many children 17 years old or younger live in your household?*
HC_4_C	Amos bele bon pikin den yu get naw? (*Record number)*<br>*How many biological children do you have at present?*
HC_5_C	Amos pikin dem way nor to yu born day nar yu kiya naw naw?<br>*How many non-biological children are you presently the principal caretaker for?*
HC_6_C	Aw yu way day tek kia fambul to di pikin (pikin in nem)?<br>*What is your relationship to (child's name)?*
HC_7_C	 *What is your current marital status?*
HC_8_C	 *Does your partner currently live with you at least half of the time?*
HC_9_C	Apat from yu, oda posin day way day liv na di ose way day tek kia of dis pikin naw?<br>*Besides yourself, is there another primary caregiver for the child that lives in the home currently?*
HC_10_C	Di posin don liv na dis ose pas 15 days insai di las 30 days?<br>*Has he/she lived at home for more than 15 days in the last 30 days?*
HC_11_C	Aw di oda posin way day tek kia fambul to di pikin (pikin in nem)?<br>*What is the other caregiver's relationship to (child's name)?*
HC_12_C	Di posin way lan buk pas una ol na dis ose usai e tap pan buk lanin?<br>*What is the highest level of education completed by anyone in the household?*
LABEL	Den kweshon dem ya day aks yu for tok bot tin dem way yu en yu man/uman day du way una day wan plas wit una pikin (lek na rum, na motoka, or way una komot). Na for kont di tem den normor way una tri day wan ples (ar lek na for tu, tri awa normor for di wik). **Amos tem pa wik way una ol tri kin day togeda way una day...**<br><br>*These questions ask you to describe things you do when both you and your partner are physically present together with your child (i.e. in the same room, in the car, on outings). Count only times when all three of you are actually within the company of one another (even if this is just a few hours per week). How often in a typical week, when all 3 of you are together, do you:*
HC_13_C	Argu bot una marade ose biznes way nor konsan di pikin bifo di pikin?<br>*Argue about your relationship or marital issues unrelated to your child, in the child's presence?*
HC_14_C	Wan posin or una tu day tel in kompin bad wod bifo di pikin?<br>*One or both of you say cruel or hurtful things to each other in front of the child?*
HC_15_C	Aw di oda posin way day tek kia fambul to di pikin (pikin in nem)?<br>What is the other caregiver's relationship to (child's name)?

Boston College IRB Approved
October 18, 2019
Through August 20, 2020

FOR APPROVALS WITH NO CR REQUIRED:

Boston College IRB Approved
September 4, 2020-
September 3, 2021

SECTION 3: EDUCATION (ED_1_C – ED_3_C)

Label	Section 3: Naw ar go aks yu kweshon dem bot yu buk laning.<br><br>*Now I would like to ask you some questions about your education and literacy.*	
ED_1_C	Wus fom yu tap?<br>*What is the highest grade/form you completed?*	P1 <<01>><br>P2 <<02>><br>P3 <<03>><br>P4 <<04>><br>P5 <<05>><br>P6 <<06>><br>JSS1 <<07>><br>JSS2 <<08>><br>JSS3 <<09>><br>SS1 <<10>><br>SS2 <<11>><br>SS3 <<12>><br>Ar neva go skul <<00>><br>Ar nor no <<998>><br>Nor ansa <<999>>
ED_2_C	Aw wel yu ebul rid?<br>How well can you read?	Yu nor sabi rid <<01>><br>Yu sabi rid smul – <<02>><br>smul but I slo en tranga fur du am<br>Yu don sabi smul fur rid <<03>><br>Yu sabi en rid fayn-fayn wan<br><<04>><br>Ar nor no <<998>><br>Nor ansa <<999>>
ED_3_C	Aw wel yu ebul rite?<br>How well can you write?	Yu nor sabi rite <<01>><br>Yu sabi rite smal – <<02>><br>smal but e slo en tranga for du am<br>Yu don sabi smal for rite <<03>><br>Yu sabi en rite fayn-fayn wan<br><<04>><br>Ar nor no <<998>><br>Nor ansa <<999>>

Boston College IRB
Approved
October 18, 2019
Through August 20, 2020

FOR APPROVALS WITH NO CR REQUIRED:

Boston College
IRB Approved
September 4, 2020-
September 3, 2021

SECTION 4: CHILD BEHAVIOR (OMCI_1_C – OMCI_19_C)

RESPONSE OPTIONS
<<01>> Nor *No*
<<02>> Yes *Yes*

Label	KRIO TRANLSATION HERE<br><br>Instructions for Caregiver:<br>I want to watch you play and talk with {child_name} using a picture book, a ball or any kind of toy as you would do normally. You may choose one of these picture books. I would observe you for 5 minutes. When I say stop, you can discontinue.<br><br>Instructions for Interviewer:<br>Observe the mother and child for 5 minutes using your stop watches. Observe behaviours and mark on scale with a tick. Stop timing and coding after 5 minutes. Gently stop mother and child at a convenient point in their interaction. If the mother stops too early (< 4 min), ask her to continue, otherwise end it.<br><br>Neither at the start of the observation nor during the observation provides the mother with instructions or guidelines about how to interact with her child. Let mother continue as usual and make your observations without disturbing or guiding the interaction.<br><br>After checking the observations are all marked on the scale, calculate the total score achieved. The value of each rating must be added together based on the scoring instructions at the end of the observation.
Item Code	Kweshon<br>Question
OMCI_1_C	*Caregiver shows positive affect for the child.*
OMCI_2_C	*Caregiver shows negative affect for the child.*
OMCI_3_C	*Caregiver shows positive touch.*
OMCI_4_C	*Caregiver shows negative touch.*
OMCI_5_C	*Caregiver expresses positive verbal statements.*
OMCI_6_C	*Caregiver expresses negative verbal statements.*
OMCI_7_C	*Caregiver is sensitive of the child's needs.*
OMCI_8_C	*Caregiver expands on child's talking.*
OMCI_9_C	*Caregiver points and names the toy or object in the book.*
OMCI_10_C	*Caregiver questions child.*
OMCI_11_C	*Caregiver answers child's question or request.*

Boston College IRB Approved
October 18, 2019
Through August 20, 2020

FOR APPROVALS WITH NO CR REQUIRED:

Boston College IRB Approved
September 4, 2020-
September 3, 2021

OMCI_12_C	*Caregiver helps child to maintain interest.*

OMCI_13_C	*Child smiles, laughs at caregiver.*
OMCI_14_C	*Child shows excitement and enjoyment, like clapping.*
OMCI_15_C	*Child is crying, frowning, frustrated.*
OMCI_16_C	*Child remains focused on activity for a significant time (for at least 1 minute).*
OMCI_17_C	*Child continues in spite of distractions.*
OMCI_18_C	*Child is vocalizing/making sounds or producing words for the purpose of communicating*.
OMCI_19_C	*Caregiver & child express enjoyment while exploring together*.

Boston College IRB Approved
October 18, 2019
Through August 20, 2020

FOR APPROVALS WITH NO CR REQUIRED:

Boston College IRB Approved
September 4, 2020-
September 3, 2021

SECTION 5: HOME ENVIRONMENT (OMCI_1_C – OMCI_43_C)

RESPONSE OPTIONS		
<<01>>	Nor	*No*
<<02>>	Yes	*Yes*

Label	KRIO TRANLSATION HERE<br><br>Instructions for Interviewer:<br>The interview should be relaxed, non-judgmental and friendly. Talk to the mother while observing mother- child interactions. Observe items when you can; ask question for items that you cannot observe. Child should be in the same place doing his/her usual activities.
Item Code	Kweshon<br>Question
HOME_1_C	*Do you receive guests at home or visit family? Neighbors or friends who visit informally or drop-by and stay long enough to at least sit down or share a drink/conversation count here.*
HOME_2_C	*Does your child usually eat supper or any meal with the whole family? It's enough to just be sitting together at the same time, this does not mean you are all sharing the same food.*
HOME_3_C	*Does the father of the child spend some time every day caring for the child? For example talking, walking, and/or playing with the child?*
HOME_4_C	*When you are away, how many other people/caregivers are available to look after the child?*
HOME_5_C	*Does your child have regular playmates that are around his/her same age? By "around" we mean within 1 year older or younger.*
HOME_6_C	*How often does your child go to the market with you or another member of the household?*
HOME_7_C	*How often does your child go out of the house/yard?*
HOME_8_C	*How often does a caregiver take your child far from home?*
HOME_9_C	*When you are busy with housework, do you try to engage your child in what you are doing (e.g., talking with him/her about what you are doing or asking questions about what you are doing)?*
HOME_10_C	*Did you show or teach your child something new this past week, like teach a new word, or help child do something difficult?*
LABEL	*Please show me what your child typically plays with on his/her own.*
HOME_11_C	*Gross motor objects available (e.g., ball, rope, ring, stone).*
HOME_12_C	*Push or pull toys available (e.g., pull with string, push box).*
HOME_13_C	*Cuddly toys available?*
HOME_14_C	*Simple eye-hand coordination materials available (e.g. single object that rattles, shakes, squeezes).*

Boston College IRB Approved
October 18, 2019
Through August 20, 2020

FOR APPROVALS WITH NO CR REQUIRED:

Boston College
IRB Approved
September 4, 2020-
September 3, 2021

HOME_15_C	*Complex eye-hand coordination materials available (e.g., different shaped blocks, pencil & paper).*
HOME_16_C	*Book or item that has both pictures and words together (not textbook, can be collection of pictures).*
HOME_17_C	*At least 1 adult book or magazine is observed in the home (includes religious books but not textbooks).*
HOME_18_C	*Do you provide materials or activities that are slightly difficult for your child?*
HOME_19_C	*Does your child have access to children's books?*
HOME_20_C	*Are there any objects that the child plays with to create a story, play pretend, or imitate actions he/she sees in the home/community?*
HOME_21_C	*Does your child play any structured games with people (e.g., circle games, clapping/singing games)?*
HOME_22_C	*Does your child know where to find his/her playthings (e.g, bag, box)?*
HOME_23_C	*Have you ever found/made/ gotten something new for your child to play with?*
HOME_23a_C	*When did you get the newest toy/object?*
HOME_24_C	*In the past week, did you look or someone in the household look at pictures in a book, calendar or magazine with the child?*
HOME_25_C	*How often do you tell stories or sing songs with your child?*
LABEL	*PROMPT: At a natural point during the session, offer some praise of the child (e.g., "You did that so well!", "She can run so fast!", etc.). Make certain that your praise is genuine - do not try to force praise. Score this item "Yes" if the mother expresses happiness, pride, agreement, gratitude, etc. in response, either verbally or with facial expressions. However, if the mother says little more than "Thank you" without feeling or with an embarrassed smile, praise the child again later to see if he/she responds with more emotion. If you consistently get a response that shows little feeling or pleasure, score this item "No".*
HOME_26_C	*After visitor praises child, mother responds positively (e.g. mother nods, smiles, thanks, agrees).*
HOME_27_C	*The mother spontaneously talks to the child.*
HOME_28_C	*The mother responds verbally to child's talk or gestures (does not ignore the child; do not include scolding or shouting).*
HOME_29_C	*Mother tells child the name of an object or person during visit.*
HOME_30_C	*Mother's speech is distinct, clear, and audible.*
HOME_31_C	*Mother talks well and freely to the interviewer.*
HOME_32_C	*Mother permits child to play freely (includes messy, noisy play).*

Boston College IRB Approved
October 18, 2019
Through August 20, 2020

FOR APPROVALS WITH NO CR REQUIRED:

Boston College IRB Approved
September 4, 2020-
September 3, 2021

HOME_33a_C	*Mother spontaneously praises child verbally without prompt at least twice. FIRST INSTANCE*
HOME_33b_C	*Mother spontaneously praises child verbally without prompt at least twice. SECOND INSTANCE*
HOME_34_C	*Mother conveys positive feelings towards child. For example: smiling, tone of voice, or using positive words*
HOME_35_C	*Mother caresses, strokes head, or kisses the child.*<br><br>*Score any hug, kiss or cuddle as "Yes". Watch closely for subtle caresses or other affectionate gestures, such as holding a hand, stroking the face or hair (not fixing/cleaning it), patting the child gently, etc. Score either obvious or subtle caresses as "Yes". Simply touching a child does not necessarily count as a caress if it is not affectionate. (No <0>, = 0 times Yes <1> = 1st time)*
HOME_36_C	*Mother makes effort to provide child with a play object during visit*<br><br>*Score this item "Yes" if the mother offers the child something to play with and examine, whether it is a toy or any household object that the child seems interested in. The child's reaction (accepting or rejecting the object) does not affect the score. (No <0>, = 0 times Yes <1> = 1st time)*
HOME_37_C	*Mother shouts at child during the visit (except for corrective shouting to stop a child from doing something dangerous).*
HOME_38_C	*Mother complains about child or says child is bad.*
HOME_39_C	*Mother hits, pushes, shakes child during visit.*
HOME_40_C	*Mother threatens the child with punishment during visit.*<br><br>*Shyiraho ''Yego ''niba Mama w'umwana amuhutaza .Niba ubona bitagaragara ko umubyeyi ari gukina n'umwana cg ari kubabaza umwana ,reba igisubizo cy'umwana .Ni ba umwana asubizanije umunezero cg ibyishimo ,ibi akenshi bigaragazo uburyo bwo ''gukirana '' mu mimikino hagati ye na mama we . Niba umwana agaragaye nk'utishimye ,atongana,ahakana,cg arira ,uko biri kose shyira ho '' Yego ''*
HOME_41a_C	*Mother restricts or interferes with child's activity during visit three or more times. FIRST INSTANCE*
HOME_41b_C	*Mother restricts or interferes with child's activity during visit three or more times. SECOND INSTANCE*
HOEM_41c_C	*Mother restricts or interferes with child's activity during visit three or more times. THIRD INSTANCE*
HOME_42_C	*Observe whether indoor/outdoor area is safe for play.*
HOME_43_C	*Mother keeps child within eyesight, looks at him/her often.*

Boston College IRB Approved
October 18, 2019
Through August 20, 2020

FOR APPROVALS WITH NO CR REQUIRED:

SECTION 6: HEALTH (HE_1_C – HE_4_C)

Boston College IRB Approved
September 4, 2020-
September 3, 2021

Label	Section 4: Naw A go lek for aks yu som question bot yu welbodi en aw yu day du. Duya memba se no rait or rong ansa nor day. Jes gee day ansa way yu fil se e rait.<br><br>*Now I would like to ask you some questions about your health and general well-being. Please remember that there are no right or wrong answers. Just give the answer that fits best for you.*	
Item Code	Questions	Coding Responses
HE_1_C	Way yu kompia yu sef to oda posin way na yu ag grup yu go say yu welbodi na?<br>*Compared to another person of your age, would you say your health is:*	Bad-off pas oda pipul <<01>><br>*Worse than others*<br>Dae same lek oda pipul <<02>><br>*Same as others*<br>Bette pas oda pipul <<03>><br>*Better than the others*<br>Ar nor no <<998>><br>Nor ansa <<999>>
HE_2_C	Omos dez insai di pas mont way yu nor ebul du di wok dem way yu blant du bikoz yu mind or yu at nor bin rest?<br>*How often in the past one month were you unable to do the work you usually do because of an emotional or psychological problem?*	Gee di exact <<00>> ➔HE_2A_C<br>Ar nor no <<998>>➔HE_3_C<br>Nor ansa <<999>>➔HE_3_C
	HE_2A_C — Gee di exact ansa:<br>*Write exact answer:*<br>➔ ____________________	
HE_3_C	Omos dez insai di pas mont way yu ledon na bed pas haf day bikoz yu mind or yu at nor bin rest?<br>*How often in the past one month did you have to lie in bed for more than half a day because because of an emotional or psychological problem?*	Gee di exact <<00>> ➔HE_3A_C<br>Ar nor no <<998>>➔HE_4_C<br>Nor ansa <<999>>➔HE_4_C
	HE_3A_C — Gee di exact ansa:<br>➔ ____________________	
HE_4_C	Insai di pas mont, omos hard time yu bin get for du yu os wok bikoz yu mind or yu at nor bin rest?<br>*During the past month, how much difficulty did you have in taking care of your household responsibilities because of an emotional or psychological problem?*	Natin-natin <<00>><br>*Not at all*<br>Smal <<01>><br>*A little*<br>Small nor mor <<02>><br>Quite a bit<br>Borku borku wan <<03>><br>Extremely<br>Ar nor no <<998>><br>Nor ansa <<999>>

Boston College IRB Approved
October 18, 2019
Through August 20, 2020

FOR APPROVALS WITH NO CR REQUIRED:

Boston College IRB Approved
September 4, 2020-
September 3, 2021

SECTION 7: INTIMATE PARTNER RELATIONSHIPS (CTS_1_C-CTS_27_C)

Label	Section 5: Naw ar go ask yu som kweshon dem bot di way way yu day wit yu patna or boyfrien/girlfrien/wef/man.<br><br>*Now I will ask you some questions about your relationships with your partner. (boyfriend/girlfriend/husband/wife).*	
Item Code	Questions	Coding Responses
CTS_1_C	Yu bin don eva married or bin don day wit eni patna/man/uman?<br>*Have you ever been married or had a partner?*	No <<00>> → Section 6<br>Yes <<01>> → CTS_2_C<br>Ar nor nor <<998>> → Section 6<br>Nor ansa <<999>> → Section 6
CTS_2_C	Yu married or yudaywit eni patna/man/uman naw naw?<br>*Are you currently married or have a partner?*	No <<00>> → CTS_4_C<br>Yes <<01>> → CTS_3_C<br>Ar nor nor <<998>> → Section 6<br>Nor ansa <<999>> → Section 6
CTS_3_C	Yu day liv wit yu patna/man/wef naw naw?<br>*Do you live with your partner now?*	No <<00>> → Section 6<br>Yes <<01>> → CTS_5_C<br>Ar nor nor <<998>> → Section 6<br>Nor ansa <<999>> → Section 6
CTS_4_C	*Have you been married or had a partner in the last three months?*	No <<00>> → Section 6<br>Yes <<01>> → CTS_5_C<br>Ar nor nor <<998>> → Section 6<br>Nor ansa <<999>> → Section 6

RESPONSE OPTIONS

<<00>>	Dis nor wan day apin	*This has never happened*
<<01>>	Wan tem insai di pas ia	*Once in the past three months*
<<02>>	Tu tem insai di pas ia	*Twice in the past three months*
<<03>>	Tri or feiv tem insai di pas ia	*3 to 5 times in the past three months*
<<04>>	Six or ten tem insai di pas ia	*6 to 10 times in the past three months*
<<05>>	Elevin or twenty tem insai di pas ia	*11-20 times in the past three months*
<<06>>	Pas twenty tem insai di pas ia	*More than 20 times in the past three months*
<<07>>	Nor bi insai di pas iya, bot e bin apin bifo	*Not in the three months, but it did happen before*
<<998>>	Ar nor nor	*Don't know*
<<999>>	No ansa	*No answer*

Boston College IRB Approved
October 18, 2019
Through August 20, 2020

FOR APPROVALS WITH NO CR REQUIRED:

Boston College IRB Approved
September 4, 2020-
September 3, 2021

Label	E nor mata aw fayn, way marade man en uman dem day, tem go kam way den nor go gri, vex pan oda posin, den kin want difren tin dem from dem sef, mek palava or dem jos fet bikos den nor gladi, den taya or for som oda risin den. Marade man en uman den kin get boku way dem for tri en setul den problem dem. Dis na di lis of tin-dem way som tem go apin way den get palava. Duya sho omos tem yu bin du wan pan den tin dem yah insai di pas ia, en omos tem yu patna bin du dem insai di pas ia. Ef yu or yu partna nor bin du wan pan den tin dem ya insai di pas iya, bot e apin bifo dat, tok am.<br><br>*No matter how well a couple gets along, there are times when they disagree, get annoyed with the other person, want different things from each other, or just have spats or fights because they are in a bad mood, are tired, or for some other reason. Couple also have many different ways of trying to settle their differences. This is a list of things that might happen when you have differences. Please indicate how many times you did each of these things in the past year, and how many times your partner did them in the past year. If you or your partner did not do one of these things in the past year, but it happened before that, say this.*<br><br>Yu partna way yu day wit naw or di wan way yu bin day wit dis beyen tem, eni tin bin don day way una nor kam to wan pan way mek yu du den tin ya: DO NOT READ "NO ANSA", "AR NO NO", OR "NOT APPLICABLE"<br><br>*With your current or most recent partner were there any circumstances or household disagreements that caused you to do the following things:*
CTS_5_C	Yu eva kos yu patna/man/uman?<br>*Used abusive language at your partner.*
CTS_6_C	Yu eva lef yu patna/man/uman?<br>*Abandon/forsake your partner*
CTS_7_C	Yu eva push/shub yu patna/man/uman?<br>*Pushed or shoved your partner*
CTS_8_C	Yu eva bokul yu patna/man/uman?<br>*Grabbed your partner*
CTS_9_C	Yu eva slap yu patna/man/uman wit yu an?<br>*Slapped your partner with hand*
CTS_10_C	Yu eva yus nef or oda wepon pan yu patna/man/uman?<br>*Used a knife or other weapon on your partner*
CTS_11_C	Yu eva nak yu patna/man/uman wit eni tin way go damaj am?<br>*Hit your partner with an object that could hurt him/her*
CTS 12_C	Yu eva jam yu patna/man/uman pan di wol?<br>*Slammed your partner against a wall*
CTS_13_C	Yu eva kik yu patna/man/uman?<br>*Kicked your partner*
CTS_14_C	Yu eva yus fors pan yu patna/man/uman (lek for nak, bokul, or yus wepon) for du mami en dadi biznes?<br>*Used force (like hitting, holding down, or using a weapon) to make your partner have sex even though they didn't want to*
CTS_15_C	Yu partna way yu day wit naw or di wan way yu bin day wit, amos tem una kin mek palava insai di mont?<br>*In your relationship with your (current or most recent) partner, how often would you say that you quarrel in an average month?*
	Now I am going to ask some questions about things your current or most recent partner may have done to you.<br>Yu partna way yu day wit naw, or di wan way yu bin day wit dis beyen tem, bin don eva du eni wan pan den tin ya:<br>*Has your current or most recent partner ever:*
CTS_16_C	Yu partna eva kin kos yu?<br>*Used abusive language*
CTS_17_C	E eva lef yu?<br>*Abandoned/forsaken you*
CTS_18_C	E eva push yu?<br>*Pushed or shoved you*
CTS_19_C	E eva bokul yu?<br>*Grabbed you*
CTS_20_C	E eva slap or pata yu?<br>*Slapped you or hit you*
CTS_21_C	E eva us nef or oda wepon pan yu?<br>*Used a knife or other weapon on you*

Boston College IRB Approved
October 18, 2019
Through August 20, 2020

FOR APPROVALS WITH NO CR REQUIRED:

Boston College
IRB Approved
September 4, 2020-
September 3, 2021

CTS_22_C	E eva nak yu wit eni tin way go damaj yu?<br>*Hit you with an object that could hurt*
CTS_23_C	E eva jam yu pan di wol?<br>*Slammed you against a wall*
CTS_24_C	E eva kik yu?<br>*Kicked you*
CTS_25_C	E eva fet yu bone to bone for mek una du mami en dadi biznes way yu nor want?<br>*Physically forced you to have sexual intercourse when you did not want to?*
CTS_26_C	Yu bin don eva du mami en dadi biznes bikoz yu day fraid say if yu nor gri e go du bad to yu?<br>*Did you ever have sexual intercourse you did not want because you were afraid of what he/she might do?*
CTS_27_C	E bin don eva fos yu for du sometin lek mami en dadi biznes way shameful to yu?<br>*Did he ever force you to do something sexual that you found degrading or humiliating?*

Boston College IRB Approved
October 18, 2019
Through August 20, 2020

FOR APPROVALS WITH NO CR REQUIRED:

Boston College IRB Approved
September 4, 2020-
September 3, 2021

SECTION 8: EMOTION REGULATION (ER_1_C – ER_36_C)

RESPONSE OPTIONS		
<<01>>	E nor kin apin so normor	Almost never
<<02>>	Somtem dem	Sometimes
<<03>>	Somtem normor	About half the time
<<04>>	Boku tem dem	Most of the time
<<05>>	Almos altem	Almost always
<<998>>	Ar nor no	*Don't know*
<<999>>	Nor ansa	*No answer*

Label	Section 10: Ar day kam rid list bot aw youg pipul kin somtem fil or ansa to satin situashone. Tink bot each wod dem fayn fayn wan en konsida if di wod dem kin bi to yu, altem, borku tem, smol tem, somtem or wan day. Memba say no rite or rong ansa nor day.<br><br>*I'm going to read a list of statements about how young people may sometimes feel or react to certain situations. Think about each statement carefully and consider whether the statement applies to you almost always, most of the time, about half the time, sometimes, or almost never. Remember, there are no right or wrong answers.*
Item Code	Kweshon<br>Question
ER_1_C	Ar no sabi bot mi filin dem.<br>*I am not clear about my feelings.*
ER_2_C	Ar no day pay attenshon bot aw ar kin fil.<br>*I do not pay attention to how I feel.*
ER_3_C	Ar kin eksperens say mi filin dem pasmak en ar nor day abul kontrol dem.<br>*I experience my emotions as overwhelming and out of control.*
ER_4_C	Ar nor get no idia aw ar day fil.<br>*I have no idea how I am feeling*
ER_5_C	E kin tranga for ondastand bot mi filin dem.<br>*I have difficulty making sense out of my feelings.*
ER_6_C	Ar no kin abdul du somtin bot mi filin dem.<br>*I do not address my feelings.*
ER_7_C	Ar nor no gben aw ar day fil.<br>*I do not know exactly how I am feeling*
ER_8_C	Ar nor bisin bot waytin ar day fil.<br>*I do not care about what I am feeling.*
ER_9_C	Ar torment bot aw ar day fil.<br>*I am confused about how I feel.*
ER_10_C	Way ar fil bad, ar nor kin no bot filin dem.<br>*When I'm upset, I do not acknowledge my emotions.*
ER_11_C	Way ar fil bad, ar kin vex pa mi sef for way ar day fil da way day.<br>*When I'm upset, I become angry with myself for feeling that way (upset).*
ER_12_C	Way ar fil bad, ar kin shem for way ar kin fil da way.<br>*When I'm upset, I become embarrassed for feeling that way (upset).*
ER_13_C	Way ar fil bad, e kin tranga for du wok.<br>*When I'm upset, I have difficulty getting work done.*
ER_14_C	Way ar fil bad, ar day go awt of kontrol.<br>*When I'm upset, I become out of control.*
ER_15_C	Way ar fil bad, ar beliv say ar go day pa am for long tem.<br>*When I'm upset, I believe that I will remain that way for a long time.*
ER_16_C	Way ar fil bad, ar beliv say ar kin end up mi filin dem wit poil at.<br>*When I'm upset, I believe that I will end up feeling very depressed.*

Boston College IRB
Approved
October 18, 2019
Through August 20, 2020

FOR APPROVALS WITH NO CR REQUIRED:

Boston College
IRB Approved
September 4, 2020-
September 3, 2021

ER_17_C	Way ar fil bad, ar beliv say mi filin dem nor value en impotant.<br>*When I'm upset, I believe that my feelings are not valid and important.*
ER_18_C	Way ar fil bad, e kin at for mek ar put mi maynd pa oda tin dem.<br>*When I'm upset, I have difficulty focusing on other things.*
ER_19_C	Way ar fil bad, ar day los kontrol.<br>*When I'm upset, I feel out of control.*
ER_20_C	Way ar fil bad, ar nor day abul don for du somtin.<br>*When I'm upset, I cannot get things done.*
ER_21_C	Way ar fil bad, ar day fil shem pan mi sef for way ar day fil da way day.<br>*When I'm upset, I feel ashamed with myself for feeling that way.*
ER_22_C	Way ar fil bad, ar nor beliv say ar go fen way smol smol for mek ar fil beteh.<br>*When I'm upset, I don't believe that I can find a way to eventually feel better.*
ER_23_C	Way ar fil bad, ar kin fil lek ar wik.<br>*When I'm upset, I feel like I am weak.*
ER_24_C	Way ar fil bad, ar nor kin fil lek ar eabul kontrol di way aw ar day bihav.<br>*When I'm upset, I do not feel like I can remain in control of my behaviors.*
ER_25_C	Way ar fil bad, ar day fil gilti for di way ar day fil.<br>*When I'm upset, I feel guilty for feeling that way.*
ER_26_C	Way ar fil bad, e kin tranga for mek ar put mi maynd pan somtin.<br>*When I'm upset, I have difficulty concentrating.*
ER_27_C	Way ar fil bad, e kin tranga for mek ar kontrol di way ar day bihav.<br>*When I'm upset, I have difficulty controlling my behaviors.*
ER_28_C	Way ar fil bad, ar biliv say natin nor day way ar go du for misef way ar go fil beteh.<br>*When I'm upset, I believe there is nothing I can do to make myself feel better.*
ER_29_C	Way ar fil bad, ar day vex pan mi sef for way ar day fil da way day.<br>*When I'm upset, I become irritated with myself for feeling that way.*
ER_30_C	Way ar fil bad, ar kin bigin fil bad bot mi sef.<br>*When I'm upset, I start to feel very bad about myself.*
ER_31_C	Way ar fil bad, ar day tink bot am ol di tem.<br>*When I'm upset, I believe that wallowing in it is all I can do.*
ER_32_C	Way ar fil bad, ar day los kontrol of aw ar day bihav.<br>*When I'm upset, I lose control over my behaviors.*
ER_33_C	Way ar fil bad, e kin tranga for mek ar tink bot eni oda tin.<br>*When I'm upset, I have difficulty thinking about anything else.*
ER_34_C	Way ar fil bad, ar nor kin tek tem for no waytin ar day rili fil.<br>*When I'm upset, I do not take time to figure out what I'm really feeling.*
ER_35_C	Way ar fil bad, e kin tek long tem for mek ar fil beteh.<br>*When I'm upset, it takes me a long time to feel better.*
ER_36_C	Way ar fil bad, ar kin ova gladi.<br>*When I'm upset, my emotions feel overwhelming.*

Boston College IRB Approved
October 18, 2019
Through August 20, 2020

FOR APPROVALS WITH NO CR REQUIRED:

Boston College IRB Approved
September 4, 2020-
September 3, 2021

SECTION 9: DEPRESSION & ANXIETY (HSCL_1_C - HSCL_25_C)
OW YU DAY DO

RESPONSE OPTIONS		
<<01>>	Natin-natin	*Not at all*
<<02>>	Smal	*A little*
<<03>>	Smal nor mor	*Quite a bit*
<<04>>	Boku boku wan	*Extremely*
<<998>>	Ar nor no	*Don't know*
<<999>>	Nor ansa	*No answer*

LABEL	Section 7: Di lis way day rite dong sai get diferen kayn wahala way pipul den kin get. Lisin fayn fayn to dem ol wan by wan en mekop aw dem bin day mona yu from las wik to tiday.<br><br>*Listed below are some symptoms or problems or worries that people sometimes have. Please listen to each one carefully and decide how much the problems bothered you in the last month, including today.*
LABEL	RAs: Use visual tool for this section
Item Code	Question
HSCL_1_C	From las mont to tiday, amos tem fraid fraid kin kam pan yu so nor mor for natin?<br>*From last month to today how much did you experience fear without cause?*
HSCL_2_C	From las mont to tiday, amos tem yu kin fil fraid?<br>*During the last month including today how much did you experience fear?*
HSCL_3_C	From las mont to tiday, amos tem yu fil taya bodi en yu hed de torn?<br>*During the last month including today how much did you experience weakness and your head turning?*
HSCL_4_C	From las mont to tiday, amos tem yu at day bit fas-fas?<br>*During the last month including today did how much has your heart been pounding?*
HSCL_5_C	From las mont to tiday, amos tem yu fil shake-shake na yu bodi?<br>*During the last month including today how much did you feel shaky inside your body?*
HSCL_6_C	From las mont to tiday, amos tem yu fil trimble trimble?<br>*During the last month including today how much did you tremble?*
HSCL_7_C	From las mont to tiday, amos tem yu hed kin at?<br>*During the last month including today how much did you have headaches?*
HSCL_8_C	From las mont to tiday, amos tem yu kin fraid nor mor en panik?<br>*During the last month including today how much did you get sudden feelings of fear and sudden panic?*
HSCL_9_C	From las mont to tiday, amos tem yu kin fil tense?<br>*During the last month including today how much did you feel tense?*
HSCL_10_C	From las mont to tiday, amos tem yu kin bet-bet?<br>*During the last month including today how much did you feel restless?*
HSCL_11_C	From las mont to tiday, amos tem yu nor kin get beteh trenk?<br>*During the last month including today how much did you not have enough energy/strength?*
HSCL_12_C	From las mont to tiday, amos tem yu kin blem yusef for tin dem way kin apin?<br>*During the last month including today how much did you blame yourself for things that occurred?*
HSCL_13_C	From las mont to tiday, amos tem yu kin day cry quik so nor mor?<br>*During the last month including today how much did you cry easily?*
HSCL_14_C	From las mont to tiday, amos tem yu don lef tin way yu bin lek for du way yu nor da ydu again?<br>*During the last month including today, how much did you stop doing things that you liked to do before that you do not do again?*
HSCL_15_C	From las mont to tiday, amos tem yu nor kin get apetite?<br>*During the last month including today, how much did you have poor appetite?*
HSCL_16_C	From las mont to tiday, amos tem e kin at for leh yu slip en yu nor kin get fayn slip?<br>*During the last month including today, how much did you have trouble falling asleep and not getting good sleep?*

Boston College IRB Approved
October 18, 2019
Through August 20, 2020

FOR APPROVALS WITH NO CR REQUIRED:

Boston College
IRB Approved
September 4, 2020-
September 3, 2021

HSCL_17_C	From las mont to tiday, amos tem yu fil se yu nor ab op again for yu tumara?<br>*During the last month including today, how much did you feel you have no hope again for tomorrow?*	
HSCL_18_C	From las mont to tiday, amos tem yu get poil at?<br>*During the last month including today, how much did you have sad heart?*	
HSCL_19_C	From las mont to tiday, amos tem yu fil for kip to yusef?<br>*During the last month including today, how much did you feel like keeeping to yourself?*	
HSCL_20_C	From las mont to tiday, amos tem yu memba se yu day don yu layf?<br>*During the last month including today, how much did you have thoughts of ending your life?*	Natin-natin ➔ WB_21_C <<00>><br>*Not at all*<br>Smal ➔ WB_21_C <<01>><br>*A little*<br>Smal nor mor ➔ WB_20A_C <<02>><br>*Quite a bit*<br>Boku boku wan ➔ WB_20A_C <<03>><br>*A lot*<br>Ar nor no ➔ WB_21_C <<998>><br>Nor ansa ➔ WB_21_C <<999>>
HSCL_20A_C	Stop day intaviu! kol day supavisor for leh e du di riks of ham safti plan. Yu kin kontinu di intaviu wans yu don wach en no if di posin wan for kil esef, di supavisor day na road, en yu don don di soshal kontrakt wit di patisipant, if nid day for am.<br>*Pause interview! Call supervisor to activate the risk of harm safety plan. You can continue the interview once you have assessed the suicidality of the participant, the supervisor is on the way, and you have completed a social contract with the participant, if needed.*	
HSCL_21_C	From las mont to tiday, amos tem yu kin fil tite insai yusef?<br>*During the last month including today, how much did you feel stuck?*	
HSCL_22_C	From las mont to tiday, amos tem yu kin worri-worri bot tin dem pas mak?<br>*During the last month including today, how much have you seriously been worried about things?*	
HSCL_23_C	From las mont to tiday, amos tem yu at nor kin get interes pan sometin?<br>*During the last month including today, how much did you lose interest in things?*	
HSCL_24_C	From las mont to tiday, amos tem yu fil se ol tin na wok way nor izi?<br>*During the last month including today how much did you feel that everything is difficult?*	
HSCL_25_C	From las mont to tiday, amos tem yu kin fil se yu na natin?<br>*During the last month including today how much did you have feelings of nothingness?*	

Boston College IRB Approved
October 18, 2019
Through August 20, 2020

FOR APPROVALS WITH NO CR REQUIRED:

Boston College IRB Approved
September 4, 2020-
September 3, 2021

SECTION 10: POST-TRAUMATIC STRESS DISORDER (PTSD_1_C – PTSD_17_C)

RESPONSE OPTIONS		
<<01>>	Nor	*No*
<<02>>	Yes	*Yes*

LABEL	Naw ar day kam aks yu som kweshon dem bot som tin dem way morna yu pasmak way don mit yu, yu bin day usai e apin, or yu bin fasin way mek yu fred pasmak, yu nor abul ep yu sef, or fiaful tin. If yu abul, yu<br><br>*Now I am going to ask you some questions about the most distressing event that you have experienced, witnessed, or were confronted with which caused intense fear, helplessness, or horror. If you feel able, can you tell me what that event was? Specify:*
PTSD_1_C	Di bad bad tin dem way yu don tok bot, na tin way apin pan di war?<br>*Was the distressing event you described above war-related?*
Label	Di statements den refer to tranga tin den way mit yu from way day war don. Duya ansah "YES" if e mit yu durin day 3 MONT way don pa.<br>*The following statements refer to difficulties you may have experienced since the end of the war. Please answer "YES" if you experienced the event described during the PAST THREE MONTHS.*
PTSD_2_C	Yu day memba oltem bot tin dem way day mek you fil bad or you day drim bot dem bad tin dem?<br>*Have you had recurrent or intrusive distressing thoughts or recollections about the trauma?*
PTSD_3_C	Yu bin don day get bad drim dem oltem, bot di bad tin dem way bin don apin?<br>*Have you been having recurrent bad dreams or nightmares about the trauma?*
PTSD_4_C	Yu maynd bin don toment pasmak wan way somtin mek yu memba tin dem way bad way bin don apin to yu? Evin lekway di patikula day rich bak way di tin apin?<br>*Have you been intensely EMOTIONALLY upset when reminded of the trauma includes anniversary reactions)?*
PTSD_5_C	Yu bin don day get tin dem way day mek yu bodi cheng kwiklek if yu day swet pasmak, you at day bit fas fas or oda tin way kin apin to yu. mor lek way somtin mek yu memba den bad tin dem?<br>*Have you been having intense PHYSICAL reactions like sweaty, heart palpitations or other things that happen when reminded of the trauma?*
PTSD_6_C	Yu bin don day tri oltem for mek yu nor memba or get enitin for du wit den bad tin dem way bin don apin to yu?<br>*Have you persistently been making efforts to avoid thoughts or feelings associated with the trauma?*
PTSD_7_C	Yu bin don day tri oltem for mek yu avoid tin dem way yu or oda pipul dem day du, way bin don apin or sai dem way day mek yu memba bot tin dem way bin don mona yu pasmak?<br>*Have you persistently been making efforts to avoid activities, situations, or places that remind you of the trauma?*
PTSD_8_C	Eni impotant tin/sai day pan den bad tin dem way apin way yu nor abul memba?<br>*Are there any important aspects about the trauma that you still cannot recall?*
PTSD_9_C	Yu nor lek egen for joke or du tin dem from way den bad tin ya apin to yu?<br>*Have you markedly lost interest in free time activities since the trauma?*
PTSD_10_C	Yu fil say yu nor day nia pipul dem way yu sabi, or push far from way den bad tin ya apin to yu?<br>*Have you felt detached or cut off from others around you since the trauma?*
PTSD_11_C	Yu bin don day fil say for mek yu abul sho filin don ambog lek yu norabul sho leknes to oda pipul dem?<br>*Have you felt that your ability to experience the whole range of emotions is impaired such as (i.e. unable to have loving feelings)?*
PTSD_12_C	Yu fil say eni plan way yu bin don mek for di bambai or tin way yu don abop pa don cheng bikos of di bad tin dem way apin to yu? Lek yu nor get wok, yu nor marade, nor get piking or long layf?<br>*Have you felt that any future plans or hopes have changed because of the trauma for example no career, marriage, children, or long life?*
PTSD_13_C	E tranga for mek yu slip or for mek yu tay pan slip?<br>*Have you had persistent difficulty falling or staying asleep?*
PTSD_14_C	Yu bin don day oltem vex pasmak kwik wan?<br>*Have you been continuously irritable or have outbursts of anger?*
PTSD_15_C	E kin mona for mek yu put yu atenshon pan somtin?<br>*Have you had persistent difficulty concentrating?*

Boston College IRB Approved
October 18, 2019
Through August 20, 2020

FOR APPROVALS WITH NO CR REQUIRED:

Boston College
IRB Approved
September 4, 2020-
September 3, 2021

PTSD_16_C	Yu olways day expet say somtin day apin to from way di tem way den bad tin dem apin to yu? For eksampul lek yu day luk for si udat day nia yu?<br>*Are you overly alert for example check to see who is around you since the trauma?*
PTSD_17_C	Yu kin skiad kwik wan pan yusef en mek lek yu nor day wit yusef, from di tem way den bad tin dem apin to yu?*Have you been jumpier, more easily startled, since the trauma?*

Boston College IRB Approved
October 18, 2019
Through August 20, 2020

FOR APPROVALS WITH NO CR REQUIRED:

Boston College IRB Approved
September 4, 2020-
September 3, 2021

SECTION 11: FUNCTIONING (FNC_1_C – FNC_21_C)

Label	Section 17: Pan dem tin ya way ar day kam kol, duya pik di wan way day sho gben aw yu welbodi day TIDAY.<br>*Under each heading, please pick the ONE answer that best describes your health TODAY.*	
Item Code	Questions	Coding Responses
FNC_1_C	For waka<br>*Mobility*	A nor get wan problem for waka <<00>><br>*I have no problems walking*<br>A get smal smal problem for waka <<01>><br>*I have slight problems walking*<br>A get smal problem for waka <<02>><br>*I have moderate problems walking*<br>A get sirios problem for waka <<03>><br>*I have severe problems walking*<br>A nor ebul waka <<04>><br>*I am unable to walk*
FNC_2_C	For tek kiya of yusef<br>*Self-care*	A nor get wan problem for was or dres misef <<00>><br>*I have no problems washing or dressing myself*<br>A get smal smal problem for was or dres <<01>><br>*I have slight problems washing or dressing myself*<br>A get smal problem for was or dres <<02>><br>*I have moderate problems washing or dressing myself*<br>A get sirios problem for was or dres <<03>><br>*I have severe problems washing or dressing myself*<br>A nor ebul for was or dres <<04>><br>*I am unable to wash or dress myself*
FNC_3_C	Tin dem way yu yus for du (lekeh: wok, buk lanin, os wok, fambul or gladi gladi tin dem)<br>*Usual activities (e.g. work, study, housework, family or leisure activities)*	A nor get wan problem for du di tin dem way a yus for du <<00>><br>*I have no problems doing my usual activities*<br>A get smal smal problem for du di tin dem way a yus for du<<01>><br>*I have slight problems*<br>A get smal problem for du di tin dem way a yus for du <<02>><br>*I have moderate problems*<br>A get sirios problem for du di tin dem way a yus for du <<03>><br>*I have severe problems*<br>A nor ebul for du di tin dem way a yus for du <<04>><br>*I am unable to do my usual activities*
FNC_4_C	Pain / somtin way day mona yu<br>*Pain/Discomfort*	A nor get wan pain or somtin way day mona mi <<00>><br>*I have no pain or discomfort*<br>A get smal smal pain or somtin way day mona mi <<01>><br>*I have slight pain or discomfort*<br>A get smal pain or somtin way day mona mi <<02>><br>*I have moderate pain or discomfort*<br>A get sirios pain or sirios tin way day mona mi <<03>><br>*I have severe pain or discomfort*<br>A get bad bad pain or bad bad tin way day mona mi <<04>><br>*I have extreme pain or discomfort*
FNC_5_C	Wondri en poil at<br>*Anxiety/Depression*	A nor day wondri or get wan poil at <<00>><br>*I am not anxious or depressed*<br>A day wondri smal smal wan or get smal poil at <<01>><br>*I am slightly anxious or depressed*<br>A day wondri smal or get small poil at <<02>><br>*I am moderately anxious or depressed*<br>A day wondri sirios wan or get get sirios poil at <<03>><br>*I am severely anxious or depressed*<br>A day wondri bad bad wan or get da bad bad poil at <<04>><br>*I am extremely anxious or depressed*

Boston College IRB Approved
October 18, 2019
Through August 20, 2020

FOR APPROVALS WITH NO CR REQUIRED:

Boston College
IRB Approved
September 4, 2020-
September 3, 2021

FNC_6_C	Wi go lek for no aw gud or bad yu welbodi wan day tiday.<br><br>*[GI AM KAD EN MAKA]*<br><br>Dis skale den nomba ram from 0 to 100- 100 minsay di best welbodi way yu kin tink bot, 0 min se di worst welbodi way yu kin tink bot. Duya mak x na di skale for sho aw yu welbodi day tiday.<br><br>*We would like to know how good or bad your health is today. (GIVE card and marker to participant) This scale is numbered from 0 to 100 – 100 means the best health you can imagine, 0 means the worst health you can imagine. Please mark an X on the scale to indicate how your health is TODAY.*	Yu welbodi wan tiday:<br>*Your health today:*<br><br>____________________<br>*[YU WAY DAY AKS KWESHON RAIT DI EGZAKT NOMBA WAY DI SIK MAN SHO]*<br><br>[Number item]
LABEL	Dis nex pat na bot problem dem way pipul den kin get bikoz of den nor get welbodi. Way a se den nor get welbodi, a min for get sik or nor day fil wel, or oda welbodi problem dem way no kin tae beteh, en di wan dem way kin tae pa posin, lek for wund/koz pain, way posin day mek lek e hed nor day, or yu get somtin way day wori yu maynd/chest pain, en rom or drugs problem. Memba al yu welbodi problem dem as yu day ansa den kweshon ya. Way a aks yu bot problem dem way yu day du somtin, duya tink bot for: add mor trenk/effort; toment or pain; way yu day du tin saful saful; aw yu don chanj in di way dem way yu day du som tin dem. A want yu bak way yu day ansa dem kweshon ya for tink bot amos problem yu bin get, dat way yu gess, insai di las 30 dayz, way yu bin day du den tin ya aw yu blant du am.<br><br>*This next part is about difficulties people have because of health conditions. By health condition I mean diseases or illnesses, or other health problems that may be short or long lasting; injuries; mental or emotional problems; and problems with alcohol or drugs. Remember to keep all your health problems in mind as you answer the questions. When I ask you about difficulties in doing an activity think about… Increased effort; Discomfort or pain; Slowness; Changes in the way you do the activity. When answering, I'd like you to think back over the last 30 days. I would also like you to answer these questions thinking about how much difficulty you have had, on average over the past month, while doing the activity as you usually do it.*	
Item Code	Questions	Coding Responses
FNC_7_C	Insai di pas 30 dayz, omos tranganes yu get tinap for long tem, lek 30 minits?<br>*In the past 30 days, how much difficulty did you have sstanding for long periods such as 30 minutes?*	No at wan <<00>><br>*None*<br>At smol <<01>><br>*Mild*<br>Nor tu at <<02>><br>*Moderate*<br>Pasmak wan <<03>><br>*Severe*<br>Pasmak pasmak wan or a nor kin ebul do am <<04>><br>*Extreme or can't do*<br>Ar no no <<998>><br>*Don't know*<br>Nor ansa <<999>><br>*No answer*
FNC_8_C	Insai di pas 30 dayz, omos tranganes yu bin get for du yu ose wok dem?<br>*In the past 30 days, how much difficulty did you have taking care of your household responsibilities?*	No at wan <<00>><br>At smol <<01>><br>Nor tu at <<02>><br>Pasmak wan <<03>><br>Pasmak pasmak wan or a nor kin ebul do am <<04>><br>Ar no no <<998>><br>Nor ansa <<999>>

Boston College IRB Approved
October 18, 2019
Through August 20, 2020

FOR APPROVALS WITH NO CR REQUIRED:

Boston College IRB Approved
September 4, 2020-
September 3, 2021

FNC_9_C	Insai di pas 30 dayz, omos tranganes yu bin get for lan niu wok, for egazampul, lan aw for go na niu ples/som sai?<br>*In the past 30 days, how much difficulty did you have learning a new task, for example, learning how to get to a new place?*	No at wan <<00>><br>At smol <<01>><br>Nor tu at <<02>><br>Pasmak wan <<03>><br>Pasmak pasmak wan or a nor kin ebul do am <<04>><br>Ar no no <<998>><br>Nor ansa <<999>>
FNC_10_C	Insai di pas 30 dayz, aw at e bin bi for yu for mek yu join/ tek pat pan community bizness (lek pan gladi gladi bizness, God biznes or oda tin dem) jes di sem way lek aw oda pipul den kin du?<br>*In the past 30 dez, how much of a problem did you have joining in community activities (for example, festivities, religious or other activities) in the same way as anyone else can?*	No at wan <<00>><br>At smol <<01>><br>Nor tu at <<02>><br>Pasmak wan <<03>><br>Pasmak pasmak wan or a nor kin ebul do am <<04>><br>Ar no no <<998>><br>Nor ansa <<999>>
FNC_11_C	Insai di pas 30 dayz, aw yu welbodi problem don wori yu at/maynd?<br>*In the past 30 days, how much have you been emotionally affected by your health problems?*	No at wan <<00>><br>At smol <<01>><br>Nor tu at <<02>><br>Pasmak wan <<03>><br>Pasmak pasmak wan or a nor kin ebul do am <<04>><br>Ar no no <<998>><br>Nor ansa <<999>>
FNC_12_C	Insai di pas 30 dayz, omos tem yu get tranganes for put yu maynd don for du somtin for 10 minits?<br>*In the 30 days, how much difficulty did you have concentrating on doing something for ten minutes?*	No at wan <<00>><br>At smol <<01>><br>Nor tu at <<02>><br>Pasmak wan <<03>><br>Pasmak pasmak wan or a nor kin ebul do am <<04>><br>Ar no no <<998>><br>Nor ansa <<999>>
FNC_13_C	Insai di pas 30 dayz, omos transganes yu bin get for waka go far ples, lek wan mile or sai way fa so?<br>*In the past 30 days, how much difficulty did you have walking a long distance such as a kilometre [or equivalent]?*	No at wan <<00>><br>At smol <<01>><br>Nor tu at <<02>><br>Pasmak wan <<03>><br>Pasmak pasmak wan or a nor kin ebul do am <<04>><br>Ar no no <<998>><br>Nor ansa <<999>>
FNC_14_C	Insai di pas 30 dayz, omos tranganes yu bin get for was yu ol bodi?<br>*In the past 30 days, how much difficulty did you have washing your whole body?*	No at wan <<00>><br>At smol <<01>><br>Nor tu at <<02>><br>Pasmak wan <<03>><br>Pasmak pasmak wan or a nor kin ebul do am <<04>><br>Ar no no <<998>><br>Nor ansa <<999>>
FNC_15_C	Insai di pas 30 dayz, omos transganes yu bin get for dres (for put yusef togeda)?<br>*In the past 30 days, how much difficulty did you have getting dressed?*	No at wan <<00>><br>At smol <<01>><br>Nor tu at <<02>><br>Pasmak wan <<03>><br>Pasmak pasmak wan or a nor kin ebul do am <<04>><br>Ar no no <<998>><br>Nor ansa <<999>>

Boston College IRB Approved
October 18, 2019
Through August 20, 2020

FOR APPROVALS WITH NO CR REQUIRED:

Boston College
IRB Approved
September 4, 2020-
September 3, 2021

FNC_16_C	Insai di pas 30 dayz, omos tranganes yu bin get for dil wit pipul dem way yu nor sabi?<br>*In the past 30 days, how much difficulty did you have dealing with people you do not know?*	No at wan <<00>><br>At smol <<01>><br>Nor tu at <<02>><br>Pasmak wan <<03>><br>Pasmak pasmak wan or a nor kin ebul do am <<04>><br>Ar no no <<998>><br>Nor ansa <<999>>
FNC_17_C	Insai di pas 30 dayz, omos tranganes yu bin get for kip padi biznes?<br>*In the past 30 days, how much difficulty did you have maintaining a friendship?*	No at wan <<00>><br>At smol <<01>><br>Nor tu at <<02>><br>Pasmak wan <<03>><br>Pasmak pasmak wan or a nor kin ebul do am <<04>><br>Ar no no <<998>><br>Nor ansa <<999>>
FNC_18_C	Insai di pas 30 dayz, omos tranganes yu bin bi get for du yu evriday wok/ skul wok?<br>*In the past 30 days, how much difficulty did you have in your day-to-day work or other activities?*	No at wan <<00>><br>At smol <<01>><br>Nor tu at <<02>><br>Pasmak wan <<03>><br>Pasmak pasmak wan or a nor kin ebul do am <<04>><br>Ar no no <<998>><br>Nor ansa <<999>>
FNC_19_C	Al dem problem ya wi don tok bot, a day kam ask yu kweshon den bot dem. Way yu chek am al, insai di pas 30 dayz, amos dezdem way dem tranganes ya bin day?<br>*Overall, in the past month, how many days were these difficulties present?*	Gee di exact:<br>➔___________(Days)
FNC_20_C	Insai di pas 30 dayz, for amos dayz way yu nor bin ebul for du di evriday tin dem or wok bikoz of dem tranganes ya bin day?<br>*In the past month, for how many days were you totally unable to carry out your usual activities or work because of these problems?*	Gee di exact:<br>➔___________(Days)
FNC_21_C	Insai di pas 30 dayz, apat from di day dem way yu nor bin ebul du anitin, for amos dez dem way yu bin ridyus pan di tin dem/wok dem way yu kin du, bikus of dem tranganes ya bin day?<br>*In the past month, not counting the days that you were totally unable, for how many days did you cut back or reduce your usual activities or work because of these problems?*	Gee di exact:<br>➔___________(Days)

Boston College IRB Approved
October 18, 2019
Through August 20, 2020

FOR APPROVALS WITH NO CR REQUIRED:

Boston College IRB Approved
September 4, 2020-
September 3, 2021

SECTION 12: CLOSING?

**LABEL**	Tenki, we almos don don.<br>*Thank you. We are almost finished.*
**END_1_C**	Aw e tan lek for mek a aks yu dem kweshion dem ya?<br>*What was it like for you to take this survey?*
**END_2_C**	Enitin day way yu wan for add way add a nor aks yu bot yet?<br>*Is there anything you would like to add that I didn't ask you about?*
**LABEL**	END: Tenki for yu tem way yu get fo mi! Tenki ya!<br>*Thanks for your time that you gave to me. Thanks!*

Boston College IRB Approved
October 18, 2019
Through August 20, 2020

FOR APPROVALS WITH NO CR REQUIRED:

Boston College IRB Approved
September 4, 2020-
September 3, 2021

CAREGIVER SCREENING INSTRUMENT

ASSIGN STUDY ID:
NAME:

SECTION 1. INFORMED CONSENT VERIFICATION

QUESTIONS FOR THE INTERVIEWER ONLY:

IC_C_1	WUS TEM DI INTAVIU STAT.<br>*INTERVIEW START DATE:*	DEZ<br>*DAY*	MONT<br>*MONTH*	IYA<br>*YEAR*
IC_C_2	INTAVIUA INITIAL DEM:<br>*INTERVIEWER'S INITIALS:*			

SECTION 2. ELIGIBILITY CRITERIA

PRIAMBUL: TENKI FOR WAY YU GRI FOR TOK TO MI EN TAK PAT PAN DIS SKRININ INTAVIU. WI GO AKS YU KWESHON DEM BOT PROBLEM DEM WAY YU GO DON GET, TIN DEM WAY YU GO DAY FIL EN TIN DEM WAY YU DAY DU WAY YU GET PROBLEM OR WAY YU AT POIL. DIS INTAVIU GO EP WI FOR NO IF YU GO TAK PAT OR YU NOR GO TAK PAT PAN DIS STODI. DUYA MEMBA SAY NO RAYT OR WRONG ANSA NOR DAY EN YU NOR GO ANSA ENI KWISHON WAY YU NOR WAN FOR ANSA.

PREAMBLE: THANK YOU FOR AGREEING TO SPEAK WITH ME AND PARTICIPATE IN THIS SCREENING INTERVIEW. WE WILL ASK YOU QUESTIONS ABOUT PROBLEMS YOU MIGHT HAVE, THINGS YOU MIGHT FEEL, AND THINGS YOU DO WHEN YOU HAVE PROBLEMS OR WHEN YOU ARE FEELING SAD. THIS INTERVIEW WILL ALLOW US TO DETERMINE IF YOU CAN BE A PART OF THIS STUDY OR NOT. PLEASE REMEMBER THAT THERE ARE NO RIGHT OR WRONG AND ANSWERS AND YOU DO NOT HAVE TO ANSWER ANY QUESTIONS YOU DO NOT WANT TO.

C_EC_1	WAETIN NA YU SURNAME?<br>*WHAT IS YOUR FAMILY NAME?*	GEE DI EXACT ANSA<br>*WRITE EXACT ANSWER*
C_EC_2	AMOS IA YU OL?<br>*HOW MANY YEARS OLD ARE YOU?*	GEE DI EXACT ANSA<br>*WRITE EXACT ANSWER*
C_EC_3	*ARE YOU CURRENTLY MARRIED OR LIVING WITH A PARTNER?*	☐ NO............................00<br>☐ YES............................01
C_EC_4	*ARE YOU CURRENTLY GOING THROUGH DIVORCE PROCEEDINGS?*	☐ NO............................00<br>☐ YES............................01
C_EC_5	*IF YOU ARE NOT LIVING WITH A PARTNER OR SPOUSE, IS THERE ANOTHER CAREGIVER IN THE HOME WHO SHARES CHILD CARE RESPONSIBILITIES (E.G., SISTER, AUNT, GRANDMOTHER)?*	☐ NO............................00<br>☐ YES............................01

Boston College IRB Approved
October 18, 2019
Through August 20, 2020

FOR APPROVALS WITH NO CR REQUIRED:

Boston College IRB Approved
September 4, 2020-
September 3, 2021

EMOTION REGULATION

RESPONSE OPTIONS		
<<01>>	E nor kin apin so normor	Almost never
<<02>>	Somtem dem	Sometimes
<<03>>	Somtem normor	About half the time
<<04>>	Boku tem dem	Most of the time
<<05>>	Almos altem	Almost always
<<998>>	Ar nor no	*Don't know*
<<999>>	Nor ansa	*No answer*

Label	Ar day kam rid list bot aw youg pipul kin somtem fil or ansa to satin situashone. Tink bot each wod dem fayn fayn wan en konsida if di wod dem kin bi to yu, altem, borku tem, smol tem, somtem or wan day. Memba say no rite or rong ansa nor day.<br><br>*I'm going to read a list of statements about how young people may sometimes feel or react to certain situations. Think about each statement carefully and consider whether the statement applies to you almost always, most of the time, about half the time, sometimes, or almost never. Remember, there are no right or wrong answers.*
Item Code	Kweshon<br>Question
Label	Enter Participant Screening ID
Label	Enter Enumerator ID
ER_1_C	Ar no sabi bot mi filin dem.<br>*I am not clear about my feelings.*
ER_2_C	Ar no day pay attenshon bot aw ar kin fil.<br>*I do not pay attention to how I feel.*
ER_3_C	Ar kin eksperens say mi filin dem pasmak en ar nor day abul kontrol dem.<br>*I experience my emotions as overwhelming and out of control.*
ER_4_C	Ar nor get no idia aw ar day fil.<br>*I have no idea how I am feeling*
ER_5_C	E kin tranga for ondastand bot mi filin dem.<br>*I have difficulty making sense out of my feelings.*
ER_6_C	Ar no kin abdul du somtin bot mi filin dem.<br>*I do not address my feelings.*
ER_7_C	Ar nor no gben aw ar day fil.<br>*I do not know exactly how I am feeling*
ER_8_C	Ar nor bisin bot waytin ar day fil.<br>*I do not care about what I am feeling.*
ER_9_C	Ar torment bot aw ar day fil.<br>*I am confused about how I feel.*
ER_10_C	Way ar fil bad, ar nor kin no bot filin dem.<br>*When I'm upset, I do not acknowledge my emotions.*
ER_11_C	Way ar fil bad, ar kin vex pa mi sef for way ar day fil da way day.<br>*When I'm upset, I become angry with myself for feeling that way (upset).*

Boston College IRB Approved
October 18, 2019
Through August 20, 2020

FOR APPROVALS WITH NO CR REQUIRED:

Boston College IRB Approved
September 4, 2020-
September 3, 2021

ER_12_C	Way ar fil bad, ar kin shem for way ar kin fil da way.<br>*When I'm upset, I become embarrassed for feeling that way (upset).*
ER_13_C	Way ar fil bad, e kin tranga for du wok.<br>*When I'm upset, I have difficulty getting work done.*
ER_14_C	Way ar fil bad, ar day go awt of kontrol.<br>*When I'm upset, I become out of control.*
ER_15_C	Way ar fil bad, ar beliv say ar go day pa am for long tem.<br>*When I'm upset, I believe that I will remain that way for a long time.*
ER_16_C	Way ar fil bad, ar beliv say ar kin end up mi filin dem wit poil at.<br>*When I'm upset, I believe that I will end up feeling very depressed.*
ER_17_C	Way ar fil bad, ar beliv say mi filin dem nor value en impotant.<br>*When I'm upset, I believe that my feelings are not valid and important.*
ER_18_C	Way ar fil bad, e kin at for mek ar put mi maynd pa oda tin dem.<br>*When I'm upset, I have difficulty focusing on other things.*
ER_19_C	Way ar fil bad, ar day los kontrol.<br>*When I'm upset, I feel out of control.*
ER_20_C	Way ar fil bad, ar nor day abul don for du somtin.<br>*When I'm upset, I cannot get things done.*
ER_21_C	Way ar fil bad, ar day fil shem pan mi sef for way ar day fil da way day.<br>*When I'm upset, I feel ashamed with myself for feeling that way.*
ER_22_C	Way ar fil bad, ar nor beliv say ar go fen way smol smol for mek ar fil beteh.<br>*When I'm upset, I don't believe that I can find a way to eventually feel better.*
ER_23_C	Way ar fil bad, ar kin fil lek ar wik.<br>*When I'm upset, I feel like I am weak.*
ER_24_C	Way ar fil bad, ar nor kin fil lek ar eabul kontrol di way aw ar day bihav.<br>*When I'm upset, I do not feel like I can remain in control of my behaviors.*
ER_25_C	Way ar fil bad, ar day fil gilti for di way ar day fil.<br>*When I'm upset, I feel guilty for feeling that way.*
ER_26_C	Way ar fil bad, e kin tranga for mek ar put mi maynd pan somtin.<br>*When I'm upset, I have difficulty concentrating.*
ER_27_C	Way ar fil bad, e kin tranga for mek ar kontrol di way ar day bihav.<br>*When I'm upset, I have difficulty controlling my behaviors.*
ER_28_C	Way ar fil bad, ar biliv say natin nor day way ar go du for misef way ar go fil beteh.<br>*When I'm upset, I believe there is nothing I can do to make myself feel better.*
ER_29_C	Way ar fil bad, ar day vex pan mi sef for way ar day fil da way day.<br>*When I'm upset, I become irritated with myself for feeling that way.*
ER_30_C	Way ar fil bad, ar kin bigin fil bad bot mi sef.<br>*When I'm upset, I start to feel very bad about myself.*
ER_31_C	Way ar fil bad, ar day tink bot am ol di tem.<br>*When I'm upset, I believe that wallowing in it is all I can do.*
ER_32_C	Way ar fil bad, ar day los kontrol of aw ar day bihav.<br>*When I'm upset, I lose control over my behaviors.*
ER_33_C	Way ar fil bad, e kin tranga for mek ar tink bot eni oda tin.<br>*When I'm upset, I have difficulty thinking about anything else.*

Boston College IRB Approved
October 18, 2019
Through August 20, 2020

FOR APPROVALS WITH NO CR REQUIRED:

Boston College
IRB Approved
September 4, 2020-
September 3, 2021

ER_34_C	Way ar fil bad, ar nor kin tek tem for no waytin ar day rili fil.<br>*When I'm upset, I do not take time to figure out what I'm really feeling.*
ER_35_C	Way ar fil bad, e kin tek long tem for mek ar fil beteh.<br>*When I'm upset, it takes me a long time to feel better.*
ER_36_C	Way ar fil bad, ar kin ova gladi.<br>*When I'm upset, my emotions feel overwhelming.*

SUICIDALITY

HSCL_20_C	From las mont to tiday, amos tem yu memba se yu day don yu layf?<br>*During the last month including today, how much did you have thoughts of ending your life?*	Natin-natin ➔ WB_21_C <<00>><br>*Not at all*<br>Smal ➔ WB_21_C <<01>><br>*A little*<br>Smal nor mor ➔ WB_20A_C <<02>><br>*Quite a bit*<br>Boku boku wan ➔ WB_20A_C <<03>><br>*A lot*<br>Ar nor no ➔ WB_21_C <<998>><br>Nor ansa ➔ WB_21_C <<999>>

SECTION 3. GIS MAPPING INFORMATION

GIS_1_C	*Where do you shop for food? At which market place?*
GIS_2_C	*If you attend church or mosque, which one do you attend? Where is it located?*
GIS_3_C	*If your children attend any school(s), which school(s) do they attend? What is the location?*
GIS_4_C	*Do you have family in the neighboring communities? If so, where do they live?*
GIS_5_C	*If you are currently working, where is your place of work or employment?*

SECTION 4. COLLECTING CONTACT INFORMATION

Tenki tenki for yu tem. wi go kep di infomashon tit en secret. Duya memba way yu tak pat pan di skrinin intaviu nor min sayden go tak yu pan de research program. Di research team go luk bak ol di skrinin intaviu en di posin na di team go kol yu for no waytin go hapin nex. For ep wi for mek wi contact yu, wi go lek for geda mor informashion, wi go kep dis informashion fyn fyn wan en sikrit
Thank you very much for your time. We will keep this information secure and confidential. Please remember your participation in the screening interview does not mean you will be enrolled in the research program. The research team will review all of the screening interviews and then a member of the team will call you to let you know what will happen next. ***If you are not eligible for the study, this does not mean anything is wrong with you or your family. If we find that our***

Boston College IRB Approved
October 18, 2019
Through August 20, 2020

FOR APPROVALS WITH NO CR REQUIRED:

Boston College
IRB Approved
September 4, 2020-
September 3, 2021

program works, we hope that all families in Sierra Leone will be able to receive it in the future. If you are not eligible to participate in our program, that does not mean you cannot participate in other available services in your community. If you do not know what those services are, we will be happy to connect you."

To help us contact you, we would like to collect some additional information. We will keep this information secure and confidential.

Fon nomba:<br>*Phone number:*	
Yu en oda posin day yus dis fon or na yu nor mor kin yus am?<br>*Do you share this phone with anyone else or is it your own personal number?*	
Yu kin yus WhatsApp?<br>*Do you use WhatsApp?*	
Us chifdom yu day?<br>*What chiefdom do you live in?*	
Us vilej yu day?<br>*What village do you live in?*	
Us aria yu day?<br>*What neighborhood do you live in?*	
Exakt adres?<br>*Exact address – including landmarks and other identifiers:*	

E kase wi nor abul for get yu, u go gi wi di nem of tu of u padi or fambul dem way go no usai yu day en aw for go to yu. Den for bi yu padi or fambul dem way go day na viliji or tong if yu go som sai. Memba contact nor for bi nor mor yu man, wife or pikin dem bikos den kin go wit yu. Wi want kontact bot posin way wuna day na di sam sai, e lek sef yu go oda sai.

In case we are unable to reach you, can you tell us the names of two friends or family members who are sure to know where you are and how to contact you? These should be friends or family that we could find in your village or town if you moved away. note: Contacts should not typically be spouses or children, since they usually move along with the respondent. We want contacts that are likely to stay in the same place when the respondent moves.

Kontakt 1<br>Contact 1:		Kontakt 2<br>Contact 2:	
Nem:<br>*Name:*		Nem:<br>*Name:*	
Udat di posin bi to yu?<br>*Relation to you?*		Udat di posin bi to yu?<br>*Relation to you?*	
Fon nomba:<br>*Phone number:*		Fon Nomba:<br>*Phone number:*	
Gi nomba (yes or no)?<br>*Shared number?*		Gi nomba (yes or no)<br>*Shared number?*	

Boston College IRB Approved
October 18, 2019
Through August 20, 2020

FOR APPROVALS WITH NO CR REQUIRED:

Boston College IRB Approved
September 4, 2020-
September 3, 2021

SECTION 5. PSYCHOLOGICAL ANALYSIS

THIS SECTION IS FOR THE INTERVIEWER TO ANSWER ONLY. DO NOT READ ALOUD THESE QUESTIONS TO THE PARTICIPANT.

Item Code	Question	Response
RH1_C	Yu biliv say dis partisipant day du tin dem way day sho say e geh problem wit e sens (fulful or efu lefu)?<br>*Do you believe that this participant displays impairment in cognitive ability?*	☐ NO……………….00<br>☐ YES……………..01
	Prompt de YES to RH1: Dis partisipant day sho say e don los e sens (fulful).<br>*Prompt if 'YES' to RH1: This participant displays cognitive impairments.*<br><br>Mak if posin wan fo du bad to insef or oda posin pan de result list.<br>*Tick 'NOT MET CRITERIA' on your Results Log.*	
RH2_C	Yu biliv say e fayn for leh den wach dis partisipant bak if e rili wan kil insef? Kil yu sef - men say di posin bin don tri for kil insef or e day tink for du bad to dem sef or geh plan for mek den geh wepon for du bad to dem sef.<br><br>*Do you believe that this participant requires further assessment regarding SERIOUS suicidality? Meaning that the person has made a prior suicide attempt, is actively thinking of harming themselves or has a plan with access to means to harm themselves, such as access to a weapon.*	☐ NO……………….00<br>☐ YES……………..01
	Prompt de YES RH2: Memba am wan tem if e say yes to RH3: posin wan du bad to insef or oda posin find out if den for wach dis participant if e kres<br>*Prompt if 'YES' to RH2: RISK OF HARM CASE. This participant needs a risk of harm assessment for suicidality.*<br><br>Kol supavisor wantem – wantem, start safeti plan.<br>*Call a supervisor immediately. Start the safety plan.*<br><br>Mak if posin wan fo du bad to insef or oda posin pan de result list.<br>*Tick 'RISK OF HARM' on your Results Log.*	
RH3_C	Yu biliv say e fayn for leh den wach dis partisipant bak for no if e day yeri, tok or si tin dem way oda posin nor day si? Day imagin say yu day si, yeri, or tok to posin way yu or oda pipul dem nor day si' –men say den geh problim wit den sens dem for no di tru lek for yeri vozy, si tin dem,or biliv say den na oda pipul dem or day som oda sai.<br>*Do you believe that this participant requires further assessment regarding psychosis? Meaning that they are having trouble with their sense of reality such as hearing voices, seeing things, or believing they are someone or somewhere else.*	☐ NO……………….00<br>☐ YES……………..01

Boston College IRB Approved
October 18, 2019
Through August 20, 2020

FOR APPROVALS WITH NO CR REQUIRED:

Boston College
IRB Approved
September 4, 2020-
September 3, 2021

	Memba am wan tem if e say yes to RH3: posin wan du bad to insef or oda posin find out if den for wach dis participant if e get kres sik<br>*Prompt if 'YES' to RH3: RISK OF HARM CASE. This participant needs a risk of harm assessment for psychosis.*<br><br>Kol supavisor wantem – wantem, start safeti plan.<br>*Call a supervisor immediately. Start the safety plan.*<br><br>Mak if posin wan fo du bad to insef or oda posin pan de result list.<br>*Tick 'RISK OF HARM' on your Results Log.*	
RH4_C	Yu biliv say day partisipant wan du bad to den sef? Way posin day wund, chuk, chap, or bet insef – men say di posin bin don tri for du bad to den sef baiwiful wan or e day tink gud gud wan for du bad to en sef tumara.<br>*Do you believe that the participant is at risk of harming themselves? Meaning that the person has made a prior attempt to harm themselves on purpose or is actively thinking of harming themselves in the future.*	☐ NO……………….00<br>☐ YES……………..01
	Memba ram wan tem if e say yes to RH3: posin wan du bad to insef or oda posin find out if den for wach dis participant if e get kres sik<br>*Prompt if 'YES' to RH4: RISK OF HARM CASE. This participant needs a risk of harm assessment for risk of self-harm.*<br><br>Kol supavisor wantem – wantem, start safeti plan<br>*Call a supervisor immediately. Start the safety plan.*<br><br>Mak if posin wan fo du bad to insef or oda posin pan de result list<br>*Tick 'RISK OF HARM' on your Results Log.*	
RH5_C	Yu biliv say di partisipant wan for du bad to oda pipul dem? Way di youth day plan for du bad to oda posin – men say di posin bin don day tri for du bad to oda pipul dis bein tem, e day rili day tink for du bad to oda posin or geh plan for du bad to posin lek e geh wepon for du bad to oda pipul dem.<br>*Do you believe that the participant is at risk of harming others? Meaning that the person has made a prior attempt to harm someone in recent times, is actively thinking of harming someone or has a plan with access or means to harm someone such as access to a weapon.*	☐ NO……………….00<br>☐ YES……………..01
	Memba ram wan tem if e say yes to RH5: posin wan du bad to insef or oda posin find out if den for wach dis participant if e wan du bad to oda pipul dem<br>*Prompt if 'YES' to RH5: RISK OF HARM CASE. This participant needs a risk of harm assessment for risk of harming others.*<br><br>Kol supavisor wantem – wantem, start safeti plan.<br>*Call a supervisor immediately or call the police, if needed. Start the safety plan.*<br><br>Mak if posin wan fo du bad to insef or oda posin pan de result list.<br>*Tick 'RISK OF HARM' on your Results Log.*	

Boston College IRB Approved
October 18, 2019
Through August 20, 2020

FOR APPROVALS WITH NO CR REQUIRED:

Boston College IRB Approved
September 4, 2020-
September 3, 2021

RH6_C	Yu biliv say di partisipnt nor saf, oda posin dem go du bad to am?Way posin day aras/fose di youth for du mami en dadi biznes,or way posin wan wund, chuk,chap.or bet di youth – men say den nor saf for leh den du mami en dadi biznes wit am or way posin go use oda tin den for wund am.<br>*Do you believe that the participant is at risk of being harmed by others? Meaning that they are at risk of sexual abuse or serious physical abuse.*	☐ NO……………….00<br>☐ YES………………01
	Krio translation needed Memba am wan tem if e say yes to RH5: posin wan du bad to insef or oda posin find out if den for wach dis participant if den wan du am bad (yus trenk/mami en dadi biznes)<br><br>*Prompt if 'YES' to RH6: RISK OF HARM CASE. This participant needs a risk of harm assessment for risk of harm by others (physical/sexual abuse).*<br><br>Kol supavisor wantem – wantem, start safeti plan.<br>Call a supervisor immediately. Start the safety plan.<br><br>Mak if posin wan fo du bad to insef or oda posin pan de result list.<br>*Tick 'RISK OF HARM' on your Results Log*.	

Boston College IRB Approved
October 18, 2019
Through August 20, 2020

FOR APPROVALS WITH NO CR REQUIRED:

Boston College IRB Approved
September 4, 2020-
September 3, 2021

MINI INTERNATIONAL NEUROPSYCHIATRIC INTERVIEW

Krio Version 1.0

USA: **D. Sheehan, D. Shytle, K. Milo, J. Janavs**
University of South Florida College of Medicine - Tampa, USA

FRANCE: **Y. Lecrubier**
Centre Hôpitalier Sainte-Anne - Paris, France

© Copyright 1998-2009 Sheehan DV and Lecrubier Y.

All rights reserved. No part of this document may be reproduced or transmitted in any form, or by any means, electronic or mechanical, including photocopying, or by any information storage or retrieval system, without permission in writing from Dr. Sheehan or Dr. Lecrubier. Researchers and clinicians working in nonprofit or publicly owned settings (including universities, nonprofit hospitals, and government institutions) may make paper copies of the instrument for their own clinical and research use.

DISCLAIMER

Our aim is to assist in the assessment and tracking of patients with greater efficiency and accuracy. Before action is taken on any data collected and processed by this program, it should be reviewed and interpreted by a licensed clinician. This program is not designed or intended to be used in the place of a full medical and psychiatric evaluation by a qualified licensed physician – psychiatrist. It is intended only as a tool to facilitate accurate data collection and processing of symptoms elicited by trained personnel.

Boston College IRB Approved
October 18, 2019
Through August 20, 2020

FOR APPROVALS WITH NO CR REQUIRED:

Boston College IRB Approved
September 4, 2020-
September 3, 2021

Patient Name	**Participant ID Code:**
Date of Birth	***Time Interview Began***
Interviewer's Name	***Time Interview Ended***
Date of Interview	***Total Time***

	**MODULE**	TIME FRAME	**MEETS CRITERIA**	**DSM-IV**	**ICD-10**	
		Past	☐	296.80	F31.9	☐
R	PSYCHOTIC DISORDERS	Lifetime<br>Current	☐<br>☐	295.10-295.90/297.1/<br>297.3/293.81/293.82<br>293.89/298.8/298.9	F20.xx-F29	☐
	MOOD DISORDER WITH PSYCHOTIC FEATURES	Lifetime<br>Current	☐<br>☐	296.24/296.34/296.44<br>296.24/296.34/296.44	F23.3/F33.3/<br>F30.2/F31.2/F31.5/<br>F31.8/F31.9/F39	☐

Boston College IRB
Approved
October 18, 2019
Through August 20, 2020

FOR APPROVALS WITH NO CR REQUIRED:

Boston College
IRB Approved
September 4, 2020-
September 3, 2021

INTERVIEWER INSTRUCTIONS

INTRODUCING THE INTERVIEW

The nature and purpose of the interview should be explained to the child or adolescent prior to the interview. A sample introduction is provided below:

"I'm going to ask you a lot of questions about yourself. This is so that I can get to know more about you and figure out how to help you. Most of the questions can be answered either 'yes' or 'no'. If you don't understand a word or a question, ask me, and I'll explain it. If you are not sure how to answer a question, don't guess - just tell me you are not sure. Some of the questions may seem weird to you, but try to answer them anyway. It is important that you answer the questions as honestly as you can so that I can help you. Do you have any questions before we start?"

CONVENTIONS:

Sentences written in «normal font» should be read exactly as written to the patient in order to standardize the assessment of diagnostic criteria.

Sentences written in «CAPITALS» should not be read to the patient. They are instructions for the interviewer to assist in the scoring of the diagnostic algorithms.

Sentences written in «**bold**» indicate the time frame being investigated. The interviewer should read them as often as necessary. Only symptoms occurring during the time frame indicated should be considered in scoring the responses.

Answers with an arrow above them (→) indicate that one of the criteria necessary for the diagnosis(es) is not met. In this case, the interviewer should go to the end of the module and circle «**NO**» in all the diagnostic boxes and move to the next module.

When terms are separated by a *slash (/)* the interviewer should read only those symptoms known to be present in the patient.

Phrases in (parentheses) are clinical examples of the symptom. These may be read to the patient to clarify the question.

RATING INSTRUCTIONS:

All questions must be rated. The rating is done at the right of each question by circling either Yes or No. Clinical judgment by the rater should be used in coding the responses. The rater should ask for examples when necessary, to ensure accurate coding. The child or adolescent should be encouraged to ask for clarification on any question that is not absolutely clear.

The clinician should take <u>each dimension</u> of the question into account (for example, time frame, frequency, severity, and/or alternatives).

Boston College IRB
Approved
October 18, 2019
Through August 20, 2020

R. PSYCHOTIC DISORDERS AND MOOD DISORDERS WITH PSYCHOTIC FEATURES

FOR APPROVALS WITH NO CR REQUIRED:

Boston College
IRB Approved
September 4, 2020-
September 3, 2021

ASK FOR AN EXAMPLE OF EACH QUESTION ANSWERED POSITIVELY. CODE YES ONLY IF THE EXAMPLES CLEARLY SHOW A DISTORTION OF THOUGHT OR OF PERCEPTION OR IF THEY ARE NOT CULTURALLY APPROPRIATE. BEFORE CODING, INVESTIGATE WHETHER DELUSIONS QUALIFY AS "BIZARRE". DELUSIONS ARE "BIZARRE" IF: CLEARLY IMPLAUSIBLE, ABSURD, NOT UNDERSTANDABLE, AND CANNOT DERIVE FROM ORDINARY LIFE EXPERIENCE. HALLUCINATIONS ARE SCORED "BIZARRE" IF: A VOICE COMMENTS ON THE PERSON'S THOUGHTS OR BEHAVIOR, OR WHEN TWO OR MORE VOICES ARE CONVERSING WITH EACH OTHER.

Naw a day kam aks yu bot somtin dem (ekspiriens dem) way nor komon way som pipul kin get.
Now I am going to ask you about unusual experiences that some people have.

					BIZARRE
R1	a	**Yu don eva biliv se pipul den day wach yu bay konin way yu nor lek?**<br>*Have you ever believed that people were secretly watching you?*<br><br>**Yu don eva biliv se sombodi don de tray fo kech yu o du yu bad?**<br>*Have you ever believed that someone was trying to get you, or hurt you?*<br><br>IF YES TO ANY, CODE YES<br>**NOTE:** ASK FOR EXAMPLES TO RULE OUT ACTUAL STALKING	**YES**	**NO**	**YES**
	b	**IF YES OR YES BIZARRE: Yu biliv so rayt naw?**<br>*Do you believe this now?*	**YES**	**NO**	**YES** → **R6**
R2	a	**Yu don eva biliv se sombodi de rid yu maynd (si waytin de nay yu at) o fil se sombodi de yeri (no) wetin yu de tink? Or yu don eva biliv fo say yu day ebul rid sombodi in yone mind or day yeri wetin den day tink?**<br>*Have you ever believed that someone was reading your mind or that someone could hear your thoughts? Or that you could actually read someone else's mind or hear what they were thinking?*<br><br>IF YES TO ANY, CODE YES	**YES**	**NO**	**YES**
	b	**IF YES OR YES BIZARRE: Yu biliv so naw?**<br>*Do you believe this now?*	**YES**	**NO**	**YES** → **R6**
R3	a	**Yu don eva biliv se sombodi or somtin put wod na yu yace way nor from yu e komot?**<br>*Have you ever believed that someone or something put thoughts in your mind that were not your own?*<br>**Yu don eva biliv se sombodi or somtin day mek yu du tin dem som kayn way way nor be yusef? Yu don eva fil se yu get debul?**<br>*Have you believed that someone or something made you act in a way that was not your usual self? Have you ever felt that you were possessed?*<br><br>IF YES TO ANY, CODE YES<br><br>**NOTE:** ASK FOR EXAMPLES AND DISCOUNT ANY THAT ARE NOT PSYCHOTIC	**YES**	**NO**	**YES**
	b	**IF YES OR YES BIZARRE: Yu biliv so rayt naw?**<br>*Do you believe this now?*	**YES**	**NO**	**YES** → **R6**

Boston College IRB Approved
October 18, 2019
Through August 20, 2020

FOR APPROVALS WITH NO CR REQUIRED:

Boston College
IRB Approved
September 4, 2020-
September 3, 2021

R4	a	**Yu don eva biliv se den de sen sikrit mesej dem (fo yu nomo) na tv, redio, intanet, nyuspepa, buk dem, or na yu gem dem or ple-ple tin dem? Yu don eva biliv se sombodi way yu nor sabi way i jes si yu lek yu tumos wan kayn way way yu nor lek?**<br>*Have you ever believed that you were being sent special messages through the TV, radio, internet, newspapers, books, magazines, or through your games or toys?*<br>*Have you ever believed that a person you did not personally know was especially interested in you?*<br><br>IF YES TO ANY, CODE YES	**YES**	**NO**	**YES**
	b	**IF YES OR YES BIZARRE: Yu biliv so rayt naw?**<br>*Do you believe this now?*	**YES**	**NO**	**YES** → **R6**
R5	a	**Yu fambul dem or padi dem don eva fil se dem yu bilivin ya den na lay lay/strenj? Duya gi mi egzampul.**<br>*Have your family or friends ever thought that any of your beliefs were strange or weird? Please give me an example.*<br><br>INTERVIEWER: ONLY CODE YES IF THE EXAMPLES ARE CLEARLY DELUSIONAL AND ARE NOT EXPLORED IN QUESTIONS R1 TO R4, FOR EXAMPLE, SOMATIC OR RELIGIOUS DELUSIONS OR DELUSIONS OF GRANDIOSITY, JEALOUSY GUILT, RUIN OR DESTITUTION, ETC.	**YES**	**NO**	**YES**
	b	**IF YES Or YES BIZARRE: Den stil tink se den yu bilivin ya na lay/strenj?**<br>*Do they still think that your beliefs are strange?*	**YES**	**NO**	**YES**
R6	a	**Yu don eva yeri tin den way oda pipul den no bin ebul yeri, lek voys den?**<br>*Have you ever heard things other people couldn't hear, such as voices?*	**YES**	**NO**	
		HALLUCINATIONS ARE SCORED "BIZARRE" ONLY IF PATIENT ANSWERS YES TO THE FOLLOWING:<br>**IF YES: Yu bin yeri voys den de tok bot yu? Yu bin yeri pas wan voys de tok go en tok kam?**<br>*Did you hear a voice talking about you? Did you hear more than one voice talking back and forth?*	**YES**		**YES**
	b	**IF YES OR BIZARRE TO R6: Yu don yeri den tin ya insay di pas mont?**<br>*Have you heard these things in the past month?*<br><br>HALLUCINATIONS ARE SCORED "BIZARRE" ONLY IF PATIENT ANSWERS YES TO THE FOLLOWING:<br>**Yu bin yeri voys den de tok bot yu? Yu bin yeri pas wan voys de tok go en tok kam?**<br>*Did you hear a voice talking about you? Did you hear more than one voice talking back and forth?*	**YES**	**NO**	**YES** → **R8**
R7	a	**Yu don eva si somtin we get fo apin or yu don eva si tin den we oda pipul den no bin ebul si?**<br>*Have you ever had visions or have you ever seen things other people couldn't see?*<br>NOTE: CHECK TO SEE IF THESE ARE CULTURALLY INAPPROPRIATE.	**YES**	**NO**	
	b	**IF YES: Yu don si den tin/drim den tin ya insay di pas mont?**<br>*Have you seen these things in the past month?*<br><br>**CLINICIAN'S JUDGMENT**	**YES**	**NO**	
R8	b	IS THE PATIENT CURRENTLY EXHIBITING INCOHERENCE, DISORGANIZED SPEECH, OR MARKED LOOSENING OF ASSOCIATIONS?	**YES**	**NO**	

Boston College IRB
Approved
October 18, 2019
Through August 20, 2020

FOR APPROVALS WITH NO CR REQUIRED:

Boston College
IRB Approved
September 4, 2020-
September 3, 2021

R9	b	IS THE PATIENT CURRENTLY EXHIBITING DISORGANIZED OR CATATONIC BEHAVIOR?	**YES**	**NO**
R10	b	ARE NEGATIVE SYMPTOMS OF SCHIZOPHRENIA, E.G. SIGNIFICANT AFFECTIVE FLATTENING, POVERTY OF SPEECH (ALOGIA) OR AN INABILITY TO INITIATE OR PERSIST IN GOAL DIRECTED ACTIVITIES (AVOLITION), PROMINENT DURING THE INTERVIEW?	**YES**	**NO**
R11	a	ARE 1 OR MORE <a> QUESTIONS FROM R1a TO R7a CODED **YES OR YES BIZARRE** AND IS EITHER:<br>MAJOR DEPRESSIVE EPISODE, (CURRENT OR RECURRENT)<br>OR<br>MANIC OR HYPOMANIC EPISODE, (CURRENT OR PAST) **CODED YES**?	**YES →R13**	**NO**

b **Yu bin fos tel mi se yu bin get tem dem way yu bin (get poil at/ yu gladi pasmak, lek waytin wi bin tok bot /yu kwik for veks oltem).**
You told me earlier that you had period(s) when you felt (depressed/high/persistently irritable)

YES **NO**

Yu bin day get den bilivin ya way yu jes tok bot normor way yu bin get poil at?
Did you have the beliefs and experiences you just described only when you were feeling depressed?

Yu bin day get den bilivin ya way yu jes tok bot normor way yu bin day fil gladi pasmak?
High

Yu bin day get den bilivin ya way yu jes tok bot normor way yu bin kwik for chanje or yu at easi for wam?
Very moody?

Yu bin day get den bilivin ya way yu jes tok bot nomor way yu bin day kwik fo veks oltem?
Very irritable?

[GIVE EXAMPLES TO PATIENTS FROM SYMPTOMS CODED YES FROM R1a TO R7a]

IF THE PATIENT EVER HAD A PERIOD OF AT LEAST 2 WEEKS OF HAVING THESE BELIEFS OR EXPERIENCES (PSYCHOTIC SYMPTOMS) WHEN THEY WERE NOT DEPRESSED/HIGH/IRRITABLE, CODE NO TO THIS DISORDER.

IF THE ANSWER IS NO TO THIS DISORDER, ALSO CIRCLE NO TO R12 AND MOVE TO R13

MOOD DISORDER WITH PSYCHOTIC FEATURES

LIFETIME

R12 a ARE 1 OR MORE <b> QUESTIONS FROM R1b TO R7b CODED **YES OR YES BIZARRE** AND IS EITHER:
MAJOR DEPRESSIVE EPISODE, (CURRENT)
OR
MANIC OR HYPOMANIC EPISODE, (CURRENT) **CODED YES**?

IF THE ANSWER IS YES TO THIS DISORDER (LIFETIME OR CURRENT) CIRCLE NO TO R13 AND R14 AND MOVE TO THE NEXT MODULE

YES **NO**

MOOD DISORDER WITH PSYCHOTIC FEATURES

CURRENT

Boston College IRB Approved
October 18, 2019
Through August 20, 2020

FOR APPROVALS WITH NO CR REQUIRED:

Boston College IRB Approved
September 4, 2020-
September 3, 2021

R13 ARE 1 OR MORE <b> QUESTIONS FROM R1b TO R6b CODED **YES BIZARRE**

OR

ARE 2 OR MORE <b> QUESTIONS FROM R1b TO R10b, CODED **YES** (RATHER THAN **YES BIZARRE**)?

AND DID AT LEAST TWO OF THE PSYCHOTIC SYMPTOMS OCCUR DURING THE SAME 1 MONTH PERIOD?

**YES** **NO**
PSYCHOTIC DISORDER
CURRENT

R14 IS **R13** CODED **YES**

OR

ARE 1 OR MORE <a> QUESTIONS FROM R1a TO R6a CODED **YES BIZARRE?**

OR

ARE 2 OR MORE <a> QUESTIONS FROM R1a TO R7a CODED **YES** (RATHER THAN **YES BIZARRE**)?

AND DID AT LEAST TWO OF THE PSYCHOTIC SYMPTOMS OCCUR DURING THE SAME 1 MONTH PERIOD?

**YES** **NO**
PSYCHOTIC DISORDER
LIFETIME

Boston College IRB Approved
October 18, 2019
Through August 20, 2020

FOR APPROVALS WITH NO CR REQUIRED:

Boston College IRB Approved
September 4, 2020-
September 3, 2021

mHealth Tools to Improve Service Delivery Quality of an Evidence-Based Family Home Visiting Intervention
R21 Caregiver Assessment Battery

Table of Measures

**Section 1: Anthropometrics**	Demographic and Household Survey
**Section 2: Household Composition**	
**Section 3: Education**	
**Section 4: Health**	
**Section 5: Intimate Partner Relationships**	Revised Conflict Tactics Scale (CTS2)
**Section 6: Emotion Regulation**	Difficulties in Emotional Regulation (DERS)
**Section 7: Anxiety and Depression**	Hopkins Symptoms Checklist (HSCL)
**Section 8: Post-traumatic Stress**	PTSD Civilian Checklist
**Section 9: Functioning**	WHO Disability Assessment Schedule
**Section 10: Parent-Child Interactions**	Observation of Mother-Child interactions (OMCI)
**Section 11: Home Environment**	Home Observation for Measurement of the Environment (HOME)
**Section 12**	Closing

Boston College IRB Approved
October 18, 2019
Through August 20, 2020

FOR APPROVALS WITH NO CR REQUIRED:

Boston College
IRB Approved
September 4, 2020-
September 3, 2021

SECTION 1: **Anthropometrics**

	Enter caregiver ID
	Enter child ID
	Enter enumerator ID
AN_1_C	Amos iya di pikin ole ?<br>*What is the child's age?*
AN_2_C	Us tem yu pikin bon?<br>*What is child's birthday?*
AN_3_C	*Calculate child's age in months.*
AN_4_C	Pik di dokument for sho di tem way di pikin bon.<br>*Select document used to verify child's birthdate.*
AN_5_C	Di pikin (pikin in nem) get pepa way sho di tem way e bon?<br>*Does (child's name) have a birth certificate?*
AN_6_C	Pik if na boy or gal pikin<br>*Select sex of child.*
AN_7_C	Duya put di fos leta for di pikin in fos nem.<br>*Enter first letter of child's name.*
AN_8_C	Duya put di fos leta for di pikin in las nem/sornem.<br>*Enter first letter of the child's last name/surname.*
LABEL	*Ask caregiver to undress child as much as possible.*
AN_9_C	*Was the child undressed to the minimum?*
AN_10_C	*How will the child's weight be assessed?*
AN_11_C	*Caregiver and the child together (kg)*
AN_12_C	*Caregiver alone (kg)*
AN_13_C	*Child's weight (kg)*
LABEL	*Age under 2 years: measure lengthy lying down;*<br>*Age 2 years or more: measure height of child standing*
AN_14_C	*Child's length (in cm)*
AN_15_C	*Child's height (in cm)*
AN_16_C	*Enter child's MUAC (cm)*

Boston College IRB
Approved
October 18, 2019
Through August 20, 2020

Boston College
IRB Approved
September 4, 2020-
September 3, 2021

SECTION 2: HOUSEHOLD COMPOSITION (HC_1_C- HC_15_C)

Label	Naw ar day kam aks yu som kweshon dem bot yu en di wan dem way una day it na di sam pot. Yu go tok bot den pipul dem ya eni aw yu go lek, wans yu abul tok bot alman.<br><br>*Now I am going to ask you several questions about EACH person who lives in your household. You can talk about these people in any order you would like, just as long as we talk about every person.*
HC_1_C	 *Enter caregiver sex*
HC_2_C	Ol in ol, amos pipul dem day liv na yu os way una day it na di sam pot?<br>*In total, how many people live in your household?*
HC_3_C	Ol in ol, amos pikin den day way na 17 iyas or nor rich, way den day liv na yu os?<br>*In total, how many children 17 years old or younger live in your household?*
HC_4_C	Amos bele bon pikin den yu get naw? (*Record number)*<br>*How many biological children do you have at present?*
HC_5_C	Amos pikin dem way nor to yu born day nar yu kiya naw naw?<br>*How many non-biological children are you presently the principal caretaker for?*
HC_6_C	Aw yu way day tek kia fambul to di pikin (pikin in nem)?<br>*What is your relationship to (child's name)?*
HC_7_C	 *What is your current marital status?*
HC_8_C	 *Does your partner currently live with you at least half of the time?*
HC_9_C	Apat from yu, oda posin day way day liv na di ose way day tek kia of dis pikin naw?<br>*Besides yourself, is there another primary caregiver for the child that lives in the home currently?*
HC_10_C	Di posin don liv na dis ose pas 15 days insai di las 30 days?<br>*Has he/she lived at home for more than 15 days in the last 30 days?*
HC_11_C	Aw di oda posin way day tek kia fambul to di pikin (pikin in nem)?<br>*What is the other caregiver's relationship to (child's name)?*
HC_12_C	Di posin way lan buk pas una ol na dis ose usai e tap pan buk lanin?<br>*What is the highest level of education completed by anyone in the household?*
LABEL	Den kweshon dem ya day aks yu for tok bot tin dem way yu en yu man/uman day du way una day wan plas wit una pikin (lek na rum, na motoka, or way una komot). Na for kont di tem den normor way una tri day wan ples (ar lek na for tu, tri awa normor for di wik). **Amos tem pa wik way una ol tri kin day togeda way una day...**<br><br>*These questions ask you to describe things you do when both you and your partner are physically present together with your child (i.e. in the same room, in the car, on outings). Count only times when all three of you are actually within the company of one another (even if this is just a few hours per week). How often in a typical week, when all 3 of you are together, do you:*
HC_13_C	Argu bot una marade ose biznes way nor konsan di pikin bifo di pikin?<br>*Argue about your relationship or marital issues unrelated to your child, in the child's presence?*
HC_14_C	Wan posin or una tu day tel in kompin bad wod bifo di pikin?<br>*One or both of you say cruel or hurtful things to each other in front of the child?*
HC_15_C	Aw di oda posin way day tek kia fambul to di pikin (pikin in nem)?<br>What is the other caregiver's relationship to (child's name)?

Boston College IRB
Approved
October 18, 2019
Through August 20, 2020

FOR APPROVALS WITH NO CR REQUIRED:

Boston College
IRB Approved
September 4, 2020-
September 3, 2021

Boston College IRB Approved
October 18, 2019
Through August 20, 2020

FOR APPROVALS WITH NO CR REQUIRED:

Boston College IRB Approved
September 4, 2020-
September 3, 2021

SECTION 3: EDUCATION (ED_1_C – ED_3_C)

Label	Section 3: Naw ar go aks yu kweshon dem bot yu buk laning.<br><br>*Now I would like to ask you some questions about your education and literacy.*	
ED_1_C	Wus fom yu tap?<br>*What is the highest grade/form you completed?*	P1 <<01>><br>P2 <<02>><br>P3 <<03>><br>P4 <<04>><br>P5 <<05>><br>P6 <<06>><br>JSS1 <<07>><br>JSS2 <<08>><br>JSS3 <<09>><br>SS1 <<10>><br>SS2 <<11>><br>SS3 <<12>><br>Ar neva go skul <<00>><br>Ar nor no <<998>><br>Nor ansa <<999>>
ED_2_C	Aw wel yu ebul rid?<br>How well can you read?	Yu nor sabi rid <<01>><br>Yu sabi rid smul – <<02>><br>smul but I slo en tranga fur du am<br>Yu don sabi smul fur rid <<03>><br>Yu sabi en rid fayn-fayn wan<br><<04>><br>Ar nor no <<998>><br>Nor ansa <<999>>
ED_3_C	Aw wel yu ebul rite?<br>How well can you write?	Yu nor sabi rite <<01>><br>Yu sabi rite smal – <<02>><br>smal but e slo en tranga for du am<br>Yu don sabi smal for rite <<03>><br>Yu sabi en rite fayn-fayn wan<br><<04>><br>Ar nor no <<998>><br>Nor ansa <<999>>

Boston College IRB
Approved
October 18, 2019
Through August 20, 2020

FOR APPROVALS WITH NO CR REQUIRED:

Boston College
IRB Approved
September 4, 2020-
September 3, 2021

Label	Section 4: Naw A go lek for aks yu som question bot yu welbodi en aw yu day du. Duya memba se no rait or rong ansa nor day. Jes gee day ansa way yu fil se e rait.<br><br>*Now I would like to ask you some questions about your health and general well-being. Please remember that there are no right or wrong answers. Just give the answer that fits best for you.*	
Item Code	Questions	Coding Responses
HE_1_C	Way yu kompia yu sef to oda posin way na yu ag grup yu go say yu welbodi na?<br>*Compared to another person of your age, would you say your health is:*	Bad-off pas oda pipul <<01>><br>*Worse than others*<br>Dae same lek oda pipul <<02>><br>*Same as others*<br>Bette pas oda pipul <<03>><br>*Better than the others*<br>Ar nor no <<998>><br>Nor ansa <<999>>
HE_2_C	Omos dez insai di pas mont way yu nor ebul du di wok dem way yu blant du bikoz yu mind or yu at nor bin rest?<br>*How often in the past one month were you unable to do the work you usually do because of an emotional or psychological problem?*	Gee di exact <<00>> ➔HE_2A_C<br>Ar nor no <<998>>➔HE_3_C<br>Nor ansa <<999>>➔HE_3_C
	HE_2A_C — Gee di exact ansa:<br>*Write exact answer:*<br>➔ ____________________	
HE_3_C	Omos dez insai di pas mont way yu ledon na bed pas haf day bikoz yu mind or yu at nor bin rest?<br>*How often in the past one month did you have to lie in bed for more than half a day because because of an emotional or psychological problem?*	Gee di exact <<00>> ➔HE_3A_C<br>Ar nor no <<998>>➔HE_4_C<br>Nor ansa <<999>>➔HE_4_C
	HE_3A_C — Gee di exact ansa:<br>➔ ____________________	
HE_4_C	Insai di pas mont, omos hard time yu bin get for du yu os wok bikoz yu mind or yu at nor bin rest?<br>*During the past month, how much difficulty did you have in taking care of your household responsibilities because of an emotional or psychological problem?*	Natin-natin <<00>><br>*Not at all*<br>Smal <<01>><br>*A little*<br>Small nor mor <<02>><br>Quite a bit<br>Borku borku wan <<03>><br>Extremely<br>Ar nor no <<998>><br>Nor ansa <<999>>

Boston College IRB
Approved
October 18, 2019
Through August 20, 2020

FOR APPROVALS WITH NO CR REQUIRED:

Boston College
IRB Approved
September 4, 2020-
September 3, 2021

SECTION 5: INTIMATE PARTNER RELATIONSHIPS (CTS_1_C-CTS_27_C)

Label	Section 5: Naw ar go ask yu som kweshon dem bot di way way yu day wit yu patna or boyfrien/girlfrien/wef/man.<br><br>*Now I will ask you some questions about your relationships with your partner. (boyfriend/girlfriend/husband/wife).*	
Item Code	Questions	Coding Responses
CTS_1_C	Yu bin don eva married or bin don day wit eni patna/man/uman?<br>*Have you ever been married or had a partner?*	No <<00>> → Section 6<br>Yes <<01>> → CTS_2_C<br>Ar nor nor <<998>> → Section 6<br>Nor ansa <<999>> → Section 6
CTS_2_C	Yu married or yudaywit eni patna/man/uman naw naw?<br>*Are you currently married or have a partner?*	No <<00>> → CTS_4_C<br>Yes <<01>> → CTS_3_C<br>Ar nor nor <<998>> → Section 6<br>Nor ansa <<999>> → Section 6
CTS_3_C	Yu day liv wit yu patna/man/wef naw naw?<br>*Do you live with your partner now?*	No <<00>> → Section 6<br>Yes <<01>> → CTS_5_C<br>Ar nor nor <<998>> → Section 6<br>Nor ansa <<999>> → Section 6
CTS_4_C	*Have you been married or had a partner in the last three months?*	No <<00>> → Section 6<br>Yes <<01>> → CTS_5_C<br>Ar nor nor <<998>> → Section 6<br>Nor ansa <<999>> → Section 6

RESPONSE OPTIONS

<<00>>	Dis nor wan day apin	*This has never happened*
<<01>>	Wan tem insai di pas ia	*Once in the past three months*
<<02>>	Tu tem insai di pas ia	*Twice in the past three months*
<<03>>	Tri or feiv tem insai di pas ia	*3 to 5 times in the past three months*
<<04>>	Six or ten tem insai di pas ia	*6 to 10 times in the past three months*
<<05>>	Elevin or twenty tem insai di pas ia	*11-20 times in the past three months*
<<06>>	Pas twenty tem insai di pas ia	*More than 20 times in the past three months*
<<07>>	Nor bi insai di pas iya, bot e bin apin bifo	*Not in the three months, but it did happen before*
<<998>>	Ar nor nor	*Don't know*
<<999>>	No ansa	*No answer*

Boston College IRB Approved
October 18, 2019
Through August 20, 2020

FOR APPROVALS WITH NO CR REQUIRED:

Boston College
IRB Approved
September 4, 2020-
September 3, 2021

Label	E nor mata aw fayn, way marade man en uman dem day, tem go kam way den nor go gri, vex pan oda posin, den kin want difren tin dem from dem sef, mek palava or dem jos fet bikos den nor gladi, den taya or for som oda risin den. Marade man en uman den kin get boku way dem for tri en setul den problem dem. Dis na di lis of tin-dem way som tem go apin way den get palava. Duya sho omos tem yu bin du wan pan den tin dem yah insai di pas ia, en omos tem yu patna bin du dem insai di pas ia. Ef yu or yu partna nor bin du wan pan den tin dem ya insai di pas iya, bot e apin bifo dat, tok am.<br><br>*No matter how well a couple gets along, there are times when they disagree, get annoyed with the other person, want different things from each other, or just have spats or fights because they are in a bad mood, are tired, or for some other reason. Couple also have many different ways of trying to settle their differences. This is a list of things that might happen when you have differences. Please indicate how many times you did each of these things in the past year, and how many times your partner did them in the past year. If you or your partner did not do one of these things in the past year, but it happened before that, say this.*<br><br>Yu partna way yu day wit naw or di wan way yu bin day wit dis beyen tem, eni tin bin don day way una nor kam to wan pan way mek yu du den tin ya: DO NOT READ "NO ANSA", "AR NO NO", OR "NOT APPLICABLE"<br><br>*With your current or most recent partner were there any circumstances or household disagreements that caused you to do the following things:*
CTS_5_C	Yu eva kos yu patna/man/uman?<br>*Used abusive language at your partner.*
CTS_6_C	Yu eva lef yu patna/man/uman?<br>*Abandon/forsake your partner*
CTS_7_C	Yu eva push/shub yu patna/man/uman?<br>*Pushed or shoved your partner*
CTS_8_C	Yu eva bokul yu patna/man/uman?<br>*Grabbed your partner*
CTS_9_C	Yu eva slap yu patna/man/uman wit yu an?<br>*Slapped your partner with hand*
CTS_10_C	Yu eva yus nef or oda wepon pan yu patna/man/uman?<br>*Used a knife or other weapon on your partner*
CTS_11_C	Yu eva nak yu patna/man/uman wit eni tin way go damaj am?<br>*Hit your partner with an object that could hurt him/her*
CTS 12_C	Yu eva jam yu patna/man/uman pan di wol?<br>*Slammed your partner against a wall*
CTS_13_C	Yu eva kik yu patna/man/uman?<br>*Kicked your partner*
CTS_14_C	Yu eva yus fors pan yu patna/man/uman (lek for nak, bokul, or yus wepon) for du mami en dadi biznes?<br>*Used force (like hitting, holding down, or using a weapon) to make your partner have sex even though they didn't want to*
CTS_15_C	Yu partna way yu day wit naw or di wan way yu bin day wit, amos tem una kin mek palava insai di mont?<br>*In your relationship with your (current or most recent) partner, how often would you say that you quarrel in an average month?*
	Now I am going to ask some questions about things your current or most recent partner may have done to you. Yu partna way yu day wit naw, or di wan way yu bin day wit dis beyen tem, bin don eva du eni wan pan den tin ya:<br>*Has your current or most recent partner ever:*
CTS_16_C	Yu partna eva kin kos yu?<br>*Used abusive language*
CTS_17_C	E eva lef yu?<br>*Abandoned/forsaken you*
CTS_18_C	E eva push yu?<br>*Pushed or shoved you*
CTS_19_C	E eva bokul yu?<br>*Grabbed you*
CTS_20_C	E eva slap or pata yu?<br>*Slapped you or hit you*

Boston College IRB Approved
October 18, 2019
Through August 20, 2020

FOR APPROVALS WITH NO CR REQUIRED:

Boston College
IRB Approved
September 4, 2020-
September 3, 2021

CTS_21_C	E eva us nef or oda wepon pan yu?<br>*Used a knife or other weapon on you*
CTS_22_C	E eva nak yu wit eni tin way go damaj yu?<br>*Hit you with an object that could hurt*
CTS_23_C	E eva jam yu pan di wol?<br>*Slammed you against a wall*
CTS_24_C	E eva kik yu?<br>*Kicked you*
CTS_25_C	E eva fet yu bone to bone for mek una du mami en dadi biznes way yu nor want?<br>*Physically forced you to have sexual intercourse when you did not want to?*
CTS_26_C	Yu bin don eva du mami en dadi biznes bikoz yu day fraid say if yu nor gri e go du bad to yu?<br>*Did you ever have sexual intercourse you did not want because you were afraid of what he/she might do?*
CTS_27_C	E bin don eva fos yu for du sometin lek mami en dadi biznes way shameful to yu?<br>*Did he ever force you to do something sexual that you found degrading or humiliating?*

Boston College IRB Approved
October 18, 2019
Through August 20, 2020

FOR APPROVALS WITH NO CR REQUIRED:

Boston College IRB Approved
September 4, 2020-
September 3, 2021

SECTION 6: EMOTION REGULATION (ER_1_C – ER_36_C)

RESPONSE OPTIONS		
<<01>>	E nor kin apin so normor	Almost never
<<02>>	Somtem dem	Sometimes
<<03>>	Somtem normor	About half the time
<<04>>	Boku tem dem	Most of the time
<<05>>	Almos altem	Almost always
<<998>>	Ar nor no	*Don't know*
<<999>>	Nor ansa	*No answer*

Label	Section 10: Ar day kam rid list bot aw youg pipul kin somtem fil or ansa to satin situashone. Tink bot each wod dem fayn fayn wan en konsida if di wod dem kin bi to yu, altem, borku tem, smol tem, somtem or wan day. Memba say no rite or rong ansa nor day.<br><br>*I'm going to read a list of statements about how young people may sometimes feel or react to certain situations. Think about each statement carefully and consider whether the statement applies to you almost always, most of the time, about half the time, sometimes, or almost never. Remember, there are no right or wrong answers.*
Item Code	Kweshon<br>Question
ER_1_C	Ar no sabi bot mi filin dem.<br>*I am not clear about my feelings.*
ER_2_C	Ar no day pay attenshon bot aw ar kin fil.<br>*I do not pay attention to how I feel.*
ER_3_C	Ar kin eksperens say mi filin dem pasmak en ar nor day abul kontrol dem.<br>*I experience my emotions as overwhelming and out of control.*
ER_4_C	Ar nor get no idia aw ar day fil.<br>*I have no idea how I am feeling*
ER_5_C	E kin tranga for ondastand bot mi filin dem.<br>*I have difficulty making sense out of my feelings.*
ER_6_C	Ar no kin abdul du somtin bot mi filin dem.<br>*I do not address my feelings.*
ER_7_C	Ar nor no gben aw ar day fil.<br>*I do not know exactly how I am feeling*
ER_8_C	Ar nor bisin bot waytin ar day fil.<br>*I do not care about what I am feeling.*
ER_9_C	Ar torment bot aw ar day fil.<br>*I am confused about how I feel.*
ER_10_C	Way ar fil bad, ar nor kin no bot filin dem.<br>*When I'm upset, I do not acknowledge my emotions.*
ER_11_C	Way ar fil bad, ar kin vex pa mi sef for way ar day fil da way day.<br>*When I'm upset, I become angry with myself for feeling that way (upset).*
ER_12_C	Way ar fil bad, ar kin shem for way ar kin fil da way.<br>*When I'm upset, I become embarrassed for feeling that way (upset).*
ER_13_C	Way ar fil bad, e kin tranga for du wok.<br>*When I'm upset, I have difficulty getting work done.*
ER_14_C	Way ar fil bad, ar day go awt of kontrol.<br>*When I'm upset, I become out of control.*
ER_15_C	Way ar fil bad, ar beliv say ar go day pa am for long tem.<br>*When I'm upset, I believe that I will remain that way for a long time.*

Boston College IRB
Approved
October 18, 2019
Through August 20, 2020

FOR APPROVALS WITH NO CR REQUIRED:

Boston College
IRB Approved
September 4, 2020-
September 3, 2021

ER_16_C	Way ar fil bad, ar beliv say ar kin end up mi filin dem wit poil at.<br>*When I'm upset, I believe that I will end up feeling very depressed.*
ER_17_C	Way ar fil bad, ar beliv say mi filin dem nor value en impotant.<br>*When I'm upset, I believe that my feelings are not valid and important.*
ER_18_C	Way ar fil bad, e kin at for mek ar put mi maynd pa oda tin dem.<br>*When I'm upset, I have difficulty focusing on other things.*
ER_19_C	Way ar fil bad, ar day los kontrol.<br>*When I'm upset, I feel out of control.*
ER_20_C	Way ar fil bad, ar nor day abul don for du somtin.<br>*When I'm upset, I cannot get things done.*
ER_21_C	Way ar fil bad, ar day fil shem pan mi sef for way ar day fil da way day.<br>*When I'm upset, I feel ashamed with myself for feeling that way.*
ER_22_C	Way ar fil bad, ar nor beliv say ar go fen way smol smol for mek ar fil beteh.<br>*When I'm upset, I don't believe that I can find a way to eventually feel better.*
ER_23_C	Way ar fil bad, ar kin fil lek ar wik.<br>*When I'm upset, I feel like I am weak.*
ER_24_C	Way ar fil bad, ar nor kin fil lek ar eabul kontrol di way aw ar day bihav.<br>*When I'm upset, I do not feel like I can remain in control of my behaviors.*
ER_25_C	Way ar fil bad, ar day fil gilti for di way ar day fil.<br>*When I'm upset, I feel guilty for feeling that way.*
ER_26_C	Way ar fil bad, e kin tranga for mek ar put mi maynd pan somtin.<br>*When I'm upset, I have difficulty concentrating.*
ER_27_C	Way ar fil bad, e kin tranga for mek ar kontrol di way ar day bihav.<br>*When I'm upset, I have difficulty controlling my behaviors.*
ER_28_C	Way ar fil bad, ar biliv say natin nor day way ar go du for misef way ar go fil beteh.<br>*When I'm upset, I believe there is nothing I can do to make myself feel better.*
ER_29_C	Way ar fil bad, ar day vex pan mi sef for way ar day fil da way day.<br>*When I'm upset, I become irritated with myself for feeling that way.*
ER_30_C	Way ar fil bad, ar kin bigin fil bad bot mi sef.<br>*When I'm upset, I start to feel very bad about myself.*
ER_31_C	Way ar fil bad, ar day tink bot am ol di tem.<br>*When I'm upset, I believe that wallowing in it is all I can do.*
ER_32_C	Way ar fil bad, ar day los kontrol of aw ar day bihav.<br>*When I'm upset, I lose control over my behaviors.*
ER_33_C	Way ar fil bad, e kin tranga for mek ar tink bot eni oda tin.<br>*When I'm upset, I have difficulty thinking about anything else.*
ER_34_C	Way ar fil bad, ar nor kin tek tem for no waytin ar day rili fil.<br>*When I'm upset, I do not take time to figure out what I'm really feeling.*
ER_35_C	Way ar fil bad, e kin tek long tem for mek ar fil beteh.<br>*When I'm upset, it takes me a long time to feel better.*
ER_36_C	Way ar fil bad, ar kin ova gladi.<br>*When I'm upset, my emotions feel overwhelming.*

Boston College IRB
Approved
October 18, 2019
Through August 20, 2020

FOR APPROVALS WITH NO CR REQUIRED:

Boston College
IRB Approved
September 4, 2020-
September 3, 2021

Boston College IRB Approved
October 18, 2019
Through August 20, 2020

FOR APPROVALS WITH NO CR REQUIRED:

Boston College IRB Approved
September 4, 2020-
September 3, 2021

SECTION 7: DEPRESSION & ANXIETY (HSCL_1_C - HSCL_25_C)
OW YU DAY DO

RESPONSE OPTIONS		
<<01>>	Natin-natin	*Not at all*
<<02>>	Smal	*A little*
<<03>>	Smal nor mor	*Quite a bit*
<<04>>	Boku boku wan	*Extremely*
<<998>>	Ar nor no	*Don't know*
<<999>>	Nor ansa	*No answer*

LABEL	Section 7: Di lis way day rite dong sai get diferen kayn wahala way pipul den kin get. Lisin fayn fayn to dem ol wan by wan en mekop aw dem bin day mona yu from las wik to tiday.<br><br>*Listed below are some symptoms or problems or worries that people sometimes have. Please listen to each one carefully and decide how much the problems bothered you in the last month, including today.*
LABEL	RAs: Use visual tool for this section
Item Code	Question
HSCL_1_C	From las mont to tiday, amos tem fraid fraid kin kam pan yu so nor mor for natin?<br>*From last month to today how much did you experience fear without cause?*
HSCL_2_C	From las mont to tiday, amos tem yu kin fil fraid?<br>*During the last month including today how much did you experience fear?*
HSCL_3_C	From las mont to tiday, amos tem yu fil taya bodi en yu hed de torn?<br>*During the last month including today how much did you experience weakness and your head turning?*
HSCL_4_C	From las mont to tiday, amos tem yu at day bit fas-fas?<br>*During the last month including today did how much has your heart been pounding?*
HSCL_5_C	From las mont to tiday, amos tem yu fil shake-shake na yu bodi?<br>*During the last month including today how much did you feel shaky inside your body?*
HSCL_6_C	From las mont to tiday, amos tem yu fil trimble trimble?<br>*During the last month including today how much did you tremble?*
HSCL_7_C	From las mont to tiday, amos tem yu hed kin at?<br>*During the last month including today how much did you have headaches?*
HSCL_8_C	From las mont to tiday, amos tem yu kin fraid nor mor en panik?<br>*During the last month including today how much did you get sudden feelings of fear and sudden panic?*
HSCL_9_C	From las mont to tiday, amos tem yu kin fil tense?<br>*During the last month including today how much did you feel tense?*
HSCL_10_C	From las mont to tiday, amos tem yu kin bet-bet?<br>*During the last month including today how much did you feel restless?*
HSCL_11_C	From las mont to tiday, amos tem yu nor kin get beteh trenk?<br>*During the last month including today how much did you not have enough energy/strength?*
HSCL_12_C	From las mont to tiday, amos tem yu kin blem yusef for tin dem way kin apin?<br>*During the last month including today how much did you blame yourself for things that occurred?*
HSCL_13_C	From las mont to tiday, amos tem yu kin day cry quik so nor mor?<br>*During the last month including today how much did you cry easily?*
HSCL_14_C	From las mont to tiday, amos tem yu don lef tin way yu bin lek for du way yu nor da ydu again?<br>*During the last month including today, how much did you stop doing things that you liked to do before that you do not do again?*
HSCL_15_C	From las mont to tiday, amos tem yu nor kin get apetite?<br>*During the last month including today, how much did you have poor appetite?*

Boston College IRB
Approved
October 18, 2019
Through August 20, 2020

FOR APPROVALS WITH NO CR REQUIRED:

Boston College
IRB Approved
September 4, 2020-
September 3, 2021

HSCL_16_C	From las mont to tiday, amos tem e kin at for leh yu slip en yu nor kin get fayn slip?<br>*During the last month including today, how much did you have trouble falling asleep and not getting good sleep?*	
HSCL_17_C	From las mont to tiday, amos tem yu fil se yu nor ab op again for yu tumara?<br>*During the last month including today, how much did you feel you have no hope again for tomorrow?*	
HSCL_18_C	From las mont to tiday, amos tem yu get poil at?<br>*During the last month including today, how much did you have sad heart?*	
HSCL_19_C	From las mont to tiday, amos tem yu fil for kip to yusef?<br>*During the last month including today, how much did you feel like keeeping to yourself?*	
HSCL_20_C	From las mont to tiday, amos tem yu memba se yu day don yu layf?<br>*During the last month including today, how much did you have thoughts of ending your life?*	Natin-natin ➔ WB_21_C <<00>><br>*Not at all*<br>Smal ➔ WB_21_C <<01>><br>*A little*<br>Smal nor mor ➔ WB_20A_C <<02>><br>*Quite a bit*<br>Boku boku wan ➔ WB_20A_C <<03>><br>*A lot*<br>Ar nor no ➔ WB_21_C <<998>><br>Nor ansa ➔ WB_21_C <<999>>
HSCL_20A_C	Stop day intaviu! kol day supavisor for leh e du di riks of ham safti plan. Yu kin kontinu di intaviu wans yu don wach en no if di posin wan for kil esef, di supavisor day na road, en yu don don di soshal kontrakt wit di patisipant, if nid day for am.<br>*Pause interview! Call supervisor to activate the risk of harm safety plan. You can continue the interview once you have assessed the suicidality of the participant, the supervisor is on the way, and you have completed a social contract with the participant, if needed.*	
HSCL_21_C	From las mont to tiday, amos tem yu kin fil tite insai yusef?<br>*During the last month including today, how much did you feel stuck?*	
HSCL_22_C	From las mont to tiday, amos tem yu kin worri-worri bot tin dem pas mak?<br>*During the last month including today, how much have you seriously been worried about things?*	
HSCL_23_C	From las mont to tiday, amos tem yu at nor kin get interes pan sometin?<br>*During the last month including today, how much did you lose interest in things?*	
HSCL_24_C	From las mont to tiday, amos tem yu fil se ol tin na wok way nor izi?<br>*During the last month including today how much did you feel that everything is difficult?*	
HSCL_25_C	From las mont to tiday, amos tem yu kin fil se yu na natin?<br>*During the last month including today how much did you have feelings of nothingness?*	

Boston College IRB Approved
October 18, 2019
Through August 20, 2020

FOR APPROVALS WITH NO CR REQUIRED:

Boston College
IRB Approved
September 4, 2020-
September 3, 2021

Boston College IRB Approved
October 18, 2019
Through August 20, 2020

FOR APPROVALS WITH NO CR REQUIRED:

Boston College
IRB Approved
September 4, 2020-
September 3, 2021

SECTION 8: FUNCTIONING (FNC_1_C – FNC_21_C)

Label	Section 17: Pan dem tin ya way ar day kam kol, duya pik di wan way day sho gben aw yu welbodi day TIDAY.<br>*Under each heading, please pick the ONE answer that best describes your health TODAY.*	
**Item Code**	**Questions**	**Coding Responses**
FNC_1_C	For waka<br>*Mobility*	A nor get wan problem for waka <<00>><br>*I have no problems walking*<br>A get smal smal problem for waka <<01>><br>*I have slight problems walking*<br>A get smal problem for waka <<02>><br>*I have moderate problems walking*<br>A get sirios problem for waka <<03>><br>*I have severe problems walking*<br>A nor ebul waka <<04>><br>*I am unable to walk*
FNC_2_C	For tek kiya of yusef<br>*Self-care*	A nor get wan problem for was or dres misef <<00>><br>*I have no problems washing or dressing myself*<br>A get smal smal problem for was or dres <<01>><br>*I have slight problems washing or dressing myself*<br>A get smal problem for was or dres <<02>><br>*I have moderate problems washing or dressing myself*<br>A get sirios problem for was or dres <<03>><br>*I have severe problems washing or dressing myself*<br>A nor ebul for was or dres <<04>><br>*I am unable to wash or dress myself*
FNC_3_C	Tin dem way yu yus for du (lekeh: wok, buk lanin, os wok, fambul or gladi gladi tin dem)<br>*Usual activities (e.g. work, study, housework, family or leisure activities)*	A nor get wan problem for du di tin dem way a yus for du <<00>><br>*I have no problems doing my usual activities*<br>A get smal smal problem for du di tin dem way a yus for du<<01>><br>*I have slight problems*<br>A get smal problem for du di tin dem way a yus for du <<02>><br>*I have moderate problems*<br>A get sirios problem for du di tin dem way a yus for du <<03>><br>*I have severe problems*<br>A nor ebul for du di tin dem way a yus for du <<04>><br>*I am unable to do my usual activities*
FNC_4_C	Pain / somtin way day mona yu<br>*Pain/Discomfort*	A nor get wan pain or somtin way day mona mi <<00>><br>*I have no pain or discomfort*<br>A get smal smal pain or somtin way day mona mi <<01>><br>*I have slight pain or discomfort*<br>A get smal pain or somtin way day mona mi <<02>><br>*I have moderate pain or discomfort*<br>A get sirios pain or sirios tin way day mona mi <<03>><br>*I have severe pain or discomfort*<br>A get bad bad pain or bad bad tin way day mona mi <<04>><br>*I have extreme pain or discomfort*
FNC_5_C	Wondri en poil at<br>*Anxiety/Depression*	A nor day wondri or get wan poil at <<00>><br>*I am not anxious or depressed*<br>A day wondri smal smal wan or get smal poil at <<01>><br>*I am slightly anxious or depressed*<br>A day wondri smal or get small poil at <<02>><br>*I am moderately anxious or depressed*<br>A day wondri sirios wan or get get sirios poil at <<03>><br>*I am severely anxious or depressed*<br>A day wondri bad bad wan or get da bad bad poil at <<04>><br>*I am extremely anxious or depressed*

Boston College IRB Approved
October 18, 2019
Through August 20, 2020

FOR APPROVALS WITH NO CR REQUIRED:

Boston College
IRB Approved
September 4, 2020-
September 3, 2021

FNC_6_C	Wi go lek for no aw gud or bad yu welbodi wan day tiday.<br><br>*[GI AM KAD EN MAKA]*<br><br>Dis skale den nomba ram from 0 to 100- 100 minsay di best welbodi way yu kin tink bot, 0 min se di worst welbodi way yu kin tink bot. Duya mak x na di skale for sho aw yu welbodi day tiday.<br><br>*We would like to know how good or bad your health is today. (GIVE card and marker to participant) This scale is numbered from 0 to 100 – 100 means the best health you can imagine, 0 means the worst health you can imagine. Please mark an X on the scale to indicate how your health is TODAY.*	Yu welbodi wan tiday:<br>*Your health today:*<br><br>______________________<br>*[YU WAY DAY AKS KWESHON RAIT DI EGZAKT NOMBA WAY DI SIK MAN SHO]*<br><br>[Number item]
LABEL	Dis nex pat na bot problem dem way pipul den kin get bikoz of den nor get welbodi. Way a se den nor get welbodi, a min for get sik or nor day fil wel, or oda welbodi problem dem way no kin tae beteh, en di wan dem way kin tae pa posin, lek for wund/koz pain, way posin day mek lek e hed nor day, or yu get somtin way day wori yu maynd/chest pain, en rom or drugs problem. Memba al yu welbodi problem dem as yu day ansa den kweshon ya. Way a aks yu bot problem dem way yu day du somtin, duya tink bot for: add mor trenk/effort; toment or pain; way yu day du tin saful saful; aw yu don chanj in di way dem way yu day du som tin dem. A want yu bak way yu day ansa dem kweshon ya for tink bot amos problem yu bin get, dat way yu gess, insai di las 30 dayz, way yu bin day du den tin ya aw yu blant du am.<br><br>*This next part is about difficulties people have because of health conditions. By health condition I mean diseases or illnesses, or other health problems that may be short or long lasting; injuries; mental or emotional problems; and problems with alcohol or drugs. Remember to keep all your health problems in mind as you answer the questions. When I ask you about difficulties in doing an activity think about… Increased effort; Discomfort or pain; Slowness; Changes in the way you do the activity. When answering, I'd like you to think back over the last 30 days. I would also like you to answer these questions thinking about how much difficulty you have had, on average over the past month, while doing the activity as you usually do it.*	
Item Code	Questions	Coding Responses
FNC_7_C	Insai di pas 30 dayz, omos tranganes yu get tinap for long tem, lek 30 minits?<br>*In the past 30 days, how much difficulty did you have sstanding for long periods such as 30 minutes?*	No at wan <<00>><br>*None*<br>At smol <<01>><br>*Mild*<br>Nor tu at <<02>><br>*Moderate*<br>Pasmak wan <<03>><br>*Severe*<br>Pasmak pasmak wan or a nor kin ebul do am <<04>><br>*Extreme or can't do*<br>Ar no no <<998>><br>*Don't know*<br>Nor ansa <<999>><br>*No answer*
FNC_8_C	Insai di pas 30 dayz, omos tranganes yu bin get for du yu ose wok dem?<br>*In the past 30 days, how much difficulty did you have taking care of your household responsibilities?*	No at wan <<00>><br>At smol <<01>><br>Nor tu at <<02>><br>Pasmak wan <<03>><br>Pasmak pasmak wan or a nor kin ebul do am <<04>><br>Ar no no <<998>><br>Nor ansa <<999>>

Boston College IRB Approved
October 18, 2019
Through August 20, 2020

FOR APPROVALS WITH NO CR REQUIRED:

Boston College
IRB Approved
September 4, 2020-
September 3, 2021

FNC_9_C	Insai di pas 30 dayz, omos tranganes yu bin get for lan niu wok, for egazampul, lan aw for go na niu ples/som sai?<br>*In the past 30 days, how much difficulty did you have learning a new task, for example, learning how to get to a new place?*	No at wan <<00>><br>At smol <<01>><br>Nor tu at <<02>><br>Pasmak wan <<03>><br>Pasmak pasmak wan or a nor kin ebul do am <<04>><br>Ar no no <<998>><br>Nor ansa <<999>>
FNC_10_C	Insai di pas 30 dayz, aw at e bin bi for yu for mek yu join/ tek pat pan community bizness (lek pan gladi gladi bizness, God biznes or oda tin dem) jes di sem way lek aw oda pipul den kin du?<br>*In the past 30 dez, how much of a problem did you have joining in community activities (for example, festivities, religious or other activities) in the same way as anyone else can?*	No at wan <<00>><br>At smol <<01>><br>Nor tu at <<02>><br>Pasmak wan <<03>><br>Pasmak pasmak wan or a nor kin ebul do am <<04>><br>Ar no no <<998>><br>Nor ansa <<999>>
FNC_11_C	Insai di pas 30 dayz, aw yu welbodi problem don wori yu at/maynd?<br>*In the past 30 days, how much have you been emotionally affected by your health problems?*	No at wan <<00>><br>At smol <<01>><br>Nor tu at <<02>><br>Pasmak wan <<03>><br>Pasmak pasmak wan or a nor kin ebul do am <<04>><br>Ar no no <<998>><br>Nor ansa <<999>>
FNC_12_C	Insai di pas 30 dayz, omos tem yu get tranganes for put yu maynd don for du somtin for 10 minits?<br>*In the 30 days, how much difficulty did you have concentrating on doing something for ten minutes?*	No at wan <<00>><br>At smol <<01>><br>Nor tu at <<02>><br>Pasmak wan <<03>><br>Pasmak pasmak wan or a nor kin ebul do am <<04>><br>Ar no no <<998>><br>Nor ansa <<999>>
FNC_13_C	Insai di pas 30 dayz, omos transganes yu bin get for waka go far ples, lek wan mile or sai way fa so?<br>*In the past 30 days, how much difficulty did you have walking a long distance such as a kilometre [or equivalent]?*	No at wan <<00>><br>At smol <<01>><br>Nor tu at <<02>><br>Pasmak wan <<03>><br>Pasmak pasmak wan or a nor kin ebul do am <<04>><br>Ar no no <<998>><br>Nor ansa <<999>>
FNC_14_C	Insai di pas 30 dayz, omos tranganes yu bin get for was yu ol bodi?<br>*In the past 30 days, how much difficulty did you have washing your whole body?*	No at wan <<00>><br>At smol <<01>><br>Nor tu at <<02>><br>Pasmak wan <<03>><br>Pasmak pasmak wan or a nor kin ebul do am <<04>><br>Ar no no <<998>><br>Nor ansa <<999>>

Boston College IRB Approved
October 18, 2019
Through August 20, 2020

FOR APPROVALS WITH NO CR REQUIRED:

Boston College
IRB Approved
September 4, 2020-
September 3, 2021

FNC_15_C	Insai di pas 30 dayz, omos transganes yu bin get for dres (for put yusef togeda)?<br>*In the past 30 days, how much difficulty did you have getting dressed?*	No at wan <<00>><br>At smol <<01>><br>Nor tu at <<02>><br>Pasmak wan <<03>><br>Pasmak pasmak wan or a nor kin ebul do am <<04>><br>Ar no no <<998>><br>Nor ansa <<999>>
FNC_16_C	Insai di pas 30 dayz, omos tranganes yu bin get for dil wit pipul dem way yu nor sabi?<br>*In the past 30 days, how much difficulty did you have dealing with people you do not know?*	No at wan <<00>><br>At smol <<01>><br>Nor tu at <<02>><br>Pasmak wan <<03>><br>Pasmak pasmak wan or a nor kin ebul do am <<04>><br>Ar no no <<998>><br>Nor ansa <<999>>
FNC_17_C	Insai di pas 30 dayz, omos tranganes yu bin get for kip padi biznes?<br>*In the past 30 days, how much difficulty did you have maintaining a friendship?*	No at wan <<00>><br>At smol <<01>><br>Nor tu at <<02>><br>Pasmak wan <<03>><br>Pasmak pasmak wan or a nor kin ebul do am <<04>><br>Ar no no <<998>><br>Nor ansa <<999>>
FNC_18_C	Insai di pas 30 dayz, omos tranganes yu bin bi get for du yu evriday wok/ skul wok?<br>*In the past 30 days, how much difficulty did you have in your day-to-day work or other activities?*	No at wan <<00>><br>At smol <<01>><br>Nor tu at <<02>><br>Pasmak wan <<03>><br>Pasmak pasmak wan or a nor kin ebul do am <<04>><br>Ar no no <<998>><br>Nor ansa <<999>>
FNC_19_C	Al dem problem ya wi don tok bot, a day kam ask yu kweshon den bot dem. Way yu chek am al, insai di pas 30 dayz, amos dezdem way dem tranganes ya bin day?<br>*Overall, in the past month, how many days were these difficulties present?*	Gee di exact:<br>➔___________(Days)
FNC_20_C	Insai di pas 30 dayz, for amos dayz way yu nor bin ebul for du di evriday tin dem or wok bikoz of dem tranganes ya bin day?<br>*In the past month, for how many days were you totally unable to carry out your usual activities or work because of these problems?*	Gee di exact:<br>➔___________(Days)
FNC_21_C	Insai di pas 30 dayz, apat from di day dem way yu nor bin ebul du anitin, for amos dez dem way yu bin ridyus pan di tin dem/wok dem way yu kin du, bikus of dem tranganes ya bin day?<br>*In the past month, not counting the days that you were totally unable, for how many days did you cut back or reduce your usual activities or work because of these problems?*	Gee di exact:<br>➔___________(Days)

Boston College IRB Approved
October 18, 2019
Through August 20, 2020

FOR APPROVALS WITH NO CR REQUIRED:

Boston College
IRB Approved
September 4, 2020-
September 3, 2021

Boston College IRB
Approved
October 18, 2019
Through August 20, 2020

FOR APPROVALS WITH NO CR REQUIRED:

Boston College
IRB Approved
September 4, 2020-
September 3, 2021

SECTION 9: POST-TRAUMATIC STRESS DISORDER (PTSD_1_C – PTSD_17_C)

RESPONSE OPTIONS		
<<01>>	Nor	*No*
<<02>>	Yes	*Yes*

LABEL	Naw ar day kam aks yu som kweshon dem bot som tin dem way morna yu pasmak way don mit yu, yu bin day usai e apin, or yu bin fasin way mek yu fred pasmak, yu nor abul ep yu sef, or fiaful tin. If yu abul, yu<br><br>*Now I am going to ask you some questions about the most distressing event that you have experienced, witnessed, or were confronted with which caused intense fear, helplessness, or horror. If you feel able, can you tell me what that event was? Specify:*
PTSD_1_C	Di bad bad tin dem way yu don tok bot, na tin way apin pan di war?<br>*Was the distressing event you described above war-related?*
Label	Di statements den refer to tranga tin den way mit yu from way day war don. Duya ansah "YES" if e mit yu durin day 3 MONT way don pa.<br>*The following statements refer to difficulties you may have experienced since the end of the war. Please answer "YES" if you experienced the event described during the PAST THREE MONTHS.*
PTSD_2_C	Yu day memba oltem bot tin dem way day mek you fil bad or you day drim bot dem bad tin dem?<br>*Have you had recurrent or intrusive distressing thoughts or recollections about the trauma?*
PTSD_3_C	Yu bin don day get bad drim dem oltem, bot di bad tin dem way bin don apin?<br>*Have you been having recurrent bad dreams or nightmares about the trauma?*
PTSD_4_C	Yu maynd bin don toment pasmak wan way somtin mek yu memba tin dem way bad way bin don apin to yu? Evin lekway di patikula day rich bak way di tin apin?<br>*Have you been intensely EMOTIONALLY upset when reminded of the trauma includes anniversary reactions)?*
PTSD_5_C	Yu bin don day get tin dem way day mek yu bodi cheng kwiklek if yu day swet pasmak, you at day bit fas fas or oda tin way kin apin to yu. mor lek way somtin mek yu memba den bad tin dem?<br>*Have you been having intense PHYSICAL reactions like sweaty, heart palpitations or other things that happen when reminded of the trauma?*
PTSD_6_C	Yu bin don day tri oltem for mek yu nor memba or get enitin for du wit den bad tin dem way bin don apin to yu?<br>*Have you persistently been making efforts to avoid thoughts or feelings associated with the trauma?*
PTSD_7_C	Yu bin don day tri oltem for mek yu avoid tin dem way yu or oda pipul dem day du, way bin don apin or sai dem way day mek yu memba bot tin dem way bin don mona yu pasmak?<br>*Have you persistently been making efforts to avoid activities, situations, or places that remind you of the trauma?*
PTSD_8_C	Eni impotant tin/sai day pan den bad tin dem way apin way yu nor abul memba?<br>*Are there any important aspects about the trauma that you still cannot recall?*
PTSD_9_C	Yu nor lek egen for joke or du tin dem from way den bad tin ya apin to yu?<br>*Have you markedly lost interest in free time activities since the trauma?*
PTSD_10_C	Yu fil say yu nor day nia pipul dem way yu sabi, or push far from way den bad tin ya apin to yu?<br>*Have you felt detached or cut off from others around you since the trauma?*
PTSD_11_C	Yu bin don day fil say for mek yu abul sho filin don ambog lek yu norabul sho leknes to oda pipul dem?<br>*Have you felt that your ability to experience the whole range of emotions is impaired such as (i.e. unable to have loving feelings)?*
PTSD_12_C	Yu fil say eni plan way yu bin don mek for di bambai or tin way yu don abop pa don cheng bikos of di bad tin dem way apin to yu? Lek yu nor get wok, yu nor marade, nor get piking or long layf?<br>*Have you felt that any future plans or hopes have changed because of the trauma for example no career, marriage, children, or long life?*
PTSD_13_C	E tranga for mek yu slip or for mek yu tay pan slip?<br>*Have you had persistent difficulty falling or staying asleep?*

Boston College IRB Approved October 18, 2019 Through August 20, 2020

Boston College IRB Approved September 4, 2020- September 3, 2021

PTSD_14_C	Yu bin don day oltem vex pasmak kwik wan?<br>*Have you been continuously irritable or have outbursts of anger?*
PTSD_15_C	E kin mona for mek yu put yu atenshon pan somtin?<br>*Have you had persistent difficulty concentrating?*
PTSD_16_C	Yu olways day expet say somtin day apin to from way di tem way den bad tin dem apin to yu? For eksampul lek yu day luk for si udat day nia yu?<br>*Are you overly alert for example check to see who is around you since the trauma?*
PTSD_17_C	Yu kin skiad kwik wan pan yusef en mek lek yu nor day wit yusef, from di tem way den bad tin dem apin to yu?*Have you been jumpier, more easily startled, since the trauma?*

Boston College IRB
Approved
October 18, 2019
Through August 20, 2020

FOR APPROVALS WITH NO CR REQUIRED:

Boston College
IRB Approved
September 4, 2020-
September 3, 2021

SECTION 10: CHILD BEHAVIOR (OMCI_1_C – OMCI_19_C)

RESPONSE OPTIONS
<<01>> Nor *No*
<<02>> Yes *Yes*

Label	KRIO TRANLSATION HERE<br><br>Instructions for Caregiver:<br>I want to watch you play and talk with {child_name} using a picture book, a ball or any kind of toy as you would do normally. You may choose one of these picture books. I would observe you for 5 minutes. When I say stop, you can discontinue.<br><br>Instructions for Interviewer:<br>Observe the mother and child for 5 minutes using your stop watches. Observe behaviours and mark on scale with a tick. Stop timing and coding after 5 minutes. Gently stop mother and child at a convenient point in their interaction. If the mother stops too early (< 4 min), ask her to continue, otherwise end it.<br><br>Neither at the start of the observation nor during the observation provides the mother with instructions or guidelines about how to interact with her child. Let mother continue as usual and make your observations without disturbing or guiding the interaction.<br><br>After checking the observations are all marked on the scale, calculate the total score achieved. The value of each rating must be added together based on the scoring instructions at the end of the observation.
Item Code	Kweshon<br>Question
OMCI_1_C	*Caregiver shows positive affect for the child.*
OMCI_2_C	*Caregiver shows negative affect for the child.*
OMCI_3_C	*Caregiver shows positive touch.*
OMCI_4_C	*Caregiver shows negative touch.*
OMCI_5_C	*Caregiver expresses positive verbal statements.*
OMCI_6_C	*Caregiver expresses negative verbal statements.*
OMCI_7_C	*Caregiver is sensitive of the child's needs.*
OMCI_8_C	*Caregiver expands on child's talking.*
OMCI_9_C	*Caregiver points and names the toy or object in the book.*

Boston College IRB
Approved
October 18, 2019
Through August 20, 2020

Boston College
IRB Approved
September 4, 2020-
September 3, 2021

OMCI_10_C	*Caregiver questions child.*
OMCI_11_C	*Caregiver answers child's question or request.*
OMCI_12_C	*Caregiver helps child to maintain interest.*

OMCI_13_C	*Child smiles, laughs at caregiver.*
OMCI_14_C	*Child shows excitement and enjoyment, like clapping.*
OMCI_15_C	*Child is crying, frowning, frustrated.*
OMCI_16_C	*Child remains focused on activity for a significant time (for at least 1 minute).*
OMCI_17_C	*Child continues in spite of distractions.*
OMCI_18_C	*Child is vocalizing/making sounds or producing words for the purpose of communicating*.
OMCI_19_C	*Caregiver & child express enjoyment while exploring together*.

Boston College IRB Approved
October 18, 2019
Through August 20, 2020

FOR APPROVALS WITH NO CR REQUIRED:

Boston College IRB Approved
September 4, 2020-
September 3, 2021

SECTION 11: HOME ENVIRONMENT (OMCI_1_C – OMCI_43_C)

RESPONSE OPTIONS		
<<01>>	Nor	*No*
<<02>>	Yes	*Yes*

Label	KRIO TRANLSATION HERE<br><br>Instructions for Interviewer:<br>The interview should be relaxed, non-judgmental and friendly. Talk to the mother while observing mother- child interactions. Observe items when you can; ask question for items that you cannot observe. Child should be in the same place doing his/her usual activities.
Item Code	Kweshon<br>Question
HOME_1_C	*Do you receive guests at home or visit family? Neighbors or friends who visit informally or drop-by and stay long enough to at least sit down or share a drink/conversation count here.*
HOME_2_C	*Does your child usually eat supper or any meal with the whole family? It's enough to just be sitting together at the same time, this does not mean you are all sharing the same food.*
HOME_3_C	*Does the father of the child spend some time every day caring for the child? For example talking, walking, and/or playing with the child?*
HOME_4_C	*When you are away, how many other people/caregivers are available to look after the child?*
HOME_5_C	*Does your child have regular playmates that are around his/her same age? By "around" we mean within 1 year older or younger.*
HOME_6_C	*How often does your child go to the market with you or another member of the household?*
HOME_7_C	*How often does your child go out of the house/yard?*
HOME_8_C	*How often does a caregiver take your child far from home?*
HOME_9_C	*When you are busy with housework, do you try to engage your child in what you are doing (e.g., talking with him/her about what you are doing or asking questions about what you are doing)?*
HOME_10_C	*Did you show or teach your child something new this past week, like teach a new word, or help child do something difficult?*
LABEL	*Please show me what your child typically plays with on his/her own.*
HOME_11_C	*Gross motor objects available (e.g., ball, rope, ring, stone).*
HOME_12_C	*Push or pull toys available (e.g., pull with string, push box).*

Boston College IRB Approved
October 18, 2019
Through August 20, 2020

FOR APPROVALS WITH NO CR REQUIRED:

Boston College
IRB Approved
September 4, 2020-
September 3, 2021

HOME_13_C	*Cuddly toys available?*
HOME_14_C	*Simple eye-hand coordination materials available (e.g. single object that rattles, shakes, squeezes).*
HOME_15_C	*Complex eye-hand coordination materials available (e.g., different shaped blocks, pencil & paper).*
HOME_16_C	*Book or item that has both pictures and words together (not textbook, can be collection of pictures).*
HOME_17_C	*At least 1 adult book or magazine is observed in the home (includes religious books but not textbooks).*
HOME_18_C	*Do you provide materials or activities that are slightly difficult for your child?*
HOME_19_C	*Does your child have access to children's books?*
HOME_20_C	*Are there any objects that the child plays with to create a story, play pretend, or imitate actions he/she sees in the home/community?*
HOME_21_C	*Does your child play any structured games with people (e.g., circle games, clapping/singing games)?*
HOME_22_C	*Does your child know where to find his/her playthings (e.g, bag, box)?*
HOME_23_C	*Have you ever found/made/ gotten something new for your child to play with?*
HOME_23a_C	*When did you get the newest toy/object?*
HOME_24_C	*In the past week, did you look or someone in the household look at pictures in a book, calendar or magazine with the child?*
HOME_25_C	*How often do you tell stories or sing songs with your child?*
LABEL	*PROMPT: At a natural point during the session, offer some praise of the child (e.g., "You did that so well!", "She can run so fast!", etc.). Make certain that your praise is genuine - do not try to force praise. Score this item "Yes" if the mother expresses happiness, pride, agreement, gratitude, etc. in response, either verbally or with facial expressions. However, if the mother says little more than "Thank you" without feeling or with an embarrassed smile, praise the child again later to see if he/she responds with more emotion. If you consistently get a response that shows little feeling or pleasure, score this item "No".*
HOME_26_C	*After visitor praises child, mother responds positively (e.g. mother nods, smiles, thanks, agrees).*
HOME_27_C	*The mother spontaneously talks to the child.*
HOME_28_C	*The mother responds verbally to child's talk or gestures (does not ignore the child; do not include scolding or shouting).*

Boston College IRB Approved
October 18, 2019
Through August 20, 2020

FOR APPROVALS WITH NO CR REQUIRED:

Boston College
IRB Approved
September 4, 2020-
September 3, 2021

HOME_29_C	*Mother tells child the name of an object or person during visit.*
HOME_30_C	*Mother's speech is distinct, clear, and audible.*
HOME_31_C	*Mother talks well and freely to the interviewer.*
HOME_32_C	*Mother permits child to play freely (includes messy, noisy play).*
HOME_33a_C	*Mother spontaneously praises child verbally without prompt at least twice. FIRST INSTANCE*
HOME_33b_C	*Mother spontaneously praises child verbally without prompt at least twice. SECOND INSTANCE*
HOME_34_C	*Mother conveys positive feelings towards child. For example: smiling, tone of voice, or using positive words*
HOME_35_C	*Mother caresses, strokes head, or kisses the child.*<br><br>*Score any hug, kiss or cuddle as "Yes". Watch closely for subtle caresses or other affectionate gestures, such as holding a hand, stroking the face or hair (not fixing/cleaning it), patting the child gently, etc. Score either obvious or subtle caresses as "Yes". Simply touching a child does not necessarily count as a caress if it is not affectionate. (No <0>, = 0 times Yes <1> = 1st time)*
HOME_36_C	*Mother makes effort to provide child with a play object during visit*<br><br>*Score this item "Yes" if the mother offers the child something to play with and examine, whether it is a toy or any household object that the child seems interested in. The child's reaction (accepting or rejecting the object) does not affect the score. (No <0>, = 0 times Yes <1> = 1st time)*
HOME_37_C	*Mother shouts at child during the visit (except for corrective shouting to stop a child from doing something dangerous).*
HOME_38_C	*Mother complains about child or says child is bad.*
HOME_39_C	*Mother hits, pushes, shakes child during visit.*
HOME_40_C	*Mother threatens the child with punishment during visit.*<br><br>*Shyiraho ''Yego ''niba Mama w'umwana amuhutaza .Niba ubona bitagaragara ko umubyeyi ari gukina n'umwana cg ari kubabaza umwana ,reba igisubizo cy'umwana .Ni ba umwana asubizanije umunezero cg ibyishimo ,ibi akenshi bigaragazo uburyo bwo ''gukirana '' mu mimikino hagati ye na mama we . Niba umwana agaragaye nk'utishimye ,atongana,ahakana,cg arira ,uko biri kose shyira ho '' Yego ''*
HOME_41a_C	*Mother restricts or interferes with child's activity during visit three or more times. FIRST INSTANCE*
HOME_41b_C	*Mother restricts or interferes with child's activity during visit three or more times. SECOND INSTANCE*

Boston College IRB Approved
October 18, 2019
Through August 20, 2020

FOR APPROVALS WITH NO CR REQUIRED:

Boston College
IRB Approved
September 4, 2020-
September 3, 2021

HOEM_41c_C	*Mother restricts or interferes with child's activity during visit three or more times. THIRD INSTANCE*
HOME_42_C	*Observe whether indoor/outdoor area is safe for play.*
HOME_43_C	*Mother keeps child within eyesight, looks at him/her often.*

SECTION 16: CLOSING?

**LABEL**	Tenki, we almos don don.<br>*Thank you. We are almost finished.*
**END_1_C**	Aw e tan lek for mek a aks yu dem kweshion dem ya?<br>*What was it like for you to take this survey?*
**END_2_C**	Enitin day way yu wan for add way add a nor aks yu bot yet?<br>*Is there anything you would like to add that I didn't ask you about?*
**LABEL**	END: Tenki for yu tem way yu get fo mi! Tenki ya!<br>*Thanks for your time that you gave to me. Thanks!*

Boston College IRB Approved
October 18, 2019
Through August 20, 2020

FOR APPROVALS WITH NO CR REQUIRED:

Boston College
IRB Approved
September 4, 2020-
September 3, 2021

Survey - Fidelity Monitoring Guide				
**Question Number**	**Question (English)**	**Question (Krio)**	**Response Options (English)**	**Response Options (Krio)**
1	Today's date:			
2	CHW ID Code:			
3	Household ID code:			
4	Cell Mentor ID code:			
5	Module number:			
6	Date of Shadowing Visit:			
7	Duration of module:			
8	Caregivers present: Select all that apply		☐ Mother<br>☐ Father<br>☐ Grandmother<br>☐ Grandfather<br>☐ Older Children<br>☐ Another Adult in the home<br>☐ Other___________________	
9	Did the child participate in the visit?		0) No<br>1) Yes<br><br>If No, why?<br>1) Away<br>2) Sick<br>3) Sleeping<br>4) Other	
**Rating Guide:**<br>**Did not Occur-**<br>**Poor-**<br>**Needs Improvement-**<br>**Average -**<br>**Excellent -**				

Boston College IRB Approved
October 18, 2019
Through August 20, 2020

FOR APPROVALS WITH NO CR REQUIRED:

Boston College IRB Approved
September 4, 2020-
September 3, 2021

10	The CHW greeted the family and asked how the family has been since the last meeting.		How well was it executed by the CHW?<br>0- Did not Occur<br>1 - Poor<br>2- Needs Improvement<br>3- Average<br>4- Excellent	0-
11	The CHW reviewed material from the previous session with the family		How well was it executed by the CHW?<br>0- Did not Occur<br>1 - Poor<br>2- Needs Improvement<br>3- Average<br>4- Excellent	0-
12	The CHW asked family what activities and skills they have practiced with their child or in their household since the last session.		How well was it executed by the CHW?<br>0- Did not Occur<br>1 - Poor<br>2- Needs Improvement<br>3- Average<br>4- Excellent	

Boston College IRB Approved
October 18, 2019
Through August 20, 2020

FOR APPROVALS WITH NO CR REQUIRED:

Boston College IRB Approved
September 4, 2020-
September 3, 2021

13	CHW asked the caregivers if there were any issues when attempting the new activities or skills.		How well was it executed by the CHW?<br>0- Did not Occur<br>1 - Poor<br>2- Needs Improvement<br>3- Average<br>4- Excellent	
14	The CHW shared knowledge from the module with the caregivers (related to the intervention)		How well was it executed by the CHW?<br>0- Did not Occur<br>1 - Poor<br>2- Needs Improvement<br>3- Average<br>4- Excellent	

Boston College IRB Approved
October 18, 2019
Through August 20, 2020

FOR APPROVALS WITH NO CR REQUIRED:

Boston College IRB Approved
September 4, 2020-
September 3, 2021

15	The CHW engaged the family in a discussion about the module topic(s) and encouraged participation from all caregivers		How well was it executed by the CHW?<br>0- Did not Occur<br>1 - Poor<br>2- Needs Improvement<br>3- Average<br>4- Excellent	
16	The CHW used suggestions and reminders to encourage the caregivers.		How well was it executed by the CHW?<br>0- Did not Occur<br>1 - Poor<br>2- Needs Improvement<br>3- Average<br>4- Excellent	

Boston College IRB Approved
October 18, 2019
Through August 20, 2020

FOR APPROVALS WITH NO CR REQUIRED:

Boston College IRB Approved
September 4, 2020-
September 3, 2021

17	**For the 15-minute active play session:** The CHW allowed both caregivers to independently practice the activity with the child.		How well was it executed by the CHW?<br>0- Did not Occur<br>1 - Poor<br>2- Needs Improvement<br>3- Average<br>4- Excellent	
18	**For the 15-minute active play session:** The CHW followed active coaching guidelines when coaching the caregivers during the active play session.		How well was it executed by the CHW?<br>0- Did not Occur<br>1 - Poor<br>2- Needs Improvement<br>3- Average<br>4- Excellent	

Boston College IRB Approved
October 18, 2019
Through August 20, 2020

FOR APPROVALS WITH NO CR REQUIRED:

Boston College IRB Approved
September 4, 2020-
September 3, 2021

19	CHW asked caregivers which information is new or most interesting to them.		How well was it executed by the CHW?<br>0- Did not Occur<br>1 - Poor<br>2- Needs Improvement<br>3- Average<br>4- Excellent	
20	The CHW gave good feedback to the caregivers (no more than 2-3 suggestions at a time).		How well was it executed by the CHW?<br>0- Did not Occur<br>1 - Poor<br>2- Needs Improvement<br>3- Average<br>4- Excellent	

Boston College IRB Approved
October 18, 2019
Through August 20, 2020

FOR APPROVALS WITH NO CR REQUIRED:

Boston College IRB Approved
September 4, 2020-
September 3, 2021

21	The CHW and caregivers discussed what new skills and activities the caregiver would adopt between now and the next session.		How well was it executed by the CHW?<br>0- Did not Occur<br>1 - Poor<br>2- Needs Improvement<br>3- Average<br>4- Excellent	
22	The CHW checked out with the family regarding what they liked most about the session and addressed any concerns.		How well was it executed by the CHW?<br>0- Did not Occur<br>1 - Poor<br>2- Needs Improvement<br>3- Average<br>4- Excellent	0-

Boston College IRB Approved
October 18, 2019
Through August 20, 2020

FOR APPROVALS WITH NO CR REQUIRED:

Boston College IRB Approved
September 4, 2020-
September 3, 2021

23	The CHW praised the caregivers for participating.		How well was it executed by the CHW?<br>0- Did not Occur<br>1 - Poor<br>2- Needs Improvement<br>3- Average<br>4- Excellent	
24	The CHW problem solved issues with the family.		How well was it executed by the CHW?<br>0- Did not Occur<br>1 - Poor<br>2- Needs Improvement<br>3- Average<br>4- Excellent	

Boston College IRB Approved
October 18, 2019
Through August 20, 2020

FOR APPROVALS WITH NO CR REQUIRED:

Boston College IRB Approved
September 4, 2020-
September 3, 2021

25	The CHW was well prepared for the session.		How well was it executed by the CHW?<br>1 - Poor<br>2- Needs Improvement<br>3- Average<br>4- Excellent	
26	The CHW answered the caregivers' questions appropriately.		How well was it executed by the CHW?<br>1 - Poor<br>2- Needs Improvement<br>3- Average<br>4- Excellent	

Boston College IRB Approved
October 18, 2019
Through August 20, 2020

FOR APPROVALS WITH NO CR REQUIRED:

Boston College IRB Approved
September 4, 2020-
September 3, 2021

27	The CHW appropriately used the Sugira Muryango manual, images and vignettes to support session delivery.		How well was it executed by the CHW?<br>1 - Poor<br>2- Needs Improvement<br>3- Average<br>4- Excellent	
28	The CHW was able to employ flexibility and creativity when delivering the intervention.		How well was it executed by the CHW?<br>1 - Poor<br>2- Needs Improvement<br>3- Average<br>4- Excellent	

Boston College IRB Approved
October 18, 2019
Through August 20, 2020

FOR APPROVALS WITH NO CR REQUIRED:

Boston College IRB Approved
September 4, 2020-
September 3, 2021

29	CHW communicates and demonstrates empathy and warmth with the family.		How well was it executed by the CHW?<br>1 - Poor<br>2- Needs Improvement<br>3- Average<br>4- Excellent	
30	The CHW established a good rapport with the family.		How well was it executed by the CHW?<br>1 - Poor<br>2- Needs Improvement<br>3- Average<br>4- Excellent	

Boston College IRB Approved
October 18, 2019
Through August 20, 2020

FOR APPROVALS WITH NO CR REQUIRED:

Boston College IRB Approved
September 4, 2020-
September 3, 2021

31	Overall, how well did the CHW deliver the intervention as intended?		How well was it executed by the CHW?<br>1 - Poor<br>2- Needs Improvement<br>3- Average<br>4- Excellent	
32	Use the space below to note any comments to guide supervision with the CHW. Focus on any areas where the CHW scored a 1 (poor) or 2 (needs improvement):			

Boston College IRB Approved
October 18, 2019
Through August 20, 2020

FOR APPROVALS WITH NO CR REQUIRED:

Boston College IRB Approved
September 4, 2020-
September 3, 2021

IMPLEMENTATION MEASURES Questionnaire: Provider

Respondent ID #	
Today's date	

Let's start the interview by asking you some basic questions about yourself

1. Demographic Characters

1.1. What is your gender?	0<br>Male		1<br>Female		
1.2. What is your age in years?					
1.3. What is the highest level of education you have completed?	0<br>None	1<br>Primary	2<br>Secondary	3<br>Institutional degree/ Certificate	4<br>Bachelor's or higher
1.4. How long have you been a community health worker?					

Boston College IRB
Approved
October 18, 2019
Through August 20, 2020

FOR APPROVALS WITH NO CR REQUIRED:

Boston College
IRB Approved
September 4, 2020-
September 3, 2021

The next questions ask about your opinions on THE FAMILY STRENGTHENING INTERVENTION. Please answer the question to the best of your knowledge. If you don't know how much or do not have an opinion about the question, please indicate that.

2. Acceptability

Question	Not at all	A little bit	A moderate amount	A lot	How much don't know
**THE FAMILY STRENGTHENING INTERVENTION**					
2.1 How much do you like *providing* THE FAMILY STRENGTHENING INTERVENTION?	1	2	3	4	8
2.2 How much do you feel good about providing THE FAMILY STRENGTHENING INTERVENTION?	1	2	3	4	8
2.3 How much do you feel good about THE FAMILY STRENGTHENING INTERVENTION as a program to help families raise their children well?	1	2	3	4	8
2.4 How much did you enjoy learning THE FAMILY STRENGTHENING INTERVENTION?	1	2	3	4	8
2.5 How much do you feel that the skills you have learned by providing this intervention will be useful in helping other families?	1	2	3	4	8
2.6 How much do you feel that the components (e.g. the activities that you do in sessions with families) of THE FAMILY STRENGTHENING INTERVENTION make sense?	1	2	3	4	8
2.7 How satisfied are you with the training you received in THE FAMILY STRENGTHENING INTERVENTION?	1	2	3	4	8
2.8 How satisfied are you with the supervision you receive when providing THE FAMILY STRENGTHENING INTERVENTION?	1	2	3	4	8
2.9 How much is the FAMILY STRENGHTENING INTERVENTION material (i.e. manual, paperwork) clear in LOCAL LANGUAGE?	1	2	3	4	8
2.10 How satisfied are you with the mobile tools that you used to monitor your performance providing the intervention?	1	2	3	4	8

Boston College IRB Approved
October 18, 2019
Through August 20, 2020

FOR APPROVALS WITH NO CR REQUIRED:

Boston College IRB Approved
September 4, 2020-
September 3, 2021

3. Appropriateness

Question	Not at all	A little bit	A moderate amount	A lot	How much don't know
**SOCIAL/CULTURAL**					
3.1 How well does THE FAMILY STRENGTHENING INTERVENTION fit the cultural values of families in your community?	1	2	3	4	8
3.2 How well does THE FAMILY STRENGTHENING INTERVENTION fit with your own personal values?	1	2	3	4	8
3.3 How well does THE FAMILY STRENGTHENING INTERVENTION fit with your cultural values?	1	2	3	4	8
3.4 How much is THE FAMILY STRENGTHENING INTERVENTION consistent with the male culture in your country?	1	2	3	4	8
3.5 How much is THE FAMILY STRENGTHENING INTERVENTION consistent with the female culture in your country?	1	2	3	4	8
**SELF PERCEPTION OF EFFECTIVENESS**					
3.7 How much is THE FAMILY STRENGHTENING INTERVENTION a good way to address problems of families?	1	2	3	4	8
3.8 How much does THE FAMILY STRENGHTENING INTERVENTION help families feel better?	1	2	3	4	8
3.9 How much is THE FAMILY STRENGTHENING INTERVENTION effective for caregiver mental health problems?	1	2	3	4	8
3.10 How much is THE FAMILY STRENGTHENING INTERVENTION likely to be effective for families in other parts of your country?	1	2	3	4	8
**TASK FIT**					
3.11 How much is providing THE FAMILY STRENGTHENING INTERVENTION something you feel you should be doing as part of your job?	1	2	3	4	8

Boston College IRB Approved October 18, 2019 Through August 20, 2020

~~FOR APPROVALS WITH NO CR REQUIRED:~~

Boston College IRB Approved September 4, 2020- September 3, 2021

4. Feasibility

Question	Not at all	A little bit	A moderate amount	A lot	How much don't know
**SKILLS**					
4.1. Are you sufficiently skilled at providing THE FAMILY STRENGTHENING INTERVENTION to your families?	1	2	3	4	8
**TIME**					
4.2. Do you have enough time for all the activities that go into providing THE FAMILY STRENGTEHNING INTERVENTION (e.g., How much documentation, handling safety)?	1	2	3	4	8
4.3. Do you have enough time to spend in supervision activities related to THE FAMILY STRENGTHENING INTERVENTION?	1	2	3	4	8
4.4. Do you have enough time to regularly provide THE FAMILY STRENGTHENING INTERVENTION to those who need it?	1	2	3	4	8
4.5. Do you have enough time to travel to and from appointments for THE FAMILY STRENGTHENING INTERVENTION?	1	2	3	4	8
**RESOURCES**					
4.6. Are you paid enough to provide THE FAMILY STRENGTHENING INTERVENTION?	1	2	3	4	8
4.7. Are you provided with the necessary money for transportation to regularly provide THE FAMILY STRENGTHENING INTERVENTION?	1	2	3	4	8
4.8. Do you have the right equipment (eg: pens/pencils/computer/internet, toys, art supplies) to regularly provide THE FAMILY STRENGTHENING INTERVENTION?	1	2	3	4	8
4.9. Do you have the resources (eg: phone, talk time) to reach your clients and/or supervisor when needed?	1	2	3	4	8

Boston College IRB Approved
October 18, 2019
Through August 20, 2020

FOR APPROVALS WITH NO CR REQUIRED:

Boston College
IRB Approved
September 4, 2020-
September 3, 2021

4.10. Are you able to reach your supervisor when needed?	1	2	3	4	8
4.11. How many hours a week do you spend on other activities apart from seeing clients related to providing THE FAMILY STRENGHTENING INTERVENTION?					
**SUPERVISION**					
4.12. Are there enough providers trained in THE FAMILY STRENGHTEHNING INTERVENTION for those that need it within the population you work with?	1	2	3	4	8
4.13. Are you able to reach your supervisor when needed?	1	2	3	4	8
4.14. Do you have sufficient access to continued clinical support and training?	1	2	3	4	8
4.15. Do you have support for self-care when needed?	1	2	3	4	8
4.16. Do you feel that you have access to the support you need to be emotionally healthy enough to provide THE FAMILY STRENGTHENING INTERVENTION?	1	2	3	4	8
4.17. In general, about how many hours per week do you spend providing THE FAMILY STRENGHTEHNING INTERVENTION to families?	1	2	3	4	8
4.18. In general, about how many days per week are you available to provide THE FAMILY STRENGTHENING INTERVENTION to families?	1	2	3	4	8
4.19. How many hours a week do you spend on other activities apart from those related to providing THE FAMILY STRENGTHENING INTERVENTION?	1	2	3	4	8
4.20. How many hours per week do you spend in supervision related meetings?					

Boston College IRB
Approved
October 18, 2019
Through August 20, 2020

FOR APPROVALS WITH NO CR REQUIRED:

Boston College
IRB Approved
September 4, 2020-
September 3, 2021

5. mHealth Tool Usability

Think about your experience using the mHealth tools. Please indicate how much you agree or disagree with each statement about the tools. In these statements, the word "system" refers to the mHealth tools.

Question	Strongly disagree	Disagree	Neutral	Agree	Strongly Agree
**5.1 I think that I would like to use this system frequently.**					
**5.2 I found this system too complex.**					
**5.3 I thought the system was easy to use.**					
**5.4 I think I would need help from a technical person to use this system.**					
**5.5 I found that the different functions and components of the system fit together well.**					
**5.6 I thought there was too much inconsistency in the system.**					
**5.7 I think most people would learn to use this system very quickly.**					
**5.8 I found this system hard to use.**					
**5.9 I felt confident using this system.**					
**5.10 I needed to learn a lot of things before I could start using this system.**					

Boston College IRB Approved
October 18, 2019
Through August 20, 2020

FOR APPROVALS WITH NO CR REQUIRED:

Boston College IRB Approved
September 4, 2020-
September 3, 2021

IMPLEMENTATION MEASURES Questionnaire: Supervisor

Respondent ID #	
Today's date	

Let's start the interview by asking you some basic questions about yourself

1. Demographic Characters

1.1. What is your gender?	0 Male		1 Female		
1.2. What is your age in years?					
1.3. What is the highest level of education you have completed?	0 None	1 Primary	2 Secondary	3 Institutional degree/ Certificate	4 Bachelor's or higher
1.4. How long have you been a community health worker supervisor?					

Boston College IRB Approved
October 18, 2019
Through August 20, 2020

FOR APPROVALS WITH NO CR REQUIRED:

Boston College IRB Approved
September 4, 2020-
September 3, 2021

The next questions ask about your opinions on THE FAMILY STRENGTHENING INTERVENTION. Please answer the question to the best of your knowledge. If you don't know how much or do not have an opinion about the question, please indicate that.

2. Acceptability

Question	Not at all	A little bit	A moderate amount	A lot	How much don't know
**THE FAMILY STRENGTHENING INTERVENTION**					
2.1 How much do you like *providing supervision* for the FAMILY STRENGTHENING INTERVENTION?	1	2	3	4	8
2.2 How much do you feel good about providing supervision for the FAMILY STRENGTHENING INTERVENTION?	1	2	3	4	8
2.3 How much do you feel good about THE FAMILY STRENGTHENING INTERVENTION as a program to help families raise their children well?	1	2	3	4	8
2.4 How much did you enjoy learning THE FAMILY STRENGTHENING INTERVENTION?	1	2	3	4	8
2.5 How much do you feel that the skills you have learned by providing supervision for the intervention will be useful in supervising other CHWs?	1	2	3	4	8
2.6 How much do you feel that the mobile tools used for supervision (e.g., electronic fidelity checklists, dashboards) make sense?	1	2	3	4	8
2.7 How satisfied are you with the training you received in THE FAMILY STRENGTHENING INTERVENTION?	1	2	3	4	8
2.8 How satisfied are you with the technology training you received prior to using mobile tools for supervision?	1	2	3	4	8
2.9 How much did you feel that the mobile tools to support supervision were useful?	1	2	3	4	8
2.10 How satisfied are you with the mobile tools that you used to monitor CHW performance providing the intervention?	1	2	3	4	8

Boston College IRB Approved October 18, 2019 Through August 20, 2020

FOR APPROVALS WITH NO CR REQUIRED:

Boston College IRB Approved September 4, 2020- September 3, 2021

3. Appropriateness

Question	Not at all	A little bit	A moderate amount	A lot	How much don’t know
**SOCIAL/CULTURAL**					
3.1 How well does THE FAMILY STRENGTHENING INTERVENTION fit the cultural values of families in your community?	1	2	3	4	8
3.2 How well does THE FAMILY STRENGTHENING INTERVENTION fit with your own personal values?	1	2	3	4	8
3.3 How well does THE FAMILY STRENGTHENING INTERVENTION fit with your cultural values?	1	2	3	4	8
3.4 How much is THE FAMILY STRENGTHENING INTERVENTION consistent with the male culture in your country?	1	2	3	4	8
3.5 How much are the mobile tools used for supervision consistent with social norms and practices in your country?	1	2	3	4	8
**SELF PERCEPTION OF EFFECTIVENESS**					
3.7 How much is THE FAMILY STRENGHTENING INTERVENTION a good way to address problems of families?	1	2	3	4	8
3.8 How much does THE FAMILY STRENGHTENING INTERVENTION help families feel better?	1	2	3	4	8
3.9 How much is THE FAMILY STRENGTHENING INTERVENTION effective for caregiver mental health problems?	1	2	3	4	8
3.10 How much are the mobile tools effective for improving supervision and performance monitoring of CHWs?	1	2	3	4	8
**TASK FIT**					
3.11 How much is providing supervision for THE FAMILY STRENGTHENING INTERVENTION something you feel you should be doing as part of your job?	1	2	3	4	8

Boston College IRB Approved October 18, 2019 Through August 20, 2020

FOR APPROVALS WITH NO CR REQUIRED:

Boston College IRB Approved September 4, 2020- September 3, 2021

4. Feasibility

Question	Not at all	A little bit	A moderate amount	A lot	How much don't know
**SKILLS**					
4.1. Are you sufficiently skilled at providing supervision for CHWs delivering THE FAMILY STRENGTHENING INTERVENTION ?	1	2	3	4	8
4.2 Are you sufficiently skilled at using mobile tools for supervision?	1	2	3	4	8
**TIME**					
4.3. Do you have enough time to spend in supervision activities related to THE FAMILY STRENGTHENING INTERVENTION?	1	2	3	4	8
4.4. Do you have enough time to regularly provide supervision for CHWS delivering THE FAMILY STRENGTHENING INTERVENTION to those who need it?	1	2	3	4	8
4.5. Do you have enough time to travel to and from supervision sessions for THE FAMILY STRENGTHENING INTERVENTION?	1	2	3	4	8
**RESOURCES**					
4.6. Are you paid enough to provide supervision for THE FAMILY STRENGTHENING INTERVENTION?	1	2	3	4	8
4.7. Are you provided with the necessary money for transportation to regularly provide supervision for THE FAMILY STRENGTHENING INTERVENTION?	1	2	3	4	8
4.8. Do you have the right equipment to regularly provide supervision for THE FAMILY STRENGTHENING INTERVENTION?	1	2	3	4	8
4.9. Do you have the resources (eg: phone, talk time) to reach CHWs you supervise when needed?	1	2	3	4	8

Boston College IRB Approved
October 18, 2019
Through August 20, 2020

FOR APPROVALS WITH NO CR REQUIRED:

Boston College
IRB Approved
September 4, 2020-
September 3, 2021

4.10. How many hours a week do you spend on other activities apart from providing supervision for THE FAMILY STRENGHTENING INTERVENTION?	1	2	3	4	8
**SUPERVISION**					
4.11. Are there enough supervisors trained in THE FAMILY STRENGTHENING INTERVENTION for those that need it within the population you work with?	1	2	3	4	8
4.12. Are you able to reach CHWS when needed?	1	2	3	4	8
4.13. Do you have sufficient access to continued clinical support and training?	1	2	3	4	8
4.14. Do you have support for self-care when needed?	1	2	3	4	8
4.15. Do you feel that you have access to the support you need to be emotionally healthy enough to provide supervision for the FAMILY STRENGTHENING INTERVENTION?	1	2	3	4	8
4.16. In general, about how many hours per week do you spend providing supervision related to the FAMILY STRENGTHENING INTERVENTION?	1	2	3	4	8
4.17. In general, about how many days per week are you available to provide supervision related to the FAMILY STRENGHTENING INTERVENTION?	1	2	3	4	8
4.18. How many hours a week do you spend on other activities apart from providing supervision related to the FAMILY STRENGTHENING INTERVENTION?	1	2	3	4	8
4.19. How many hours per week do you spend in supervision related meetings?					

Boston College IRB Approved
October 18, 2019
Through August 20, 2020

FOR APPROVALS WITH NO CR REQUIRED:

Boston College IRB Approved
September 4, 2020-
September 3, 2021

5. mHealth Tool Usability

Think about your experience using the mHealth tools. Please indicate how much you agree or disagree with each statement about the tools. In these statements, the word “system” refers to the mHealth tools.

Question	Strongly disagree	Disagree	Neutral	Agree	Strongly Agree
**5.1 I think that I would like to use this system frequently.**					
**5.2 I found this system too complex.**					
**5.3 I thought the system was easy to use.**					
**5.4 I think I would need help from a technical person to use this system.**					
**5.5 I found that the different functions and components of the system fit together well.**					
**5.6 I thought there was too much inconsistency in the system.**					
**5.7 I think most people would learn to use this system very quickly.**					
**5.8 I found this system hard to use.**					
**5.9 I felt confident using this system.**					
**5.10 I needed to learn a lot of things before I could start using this system.**					

Boston College IRB Approved
October 18, 2019
Through August 20, 2020

FOR APPROVALS WITH NO CR REQUIRED:

Boston College IRB Approved
September 4, 2020-
September 3, 2021

Post Intervention Interview for Caregivers

WELCOME/PREAMBLE

Thank you again for agreeing to participate in this study. In order to help me understand what your experience with the Family Strengthening Intervention has been like, I would like to ask you some follow-up questions that we ask all of the families who participate in the intervention.

In order to remember what we talk about in these family interviews, I will audio record our conversation and I will write notes. We will then write a report of what was said in our interview. The identity of your family members will be kept confidential, meaning that we will not use your name or other identifying information in any of the reports or summaries of our interview today. Do you have any questions?

Remember, this is your time and we want to hear from you. There are no right or wrong answers. Please feel comfortable to talk about your thoughts and opinions openly. As we discussed in the consent form, I will not reveal anything that you say without your permission, unless I am worried about your safety or the safety of someone else. Do you have any questions about that?

Thank you again for helping us with this important project. By sharing your ideas today, we hope to improve our understanding of how we can help families with young children in Sierra Leone. Do you have any questions?

Now, let's get started.

1. In general, how have things been going since you have been in the Family Strengthening Intervention?

2. What was it like for your family to do the intervention?
   PROBE: What were the things that you liked or didn't like about the intervention?
   PROBE: How would you describe the experience of being in the intervention?

3. How feasible or not was it for your family to participate in the intervention?
   PROBE: What challenges, if any, did your family face in taking part in the intervention?
   PROBE: Amount of time spent meeting with "coaches," scheduling, privacy, explanations to community members, transportation to see social workers if needed, etc.

4. How feasible was it for you and your family to try to find time to repeat some activities and strategies learned through the intervention?
   PROBE: How were you satisfied with the time between sessions, was it enough so to help you to practice what you learned through the intervention?
   PROBE: Does everyone in the family, most particularly parents do find time to talk and help their children to learn new things?

Boston College IRB Approved
October 18, 2019
Through August 20, 2020

Boston College
IRB Approved
September 4, 2020-
September 3, 2021

5. How satisfied were you and your family with the intervention overall?
PROBE: What if anything went well for your family in the intervention?
PROBE: What if anything didn't go so well for your family in the intervention?
Include long descriptions where possible.

6. How did participating in the intervention impact your family, if at all? *Include examples.*
PROBE: What changes have you noticed in your family since the program ended?
PROBE: In what ways, if any, did things stay the same?
PROBE: Daily routines, communication, the way individual family members think and feel

7. How is each of the different people in the family doing today compared to where they were before the intervention?
PROBE: What sort of specific issues, good and bad, have you experienced?
PROBE Are there any particular struggles or difficulties that your family faces at present?

8. What, if anything, about how you interact with your children has changed after participating in the Family Strengthening Intervention?

9. In what ways, if at all, did participating in the intervention influence **relationships between caregivers and children**? *Include examples.*
PROBE: parenting, communication, discipline, the way caregivers think and feel about their children

10. *For dual caregivers:* In what ways, if at all, did participating in the intervention influence **relationship with your intimate partner.** ? *Include examples.*
PROBE: What changes have you notices?
PROBE: In what ways, if any, did things stay the same?
PROBE: In what ways, if any, do you and your partner handle problems differently since the intervention?

11. What, if anything, did you learn during the intervention that you did not know before?
PROBE: Skills, problem-solving, communication, new knowledge

12. In what ways, if at all, did the Family Strengthening Intervention impact your family's understanding of **children's development**?
PROBE: What, if anything, did you learn about young children's development that you did not know before?
PROBE: What, if anything, did you learn about nutrition that you did not know before?

13. What was it like to work with your "coach"?

Boston College IRB Approved
October 18, 2019
Through August 20, 2020

FOR APPROVALS WITH NO CR REQUIRED:

Boston College IRB Approved
September 4, 2020-
September 3, 2021

PROBE: What, if anything, did you like or not like about your "coach"?
Include examples.
PROBE: In what ways were you satisfied or dissatisfied with your "coach"?
PROBE: What, if anything, went well with your "coach"?
PROBE: What if anything didn't go so well with your "coach"?
PROBE: How do you think members of the community regard the coach after the intervention?

14. In what ways, if at all, can we improve the intervention to better assist families raising young children in Sierra Leone?

15. Are there any other things that I haven't asked you about? Please feel free to tell me about any additional thoughts or ideas you have related to the intervention.

Thank you!

Boston College IRB Approved
October 18, 2019
Through August 20, 2020

FOR APPROVALS WITH NO CR REQUIRED:

Boston College IRB Approved
September 4, 2020-
September 3, 2021

Semi-Structured Interview Guide: CHW Coaches

WELCOME/PREAMBLE

Thank you again for agreeing to share your experiences as a coach with me. In order to help me understand what your experience with the Family Strengthening Intervention has been like, I would like to ask you some follow-up questions.

In order to remember what we talk about, I will audio-record our conversation and I will write notes. I will then write a report of what was said in our interview. Your name will not be included on the report, and I will not tell anyone what you said today. When the results are reported, all responses will be de-identified. Do you have any questions?

Remember, this is your time and we want to hear from you. There are no right or wrong answers. Please feel comfortable to talk about your thoughts, opinions, and experiences openly.

By sharing your ideas today, we hope to improve our understanding of how we can help families better understand early childhood development and strengthen their families to help improve their child's development. Do you have any questions?

Now let's get started.

1. **Tell me about your experience with facilitating the Family Strengthening Intervention in Sierra Leone.**
    - What do you think went particularly well in the intervention?
    - What aspects of the intervention did not go well?
    - What challenges, if any, did you face in being a coach?

2. **In what ways, if any, did participating in the intervention impact the families you worked with?**
    - How were family relationships at the beginning, middle and end of the intervention? Describe any changes or anything that didn't change over time.
    - What did you observe happening between the family members and you as the intervention went on?
    - Tell me about what you think participants got out of the Family Strengthening Intervention?

3. **Tell me about the ways the content of the intervention fit or did not fit the needs of the families you worked with.**
    - What things, if anything, were particularly good or helpful about the intervention for the kinds of problems Sierra Leonean children and families have?
    - What things, if anything, were not helpful about the intervention?
    - What sessions were most helpful and why?
    - What things, if anything, felt like they didn't get enough attention or that families needed more time to discuss?

4. **What particular strategies or techniques did you use to build rapport with the families and facilitate the intervention?**

5. **How feasible or not was it for families to participate in the intervention?**
    - What challenges, if any, did families face participating in the intervention?
    - Amount of time spent meeting with coaches, scheduling, privacy, explanations to community members.

Boston College IRB Approved
October 18, 2019
Through August 20, 2020

FOR APPROVALS WITH NO CR REQUIRED:

Boston College IRB Approved
September 4, 2020-
September 3, 2021

6. **How satisfied or dissatisfied were the families with the intervention overall?**
   - What, if anything, seemed to go well?
   - What, if anything, did families find comfortable or uncomfortable?
   - What feedback did families share with you about the intervention?

7. **How satisfied or dissatisfied were YOU with the intervention overall?**
   - What were the best things about the intervention?
   - What were the worst things about the intervention?
   - What, if anything, did you find surprising or unexpected in working with families?
   - What challenges did you face in conducting the intervention?
   - What were your most rewarding moments?

8. **Tell me about your experience with the Family Strengthening training you received <u>before</u> the intervention.**
   - Who led your training, and what did your training consist of?
   - In what ways, if at all, did you feel prepared when you began?
   - In what ways, if at all, did you not feel prepared when you began?
   - How do you feel now compared to before the intervention began?
   - What could we have added to the training that would have helped you?

9. **Tell me about your experience with the technology training you received <u>before</u> the intervention.**
   - In what ways, if at all, were the trainings helpful to you during the intervention?
   - What parts of the training were particularly helpful or unhelpful?

10. **Tell me about your experience with Family Strengthening Intervention supervision.**
    - In what ways, if any, was the supervision you received from supervisors helpful or not helpful?
    - What else could we have done in terms of supervision that would have helped you?

11. **Tell me about your experiences using the mHealth Tools?**
    - In what ways were these tools easy to use?
    - In what ways were these tools hard to use?
    - Did you face any challenges in using these tools?
    - How satisfied or dissatisfied were you with these tools?
    - What did you like the most about these tools?
    - What did you like the least about these tools?
    - Did you feel like the technology training prepared you to use these tools?
    - What could be done to improve these tools?

CLOSING

12. **In what ways, if at all, can we improve the intervention to better assist families to raise their children well?**

13. **In what ways, if at all, can we improve the mHealth tools to better support supervision and training?**

14. **In what ways, if at all can we improve the intervention to better assist and support coaches?**
    - What, if anything, would have made being a coach better for you?
    - What other resources or materials would have made it easier to deliver the intervention?

Boston College IRB
Approved
October 18, 2019
Through August 20, 2020

FOR APPROVALS WITH NO CR REQUIRED:

Boston College
IRB Approved
September 4, 2020-
September 3, 2021

Semi-Structured Interview Guide: CHW

Supervisors

WELCOME/PREAMBLE

Thank you again for agreeing to share your experiences as a supervisor with me. In order to help me understand what your experience as a supervisor for CHWs delivering the Family Strengthening Intervention has been like, I would like to ask you some follow-up questions.

In order to remember what we talk about, I will audio-record our conversation and I will write notes. I will then write a report of what was said in our interview. Your name will not be included on the report, and I will not tell anyone what you said today. When the results are reported, all responses will be de-identified. Do you have any questions?

Remember, this is your time and we want to hear from you. There are no right or wrong answers. Please feel comfortable to talk about your thoughts, opinions, and experiences openly.

By sharing your ideas today, we hope to improve our understanding of how we can help improve supervision, training, and service delivery quality of CHWs in Sierra Leone who work with families. Do you have any questions?

Now let's get started.

1. **Tell me about your experience as a supervisor for the Family Strengthening Intervention in Sierra Leone.**
    - What do you think went particularly well in the intervention?
    - What aspects of the intervention did not go well?
    - What challenges, if any, did you face in being a superivsor?

2. **Tell me about the ways the electronic fidelity checklist and audio recordings helped you provide supervision to CHWs delivering the Family Strengthening Intervention.**
    - What things, if any, were particularly good or helpful about the electronic fidelity checklist?
    - What things, if any, were not helpful about the electronic fidelity checklist?
    - What things, if any, were particularly helpful about viewing the audio recording clips?
    - What things, if any, felt too hard or burdensome about viewing the audio recording clips? Or completing the fidelity checklist?

3. **How feasible or not was it to provide supervision to CHWs delivering the intervention?**
    - What challenges, if any, did CHWs face participating in supervision?

Boston College IRB Approved
October 18, 2019
Through August 20, 2020

FOR APPROVALS WITH NO CR REQUIRED:

Boston College IRB Approved
September 4, 2020-
September 3, 2021

- What challenges, if any, did you face leading supervision?
-

4. **How satisfied or dissatisfied were you with the mHealth supported supervision?**
   - What, if anything, seemed to go well?
   - What, if anything, was challenging about supervision?
   - What were your most rewarding moments?
   - What else could we have done in terms of supervision that would have helped you?

5. **Tell me about your experience with the Family Strengthening training you received before the intervention.**
   - Who led your training, and what did your training consist of?
   - In what ways, if at all, did you feel prepared when you began?
   - In what ways, if at all, did you not feel prepared when you began?
   - How do you feel now compared to before the intervention began?
   - What could we have added to the training that would have helped you?

6. **Tell me about your experience with the technology training you received before the intervention.**
   - In what ways, if at all, were the trainings helpful to you during the intervention?
   - What parts of the training were particularly helpful or unhelpful?

7. **Tell me about your experience with Family Strengthening Intervention supervision.**
   - In what ways, if any, was the supervision you received from supervisors helpful or not helpful?
   - What else could we have done in terms of supervision that would have helped you?

8. **Tell me about your experiences using the mHealth Tools?**
   - In what ways were these tools easy to use?
   - In what ways were these tools hard to use?
   - Did you face any challenges in using these tools?
   - How satisfied or dissatisfied were you with these tools?
   - What did you like the most about these tools?
   - What did you like the least about these tools?
   - Did you feel like the technology training prepared you to use these tools?
   - What could be done to improve these tools?

CLOSING

9. **In what ways, if at all, can we improve the intervention to better assist families to raise their children well?**

Boston College IRB Approved
October 18, 2019
Through August 20, 2020

10. **In what ways, if at all, can we improve the mHealth tools to better support supervision and training?**

FOR APPROVALS WITH NO CR REQUIRED:

Boston College
IRB Approved
September 4, 2020-
September 3, 2021

11. In what ways, if at all can we improve supervision and training to better support CHWs providing services to families?

- What, if anything, would have made being a supervisor better for you?
- What other resources or materials would have made it easier to supervise CHWs?

Boston College IRB Approved
October 18, 2019
Through August 20, 2020

FOR APPROVALS WITH NO CR REQUIRED:

Boston College IRB Approved
September 4, 2020-
September 3, 2021

UI/UX Think Aloud and Semi-Structured Interview with Interventionist

Date: ___________________

Interventionist name: _________________

Interviewer name: _________________

Base-line UI/UX Think Aloud and Semi-Structured Interview with Interventionist

Thank you so much for taking the time to test out our new digital tools to support supervision and performance monitoring. As part of our pilot testing, we would like to give you a prototype version of the digital tools to test. In this first half, we would like for you to go through the digital tools without our assistance, and we will simply watch and listen silently as you provide a first impression of your experience.

We ask that you "think aloud," and verbalize any and all of the thoughts that come to your mind as you use the digital tools. Before we continue with the introduction, we are going to show you a brief video that demonstrates what a Think Aloud could look like.

Play video

If you noticed, he did not read all of the text he saw on the app, but instead, attempted to navigate through it and explain that process. After watching the video, do you have any questions about the think aloud process?

Following your "think aloud" with the app, I will then ask you some follow up questions and clarify any parts of the "think aloud" process that need doing so. Throughout this process, I will be audio-recording and writing notes to make sure that I have a complete picture of your impressions of the digital tools. Do you have any questions?

Before we start, I would like to briefly summarize some important things to keep in mind:

1. Don't worry about forming fully developed thoughts, we would just like to hear you verbalize your thoughts like a stream of consciousness as you navigate through the user-interface.
2. We want honest feedback. Please don't hesitate to point out what you don't like. Both positive and negative feedback about the digital tools will help us to improve them.

Thank you again for participating today. Any questions?

If you don't have any more questions, you can begin using the digital tools and speaking your thoughts out loud…
Now that you've had a chance to use the digital tools, I'd like to ask a couple of follow up questions about your experience.

Boston College IRB Approved
October 18, 2019
Through August 20, 2020

FOR APPROVALS WITH NO CR REQUIRED:

Boston College IRB Approved
September 4, 2020-
September 3, 2021

1. How clear, if at all, was it for you when navigating the digital tools? That is, did you find it easy or difficult to navigate without any previous instruction?
2. How do you feel about the way the digital tools looks? How do you feel about the way the digital tools are structured?<br><br>Probe: What, if anything, about the digital tools specifically makes them easier/harder to engage with?
3. How easy to read, if at all, is the text in the digital tools?
4. How easy, if at all, was it to understand icons or other images used in the digital tools?<br><br>Probe: How did the content flow for you?
5. What aspects of the digital tools, if any, did you find confusing or challenging?

Boston College IRB
Approved
October 18, 2019
Through August 20, 2020

FOR APPROVALS WITH NO CR REQUIRED:

Boston College
IRB Approved
September 4, 2020-
September 3, 2021

6. What kinds of features would you like to see added to the digital tools?

7. What features do you feel are unnecessary and could be removed?

8. What are some of the strongest features/qualities and why?)

9. Do you think the digital tools would be helpful to you for supervision and performance monitoring? Why or why not?

10. Do you think other CHWs and/or supervisors would find the digital tools useful? Why or why not?

11. What challenges, if any, do you anticipate other CHWs and/or supervisors experiencing with the digital tools?

Boston College IRB Approved
October 18, 2019
Through August 20, 2020

FOR APPROVALS WITH NO CR REQUIRED:

Boston College IRB Approved
September 4, 2020-
September 3, 2021

12. Do you think someone's previous experience with technology would impact their ability to learn how to use the digital tools effectively? Why or why not?
13. What concerns, if any, do you have about the digital tools?
14. Is there anything else you would like to share?

Thank you again very much for taking the time to meet with us and participate in this process. Your feedback will be very helpful!

Boston College IRB
Approved
October 18, 2019
Through August 20, 2020

FOR APPROVALS WITH NO CR REQUIRED:

Boston College
IRB Approved
September 4, 2020-
September 3, 2021

Protocol Title: mHealth Tools to Improve Service Delivery Quality of an Evidence-Based Family Home Visiting Intervention to Prevent Family Violence among High Risk Families in Sierra Leone

PI Name: Desrosiers, Alethea

IRB#: 21.006.01

Date: August 4, 2020

Memo: Boston College IRB Revised Application and Responses to Reviewer Queries

To Whom it May Concern:

The Purpose of this memorandum is to confirm that protocol #21.006.01 has been revised and resubmitted as per the requests of the BC IRB. Responses to the reviewer queries are detailed below. Revisions that appear in the text of the application, the consent forms, and the recruitment scripts are bolded. We have also included the requested list of acronyms and the organizational chart. Thank you for your time and careful consideration of this protocol. I am more than happy to participate in a zoom call with the board if this would be helpful to clarify additional questions. Dr. Jordan Farrar, Associate Director of Research for the Research Program on Children and Adversity, Boston College School of Social Work, can also be available for a zoom call. Please do not hesitate to contact me with any further questions or concerns.

Sincerely,

Alethea Desrosiers, Ph.D.
Research Assistant Professor
Boston College School of Social Work
140 Commonwealth Ave
Chestnut Hill, MA 02467

Boston College IRB Approved
October 18, 2019
Through August 20, 2020

FOR APPROVALS WITH NO CR REQUIRED:

Boston College IRB Approved
September 4, 2020-
September 3, 2021

General:

1. Please add Chokdee Rutirasiri to your list of research staff in the protocol. Please also upload his training certificate.

We have uploaded Chokdee Rutirasiri's CITI training certificate and added him to our research staff list.

2. Please describe how Dr. Esliker and the research assistants from the University of Makeni will be trained. If there is a training course similar to CITI that they have taken or will take, please upload their training certificates.

Dr. Esliker and the research assistants will complete CITI training and training in Good Clinical Practice. We have uploaded Dr. Esliker's CITI training certificate. Additionally, the Program Manager will be responsible for developing a more study-specific training plan and schedule of activities for the research assistants to cover topics such as risk of harm protocols and referral processes. Because we do not yet have funding for this study, we have not yet hired a Program Manager. After we have hired the Program Manager and the Program Manager has outlined the training plan, we can forward it to the BC IRB as needed. CITI training certificates of research assistants will be uploaded after they are completed.

3. Do you expect the CHWs to be able to read? If so, please provide a rationale for why you would like to obtain oral consent rather than a physical signature for consent documents in section VI of the application. Also, a signature waiver should be requested for relevant consent forms in section VI.

CHWs in Sierra Leone have the ability to read and write, but literacy levels are generally low. Due to the low literacy levels, we would like to obtain oral consent. This is the same procedure we have used to obtain informed consent from interventionists in our Youth Forward study in Sierra Leone, which recruited lay workers from the community to deliver an evidence-based mental health intervention.

We have added the signature waiver request for relevant consent forms.

4. Please note that you cannot begin data collection or recruitment until you submit an amendment with the Sierra Leone ethics board approval letter.

We have taken note of this.

The IRB would like you to provide more cultural context for the study due to the fact that we typically require local ethics approval **prior** to issuing an IRB approval, but you indicated that they will not issue an approval until the BC IRB approval has been granted.

5. Please specify which cultural (ethnic, tribal) communities you intend to recruit, as there are many in Sierra Leone. How will you ensure that the study is culturally appropriate for these population(s)?

We plan to recruit participants in Makeni. We do not plan to screen for ethnic or tribal group, and we do not ask potential participants to provide this information. In our ongoing NIH-funded U19 and R01 studies in Sierra Leone, we recruited participants in three rural districts (Kailahun, Kono, and Koinadugu), and we did not screen or target specific ethnic groups. Since we will be

Boston College IRB
Approved
October 18, 2019
Through August 20, 2020

FOR APPROVALS WITH NO CR REQUIRED:

Boston College
IRB Approved
September 4, 2020-
September 3, 2021

working in communities, targeting a specific tribal group could be stigmatizing and exclusionary. Our goal is for the family strengthening intervention to be culturally relevant to all communities, regardless of tribal history. This was our approach in developing the Youth Readiness Intervention in Sierra Leone, which we have now scaled out within the context of employment programming in our ongoing Youth Forward study. Our Community Advisory Board will also consist of diverse members to help ensure that the intervention is culturally relevant. We have successfully used Community Advisory Boards in the development of the Family Strengthening Intervention and the Youth Readiness Intervention, and this will be a key part of the cultural adaptation process in the current study.

6. Please consider the ethnic composition of your Community Advisory Board, and whether it represents the groups who will serve as subjects.

We will work with community stakeholders as we configure the Community Advisory Board and will consider important factors like ethnicity when inviting participants.

7. Please specify whether you plan to match family and CHW on ethnicity, as the Krio people have a high social status and the dynamics between the families and researchers may be affected. Is there a recent history of inter-group conflict that needs to be considered? The same issue applies to the CHWs and their supervisors, since some of these groups are also serving as subjects.

There is no recent history of inter-group conflict in Sierra Leone. Because CHWs and their supervisors will already be working with families in the same communities in Makeni from which we recruit families, and also living in these communities, we will not match further based on ethnicity. The National Community Health Worker Policy states that CHWs are required to live in that community and communicate in the languages spoken within that community. Matching based on ethnicity could trigger issues related to discrimination, and we would not be able to enforce matching in the control condition (because this type of matching is not part of existing CHW services) to ensure comparability across conditions. Matching would also go against clearly defined government policy.

8. Is there any concern about CHWs disclosing what they learn about a member of their community? If so, how will you ensure CHWs do not disclose this information?

As part of training in Good Clinical Practice, CHWs will be trained in protocols for protection of participant privacy and confidentiality. This will include guidance about not disclosing the identity of any families who are participating to anyone outside of the research study.

9. Please provide a description of the staff training and procedure for reporting adverse events.

All staff will complete CITI training and training in Good Clinical Practice. As stated in the response to point 2, we can forward a more detailed plan of additional training activities for the research assistants after the Program Manager has been hired. Detailed procedures for reporting adverse events are described in Section G.1 and are as follows:

Assessment of Adverse Events:
Adverse events due to participation in psychosocial assessments are anticipated to be rare; however, we have a plan in place to respond should such adverse events occur. We will train the study team in how to identify signs of distress in caregivers. If a participant becomes

Boston College IRB Approved
October 18, 2019
Through August 20, 2020

FOR APPROVALS WITH NO CR REQUIRED:

Boston College
IRB Approved
September 4, 2020-
September 3, 2021

distressed during the psychosocial assessment, social workers will be available to provide additional individual support and assessment of need for additional mental health services. Should any participant endorse survey items indicating thoughts of suicide, the interviewer will discontinue the survey administration to assess for risk of harm.

In any situation involving study participants in immediate risk of harm (i.e., current suicidal ideation or plan, report of current physical or sexual abuse) the study team member will activate the safety plan.

Safety plan to identify and address risk of harm cases: Situations involving study participants in immediate risk of harm will be triaged by the study team member present at the time of interview. The Program Manager and UNIMAK RAs will discuss all risk of harm cases and report them to the PI. The PI will notify the Boston College IRB of risk of harm cases within 24 hours. Risk of harm cases will be referred to appropriate local mental health counselors and emergency facilities as needed within 24 hours. Study social workers or the Program Manager will be available to provide additional individual support and referrals to local social workers for additional mental health services. A standardized form for reporting any activation of the safety plan will be designed and all study team members will be trained in its application. All RAs will complete CITI training on research ethics and compliance and will receive extensive training on the proper protocol for recognizing and responding to risk of harm situations. The following situations will activate the action plan.

- *Flagged questions: Some survey items on the psychosocial assessment are designed to assess participants' risk behaviors and welfare, including critical issues such as suicidal ideation, physical abuse, and sexual abuse. Specific questions related to these problems are "flagged"; a positive response to flagged questions will mandate activation of the safety plan. The use of flagged questions is intended to minimize variability in research staff response and to ensure that all research personnel respond to certain indicators of risk of harm.*
- *Concerning scores: For example, a concerning score for intimate partner violence reported by a participant will activate the safety plan.*

Potential action plan responses are:

- *If suicidal ideation is reported by a participant: Research staff will conduct an immediate follow-up assessment with the participant. Staff will use scripts, which have been developed for these follow-up sessions, and include specific text for discussing mental health problems and access to services (e.g., "Based on your responses to some of these questions, I have concerns about your safety and would like to have someone on our team touch base with you"). After having this session with any participants in distress, staff will contact the Program Manager for an initial assessment of suicidal risk and will inform the PI. Trained study social workers from CARITAS will conduct this assessment to determine current suicidal ideation or psychosis. Appropriate referrals will be made to the most suitable mental health services (e.g., social work, psychology, or psychiatry) depending on level of clinical need. Referral services will be contacted if suicidal ideation is ongoing and the participant cannot be kept safe with social work and family level interventions.*
- *If ongoing abuse is reported by a participant: Research staff will inform the Program Manager or PI, and if appropriate, a local authority through the current legal, health, and social services channels. If reported abuse relates to a child in the study, a report will be submitted to the Ministry of Social Welfare District Council or Family Support Unit of the*

Boston College IRB Approved
October 18, 2019
Through August 20, 2020

FOR APPROVALS WITH NO CR REQUIRED:

Boston College IRB Approved
September 4, 2020-
September 3, 2021

Police, as required by national law. In abuse cases involving participants, the safety plan recommends informing the participants' primary care or mental health counselors.

- *<u>If a participant scores at very high levels of intimate partner violence or indicates ongoing intimate partner violence</u>: Research study social workers will perform an initial evaluation, confer with the Program Manager and PI, and refer for appropriate care through the primary care counselor or mental health provider.*
- *<u>Referrals for participants in need of stage two treatment</u>: At the time of psychosocial assessment interviews, participants will be asked questions to assess for anxiety and stress. Participants will be asked, "Do you feel you need any additional care or services due to your experiences today? Are there any questions or concerns that you would like to discuss with a mental health provider or other health professional?" Any risk of harm concerns will be discussed at this time. If needed, the counselors will provide referrals to appropriate services. All questions will be referred to Dr. Desrosiers and the Program Manager. All Safety Plan Activation Forms will be saved as an encrypted file in a password protected folder on a password protected computer managed by the Program Manager. This folder will also be saved on Box, a secure HIPAA-compliant, cloud-based platform. The study team will minimize the use of paper logs, instead utilizing computers and tablets to track participant-related study logs.*

We will refer study participants who show persistent impairment and distress due to post-traumatic stress disorder, anxiety, depression, or mental health concerns to mental health nurses with the Peripheral Health Unit in the community or to other community programs. If necessary and with the consent of participants, a trusted community or family member may also be enlisted as a source of interpersonal support. The Program Manager and research staff will discuss all cases requiring treatment and report them to the PI. We will design a standardized form for reporting referrals; all study team members will be trained in its application.

This protection against risk of harm plan has been successfully implemented and used in our prior work in Sierra Leone (U19MH109989/Youth Forward). We have conducted resource and referral mapping to ensure that the appropriate referrals can be made given the geographic location of participants. Available health-related services as well as non-government organizations (NGOs) and community-based organizations have been documented at three levels: the community level, the chiefdom level, and the district level. This data provides a map of available referral services from which the flow of referrals can be documented depending on the situation. This ensures referrals are made to clinics and mental health professionals that are accessible to each participant and appropriate to their specific situation.

Adverse events (AEs) will be graded according to their attribution (either related or unrelated to the study) and their severity. Serious Adverse Events (SAE) are any adverse event that meets one of these criteria:

- *The event results in death*
- *The event is life-threatening*
- *The event results in an inpatient hospitalization or prolongation of existing hospitalization*
- *The event results in permanent or severe disability or permanent damage*
- *A pregnancy results in a congenital anomaly or birth defect*
- *Based on appropriate medical judgment, the event may jeopardize the subject's health and may require medical or surgical intervention to prevent one of the other outcomes listed above*

Boston College IRB Approved
October 18, 2019
Through August 20, 2020

AEs and SAEs will be graded as:

FOR APPROVALS WITH NO CR REQUIRED:

Boston College IRB Approved
September 4, 2020-
September 3, 2021

- *Mild (no limitation of usual activities),*
- *Moderate (some limitation), or*
- *Severe (inability to carry out usual activities).*

AEs and SAEs will be attributed according to the relationship to the study product and/or procedures as:

- *Not related,*
- *Unlikely,*
- *Possible,*
- *Probable, or*
- *Definite.*

Adverse Event Reporting:
Information on AEs and SAEs will be reported to the PI (Desrosiers) and in-country Program Manager on a weekly basis. An adverse event report will be generated for each event that will be included in the weekly report. This weekly AE/SAE report will only be submitted when there are relevant AEs and SAEs to report. The field-based managers and study social workers will meet with the research assistant who triggered the AE or SAE to follow up and document the presenting concerns. The response to ensure safety and access to relevant services will also be documented, including any referrals to social work or mental health services that were made and the subsequent outcome. These monthly reports and weekly AE and SAE reports will be stored in study records.

For AEs and SAEs the PI will submit a Report to the BC IRB within five business days via the online CyberIRB system. Any SAEs will also be reported to the NIMH Program Official (PO) and DSMB and the Sierra Leone Ethics and Scientific Review Committee (see the table below for a detailed plan regarding when and to whom reports will be made):

a) *Local/internal adverse event which in the opinion of the PI are unexpected and at least possibly related to the study procedures.*
b) *A harm is "unexpected" when its specificity and severity are not accurately reflected in the consent document.*
c) *A harm is "at least probably related to the study procedures" if in the opinion of the PI, the research procedures more likely than not caused the harm.*
d) *External adverse event which in the opinion of the PI or NIH requires changes to the protocol or informed consent form.*

Continuing review reports will be submitted to the BC IRB on an annual basis. Reports will be made to the NIMH-PO/DSMB per the usual NIH/NIMH policy. As per the Sierra Leone Ethics and Scientific Review Committee policy, reporting will be done on an annual basis during continuing reviews.

These reports will contain:

a) *The number of AEs that are related, serious, and unexpected that occurred during the review period;*
b) *The number of AEs that are related, expected, and greater than expected in terms of severity or frequency that occurred during the review period;*
c) *The number of AEs that are related, non-serious, and unexpected that occurred during the review period;*
d) *The number of other problems (unanticipated problems, protocol violations, protocol deviations) that occurred during the review period;*

Boston College IRB Approved
October 18, 2019
Through August 20, 2020

FOR APPROVALS WITH NO CR REQUIRED:

Boston College IRB Approved
September 4, 2020-
September 3, 2021

e) The number of complaints that occurred during the review period and how each complaint was handled; and

f) The number of participant withdrawals, an explanation of why the participant withdrew or was withdrawn, how the withdrawals affect participant enrollment numbers during the period of review, and how the withdraws affect overall participant enrollment.

In addition, the PI will report to the BC IRB as needed on:

a) Information that indicates a change to the risks or potential benefits of the Human Research. For example:
   - *An interim analysis, safety monitoring report, publication in the literature, or revised investigator brochure that indicates an increase in the frequency or magnitude of a harm, uncovers a new risk, or provides more information about the benefits of the Human Research.*
   - *Protocol violation that harmed participants or others or that indicates participants or others might be at increased risk of harm.*
   - *Complaint of a participant that indicates participants or others might be at increased risk of harm or at risk of a new harm.*

10. The IRB recommends that the Community Advisory Board review the recruitment and screening process for Sierra Leonean families for cultural appropriateness and feasibility. Please clarify if this is possible, or if they have already contributed to this process.

The recruitment and screening process has been reviewed by our in-country local partners at Caritas-Freetown and the University of Makeni, as well as by the National Community Health Worker Coordinator and Regional Community Health Worker Coordinator at the Ministry of Health and Sanitation. All parties have confirmed the feasibility and cultural appropriateness of our recruitment and screening process. We have also successfully used the same screening process that we propose in this study in our Youth Forward Study (U19MH109989/Youth FORWARD) in Sierra Leone, and the process was both feasible and appropriate. Therefore, we do not think it is feasible or necessary to also request that the Community Advisory Board review the recruitment and screening process.

Recruitment:

1. There was some concern from the IRB committee that the number of times research staff members contact potential participants to recruit them may be seen as coercive. Please consider whether or not calling a household multiple times before visiting on three occasions is excessive and revise accordingly. If you have a strong rationale for using this recruitment method, please explain further.

Our recruitment procedures is to try calling a potential participants three times and then visit the household once if the potential participant did not answer the phone after the third attempt to call. This means that if a participant does not pick up the phone on the first occasion, we attempt to call again on a second occasion. If there is no answer on the second occasion, we will call a third time. If there is no answer the third time, we will visit the household. We have used this protocol in our ongoing NIH-funding studies in Sierra Leone to address challenges related to frequent network connectivity problems, phone number changes, and a high level of mobility. This procedure has helped us reach target enrollment numbers and increased the feasibility of enrolling large or hard to reach samples. Because we do not ask a participant whether they are interested in the study more than once, we do not view this recruitment strategy as either excessive or coercive.

Boston College IRB Approved
October 18, 2019
Through August 20, 2020

FOR APPROVALS WITH NO CR REQUIRED:

Boston College
IRB Approved
September 4, 2020-
September 3, 2021

2. Please describe what you will tell families who do not meet eligibility criteria to mitigate the potential for family members to be upset or think something is "wrong" with them for not meeting the criteria. Do you have a specific script for these situations?

In Section 4. of the Screener, we state the following to families: "Please remember your participation in the screening interview does not mean you will be enrolled in the research program. The research team will review all of the screening interviews and then a member of the team will call you to let you know what will happen next".

We have added the following additional statement: "If you are not eligible for the study, this does not mean anything is wrong with you or your family. If we find that our program works, we hope that all families in Sierra Leone will be able to receive it in the future. If you are not eligible to participate in our program, that does not mean you cannot participate in other available services in your community. If you do not know what those services are, we will be happy to connect you."

3. The beginning of the screening script/form asks whether consent has been completed; however, the revised application indicates that participants will not be consented prior to screening. Please clarify and revise to reduce confusion among participants.

We have removed this first item from the screening form.

4. The screening script/form indicates that participants do not need to answer questions if they do not want to. Please describe what will happen if participants do not answer enough questions on the DERS to allow for adequate scoring.

In our Youth Forward scale up study in Sierra Leone, we used the DERS as a screening instrument. We included the same script language in the current study that we did in the Youth Forward study, stating that participants do not need to answer a question if they do not want to. Out of 2,666 potential participants who completed the DERS screener, missingness was 1.1%, which is very low. We anticipate that missingness will be similarly low in the current study. Additionally, the screener will be programmed in RedCap to automatically compute a total score based on participant responses. Research assistants will be trained to explain response options and encourage participants to respond to the best of their ability.

5. In the recruitment scripts, please add that the University of Makeni is also conducting the research.

We have added this to the recruitment scripts.

6. The recruitment scripts still indicate that participants will receive a "household gift of soap or foodstuffs" as compensation, but the cover letter outlining your revisions indicates that all documents should read "home gift (e.g., soap)." Please make this consistent.

We have revised the language in the recruitment script so that it reads home gift (e.g., soap).

Boston College IRB Approved
October 18, 2019
Through August 20, 2020

7. Probing a participant for the reason(s) they decline to participate or withdraw early from the study may be coercive. Please revise your protocol to omit this part of the process. If a participant declines to participate or wishes to withdraw after consenting, no further questions

FOR APPROVALS WITH NO CR REQUIRED:

Boston College
IRB Approved
September 4, 2020-
September 3, 2021

should be asked.

*We have revised this to read: "If a participant decides to withdraw from the study, **the research team will document this, and the participant will be withdrawn."***

Application:

1. Please provide the NIMH grant number in section III of the application.

We have provided the NIMH grant number: R21MH124071

2. In section V.D.1 of the application, you indicated that family inclusion criteria include "cohabitating caregivers" but then future references are to "parents". Please review the consent forms for consistency about this.

We have reviewed consent forms and updated language to so that "cohabitating caregivers" replaces "parents".

3. In section V.D.5, there appears to be a cut and paste error from Youth Forward Study: "The team will attempt three times to contact the potential participants in-person before considering the **youth** ineligible or uninterested." Please revise.

We have revised this statement.

4. Section V.D.5 states "UNIMAK RAs will also generate an advertisement/flyer that can be displayed at the Peripheral Health Unit." The cover letter indicates that recruitment will not include posting advertisements. This statement should be deleted from the application or you should include the flyer with the application.

Below is the text in section V.D.5. We will not use advertisements.

> *The Program Manager and UNIMAK RAs will work with the Ministry of Health and Sanitation and the CHW focal person to identify potential participants. A recruitment script will be used to explain the study to eligible participants and determine if they are interested in participating. The RAs will attempt to contact potential participants by phone up to three times before traveling to the address provided to contact them in person. The team will attempt three times to contact the potential participants in-person before considering **that potential participant to be** ineligible or uninterested. For those participants who consent to the study, their contact information will be securely stored, and they will be contacted by UNIMAK RAs.*

5. In section V.F.1, you indicated that audio recordings will be kept in locked file cabinets and erased at study completion. What format will be used for audio recordings? We recommend digital voice recorders that allow data to be downloaded to password protected computer audio files as soon as possible after data collection.

We will use digital audio recorders and download data to password protected computer audio files as soon as possible after data collection.

Boston College IRB Approved
October 18, 2019
Through August 20, 2020

FOR APPROVALS WITH NO CR REQUIRED:

Boston College IRB Approved
September 4, 2020-
September 3, 2021

Consent Forms:

1. The consent forms currently state that the study could benefit Sierra Leone. This is an unlikely outcome for the study, and as such, the IRB requests that you revise the expected benefits to be less overstated.

The caregiver consent form stated the following: "Your input about the program will also be used to improve it so that other families in Sierra Leone can benefit too." We do not state that Sierra Leone would benefit, but that other families might benefit. We have revised the statement as follows in attempt to be clearer: "Your input about the program will also be used to improve it so that other families in Sierra Leone can benefit from it". This language is equivalent to the language used in our BC IRB approved consent forms in the ongoing study in Rwanda to scale out the Family Strengthening Intervention, and the goal is to conduct a larger implementation-effectiveness trial in Sierra Leone after we have obtained data on feasibility and preliminary efficacy. The long-term goal would be to make the Family Strengthening Intervention available to all families if the intervention demonstrates effectiveness, and the first step is the pilot-feasibility study that includes input from families on acceptability and appropriateness.

2. Please consider whether everyone you plan to recruit has a phone and what you will do if some do not.

We have consulted with our in-country partners on this matter, and they have informed us that almost all households in Makeni have a phone. In the event that a potential participant does not have a phone, study RAs will be advised to make a home visit. We have used this approach successfully in our Youth Forward study in Sierra Leone (U19MH109989/Youth Forward).

3. The consent for focus groups should include mention that confidentiality cannot be guaranteed by the research team because there are other subjects participating who might disclose information.

We have added the recommended statement above to the consent for focus groups.

Caregiver Consent:

1. Please review the consent form for typographical errors.

We have reviewed the consent form for typographical errors.

2. Please include the number of expected participants/families in each group in the consent form.

We would like to please request that this information is not included in the consent form. Our BC IRB approved consent forms for our two ongoing studies in Sierra Leone do not include the number of participants expected for each group. We are concerned about a lack of consistency in what we include on consent forms across our ongoing projects.

3. Please provide a reminder about the standard services that the control group will receive. (E.g. "As you might know, these services include…"

Boston College IRB Approved
October 18, 2019
Through August 20, 2020

We have added the following statement to the consent form: "As you might know, standard services include 3 home visits to support maternal and child health".

FOR APPROVALS WITH NO CR REQUIRED:

Boston College IRB Approved
September 4, 2020-
September 3, 2021

4. Please specify more explicitly the activities/expectations for those in the intervention group and those in the control group (see below).
For example:
If you are selected to receive the Family Strengthening Intervention, you will…
If you are selected to receive standard home-based visiting services, you will…
We have revised the caregiver consent form to be more explicit about activities in each group.

5. Please indicate in the consent form when and how often questionnaires will be administered to those in the control group.

We have revised the consent form to indicate that both control and intervention families will complete questionnaires according to the same time frame/assessment schedule. The consent form now reads as follows:

*"If you decide that you want to be in our study, you will do three surveys. This will happen before the **family strengthening intervention and standard services** start, after they finish, and then another 3-months later".*

CHW Supervisor/ CHW Consent Forms:

1. There is a typographical error in the title of the consent form (UI/UX) vs (UI/IX). Please revise.

We have revised the typographical error.

2. In the consent form, please indicate that refusal to participate or withdrawal will not affect occupational status.

We have added the word "occupational" to the following statements that were in CHW and supervisor consent forms as follows:

*"You will not lose your **occupational** status as a CHW in the community if you decide not to participate".*

*"You will not lose your **occupational** status as a CHW supervisor in the community if you decide not to participate".*

3. In the consent form, you stated that other people will not know if they are in the study; however, other people in the focus group will know they are in the study. Please revise.

*We have revised this to read "**Besides the other focus group discussion participants,** other people will not know if you are in our study".*

4. The statement, "the information we write down about you and other adult caregivers will be private" is not clear. Who does "other adult caregivers" refer to?

We have revised this statement and omitted the phrase "other adult caregivers".

Boston College IRB
Approved
October 18, 2019
Through August 20, 2020

FOR APPROVALS WITH NO CR REQUIRED:

Boston College
IRB Approved
September 4, 2020-
September 3, 2021

mHealth Supported FSI-ECD in Sierra Leone
Organizational Chart

Ethics and Review Committees

1. Boston College IRB
2. Sierra Leone Ethics and Scientific Review Committee (SLESRC)

BC Funder
National Institute of Mental Health

Partners in the Research:

- Boston College School of Social Work
- University of Makeni
- Caritas Freetown

Government Stakeholders:

- Ministry of Health and Sanitation
  National Community Health Worker Program

Technical Oversight

- Caritas Freetown - Program Manager (based in Sierra Leone)
- Boston College - Principal Investigator

Data Collection

- Caritas Freetown - Program Manager
- University of Makeni - Research Assistants supervised by the Program Manager

FSI-ECD Implementation

- Community Health Workers
- Community Health Work Supervisors

Research Capacity Building

- University of Makeni - Co-Investgator (based in Sierra Leone)

Boston College IRB Approved
October 18, 2019
Through August 20, 2020

FOR APPROVALS WITH NO CR REQUIRED:

Boston College IRB Approved
September 4, 2020-
September 3, 2021

mHealth Supported FSI-ECD in Sierra Leone
Organizational Chart

The above organization chart outlines the key players in the mHealth supported FSI-ECD pilot study in Sierra Leone.

Ethics Committees:
Two separate IRB committees review the study protocol and all associated data collection instruments, consent forms, etc.

- Boston College IRB protocol was submitted on 6/3/2020.
- SLESRC to be submitted pending Boston College IRB approval.

Government Stakeholders:
Community Health Workers and Community Health Worker Supervisors who are part of the National Community Health Worker Program within the Ministry of Health and Sanitation will be recruited to deliver the FSI-ECD and supervise delivery. Community Health Workers will also be recruited to participate in the user centered design process to develop the mHealth tools. Community Health Workers will not collect participant data.

Caritas Freetown:
Caritas Freetown is a Non-Governmental Organization that was established in 1981 by the Catholic Bishops Conference of Sierra Leone and is dedicated to post war peace building initiatives as well as humanitarian relief, youth empowerment, poverty eradication, gender equality, healthcare, and early child development. The Caritas Freetown-based Program Manager will oversee data collection by University of Makeni research assistants. All participant data will collected using RedCap and loaded onto Android tablets. University of Makeni research assistants will consent and enroll participants, with oversight by the Program Manager. Caritas Freetown has extensive experience in both qualitative and quantitative data collection and management, training in good clinical practice and human subjects research ethics, and proper storage of participant data through work on prior NIH-funded studies with Boston College-based researchers. Caritas will be responsible for paying Community Health Workers through their subaward.

University of Makeni:
The University of Makeni is a private university in the Northern Province of Sierra Leone located about two hours from the capital of Freetown. The University of Makeni will be the research capacity building hub for the current study, headed by Co-Investigator Dr. Esliker. Research capacity building activities will include 1:1 mentorship of four student research assistants; short courses in research methods, data collection, and data analysis; access to data to contribute to publications; management of a small research project fund.

Boston College IRB Approved
October 18, 2019
Through August 20, 2020

FOR APPROVALS WITH NO CR REQUIRED:

Boston College IRB Approved
September 4, 2020-
September 3, 2021

Protocol Title: mHealth Tools to Improve Service Delivery Quality of an Evidence-Based Family Home Visiting Intervention to Prevent Family Violence among High Risk Families in Sierra Leone

PI Name: Desrosiers, Alethea

IRB#: 21.006.01

Date: July 1, 2020

Memo: Boston College IRB Revised Application and Responses to Reviewer Queries

To Whom it May Concern:

The Purpose of this memorandum is to confirm that protocol #21.006.01 has been revised and resubmitted as per the requests of the BC IRB. Responses to the reviewer queries are detailed below. Revisions that appear in the text of the application, the consent forms, and the recruitment scripts are bolded. We have also included the requested list of acronyms and the organizational chart. Thank you for your time and careful consideration of this protocol. I am more than happy to participate in a zoom call with the board if this would be helpful to clarify additional questions. Dr. Jordan Farrar, Associate Director of Research for the Research Program on Children and Adversity, Boston College School of Social Work, can also be available for a zoom call. Please do not hesitate to contact me with any further questions or concerns.

Sincerely,

Alethea Desrosiers, Ph.D.
Research Assistant Professor
Boston College School of Social Work
140 Commonwealth Ave
Chestnut Hill, MA 02467

Boston College IRB Approved
October 18, 2019
Through August 20, 2020

FOR APPROVALS WITH NO CR REQUIRED:

Boston College IRB Approved
September 4, 2020-
September 3, 2021

Acronym List

CHW	Community Health Worker
DERS	Difficulties in Emotion Regulation Scale
FSI-ECD	Family Strengthening Intervention for Early Childhood Development
GoSL	Government of Sierra Leone
ICER	Incremental Cost-effectiveness Ratio
IRB	Institutional Review Board
LMICs	Lower- and Middle- Income Countries
NIMH	National Institute of Mental Health
PHU	Peripheral Health Unit
PI	Principal Investigator
QALYs	Quality Adjusted Life Years
RA	Research Assistant
RCT	Randomized Controlled Trial
RPCA	Research Program on Children and Adversity
UI/UX	User Interface/User Experience
UN	United Nations
UNIMAK	University of Makeni
WHO	World Health Organization
WHO-DAS	World Health Organization Disability Assessment Schedule

Boston College IRB
Approved
October 18, 2019
Through August 20, 2020

FOR APPROVALS WITH NO CR REQUIRED:

Boston College
IRB Approved
September 4, 2020-
September 3, 2021

Administrative issues

The project involves personnel from a different university, the University of Makeni. Please provide more detail how the two institutions will share responsibility for the research.

Dr. Esliker, who will serve as the University of Makeni (UNIMAK) Co-Investigator, will be responsible for the following: (a) identifying 3-4 student RAs who will receive 1:1 mentoring from Co-Investigators and training on fundamental research skills; (b) training student RAs to conduct qualitative and quantitative data collection; (c) providing supervision and mentorship to student RAs and monitoring data collection procedures; (d) providing logistical support for RA training, travel and field costs; e) embedding research experiences and access to data for original publications to UNIMAK scholars and students; (f) presenting at the annual H-BioMed Symposium, the U19 Annual Meeting in Freetown, or another agreed upon academic conference; (g) attending the annual 3 day networking meeting at Boston College. Drs. Esliker and Desrosiers will provide close oversight and guidance to the Program Manager and help lead through weekly teleconference meetings, and Dr. Esliker will lead the RA trainings with the Program Manager. Caritas-Freetown will provide office space, logistics, and travel support to the program manager and will also be the link to service referrals for any risk of harm cases via their trained social workers.

As the Principal Investigator, Dr. Desrosiers, Research Assistant Professor at Boston College School of Social Work, will have overall responsibility for project management. She will communicate with Dr. Esliker and the Program Manager via weekly teleconference meetings to coordinate study recruitment, enrollment, and data collection activities and to monitor overall study progress. Dr. Desrosiers will travel to Sierra Leone for onsite visits 1-2 times per year to provide guidance and research mentorship according to current COVID-19 related travel advisories. Dr. Betancourt will also participate in teleconference meetings, as needed, to advise on study progress and ongoing research capacity building activities at the University of Makeni. All risk of harm cases and adverse events will be reported to Dr. Desrosiers and the Boston College IRB. Dr. Desrosiers will oversee all NIH reporting requirements.

1. Please describe the role of the Sierra Leonean Ethics Committee and any analogous committee at the University of Makeni or associated with Caritas.

   The Sierra Leone Ethics and Scientific Review Committee (SLESRC) under the Ministry of Health and Sanitation within the Government of Sierra Leone is the regulatory committee for all scientific research conducted in Sierra Leone that falls under the category of health and sanitation. The SLESRC meets regularly throughout the year, and all research conducted in the country related to health and sanitation must be reviewed by them. The University of Makeni and Caritas do not have an analogous committee.

2. NIH funded studies require a single IRB(click link for details). Please check with your program officer to determine whether the Sierra Leonean Ethics Committee, UNIMAK's IRB (if there is one) and BC's IRB need to enter into a single IRB agreement. This involves additional steps for all participating sites' IRBs. Which IRB is the IRB of record will affect some wording of the consent forms.

   This study was classified as a single-site study by NIH. According to the website link provided on NIH policies for single IRBs, only multi-site, domestic studies require a single IRB. The website states, "this policy applies to the domestic sites of NIH-funded multi-site studies where each site

Boston College IRB
Approved
October 18, 2019
Through August 20, 2020

FOR APPROVALS WITH NO CR REQUIRED:

Boston College
IRB Approved
September 4, 2020-
September 3, 2021

will conduct the same protocol involving non-exempt human subjects research". Additional clarification on this point has been requested from the Program Officer assigned to this study and will be forwarded once it is received.

3. Does any of these review boards have a Federal wide Assurance Number?

   The Sierra Leone Ethics and Scientific Review Committee does not have a Federal Wide Assurance Number. Its IRB number is IRB00007981. The Federal Wide Assurance Number for the Ministry of Health and Sanitation is FWA00009073. The Sierra Leone Ethics and Scientific Review Committee falls within the jurisdiction of the Ministry of Health and Sanitation.

4. Do you currently have approvals for this study? Please upload the approval letters if so.

   We are first seeking approval from the Boston College IRB, and then we plan to seek approval from the Sierra Leone Ethics and Scientific Review Committee.

5. Documentation of human subjects training will be required for members of the research team who will have contact with human subjects or with identifiable data. If CITI training is not appropriate, please describe the alternatives that will be used. Is Good Clinical Practice training part of the training program for all researchers on the project?

   Training in Good Clinical Practice is part of the training program for all researchers in the project. All research team members will be required to complete the CITI training.

6. Provide an Organizational chart

   We have included an organization chart in our revised application.

7. Provide a list of acronyms

   We have included a list of acronyms in our revised application, included in this letter.

Body of the application

1. Please provide a description of how the intervention has been adapted from that used in Rwanda to what is being used in the present application for Sierra Leone. Also provide a copy of the intervention materials and all of the test instruments (including the DERS).

   Cultural adaptation of the intervention for use in Sierra Leone is part of the research study. This will take place in coordination with community advisory boards in Sierra Leone and facilitated by the Program Manager. We have included a copy of the intervention used in Rwanda that will be adapted for use in Sierra Leone. All testing instruments are included in the caregiver quantitative assessment battery and identified in the table of contents by their name. This includes the DERS.

2. The application currently states that community health workers who are part of the intervention will receive financial support for the study duration. Please specify whether the payments are over and above the compensation for CHWs participation in the study (focus groups, interviews,

Boston College IRB
Approved
October 18, 2019
Through August 20, 2020

FOR APPROVALS WITH NO CR REQUIRED:

Boston College
IRB Approved
September 4, 2020-
September 3, 2021

questionnaires), whether the payments come from BC funds, and whether the payments are over and above what the CHWs receive from the government.

CHWs who deliver the FSI-ECD will be paid 30,000 Leones per day (2-3 USD) that they work three hours on the project. This payment is in addition to the incentives CHWs receive from the Government of Sierra Leone. CHWs who deliver the FSI-ECD will receive 18,000 Leones for completion of questionnaires, and those who also participate in key informant interviews will receive 18,000 Leones for their participation in the interview. CHWs who participate in the user-centered design focus group discussions will also receive 18,000 Leones for their participation in each discussion. All funds for CHWs will be provided by Boston College funds through the subaward to Caritas-Freetown, who will handle the processing of these local expenses.

3. Please clarify what research data (if any) community health workers will collect from study subjects (the families). The question is whether or not the CHWs will function as research staff as well as government employees, or just as government employees. Please clarify who will be conducting focus groups, who will be collecting data in each part of the project, who will be delivering the intervention, etc.

   CHWs will not function as research staff. They will not collect study data from families. CHWs will deliver the intervention. The problem analysis focus groups will be facilitated in by the Program Manager and a Directorate of Science, Technology and Innovation (DSTI) team member. Consultant Chokdee Rutirasiri will provide expertise on the user-centered design process and will lead the focus groups remotely (e.g., via zoom or teleconference). Study research assistants will collect participant data from families, CHWs, and CHW supervisors. The Program Manager will oversee all data collection activities. This includes the problem analysis focus groups, UI/UX testing sessions, quantitative assessments for families, key informant interviews with families, key informant interviews with CHWs, and supervisors who delivered the intervention, and implementation science quantitative assessments with CHWs and supervisors.

4. Your application states that research assistants will generate advertisements that will be posed for recruitment, but they are not included. Please include the advertisements with the application materials and describe where they will be posted.

   We have amended the description of the recruitment strategy, as this was an error. Recruitment will not include advertisements.

5. Please provide more detail about the control group families. Is it correct that the control group families will be tested at the same three points in time as the families in the intervention? What are the standard services provided to control families? (This information was missing from the research summary, section C.) In section V.H.2 you indicate that control group families will receive 3 home visits focused on psychoeducation for maternal and child health. This needs to be clarified and more information about the psychoeducational information and who will deliver it needs to be included.

   Control families will complete questionnaires at the same time points as families who receive the intervention. Standard CHW care involves three home visiting, educational sessions delivered to families following childbirth, with weekly supervision via phone or face-to-face. Topics of home visiting sessions include: skilled post-natal care for mothers, early initiation of breastfeeding and exclusive breastfeeding practices, adequate nutrition, immunization services and timely use of these services, hand washing and hygiene practices (including waste disposal and food hygiene), building the capacity of family members to appropriately take care of newborns and children under

Boston College IRB Approved
October 18, 2019
Through August 20, 2020

FOR APPROVALS WITH NO CR REQUIRED:

Boston College IRB Approved
September 4, 2020-
September 3, 2021

age 5, and building the capacity of family members to recognize and act on postnatal danger signs for newborns, mothers, and children under 5. CHWs also conduct screenings for acute malnutrition and growth monitoring to identify early referrals, and they can provide family planning methods, deworming tablets and other vitamins for acute malnutrition, dehydration, and anti-malaria treatment. Each home-visiting session lasts approximately 60 minutes.

CHWs in Sierra Leone are community based workers that help members of their own communities access health and social services. CHWs are selected by their community and trained to provide basic health services and health education. Guidelines for selection include gender parity, the ability to communicate, an interest in health, the ability to perform CHW tasks, the ability to read and write in the local language, aged 18 years or above, and physical and mental fitness to provide services. All CHWs are required to complete a 10-day standardized training program. The training program involves the following modules: (a) working with communities and households and behavior change communication; (b) water, sanitation, and hygiene; (c) maternal and newborn health; (d) infant and young child high impact preventive and treatment interventions; (e) community integrated management of newborn and childhood illnesses; (f) adolescent sexual and reproductive health rights; (g) sexual and gender-based violence. In its Policy for Community Health Workers, The Ministry of Health and Sanitation has emphasized the importance of CHWs in providing basic services to improve maternal and child health outcomes and incorporates this into the training of CHWs. Training modules related to maternal and child health include education and skills training on the following topics: the importance of antenatal care visits, skilled neonatal care, exclusive breastfeeding, early referrals and danger signs for newborns and young children, community based family planning, immunization, identification of malnutrition and low birth weight, malaria and other disease prevention and control, and integrated management of childhood illnesses,

6. Please provide more information about how families will be recruited, consented, and randomized into control and intervention groups based on non-adjacent geographical locations. For example, will all eligible families within a specific region have an equal probability of (random) selection into the intervention group? Will the selection of families within a region designated as an intervention region be based on recommendations from the CHW for that region? The application suggests that the CHW program administrator will use personal knowledge to identify likely families who are probably going to be eligible. Will the same process be used for control families?

   Randomization will occur at the level of the family. Different CHWs will provide the FSI-ECD and standard care to minimize contamination risks. We will also use a set of randomization rules to reduce the risk of contamination, such as GIS mapping to ensure nonadjacency of families. GIS mapping will consider the home address of families as well as points of potential contact, such as schools, places of worship (e.g., church, mosque), markets, and addresses of extended family members. The Program Manager and study RAs will recruit families in coordination with the CHW Focal Person, who is the Ministry of Health and Sanitation Community Health Worker Program official responsible for coordinating the work of CHWs and supervisors within peripheral health units. Peripheral Health Units are key units within the Sierra Leone healthcare system. Through smaller community-based subunits, they deliver "first line" care, including prenatal care, routine deliveries, immediate postnatal and neonatal care, community outreach services, routine vaccination, and treatment of childhood illnesses and malnutrition. Peripheral Health Units maintain records of families in the community who have sought services and will be able to identify families with a child aged 6-36 months by reviewing their records. The CHW Focal Person will generate a randomized list of all families in the target communities with children aged 6-36 months. Research Assistants will then contact potential families and explain the study using the recruitment script.

Boston College IRB
Approved
October 18, 2019
Through August 20, 2020

FOR APPROVALS WITH NO CR REQUIRED:

Boston College
IRB Approved
September 4, 2020-
September 3, 2021

The Program Manager and one RA will conduct screening and consent with families who express interest in the study. Drs. Esliker and Desrosiers will review the protocol for screening and consent with the Program Manager prior to launching recruitment. The Program Manager will administer the Screening Instrument, which includes the Difficulties in Emotion Regulation Scale (DERS), to determine eligibility. Data from the screening will not be used in the research study. The same procedure will be used to recruit, consent and enroll both intervention and control families. Eligible families within a specific community will have an equal probability of (random) selection into the intervention group.

7. More detail is needed about the screening process used to determine eligibility based on the Difficulties in Emotion Regulation Scale (DERS), which is administered using computer tablets. Is there a consent process for administering the screening tool? This will depend on whether or not the screening data will be used for research purposes. The screening appears to take place prior to study enrollment. How will families be told about the screening and, for some families, that they are not eligible (too healthy) for the study? Please provide detail on how confidentiality will be protected if there are neighboring families who are/are not eligible for the study.

   There is not a consent for screening because we will not use screening data for research purposes. Families will be told about the screening process when RAs read the recruitment script. RAs will say the following to potential families: "If you decide you want to be in our study, I will ask you to participate in the screening interview, which means that we will ask you some questions and you can answer however you want to. We will ask you questions about problems you might have, things you might feel, and things you do when you have problems or when you are feeling sad. This interview will allow us to determine if you can be a part of this study or not. Please remember that you do not have to answer any questions you do not want to. We will contact you if you are eligible to participate based on the screening interview." During the Screening interview, caregivers are reminded that their participation in the screening interview does not mean you will be enrolled in the research study, and that a member of the team will call you to let you know if they are eligible to participate. We have included the screening instrument, which includes the DERS, in our revised application.

   As part of training in Good Clinical Practice, CHWs will be trained in protocols for protection of participant privacy and confidentiality. This will include guidance about not disclosing the identity of any families who are participating to anyone outside of the research study. Study RAs will also receive detailed training in protection of privacy and confidentiality. Additionally, it is common practice for CHWs to go to the homes of families and deliver basic services at a time convenient for the family (i.e., standard services), and therefore would not be considered unusual by neighboring families. CHWs will also be trained on how to identify times that can most ensure privacy in the home and minimize potential contact with neighboring families.

8. Please clarify how you will screen for/exclude people who meet the exclusion criteria including suicidal tendencies, psychosis, ongoing divorce, etc. How are you assessing those criteria?

   We have included single items in the screening instrument to ask participants whether they are involved in an ongoing divorce process. We have also included one item that assesses suicidality. If a participant answer the question on suicidality in the affirmative, this will activate the risk of harm

Boston College IRB Approved
October 18, 2019
Through August 20, 2020

FOR APPROVALS WITH NO CR REQUIRED:

Boston College
IRB Approved
September 4, 2020-
September 3, 2021

protocol immediately. The risk of harm protocol is detailed in the response 6 in the Consent Documents Section of this letter and in the text of the application.

As part of the RA training, we will train RAs about identifying the signs and symptoms of psychosis and cognitive impairment. The Screening Instrument Section on Psychological Analysis includes questions pertaining to the interviewer's (RA's) perception of current functional status of the participant. If the RA determines that the participant shows signs of psychosis, the Mini-SCID section on psychosis will be administered. We have included the Mini-SCID in this application as well. Additionally, these before mentioned procedures have been used successfully in the BC IRB approved Youth Forward Study and R01 Diffusion and Spillover studies in Sierra Leone.

9. The compensation is inconsistent between the application and consent forms (foodstuffs, household gifts, telephone credits). If these differences are intentional, please explain the motivation. If they are not intentional, the inconsistencies should be edited.

   We have edited this to remove inconsistencies. The inconsistencies were not intentional. All documents should now read "home gift (e.g., soap)".

10. With respect to the Community Health Workers, more detail is needed about their employment status and their recruitment. More detail is needed about how the selection will be performed if there are more than enough eligible candidates. And, if there is a shortage of eligible CHWs, the question is how a decision not to participate might affect a CHW's status (the possibility of coercion).

    CHWs in Sierra Leone are community based workers that help members of their own communities access health and social services. CHWs are selected by their community and trained to provide basic health services and health education. Guidelines for selection include gender parity, the ability to communicate, an interest in health, the ability to perform CHW tasks, the ability to read and write in the local language, aged 18 years or above, and physical and mental fitness to provide services. All CHWs are required to complete a 10-day standardized training program. The training program involves the following modules: (a) working with communities and households and behavior change communication; (b) water, sanitation, and hygiene; (c) maternal and newborn health; (d) infant and young child high impact preventive and treatment interventions; (e) community integrated management of newborn and childhood illnesses; (f) adolescent sexual and reproductive health rights; (g) sexual and gender-based violence. In its Policy for Community Health Workers, The Ministry of Health and Sanitation has emphasized the importance of CHWs in providing basic services to improve maternal and child health outcomes and incorporates this into the training of CHWs.

    CHWs will be recruited using a randomized list of CHWs in the designated communities. The CHW focal person overseeing the work of CHWs in each community will provide names of potential CHWs. Study RAs will then contact CHWs based on the randomization list and read the recruitment script. This process will be continued until our target sample size is reached. If CHWs decide not to participate, this will not impact their ability to serve as CHWs in their community or their status in the community. RAs will explain this during the consent process. Because there are approximately 14,500 trained CHWs in Sierra Leone that are distributed in communities throughout the country, we anticipate that we will be able to reach our target sample size of eight CHWs and two supervisors.

Boston College IRB Approved
October 18, 2019
Through August 20, 2020

FOR APPROVALS WITH NO CR REQUIRED:

Boston College IRB Approved
September 4, 2020-
September 3, 2021

11. The terminology used throughout the application is inconsistent. For example, the application refers to focus groups as discussion sessions AND focus groups. Please make the terminology consistent throughout in order to clarify whether these are two different things.

    We have clarified our language to remove inconsistencies with our use of focus groups as discussion sessions and as focus groups. These are not two different things.

12. How will you manage a situation where a community health worker is endangered during participation in the research?

    If a community health worker is endangered during participation in research, this will also activate the safety plan. We have added this to section G.1.

Consent documents

1. The consent form only focuses on the families who will receive the intervention and does not seem appropriate for families in the control group. Please edit the form or create two consent forms - one for the intervention families and one for the control group.

   We have added language in the consent form so that it is more appropriate for control families.

2. Please provide the Boston College IRB's contact information in the consent form. The details may change as a function of which IRB is the IRB of record.

   We have included the Boston College IRB contact information in the consent form.

3. Your consent forms are missing some of the required elements such as Boston College contact information, the standard confidentiality statement about who will have access to the data, and information about how data will be stored securely. Please use our consent form template on our website to make sure you have all the required elements of consent: https://www.bc.edu/content/bc-web/research/sites/vice-provost-for-research/research-protections/forms---templates.html

   We have added the required elements that were missing based on the consent form template.

4. Please explicitly state in the consent forms how long the families will be in the study.

   We have added the following statements to the consent form "Your participation in the study will last approximately 7 months. This includes 3-4 months of participation in the family strengthening intervention and completion of a survey 3-months afterwards."

5. The application states that two parents must be present in the household to be eligible for participation and that both must consent to participate. What happens if one parent drops out of the study after enrollment? Must the entire family drop out or can a single parent continue to participate?

   Caregivers will be instructed, as part of the consenting process, that they can withdraw from the study at any time. If this occurs, study interventionists will be trained on how to probe further to understand the reason why in case we are able to address any concerns the caregiver may have

Boston College IRB
Approved
October 18, 2019
Through August 20, 2020

FOR APPROVALS WITH NO CR REQUIRED:

Boston College
IRB Approved
September 4, 2020-
September 3, 2021

about participating or correct any misinformation that may be fueling a decision to no longer participate. In the case of a dual caregiver home, if one caregiver withdraws but the other would like to continue, that is allowable and highly encouraged.

6. Please describe your plan for what to do if a family member becomes endangered during participation, whether or not the danger is due to participation.

   If a family member becomes endangered during participation, study team members will activate the safety plan. We have clarified that the safety plan will be activated regardless of whether the risk of harm is due to participation in the study in the text of our application. Detailed information about the safety plan is pasted below and appears in section G.1.

   Safety plan to identify and address risk of harm cases: Situations involving study participants in immediate risk of harm will be triaged by the study team member present at the time of interview. The Program Manager and UNIMAK RAs will discuss all risk of harm cases and report them to the PI. The PI will notify the Boston College IRB of risk of harm cases within 24 hours. Risk of harm cases will be referred to appropriate local mental health counselors and emergency facilities as needed within 24 hours. Study social workers or the Program Manager will be available to provide additional individual support and referrals to local social workers for additional mental health services. A standardized form for reporting any activation of the safety plan will be designed and all study team members will be trained in its application. For situations involving cases of suspected abuse (e.g., child abuse), the Program Manager will also contact the local Family Support Unit to report the case. All RAs will complete CITI training on research ethics and compliance and will receive extensive training on the proper protocol for recognizing and responding to risk of harm situations.

   Potential action plan responses are:

   - If suicidal ideation is reported by a participant: Research staff will conduct an immediate follow-up assessment with the participant. Staff will use scripts, which have been developed for these follow-up sessions, and include specific text for discussing mental health problems and access to services (e.g., “Based on your responses to some of these questions, I have concerns about your safety and would like to have someone on our team touch base with you”). After having this session with any participants in distress, staff will contact the Program Manager for an initial assessment of suicidal risk and will inform the PI. Trained study social workers from CARITAS will conduct this assessment to determine current suicidal ideation or psychosis. Appropriate referrals will be made to the most suitable mental health services (e.g., social work, psychology, or psychiatry) depending on level of clinical need. Referral services will be contacted if suicidal ideation is ongoing and the participant cannot be kept safe with social work and family level interventions.
   - If ongoing abuse is reported by a participant: Research staff will inform the Program Manager or PI, and if appropriate, a local authority through the current legal, health, and social services channels. If reported abuse relates to a child in the study, a report will be submitted to the Ministry of Social Welfare District Council or Family Support Unit of the Police, as required by national law. In abuse cases involving participants, the safety plan recommends informing the participants’ primary care or mental health counselors.
   - If a participant scores at very high levels of intimate partner violence or indicates ongoing intimate partner violence: Research study social workers will perform an initial evaluation, confer with the Program Manager and PI, and refer for appropriate care through the primary care counselor or mental health provider.

Boston College IRB Approved
October 18, 2019
Through August 20, 2020

FOR APPROVALS WITH NO CR REQUIRED:

Boston College IRB Approved
September 4, 2020-
September 3, 2021

- Referrals for participants in need of stage two treatment: At the time of psychosocial assessment interviews, participants will be asked questions to assess for anxiety and stress. Participants will be asked, "Do you feel you need any additional care or services due to your experiences today? Are there any questions or concerns that you would like to discuss with a mental health provider or other health professional?" Any risk of harm concerns will be discussed at this time. If needed, the counselors will provide referrals to appropriate services. All questions will be referred to Dr. Desrosiers and the Program Manager. All Safety Plan Activation Forms will be saved as an encrypted file in a password protected folder on a password protected computer managed by the Program Manager. This folder will also be saved on Box, a secure HIPAA-compliant, cloud-based platform. The study team will minimize the use of paper logs, instead utilizing computers and tablets to track participant-related study logs.

7. One particular source of risk or possible coercion should be discussed more explicitly in the consent form using examples or language that participants will be sure to understand. (e.g, "Sometimes the research team finds out from a wife's answers that her husband hits her or the child, or threatens to hit. If that happens, the research team has to tell _____ (whatever officer is told)…" It would also be helpful to comment on local laws and/or customs regarding abuse reporting. For example, whether the jurisdiction in question legally mandates reporting in ways similar to what we are accustomed to here and, if so, under what circumstances.

   We have added the above recommended language to the consent form.

8. Please provide a description of how the study design and research team will handle the potential for coercion involving, for example, disagreement between spouses about whether or not to participate, and how the core elements of consent will be repeated prior to each of three assessments, how the research team will communicate that subjects do not have to respond to any one item that makes them uncomfortable, and how the basics of the consent process will be repeated prior to each of the three assessments.

   We have added language to the caregiver consent form to address how disagreement about spouses about whether to participate will be handled, how the core elements of consent will be repeated, and how participants do not need to respond to items that make them uncomfortable. We have pasted these statements below.

   "Before each survey, we will review the basic things we discuss today and ask for your verbal consent to participate in the survey".

   "You do not have to respond to a survey item if it makes you feel upset".

   "Sometimes spouses/partners disagree about whether to participate in the study. If this happens, you can take your time to think about it. You can contact me later if you decide you would like to be in the study."

9. In the CHW Supervisor UI/UX Consent: The consent states "We are asking you to be in this study because you are a community health worker." It should be corrected to state "community health worker supervisor."

   After reviewing the CHW and CHW supervisor UI/UX consents, the error is in the title of the CHW UI/UX consent. We have removed "supervisor" from the CHW UI/UX consent form title.

Boston College IRB Approved
October 18, 2019
Through August 20, 2020

FOR APPROVALS WITH NO CR REQUIRED:

Boston College IRB Approved
September 4, 2020-
September 3, 2021

10. Once consent forms are finalized, please submit all translations in an amendment.

We will submit translated versions in an amendment once the forms have BC IRB approval.

Boston College IRB Approved
October 18, 2019
Through August 20, 2020

FOR APPROVALS WITH NO CR REQUIRED:

Boston College IRB Approved
September 4, 2020-
September 3, 2021

Protocol Title: mHealth Tools to Improve Service Delivery Quality of an Evidence-Based Family Home Visiting Intervention to Prevent Family Violence among High Risk Families in Sierra Leone

PI Name: Desrosiers, Alethea

IRB#: 21.006.01

Date: August 31, 2020

Memo: Boston College IRB Revised Application and Responses to Reviewer Queries

To Whom it May Concern:

The Purpose of this memorandum is to confirm that protocol #21.006.01 has been revised and resubmitted as per the requests of the BC IRB. Responses to the reviewer queries are detailed below. Revisions that appear in the text of the application, the consent forms, and the recruitment scripts are bolded. Thank you for your time and careful consideration of this protocol. Please do not hesitate to contact me with any further questions or concerns.

Sincerely,

Alethea Desrosiers, Ph.D.
Research Assistant Professor
Boston College School of Social Work
140 Commonwealth Ave
Chestnut Hill, MA 02467

General:

1. As discussed during the IRB meeting, please check with your in-country partners to see if there is enough bandwidth to upload audio recording files before deleting them.

Boston College IRB Approved
October 18, 2019
Through August 20, 2020

FOR APPROVALS WITH NO CR REQUIRED:

Boston College IRB Approved
September 4, 2020-
September 3, 2021

Per our conversation at the meeting, we would prefer that recordings are erased from the recorder as soon as possible after uploading to a computer.

We have confirmed that there is a possibility that bandwidth will be insufficient at times to upload recordings immediately. As such, recordings will be saved to a password protected hard drive and erased from the recorder immediately after they are saved.

2. Please confirm that research assistants will be supervised throughout the entire research process, and specify that the program manager will be the one responsible for this.

We confirm that research assistants will be supervised by the Program Manager throughout the research process.

3. In your revisions cover letter, please briefly explain who Dr. Marsh is and what their capacity on this project will be.

Dr. Marsch is a consultant on the project with expertise in incorporating technology into behavioral health interventions and facilitating "think aloud" protocols. Dr. Marsch will help guide and facilitate the "think aloud" protocol process with study participants during the user-centered design process to develop the mHealth tools.

4. Please explicitly state that a family can continue their participation in the study even if one of the caregivers decides to leave the study early.

We have explicitly stated that a family can continue their participation in the study even if one of the caregivers decides to leave the study early. This is bolded in the application.

5. Please provide an explanation of how the PI will be involved in the study and the level of oversight they will have on the project.

Dr. Desrosiers (PI) will oversee all aspects of the project, including all stages of study implementation, monitoring of study progress, and coordinating with research partners. Drs. Desrosiers and Esliker (Co-I) will provide close guidance to the Program Manager as well as overall study leadership through weekly teleconference meetings, and Dr. Esliker will lead the RA trainings with the Program Manager. Dr. Betancourt (Co-I) will join teleconference meetings to provide additional senior leadership. Pending COVID-19 travel restrictions, Dr. Desrosiers, will travel to Sierra Leone 1-2 times per year to provide additional in-person leadership and study oversight. In the event that COVID-19 travel restrictions prohibit travel to Sierra Leone, Dr. Desrosiers will hold daily check-ins with the Program Manager via WhatsApp during periods of data collection and additional teleconference meetings as needed. Given the small scope of FSI-ECD participant recruitment, the in-country Program Manager will be highly capable of carrying out the proposed study activities, with Drs. Desrosiers and Esliker providing oversight.

Boston College IRB Approved
October 18, 2019
Through August 20, 2020

FOR APPROVALS WITH NO CR REQUIRED:

Boston College IRB Approved
September 4, 2020-
September 3, 2021

6. Please revise the order of some of the scales so that PTSD measures are not given immediately before asking the caregivers to interact with their child(ren).

We have revised the order of the scales so that the PTSD measure is administered prior to the measure assessing functioning rather than immediately before asking caregivers to interact with their child(ren). We also reordered the scales so that the measurements of caregiver-child interactions and the home environment precede caregiver mental health scales.

7. Please provide a brief description of who will run the Community Advisory Board meetings and how.

The Program Manager has extensive experience overseeing Community Advisory Boards from the prior in-country work to develop the Youth Readiness Intervention as well as to support the launch of Dr. Betancourt's Longitudinal Study of War Affected Youth whereby Advisory Boards were convened to discuss and finalize study measures and protocols related to participant tracking, data collection, and compensation.
As such, the Program Manager will run the Community Advisory Board meetings. The Program Manager will schedule and attend all Community Advisory Board meetings in person and facilitate group discussions to obtain feedback on FSI-ECD adaptation. A research assistant will accompany the Program Manager to take notes during meetings.

8. Please revise your materials (consent, recruitment, etc) to reflect a general name such as "caregiver" instead of "parent" or "mother/father" as the family caregivers may not be mothers and fathers.

We have revised all materials so that the term “caregiver” is consistent.

Boston College IRB
Approved
October 18, 2019
Through August 20, 2020

FOR APPROVALS WITH NO CR REQUIRED:

Boston College
IRB Approved
September 4, 2020-
September 3, 2021

CITI PROGRAM

Completion Date 26-Apr-2018
Expiration Date 25-Apr-2021
Record ID 26828902

This is to certify that:

Musu Jambai

Has completed the following CITI Program course:

**Human Research**	(Curriculum Group)
**Social/Behavioral Research Course**	(Course Learner Group)
**1 - Basic Course**	(Stage)

Under requirements set by:

Boston College

CITI
Collaborative Institutional Training Initiative

Boston College IRB Approved
October 18, 2019
Through August 20, 2020

Verify at www.citiprogram.org/verify/?w66654109-d084-411f-9e0c-eef5be6ba53e-26828902

FOR APPROVALS WITH NO CR REQUIRED:

Boston College IRB Approved
September 4, 2020-
September 3, 2021

CITI PROGRAM

Completion Date	17-Apr-2018
Expiration Date	16-Apr-2021
Record ID	26828893

This is to certify that:

Mahmoud Feika

Has completed the following CITI Program course:

**Human Research**	(Curriculum Group)
**Social/Behavioral Research Course**	(Course Learner Group)
**1 - Basic Course**	(Stage)

Under requirements set by:

Boston College

CITI

Collaborative Institutional Training Initiative

Boston College IRB Approved
October 18, 2019
Through August 20, 2020

Verify at www.citiprogram.org/verify/?wf91cf3a2-8d6c-49cc-b2bc-54d9f375c9fd-26828893

FOR APPROVALS WITH NO CR REQUIRED:

Boston College IRB Approved
September 4, 2020-
September 3, 2021

CITI PROGRAM

Completion Date 01-Aug-2020
Expiration Date 01-Aug-2023
Record ID 37669504

This is to certify that:

Julia Monica Bangura

Has completed the following CITI Program course:

Not valid for renewal of certification through CME. Do not use for TransCelerate mutual recognition (see Completion Report).

Human Research (Curriculum Group)
Social/Behavioral Research Course (Course Learner Group)
1 - Basic Course (Stage)

Under requirements set by:

Boston College

CITI
Collaborative Institutional Training Initiative

Boston College IRB Approved
October 18, 2019
Through August 20, 2020

Verify at www.citiprogram.org/verify/?w93b43344-83bc-4363-9fca-242b7479a572-37669504

FOR APPROVALS WITH NO CR REQUIRED:

Boston College IRB Approved
September 4, 2020-
September 3, 2021

CITI PROGRAM

Completion Date 01-Aug-2020
Expiration Date 01-Aug-2023
Record ID 37669053

This is to certify that:

Medrine Sama Koroma

Has completed the following CITI Program course:

Not valid for renewal of certification through CME. Do not use for TransCelerate mutual recognition (see Completion Report).

**Human Research**	(Curriculum Group)
**Social/Behavioral Research Course**	(Course Learner Group)
**1 - Basic Course**	(Stage)

Under requirements set by:

Boston College

CITI
Collaborative Institutional Training Initiative

Boston College IRB Approved
October 18, 2019
Through August 20, 2020

Verify at www.citiprogram.org/verify/?wbb9a3f37-12bc-415f-aac1-9e9cfc945b2c-37669053

FOR APPROVALS WITH NO CR REQUIRED:

Boston College IRB Approved
September 4, 2020-
September 3, 2021

CITI PROGRAM

Completion Date 31-Jul-2020
Expiration Date 31-Jul-2023
Record ID 37668642

This is to certify that:

Abdulrahman Jimmy

Has completed the following CITI Program course:

Not valid for renewal of certification through CME. Do not use for TransCelerate mutual recognition (see Completion Report).

**Human Research**	(Curriculum Group)
**Social/Behavioral Research Course**	(Course Learner Group)
**1 - Basic Course**	(Stage)

Under requirements set by:

Boston College

CITI
Collaborative Institutional Training Initiative

Boston College IRB Approved
October 18, 2019
Through August 20, 2020

Verify at www.citiprogram.org/verify/?wb7a103a4-a02d-4ac3-bcb9-3f3c0261d788-37668642

FOR APPROVALS WITH NO CR REQUIRED:

Boston College IRB Approved
September 4, 2020-
September 3, 2021

CITI PROGRAM

Completion Date 31-Jul-2020
Expiration Date 31-Jul-2023
Record ID 37644651

This is to certify that:

Abraham Isiaka Jimmy

Has completed the following CITI Program course:

Not valid for renewal of certification through CME. Do not use for TransCelerate mutual recognition (see Completion Report).

**Human Research**	(Curriculum Group)
**Social/Behavioral Research Course**	(Course Learner Group)
**1 - Basic Course**	(Stage)

Under requirements set by:

Boston College

CITI
Collaborative Institutional Training Initiative

Boston College IRB Approved
October 18, 2019
Through August 20, 2020

Verify at www.citiprogram.org/verify/?w597707e1-a8c3-4819-b36e-24a32d5e9233-37644651

FOR APPROVALS WITH NO CR REQUIRED:

Boston College IRB Approved
September 4, 2020-
September 3, 2021
